# GGA1 interacts with the endosomal Na+/H+ exchanger NHE6 governing localization to the endosome compartment

**DOI:** 10.1016/j.jbc.2024.107552

**Published:** 2024-07-11

**Authors:** Li Ma, Ravi Kiran Kasula, Qing Ouyang, Michael Schmidt, Eric M. Morrow

**Affiliations:** 1 Department of Molecular Biology, Cell Biology and Biochemistry, Brown University, Providence, Rhode Island, USA; 2 Center for Translational Neuroscience, Brown University, Providence, Rhode Island, USA

**Keywords:** exchangers, endosome, intracellular trafficking, lysosome, Golgi, Na+/H+ exchanger 6 (NHE6), Golgi-associated, gamma adaptin ear containing, ARF binding protein 1 (GGA1), Christianson syndrome (CS)

## Abstract

Mutations in the endosomal Na+/H+ exchanger 6 (NHE6) cause Christianson syndrome, an X-linked neurological disorder. NHE6 functions in regulation of endosome acidification and maturation in neurons. Using yeast two-hybrid screening with the NHE6 carboxyl terminus as bait, we identify Golgi-associated, gamma adaptin ear-containing, ADP-ribosylation factor (ARF) binding protein 1 (GGA1) as an interacting partner for NHE6. We corroborated the NHE6-GGA1 interaction using: coimmunoprecipitation; overexpressed constructs in mammalian cells; and coimmunoprecipitation of endogenously expressed GGA1 and NHE6 from neuroblastoma cells, as well as from the mouse brain. We demonstrate that GGA1 interacts with organellar NHEs (NHE6, NHE7, and NHE9) and that there is significantly less interaction with cell-surface localized NHEs (NHE1 and NHE5). By constructing hybrid NHE1/NHE6 exchangers, we demonstrate the cytoplasmic tail of NHE6 interacts most strongly with GGA1. We demonstrate the colocalization of NHE6 and GGA1 in cultured, primary hippocampal neurons, using super-resolution microscopy. We test the hypothesis that the interaction of NHE6 and GGA1 functions in the localization of NHE6 to the endosome compartment. Using subcellular fractionation experiments, we show that NHE6 is mislocalized in GGA1 KO cells, wherein we find less NHE6 in endosomes, but more NHE6 transport to lysosomes, and more Golgi retention of NHE6, with increased exocytosis to the surface plasma membrane. Consistent with NHE6 mislocalization, and Golgi retention, we find the intraluminal pH in Golgi to be alkalinized in GGA1-null cells. Our study demonstrates a new interaction between NHE6 and GGA1 which functions in the localization of this intracellular NHE to the endosome compartment.

Loss-of-function mutations in the X-linked endosomal Na+/H+ exchanger 6 (NHE6, encoded by the SLC9A6 gene) cause the neurological disorder Christianson syndrome (CS) (1, 2, 3, 4, 5, 6, 7, 8, 9, 10). In mammalian cells, NHE6, NHE7, NHE8, and NHE9 are generally considered to be intracellular, with NHE6 and NHE9 studied as endosomal NHEs (4, 11, 12, 13, 14, 15). On the other hand, NHE1, NHE2, NHE3, NHE4, and NHE5 are reported to be cell-surface NHEs (15, 16). NHEs are evolutionary-conserved transmembrane proteins that regulate the electroneutral exchange of H+ ions with Na+ or K+ ions (17, 18). NHE proteins are composed of a conserved 12 membrane-spanning domain in the N terminus encompassing the NHE, and a less-conserved cytoplasmic C terminus that is involved in protein binding (14). They regulate a range of critical physiological functions including cytosolic/organellar pH, cell volume, acid-base homeostasis, as well as broader cellular functions, such as intracellular trafficking and posttranslational modification (14). Mutations of some plasma membrane NHEs are associated with neural dysfunction (14, 19, 20, 21, 22, 23, 24, 25). Also, NHE5 has been shown to modulate synaptic plasticity by negatively regulating activity-dependent dendritic spine growth (26). Prior studies have demonstrated that loss of NHE6 leads to overacidification of endosomes, as well as defects in endosome maturation and lysosome function (4, 8, 27); however, the cellular mechanisms of disease in CS, and the function of NHE6 in endosome maturation are incompletely understood.

Protein-protein interactions provide important clues for cellular functions; however, very few NHE6-interacting proteins are known. Cytosolic domains of the endosomal NHE6 and NHE9 are reported to bind the scaffold protein RACK1, and the NHE6-Rack1 interaction is proposed to control receptor recycling in cultured cells (28). NHE6 also directly interacts with secretory carrier membrane protein 5 (SCAMP5), and this SCAMP5-dependent recruitment of NHE6 to synaptic vesicles plays a critical role in manifesting presynaptic efficacy both at rest and during synaptic plasticity (29, 30).

Here, we used yeast two-hybrid screening to find new NHE6 interacting partners. We found that Golgi-associated, gamma adaptin ear-containing, ARF binding protein 1 (GGA1), a member of the GGA protein family (31, 32, 33, 34, 35, 36, 37, 38, 39, 40, 41), is a new NHE6-interacting protein. The NHE6 cytoplasmic domain plays the main role in GGA1 interaction. GGA1 also binds strongly to other intracellularly localized NHEs, including NHE7 and NHE9, but significantly less to plasma membrane localized NHE1 and NHE5. GGA proteins are monomeric clathrin adaptors that package and traffic cargo from the trans-Golgi network (TGN) to the endocytic pathway, and also mediate retrograde trafficking from the endocytic pathway to the TGN (34, 36, 42, 43, 44, 45, 46, 47, 48). There are three members of the mammalian GGA family (GGA1-3), that contain (1) an N-terminal VHS domain that binds proteins with an acidic di-leucine signal like mannose 6-phosphate receptors, (2) a GAT domain that binds Arf:GTP, (3) a connecting Hinge linker region that recruits clathrin, and (4) a C-terminal GAE domain that recruits accessory proteins (45, 49). GGAs function in trafficking cation-dependent and cation-independent mannose 6-phosphate receptors, which are critical for delivering newly synthesized precursor lysosomal enzymes to the endocytic pathway where they will ultimately participate in the degradation of cellular materials in acidic lysosomes (38, 41, 48, 50, 51, 52). Here, we demonstrate that the interaction of GGA1 with NHE6 functions in the localization of NHE6 to the endosome compartment, and in the absence of GGA1, NHE6 is mislocalized, including retention in the Golgi leading to intralumen alkalinization and increased exocytosis of NHE6 to the cell surface membrane.

## Results

### Identification of GGA1 as an NHE6-interacting partner

**Figure 1:**
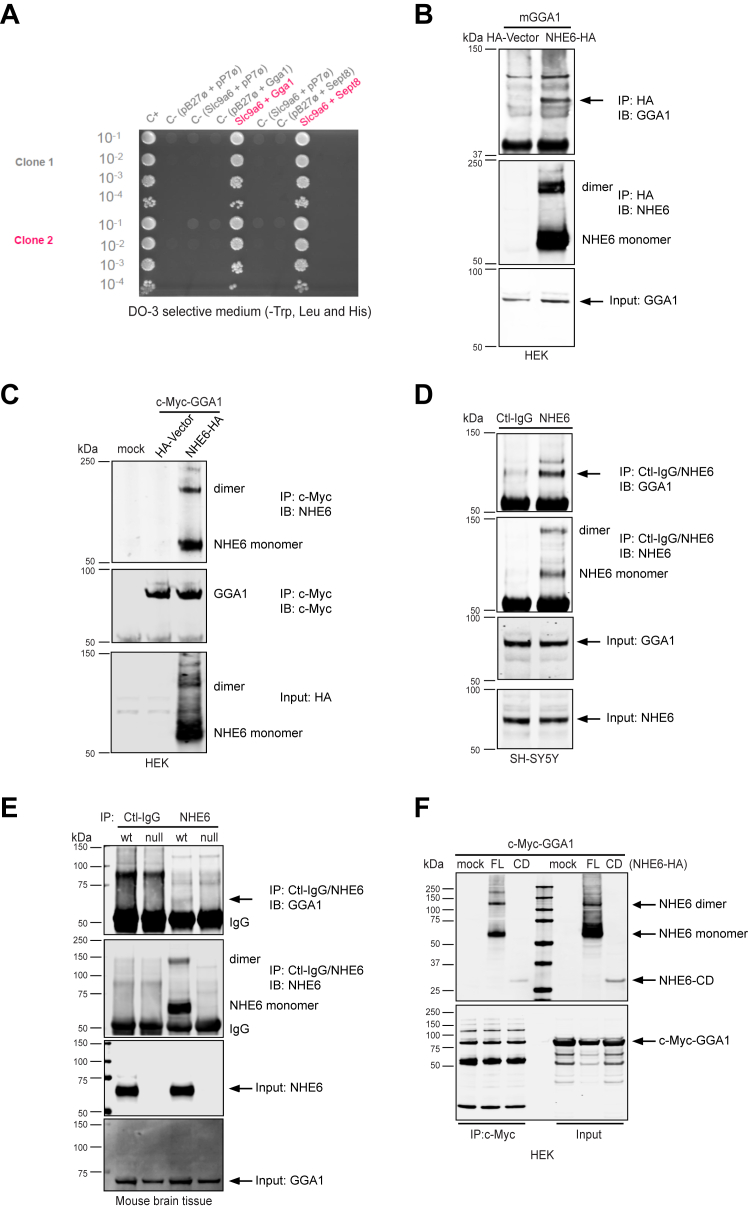
**Identification of GGA1 as a new NHE6 interacting partner**. *A*, yeast cells containing bait construct pB27-NHE6 (N-LexA-NHE6-537–630, amino acids 537–630 of *Slc9a6* cloned into the pB27 plasmid hgx2030v2_pB27) and prey constructs: pP6-GGA1 (N-GAL4-GGA1, clone RHC_RP_hgx2030v2_pB27_C-38 cloned into pP6) or pP6-Sept8 (N-GAL4-Sept8, clone RHC_RP_hgx2030v2_pB27_B-2 cloned into pP6). Yeast cells were obtained by mating and spotted, at the dilutions indicated, on DO-3 selective media lacking Trp, Leu, and His. Negative controls included the following empty bait or prey vectors: pB27 and pP7, or pB27+Gga1, pB27+Sept8, Slc9a6+pP7. *B*, cell lysates from HEK293T cells expressing mGGA1 with either NHE6-HA or HA-vector were immunoprecipitated with anti-HA antibody. The precipitates were analyzed with both anti-GGA1 and anti-NHE6 blotting. Total cell lysates (TCLs) were subjected to Western blot analysis with anti-GGA1 antibody. All samples were separated by 4 to 12% SDS–PAGE unless mentioned separately. *C*, cell lysates from HEK293T cells expressing c-Myc-GGA1 with either NHE6-HA or HA-vector were immunoprecipitated with anti-c-Myc antibody. The precipitates were probed with anti-NHE6 and c-Myc antibodies. TCLs were subjected to Western blot analysis with anti-HA to detect the expression of NHE6. *D*, Co-IP assay in SH-SY5Y neuroblastoma cells to detect interaction between endogenous NHE6 and GGA1. TCLs from SH-SY5Y cells were precipitated by anti-NHE6 antibody, with normal rabbit IgG (Santa Cruz, sc-2027) used as a control. The precipitates were subjected to Western blot analysis using anti-GGA1 and anti-NHE6 antibodies. TCLs were subjected to Western blot analysis with anti-NHE6 and anti-GGA1 to detect the expression level of endogenous NHE6 and GGA1. *E*, brain tissue from NHE6-null and WT males at postnatal day 0 (P0) was collected and homogenized. TCLs were precipitated by anti-NHE6 antibody, with normal rabbit IgG (Sigma-Aldrich 12–370) used as a control. The precipitates were subjected to Western blot analysis using both anti-GGA1 and anti-NHE6 antibodies. TCLs were subjected to Western blot analysis with anti-NHE6 and anti-GGA1 antibodies to detect the expression level of GGA1 and NHE6. *F*, TCLs from HEK293T cells expressing c-Myc-GGA1 with either HA-tagged full-length NHE6 (NHE6-FL) or cytoplasmic domain NHE6 (NHE6-CD) were immunoprecipitated with anti-c-Myc antibody. The precipitates were probed with HA and c-Myc antibodies. TCLs were subjected to Western blot analysis with anti-HA or c-Myc antibodies to detect the expression of NHE6-FL or NHE6-CD and c-Myc-GGA1. GGA1, Golgi-associated, gamma adaptin ear-containing, ARF binding protein 1; NHE6, Na+/H+ exchanger 6; Co-IP, coimmunoprecipitation; Sept8, Septin8; IgG, immunoglobulin G.

A yeast two-hybrid screen was performed using the mouse NHE6 cytoplasmic tail amino acids Leu^537^-Asp^630^ (NP_766368.2) as bait to discover possible NHE6-interacting proteins from a random-primed rat hippocampus complementary DNA (cDNA) library. A total of 53.8 million cDNA clones were screened and 123 His+ colonies were selected on DO-3 medium lacking tryptophan, leucine, and histidine. Among these 123 colonies, sequence analyses revealed that 13 colonies coded for the GGA1 (Table S1). All GGA1 sequences showed the maximally high confidence Predicted Biological Score (PBS). Another 24 clones coded for Septin8 (Sept8) which also showed very high confidence scores; however, Sept8 failed to show an interaction with NHE6 in the follow-up immunoprecipitation (IP) analysis and was not studied further. As shown in Figure 1*A*, NHE6 with either GGA1 (lane 5) or Sept8 (lane 8) in bait-prey combination allowed growth on DO-3 medium, while negative controls containing empty bait and prey vectors, pB27 and pP7 (lane 2), or pB27 and pP7 in combination with prey and bait molecules (lanes 3, 4, 6, and 7) did not grow on this medium. These results from yeast two-hybrid screening suggest that GGA1 is a new interaction partner for NHE6.

IP analysis was then used to confirm this NHE6-GGA1 interaction in mammalian cells. To test whether NHE6 interacts with GGA1 in mammalian cells, HEK293T cells were cotransfected with pCMV-mGGA1 and either pNHE6-HA or hemagglutinin (HA)-vector. Cell lysates were immunoprecipitated with anti-HA antibody to pull down NHE6 and subjected to Western blot analysis to detect bound GGA1 using anti-GGA1 antibody. As shown in Figure 1*B*, GGA1 was detected in anti-HA immunoprecipitates from cells expressing GGA1 and NHE6, but not from cells expressing GGA1 and HA vector, indicating NHE6 is able to pull down and interact with GGA1.

We then tested the reciprocal coimmunoprecipitation (co-IP), *i.e.* whether GGA1 could pulldown NHE6. HEK293T cells were cotransfected with pCMV/c-Myc-GGA1 and either pNHE6-HA or HA-vector. Cell lysates were immunoprecipitated with anti-c-Myc antibody to pull down GGA1 and subjected to Western blot analysis to detect bound NHE6 using a custom-made anti-NHE6 antibody (4). As shown in Figure 1C, NHE6 was detected in anti-c-Myc immunoprecipitates from cells expressing GGA1 and NHE6, but not from cells expressing GGA1 and HA-vector, indicating GGA1 is able to coprecipitate and interact with NHE6.

To examine whether this interaction between NHE6 and GGA1 could be detected endogenously without overexpression of constructs, SH-SY5Y neuroblastoma cell lysates were precipitated with either anti-NHE6 antibody or control-immunoglobulin G (IgG), followed by immunoblotting with anti-GGA1 antibody. GGA1 was detected in immunoprecipitates from NHE6 precipitates, but not control-IgG precipitates (Fig. 1*D*). This result supports a physical interaction between NHE6 and GGA1 in endogenously expressed proteins.

Since NHE6 is highly expressed in the brain (4), we then investigated the NHE6-GGA1 interaction in cortical brain tissue from NHE6-null and WT littermate controls at postnatal day 0 (P0). Homogenized cortical brain lysates were immunoprecipitated with anti-NHE6 antibody or control-IgG, followed by immunoblotting with anti-GGA1 antibody. In WT cortical tissue, GGA1 was detected in anti-NHE6 precipitates, but not control-IgG precipitates (Fig. 1*E*). Further, GGA1 was not detected in NHE6-null tissue (Fig. 1*E*). In the input, NHE6 protein was only detected in NHE6 WT samples but not in NHE6-null, and the GGA1 protein was expressed in both NHE6 WT and NHE6-null samples. (Fig. 1*E*, third and fourth panel). This result further demonstrates that NHE6 interacts with GGA1 in mouse brain tissue. To further confirm the NHE6 carboxyl terminus alone can interact with GGA1 in mammalian cells, HEK293T cells were cotransfected with pCMV/c-Myc-GGA1 and either a full-length NHE6 (pNHE6-FL-HA) or cytoplasmic domain (CD, pNHE6-CD-HA) construct. Cell lysates were immunoprecipitated with anti-c-Myc antibody to pull down GGA1 and then subjected to Western blot analysis to detect bound NHE6 using anti-HA antibody. Both full-length and CD NHE6 products were detected in immunoprecipitated anti-c-Myc, indicating the NHE6 cytoplasmic domain alone can interact with GGA1 (Fig. 1*F*).

### GGA1 shows strong interaction with organellar NHEs (NHE6/7/9) but not with cell surface localized NHEs (NHE1/5)

To determine whether GGA1 solely interacts with NHE6 or interacts with other NHEs, we tested the extent to which plasma membrane (*e.g.* NHE1 and NHE5) and organellar (NHE6, NHE7, and NHE9) NHEs interact with GGA1 (Fig. S1, *A*–*C*). NHE5, NHE7, and NHE9 were all constructed with an HA-tag in the pReceiver-M07 vector, and expression was confirmed by both HA (Fig. S1*A*) and NHE-specific antibodies (Fig. S1*B*). We then immunoprecipitated HEK293T cells that were cotransfected with pCMV/c-Myc-GGA1 and HA-tagged NHEs (NHE5, NHE6, NHE7, or NHE9). Like NHE6 (Fig. S1*C*, top panel, lane 2), organellar NHE7 and NHE9 (Fig. S1*C*, top panel, lanes 4 and 5) interact with GGA1. The plasma membrane NHE5 had dramatically reduced potential for interaction with GGA1 (Fig. S1*C*, top panel, lane 3). This indicates that GGA1 preferentially interacts with organellar NHEs compared to the plasma membrane NHE5.

**Figure 2:**
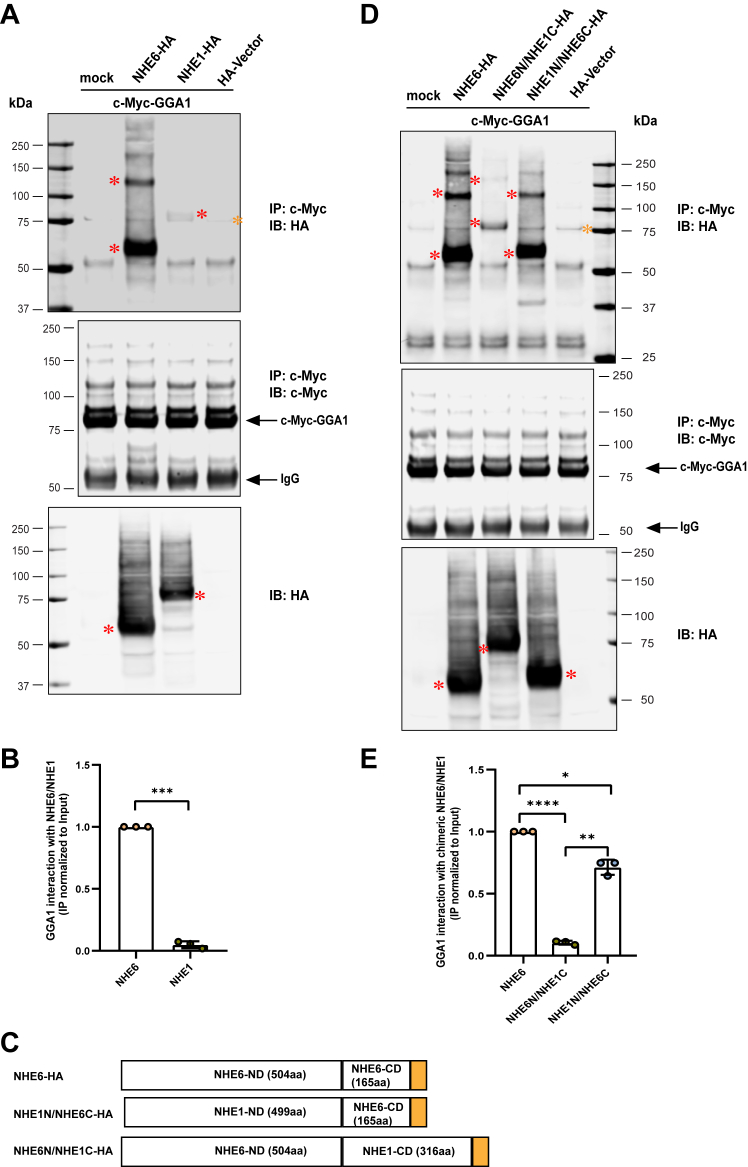
**GGA1 interacts strongly with the NHE6 C-term but not with the NHE1 C-term**. *A*, total cell lysates (TCLs) from HEK293T cells expressing c-Myc-GGA1 with either NHE6-HA or NHE1-HA were immunoprecipitated with anti-c-Myc antibody. NHE1-HA used here was mutagenesis (G720D) corrected hNHE1-HA. Control (only c-Myc-GGA1) and HA-vector (HA-vector and c-Myc-GGA1 cotransfectants) were used as control. The precipitates were probed with HA and c-Myc antibodies. TCLs were subjected to Western blot analysis with the anti-HA antibody to detect the expression of NHE6 and NHE1. *Red asterisks* indicate NHE6 (monomer and dimer) and NHE1 band, *orange asterisk* indicates a nonspecific band. *B*, quantification of GGA1 interaction with NHE6 and NHE1 showing a normalization of immunoprecipitated products with corresponding inputs (mean ± SD; n = 3; ∗∗∗*p* = 0.0003; unpaired two-tailed Student’s *t* test with Welch’s correction). *C*, domain schematics of HA-tagged NHE6, and chimeric snap constructs NHE1-N terminus+NHE6-C terminus (NHE1N/NHE6C) and NHE6-N terminus+NHE1-C terminus (NHE6N/NHE1C). *D*, TCLs from HEK293T cells expressing c-Myc-GGA1 with HA-tagged NHE6, NHE1N/NHE6C, and NHE6N/NHE1C were immunoprecipitated with anti-c-Myc antibody. Control (only c-Myc-GGA1) and HA-vector (HA-vector and c-Myc-GGA1 cotransfectants) were used as control. The precipitates were probed with HA and c-Myc antibodies. TCLs were subjected to Western blot analysis with anti-HA to detect the expression of NHE6 and chimeric snap constructs. *Red asterisks* indicate NHE6 and chimeric NHE6/NHE1 band (monomer and dimer), *orange asterisk* indicates a nonspecific band. *E*, quantification of GGA1 interaction with NHE6, NHE6N/NHE1C and NHE1N/NHE6C showing as normalization of immunoprecipitated products with corresponding inputs (mean ± SD; n = 3; ∗∗∗∗*p* < 0.0001 for NHE6 *versus* NHE6N/NHE1C; ∗*p* = 0.0146 for NHE6 *versus* NHE1N/NHE6C; ∗∗*p* = 0.0021 for NHE6N/NHE1C *versus* NHE1N/NHE6C; unpaired two-tailed Student’s *t* test with Welch’s correction). GGA1, Golgi-associated, gamma adaptin ear-containing, ARF binding protein 1; NHE6, Na+/H+ exchanger 6; IgG, immunoglobulin G; Ha, hemagglutinin.

We also examined whether GGA1 interacts with the plasma membrane localized NHE1. We detect a weak interaction between GGA1 and NHE1 (Fig. 2, *A* and *B*), while the binding is notably lower compared to NHE6 (quantified in Fig. 2*B*). To further investigate the NHE domain of interaction with GGA1, we then generated two NHE6-NHE1 chimeric constructs by using the in-fusion snap method (Takara), that swaps the N terminus exchanger domain and C-term cytoplasmic tail between NHE1 and NHE6 (Fig. 2*C* and Table S2). As shown in Figure 2, *D* and *E*, the NHE6 C terminus with the NHE1 N terminus (NHE1N/NHE6C) maintains a strong interaction with GGA1 (Fig. 2*D* lane 4 *versus* lane 2, Fig. 2*E*). However, the NHE1 C terminus with the NHE6 N terminus (NHE6N/NHE1C) dramatically reduces the interaction with GGA1 (Fig. 2*D* lane 3 *versus* lane 2, Fig. 2*E*). These findings support the interpretation that the NHE6 C-term is both necessary, and largely sufficient, for the strongest interaction with GGA1.

To determine the extent to which NHE interaction is unique to GGA1, we performed IP experiments in GGA3. Specifically, we pulled down GGA3 and examined its interaction with either NHE6 or NHE9 in coexpression studies in HEK293T cells. Interestingly, as shown from Fig. S2*A*, both NHE6 and NHE9 were coimmunoprecipitated by GGA3 (lanes 2 and 3). Also, GGA3 and GGA1 could form a heterodimer (lane 4). Thus, our results suggest a broad interaction between GGAs and endosomal NHE family members.

### GGA1 GAE domain shows the strongest interaction with NHE6

**Figure 3:**
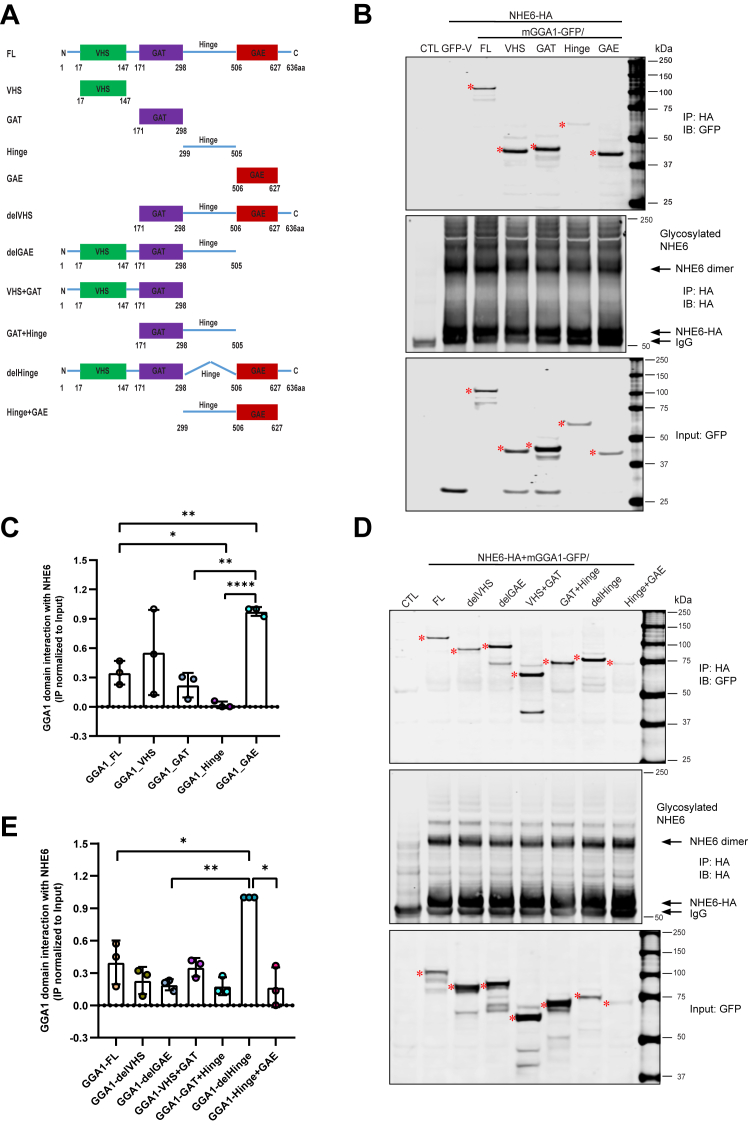
**Mapping of interaction regions between NHE6 and GGA1**. *A*, structure of mGGA1 domains: VHS (aa(17–147), *green*), GAT (aa(171–298), *purple*), GAE (aa(506–627), *red*), Hinge (aa(299–505)), del GAE (aa(1–505)), del VHS (aa(171–635)), VHS + GAT (aa(1–298)), Hinge+GAE (aa(299–627)), GAT+Hinge (aa(171–505)), and del Hinge (del aa(299–505)). *B* and *D*, GFP-tagged GGA1 construct mGGA1-FL/VHS/GAT/Hinge/GAE (*B*), or GFP-tagged GGA1 construct mGGA1-FL/delVHS/delGAE/VHS+GAT/GAT+Hinge/delHinge/Hinge+GAE (*D*) was cotransfected with NHE6-HA in HEK293T cells. Anti-HA immunoprecipitates were analyzed with anti-GFP immunoblotting to identify GGA1 region(s) necessary for NHE6 interaction. TCLs were subjected to Western blot analysis with anti-GFP to detect the expression level of GGA1 constructs. *Red asterisks* indicate GGA1 FL/domains bands. *C* and *E*, quantification of GGA1 domain interaction with NHE6 showing as normalization of immunoprecipitated products with corresponding inputs. (mean ± SD; n = 3; ∗*p* < 0.05, ∗∗*p* < 0.01, ∗∗∗∗*p* < 0.0001; Unpaired two-tailed Student’s *t* test with Welch’s correction). GGA1, Golgi-associated, gamma adaptin ear-containing, ARF binding protein 1; HA, hemagglutinin; NHE6, Na+/H+ exchanger 6; TCL, total cell lysate.

GGA1 is composed of a VHS domain, GAT domain, GAE domain, and Hinge linker domain in between GAT and GAE (31, 32, 53, 54, 55). To identify the region of GGA1 that mediates the interaction with NHE6, we constructed a series of domains (VHS, GAT, GAE, and Hinge) (Fig. 3*A*) to compare the ability to bind NHE6 by co-IP assay. As shown in Figure 3*B*, all GGA1 single domains interact with NHE6, except the Hinge domain that demonstrated an extremely low interaction (Fig. 3*C*). The GAE domain showed the strongest binding activity with NHE6 (Fig. 3*C*). To confirm the weak interaction of the Hinge domain and the strong interaction of GAE with NHE6, we generated more GGA1 domain constructs (including, delGAE, delVHS, VHS + GAT, Hinge+GAE, GAT+Hinge and delHinge, Fig. 3*A*). All of these domains interacted with NHE6, and deletion of the Hinge region (delHinge) showed the strongest interaction with NHE6 (Fig. 3, *D* and *E*). Therefore, the Hinge domain may function as a binding interfering domain wherein this domain weakens the GGA1-NHE6 interaction. Compared to delGAE (VHS + GAT+Hinge), delHinge has a stronger binding activity further supporting the Hinge linker’s negative function, which also provides additional support for the strongest binding activity function of GAE domain as shown in Figure 3*C*. Although GAE shows the strong binding activity (Fig. 3, *B* and *C*), Hinge+GAE dramatically reduced the binding activity, further indicating that the Hinge domain may function as a binding interfering domain.

### Colocalization of NHE6 and GGA1 in neurons using super-resolution microscopy

**Figure 4:**
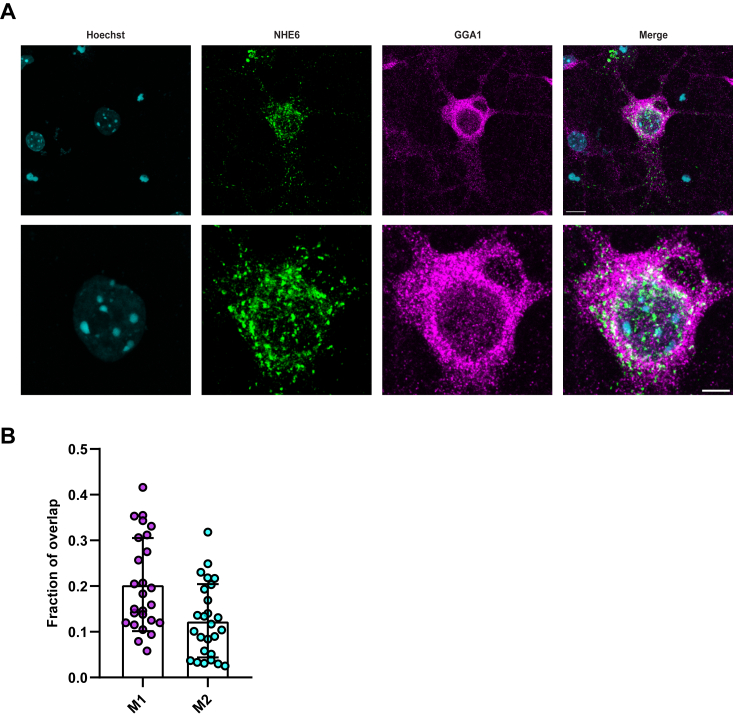
**NHE6 and GGA1 colocalization using confocal microscopy in mouse neurons *in vitro***. *A*, representative image of mouse primary hippocampal neurons at 14 days *in vitro* (14 DIV) were costained with anti-NHE6 (*green*) and anti-GGA1 (*magenta*) antibodies. Hoechst = *blue*. The *bottom panel* is a zoomed in of the *top panel*. The scale bar represents *top panel* = 10 μm; *bottom* panel=5 μm. *B*, quantification of NHE6 and GGA1 colocalization by Mander’s coefficients. M1 = fraction of NHE6 overlapping GGA1; M2 = fraction of GGA1 overlapping NHE6 (n = 26 images from three WT mice hippocampal cultures, mean ± SD). M1 and M2 are plotted as *magenta* and *blue* color, respectively. GGA1, Golgi-associated, gamma adaptin ear-containing, ARF binding protein 1; NHE6, Na+/H+ exchanger 6.

The finding that NHE6 interacts with GGA1 led us to explore where the colocalization of these two proteins occurs in cells such as neurons. NHE6 mainly localizes in early, recycling, and late endosomes (LEs) (4, 12, 56). In dissociated hippocampal neurons *in vitro*, NHE6 localization is punctate, adjacent to Golgi apparatus and distributed throughout the cell (4). Mouse hippocampal neurons were then stained for NHE6 and GGA1 using immunocytochemistry and initially imaged using confocal microscopy (Fig. 4*A*). (Demonstration of specificity of the antibodies is shown in Fig. S3.) As shown in Figures 4*A*, NHE6 and GGA1 colocalize to discrete punctae in the perinuclear region. Further Mander’s colocalization analysis showed the fraction of NHE6 overlapping with GGA1 (M1) is 0.2034 ± 0.1019 and the fraction of GGA1 overlapping with NHE6 (M2) is 0.1240 ± 0.08020. (Fig. 4*B*).

**Figure 5:**
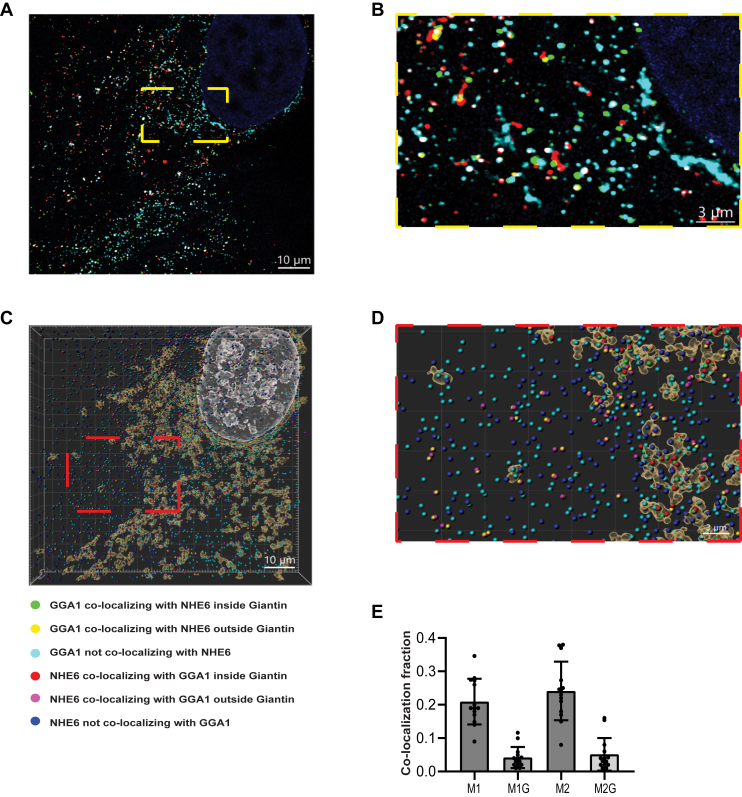
**Colocalization of NHE6 and GGA1 by 5x expansion microscopy imaging in primary neurons *in vitro*.** Colocalization of GGA1 and NHE6 with respect to Golgi in rat hippocampal neurons: (*A*) shows a single frame of 5× expanded rat hippocampal neuron on DIV 14 costained for nucleus (*blue*), Giantin (*cyan*), GGA1 (*red*), and NHE6 (*green*) from a z-stack acquired using confocal microscopy. Corrected scale bar represents ∼2.27 μm. The intensities were adjusted for visualization. *B*, enlarged image from (*A*) shown in *yellow box*. The corrected scale bar represents ∼0.68 μm. *C*, shows the 3D reconstruction of the z-stack using IMARIS surface tools for the nucleus (*white*), and Giantin (*brown*) and spot classification for GGA1 (*green*, *yellow*, or *cyan*), and NHE6 (*red*, *pink*, or *blue*). The spots are classified based on the colocalization of a GGA1 or NHE6 with the other stain. The corrected scale bar represents ∼2.27 μm. *D*, enlarged image from (*C*) shown in *red box*. The corrected scale bar represents ∼0.68 μm. *E*, colocalization fraction of GGA1 or NHE6 analyzed using IMARIS software. M1: fraction of GGA1 colocalizing with NHE6; M1G: fraction of colocalizing GGA1 with NHE6 inside Giantin; M2: fraction of NHE6 colocalizing with GGA1; M2G: fraction of colocalizing NHE6 with GGA1 inside Giantin. The color code for (*C* and *D*) are described to the right of image (n = 14 cells from four unique neuronal cultures). The corrected scale bar refers to scale correction after accounting for physical expansion of the cells, based on Fig. S5*A*. GGA1, Golgi-associated, gamma adaptin ear-containing, ARF binding protein 1; NHE6, Na+/H+ exchanger 6.

Importantly, to attain a higher resolution of colocalized puncta, we also used a 5× expansion microscopy technique (57) to acquire multicolor z-stack super-resolution images of rat hippocampal neurons, colabeled with NHE6, GGA1, and the Golgi marker Giantin (Fig. 5, *A* and *B*). We optimized the antibody dilution to ensure the specificity of antibodies in the expansion gel (Fig. S4). As shown in the high-resolution image in Figure 5*B*, we are able to visualize NHE6 (green) and GGA1 (red) colocalization, demonstrated as yellow puncta (corrected scale bar: approximately 2.27 μm). We created surfaces for Giantin and spots for NHE6 and GGA1 (Fig. 5, *C* and *D*) for quantification. From the shortest distance of these spots, and their proximity to Giantin surfaces, we classified the NHE6 and GGA1 spots into six different categories (Fig. 5, *C* and *D*). Expansion factor is measured, and the average shortest distance values are shown in Fig. S5. Based on the number of spots in each category, we found that a fraction of 0.2094 ± 0.06840 (mean ± SD) GGA1 spots colocalize with NHE6. Approximately, 0.04189 ± 0.03159 (mean ± SD) NHE6/GGA1 colabeled spots are within Giantin (Fig. 5*E*). About 0.2412 ± 0.08785 (mean ± SD) NHE6 spots colocalize with GGA1, and approximately 0.05129 ± 0.04874 (mean ± SD) NHE6/GGA1 colabeled spots are within Giantin (Fig. 5*E*). These expansion results further confirm NHE6 and GGA1 colocalization using high-resolution microscopy techniques.

### GGA1 functions to stabilize NHE6 in the endosome compartment

To elucidate the functional importance of the interaction between NHE6 and GGA1, we established two GGA1-null subclonal cell lines that were gene-edited to KO GGA1 (Table S3). Sanger sequencing confirmed the gene targeting events in the two lines (Fig. S6, *A*–*C*). Further Western blotting (Fig. S7*A*) shows these GGA1-null lines lead to the loss of GGA1 protein. Protein expression of GGA3 and tubulin was not changed (Fig. S7*A*), demonstrating the specificity of our GGA1 KO lines. Also, NHE6 protein level was not altered, suggesting that loss of GGA1 does not affect NHE6 protein levels (Fig. S7*A*).

**Figure 6:**
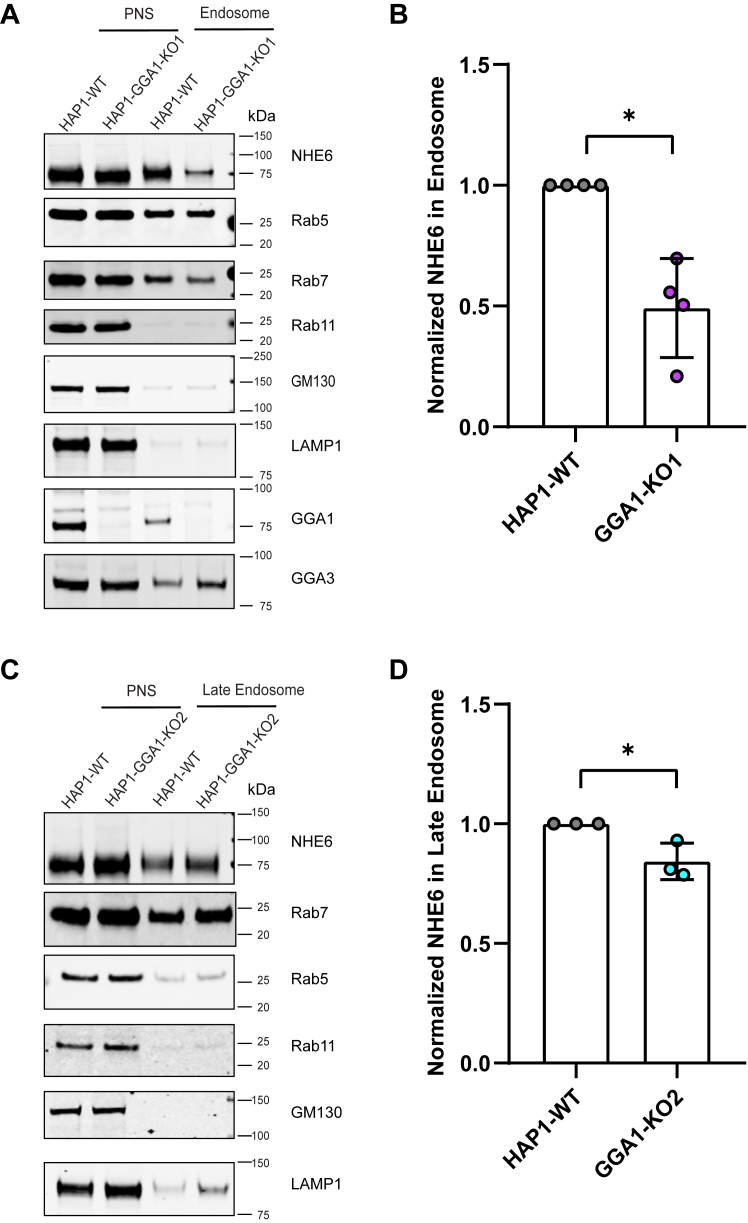
**Loss of GGA1 leads to less NHE6 in endosomes**. *A*, Western blot of NHE6 protein in early endosome fractionation in HAP1 GGA1 KO line 1 and WT cells using the following markers: Rab5 (early endosome), Rab7 (late endosome), Rab11 (recycling endosome), LAMP1 (lysosome), and GM130 (Golgi). PNS = post nuclear supernatant. *B*, quantification of NHE6 protein levels in endosome fractions in HAP1 GGA1-KO line 1 and WT cells. The normalization was performed as follows: NHE6 in endosomes was first normalized to NHE6 in PNS (termed A); Rab5 and Rab7 in endosomes was normalized to Rab5 and Rab7 in PNS (termed B); normalized NHE6 in endosomes was then calculated as A divided by *B*. (mean ± SD; n = 4; ∗*p* = 0.0158, Unpaired two-tailed Student’s *t* test with Welch’s correction). *C*, Western blot of NHE6 protein in late endosome fractionation in HAP1 GGA1 KO line 2 and WT cells using the following markers: Rab5 (early endosome), Rab7 (late endosome), Rab11 (recycling endosome), LAMP1 (lysosome), and GM130 (Golgi). PNS = post nuclear supernatant. *D*, quantification of NHE6 in late endosome fractionation in HAP1 GGA1KO line 2 and WT cells. For normalization: NHE6 in late endosomes was first normalized to NHE6 in PNS (termed A); Rab7 in late endosomes was normalized to Rab7 in PNS (termed B); normalized NHE6 in late endosomes was then calculated as A divided by B. (mean ± SD; n = 3; ∗*p* = 0.0237, Unpaired two-tailed Student’s *t* test). GGA1, Golgi-associated, gamma adaptin ear-containing, ARF binding protein 1; NHE6, Na+/H+ exchanger 6.

Accumulating evidence indicates that GGAs localize to Golgi and endosomes and regulate cargo trafficking between the Golgi and endosomes (33, 34, 53, 58, 59, 60). NHE6 is distributed throughout the endocytic pathway, (4, 12, 61). Thus, we hypothesize that GGA1 regulates NHE6 trafficking from both Golgi to endosomes and retrograde endosome-Golgi. To test this hypothesis, we conducted a series of subcellular fractionations to investigate NHE6 distribution across different organelles in the presence and absence of GGA1 using HAP1-GGA1-KO cell lines. HAP1-WT and GGA1-KO cells were used to fractionate endosomes using a method based on protocols published by de Araujo *et al.* (2015a and b) (62, 63). The purity of the fractionation product was verified by detecting different organelle markers: Rab5 for early endosomes (EEs), Rab7 for LEs, Rab11 for recycling endosomes; LAMP1 for lysosomes; and GM130 for Golgi (Fig. 6*A*). As shown from Figure 6*B*, GGA1 KO lines had a significant reduction of NHE6 in the endosome fraction, indicating GGA1 is playing a role to stabilize NHE6 in endosomes. We then investigated how loss of GGA1 affected NHE6 using a fractionation method to isolate a cellular fractionation enriched for the LE compartment (62, 63). We also observed an average of 15.72% reduction of NHE6 in the LE compartment in GGA1 KO lines compared to control cells (Fig. 6, *C* and *D*). These data support the interpretation that GGA1 is required to stabilize NHE6 localization to endosome compartments.

### Loss of GGA1 disrupts NHE6 cellular distribution

**Figure 7:**
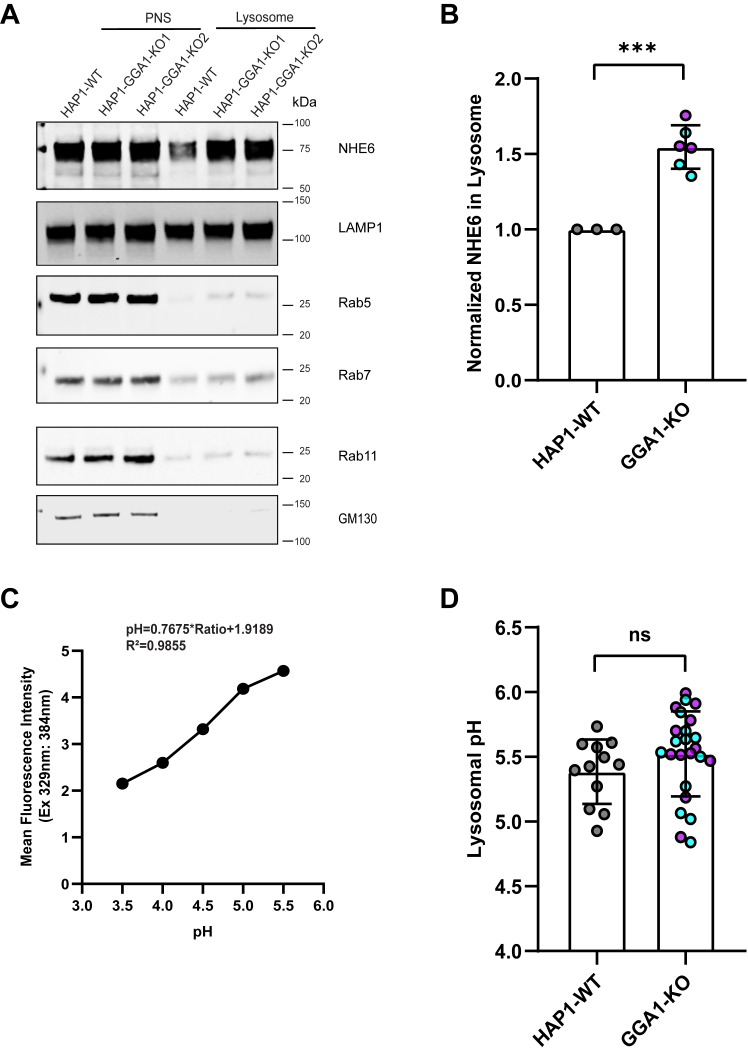
**Loss of GGA1 leads to greater NHE6 in lysosomes but does not affect lysosome pH**. *A*, Western blot of NHE6 protein in lysosome fractionation in HAP1 GGA1 KO and WT cells using the following markers: LAMP1 (lysosome), Rab5 (early endosome), Rab7 (late endosome), Rab11 (recycling endosome), GM130 (Golgi). PNS=post nuclear supernatant. *B*, quantification of NHE6 in lysosome fractionation in HAP1 GGA1 KO and WT cells. The normalization was performed as follows: NHE6 in lysosome was first normalized to NHE6 in PNS (termed A); LAMP1 in lysosome was normalized to LAMP1 in PNS (termed B); normalized NHE6 in lysosome was then calculated as A divided by *B*. (mean ± SD; n = 3; ∗∗∗*p* = 0.0002, Unpaired two-tailed Student’s *t* test with Welch’s correction). GGA1-KO1 and KO2 are presented as *magenta* and *blue* color, respectively. *C*, pH calibration curve graph for lysosome pH measurement by LysoSensor *Yellow/Blue* DND-160 after 1 min incubation. *D*, quantification of lysosomal pH in HAP1 GGA1 KO and WT cells. (mean ± SD; n = 12 replicates; ns=not significant; Unpaired two-tailed Student’s *t* test with Welch’s correction) GGA1-KO1 and KO2 are presented as *magenta* and *blue* color, respectively. GGA1, Golgi-associated, gamma adaptin ear-containing, ARF binding protein 1; NHE6, Na+/H+ exchanger 6.

We performed lysosome compartment fractionation in HAP1 cells to determine how GGA1 affects the distribution of NHE6 in lysosomes. As shown from Figure 7*A*, NHE6 is found at a low level in the fractionated lysosome compartment, using LAMP1 as a lysosome marker. NHE6 was significantly elevated in the fractionated lysosome compartment in GGA1 KO (Fig. 7*B*). This implies that loss of GGA1 promotes mislocalization of NHE6 to lysosomes.

Since loss of NHE6 leads to hyperacidification of the lysosome compartment (8), we wondered whether enriched NHE6 localization in lysosomes in GGA1 KO lines would lead to alkalinization of lysosome pH. We measured lysosome pH using the unconjugated pH-dependent fluorescent probe LysoSensor DND-160. Considering the risk of lysosome alkalization as an effect of LysoSensor in longtime treatment, we used a short 1-min treatment time at the concentration of 1μM according to the instructions described in experimental procedures. We calculated lysosome pH using the equation derived from a standard curve and converting the mean intensity to mean lysosomal pH (Fig. 7*C*). There were no statistically significant differences in intraluminal lysosomal pH between GGA1 KO and control lines (Fig. 7*D*). From these findings we conclude that loss of GGA1 leads to greater distribution of NHE6 to lysosomes, while lysosome pH is unaffected.

**Figure 8:**
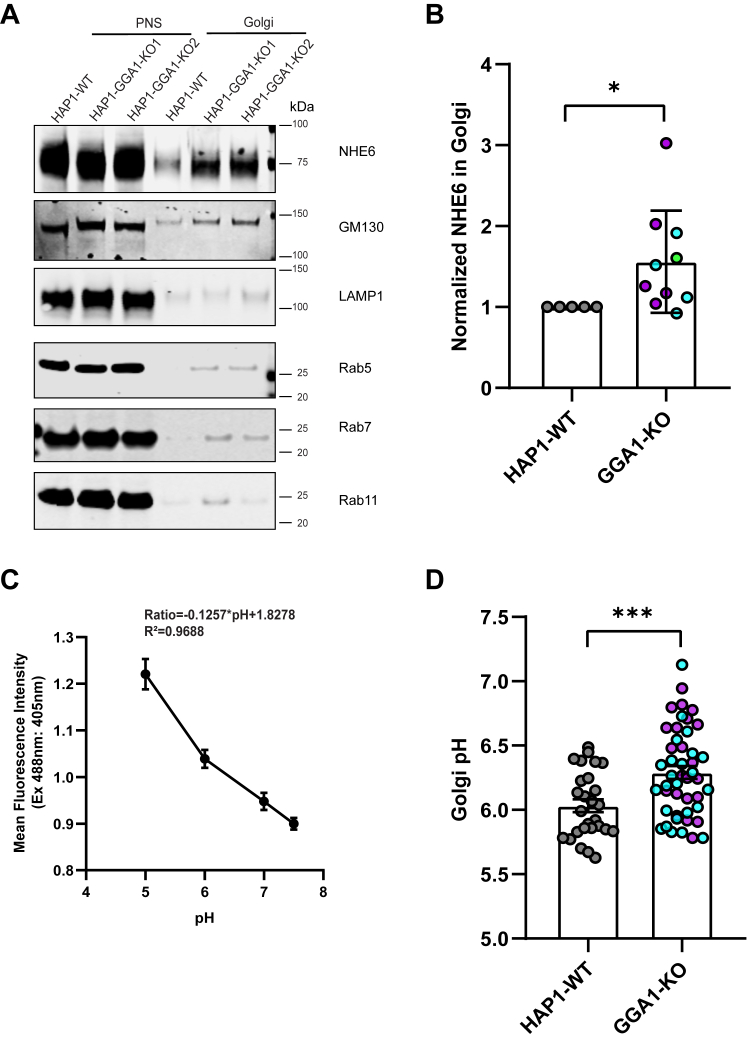
**Loss of GGA1 leads to more NHE6 in Golgi and alkalinization of Golgi pH**. *A*, Western blot of NHE6 protein in Golgi fractionation from HAP1 GGA1 KO and WT cells using the following markers: GM130 (Golgi), LAMP1 (lysosome), Rab5 (early endosome), Rab7 (late endosome), and Rab11 (recycling endosome). PNS = post-nuclear supernatant. *B*, quantification of NHE6 in Golgi fractionation in HAP1 GGA1 KO and WT cells. The normalization was performed as follows: NHE6 in Golgi was first normalized to NHE6 in the PNS (termed A); GM130 in Golgi was normalized to GM130 in PNS (termed B); normalized NHE6 in Golgi was then calculated as A divided by *B*. (mean ± SD; n = 5; GGA1-KO1 and KO2 are presented as *magenta* and *blue* color, respectively; ∗*p* = 0.0207, Unpaired two-tailed Student’s *t* test with Welch’s correction). GGA1-KO1 and KO2 are presented as *magenta* and *blue* color, respectively. *C*, graph of the Golgi pH calibration curve in HAP1 GGA1 KO and WT cells using TGN38-pHluorin construct. *D*, quantification of Golgi pH in HAP1 GGA1 KO and WT cells. (mean ± SD; WT n = 27, GGA1 KO1 n = 22, GGA1 KO2 n = 24 cells; ∗∗∗*p* = 0.0005, Unpaired two-tailed Student’s *t* test with Welch’s correction). GGA1-KO1 and KO2 are presented as *magenta* and *blue* color, respectively. GGA1, Golgi-associated, gamma adaptin ear-containing, ARF binding protein 1; NHE6, Na+/H+ exchanger 6; TGN, trans-Golgi network.

We then performed Golgi fractionation in GGA1 KO HAP1 cells to determine whether loss of GGA1 alters NHE6 Golgi localization. As compared to control cells, we detected a statistically significant elevation in the amount of NHE6 in the Golgi fraction in GGA1 KO cells (Fig. 8*B*). We then measured luminal TGN pH using a ratiometric TGN38-pHluorin construct in GGA1 KO HAP1 cells (64, 65, 66). We first confirmed that TGN38-pHluorin is properly trafficked to Golgi compartments as it colocalizes with the cis-Golgi (GM130, Fig. S8*A*), and TGN (TGN38, Fig. S8*B*) markers. We then calculated Golgi pH using the equation derived from a standard curve and converting the mean intensity to mean Golgi pH (Fig. 8*C*). Loss of GGA1 caused a significant increase in TGN pH compared to the control HAP1 line (Fig. 8*D*). These findings suggest that loss of GGA1 leads to alkalinization of the TGN, possibly mediated by increased NHE6 protein level in the Golgi.

**Figure 9:**
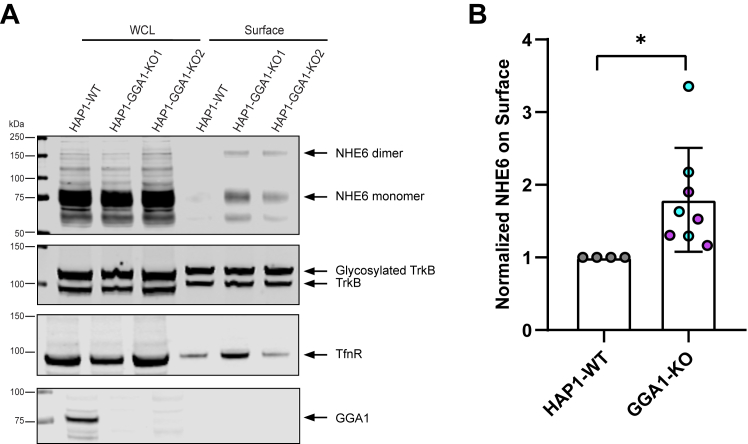
**Loss of GGA1 leads to more NHE6 on plasma membrane**. *A*, whole cell lysate (WCL) and biotinylated surface were separated by Western blot and detected by immunoblotting with anti-NHE6, transferrin receptor (TfnR), tropomyosin receptor kinase B (TrkB), and GGA1 antibodies. TfnR and TrkB are shown to demonstrate the quality of the cell surface labeling and fractionation; whereas GGA1, an intracellular protein, is not detected in the cell surface fraction. *B*, quantification of NHE6 on plasma membrane in HAP1 GGA1 KO and WT cells. The normalization was performed as follows: NHE6 on the surface was first normalized to NHE6 in WCL (termed A); the plasma membrane protein TfnR on the surface was normalized to TfnR in WCL (termed B); normalized NHE6 on the surface was then calculated as A divided by *B*. (mean ± SD; n = 4 for each line, GGA1-KO1 and KO2 are presented as *magenta* and *blue color*, respectively; ∗*p* = 0.0163, Unpaired two-tailed Student’s *t* test with Welch’s correction). GGA1-KO1 and KO2 are presented as *magenta* and *blue* color, respectively. GGA1, Golgi-associated, gamma adaptin ear-containing, ARF binding protein 1; NHE6, Na+/H+ exchanger 6.

Finally, we examined whether loss of GGA1 alters NHE6 distribution to the cell surface, as knocking down GGA1, GGA2, or GGA3 by siRNA in HeLa cells increases extracellular secretion of the lysosome enzyme cathepsin D (38). As shown from Figure 9, GGA1 KO cells exhibited significantly more NHE6 on the cell surface compared to control cells. Therefore, loss of GGA1 leads to increased trafficking of NHE6 to the plasma membrane. This result suggests that the interaction of NHE6 with GGA1 may promote NHE6 endosome localization, and loss of GGA1 enables NHE6 exocytosis from Golgi to the cell surface. Overall, in the absence of GGA1, we observe mislocalization of NHE6 in the cell: we observe physiologically relevant NHE6 elevations in Golgi, reduced levels of trafficking to endosome, elevations in lysosome, as well as elevations on the cell surface.

## Discussion

NHE6 functions in regulating intraendosomal pH and endosome maturation (4, 8), yet the mechanism of this process is not completely understood. In this study, we identify new NHE6-interaction partners—GGA1 and GGA3. We demonstrate an interaction between the C terminus of NHE6 and the GGA1 protein by two-hybrid screening. A strong result of 13 among 123 captured clones in the yeast-two hybrid encoded the GGA1 protein, indicating the C terminus of NHE6 is sufficient for binding with GGA1 in yeast cells. The chimeric NHE1/NHE6 experiment further indicates that the NHE6 C terminus is the predominant domain for the interaction with GGA1. We further demonstrate that this interaction occurs in endogenously expressed GGA1 and NHE6 in mammalian cells and mouse brain tissue.

We further demonstrate that GGA1 predominantly interacts with organellar NHEs (*e.g.* NHE6, NHE7, and NHE9), and less to cell-surface-localized NHEs (*e.g.* NHE1 and NHE5). Additionally, we show that GGA3 interacts with organellar NHE6 and NHE9. Taken together, these findings indicate that organellar NHE family members, that have high C terminus similarity, are new interaction partners with GGA family members. Overall, these data suggest that GGA1 may function in transporting NHEs between the Golgi and endocytic pathway.

We characterized the GGA1 domains responsible for binding with NHE6. The GGA1 domains VHS, GAT, and GAE regions all interact with NHE6, with the GAE domain showing the strongest interaction with NHE6. Further experiments will be required to address the underlying mechanism regarding the strong interaction of GAE domain with NHE6. Although the GAE domain strongly interacts with NHE6, the “Hinge linker and GAE” domain reduces this interaction., This finding suggests that the Hinge linker domain functions as a binding interfering domain. In the Hinge linker domain, the AC-LL motif functions as an autoinhibitor by competing to bind the ligand-banding site in VHS domain (37, 55). In this study, it is possible the AC-LL motif in GGA1 Hinge linker domain (^357^DDELM^361^) may play an autoinhibition role on NHE6 *versus* GGA1 VHS domain interaction. NHE6 plasmids used in this study contain the NHE6.2 isoform, which share the same C terminus sequence with the canonical NHE6.1. The major difference between these isoforms is that NHE6.2 has a shorter N terminus by 32 amino acid residues, which does not appear to affect the interaction between NHE6 and GGA1/3.

Using two HAP1 GGA1 KO cell lines, we find loss of GGA1 alters the distribution of NHE6. In GGA1 KO cells, NHE6 protein was less likely to be found in EEs and more likely to be found in lysosomes, Golgi, and at the plasma membrane. This is consistent with loss of GGAs leading to greater extracellular secretion of lysosomal enzymes *via* the plasma membrane (34). Given that a function of NHE6 is to alkalinize endolysosomal compartments (4, 8), we examined whether NHE6 mislocalization in GGA1 KO cells disrupts organellar pH. Despite enriched NHE6 in lysosomes in GGA1 KO cells, there were no significant differences in lysosomal pH. However, Golgi pH was significantly increased (*i.e.* more alkaline) in GGA1 KO cells compared to WT cells. Taken together, these findings expand our understanding of how GGA1 regulates NHE6 trafficking and the functional consequences of disrupting this interaction. Loss of GGA1 alters the distribution of NHE6. In HAP1 GGA1 KO cell lines, NHE6 is less likely to be found in EEs, whereas it is enriched in lysosomes, Golgi, and at the cell surface. Previous reports have indicated that minimal NHE6 is localized to lysosomes (12, 61). Despite enriched NHE6 trafficking to lysosomes, we did not detect a difference in pH in the lysosomal lumen. Therefore, we posit that the lysosome-associated NHE6 is not a key regulator for lysosomal pH and/or is nonfunctional. Interestingly, Golgi fractionation showed that more NHE6 accumulated in the Golgi in the absence of GGA1, at the same time, an alkalinized Golgi lumen was detected consistent with NHE6 retention in the Golgi.

Loss of NHE6 leads to CS, a neurogenetic disorder associated with neurodegenerative features including progressive cerebellar atrophy, motor decline, and neurodegeneration, which appears to be due in part to endolysosome dysfunction (8, 10, 67, 68). An important question related to CS is the extent to which underlying pathogenic mechanisms are shared with more common neurodegenerative conditions such as Alzheimer’s disease. The interaction between NHE6 and GGA1 supports these ideas of overlap; for example, GGA1 regulates the levels of β-site amyloid precursor protein-cleaving enzyme 1 (BACE1) (39, 69), and also modulates the processing of amyloid precursor protein to amyloid-β (70, 71, 72).

Our study establishes a novel NHE6 binding partner—GGA1—using different models and multiple techniques, including yeast two-hybrid, IP, subcellular fractionation, confocal microscopy, and expansion microscopy. GGA1 domains, such as VHS, GAT, and GAE are all able to bind NHE6. We extend our investigation to identify which NHEs interact with GGA1. We find that GGA1 interacts with other organellar NHEs like NHE6 (*e.g.* NHE7 and NHE9), but weakly with plasma membrane NHEs (*e.g.* NHE1 and NHE5). We also find that another GGA family member—GGA3—interacts with the organellar NHEs, NHE6, and NHE9. This work highlights the relationship between GGAs and NHEs. Using confocal microscopy and expansion microscopy we visualized the distribution of NHE6-GGA1 colocalization in neurons *in vitro*. These experiments demonstrate that NHE6-GGA1 colocalization occurs predominantly in the perinuclear region in the Golgi complex and elsewhere, presumably endosomes. We discover that loss of GGA1 alkalinizes the TGN. We believe NHE6 is involved in this finding given (1) GGA1’s role in NHE6 trafficking and (2) NHE6’s role in regulating pH. Interestingly, we also observed greater NHE6 levels in the TGN in our Golgi fractionation experiment. Therefore, we suspect both enhanced NHE6 exchanger activity and greater NHE6 levels in the TGN mediates the higher TGN pH in GGA1 KO cells. Abnormal Golgi pH impairs glycosylation and protein trafficking and is associated with a range of human diseases (73, 74). Notably, loss of the Angelman syndrome protein Ube3a leads to an overlapping similar Golgi pH phenotype as loss of GGA1, as the Golgi complex becomes hyperalkalinized (75). A limitation of our study is that LysoSensor DND-160 is not a restricted measure of lysosome pH, but rather the pH of acidic organelles. While LysoSensor is most likely to measure the luminal pH of acidic lysosomes, it may also include highly acidic LEs. Regardless, our finding that loss of GGA1 enhances NHE6 localization to lysosomes, but does not affect lysosome pH suggests that NHE6 exchanger function is less active in lysosomes. Additionally, while the two methods of endosome fractionation are very effective in recovering early (Rab5-positive) and LEs (Rab7-positive) together (63), or alternatively for LEs alone (63), we do not recover recycling endosomes (Rab11-positive); thereby, our studies do not address the role of the interaction of GGA1 and NHE6 with regard to recycling endosomes. Some studies have indicated that GGA1 can regulate the recycling pathway, such as for β-site amyloid precursor protein-cleaving enzyme (BACE) trafficking (58, 72); however, future studies will be required to understand the effect of GGA1 on NHE6 trafficking in recycling endosomes.

In summary, our study identifies GGA1 as a new binding partner with NHE6 and establishes a link between NHEs and GGAs. We find that NHE6 and GGA1 localize throughout the cell including endosomes and the Golgi complex. Loss of GGA1 leads to NHE6 mislocalization as its expression is increased in lysosomes, Golgi, and the plasma membrane, but decreased in endosomes. Functionally, loss of GGA1 alkalinizes TGN pH that is likely mediated by Golgi retention of NHE6.

## Experimental procedures

### Yeast two-hybrid screening and assay

The coding sequence for amino acids Leu^537^-Asp^630^ of the mouse NHE6 protein (GenBank accession number gi: 120577706) was PCR-amplified and cloned into pB27 as a C-terminal fusion to LexA (N-LexA-NHE6-C). The peptide sequence corresponded to: LHIRVGVDSDQEHLGVPDNERRTTKAESAWLFRMWYNFDHNYLKPLLTHSGPPLTTTLPACCGPIARCLTSPQAYENQEQLKDDDSDLILNDGDISLTYGDSTVNTESATASAPRRFMGNSSEDALDRELTFGDHELVIRGTRLVLPMDDSEPALNSLGDTRHSPA. The construct was checked by sequencing the entire insert and used as a bait to screen a random-primed rat hippocampus cDNA library constructed into pP6. pB27 and pP6 derive from the original pBTM116 (76) and pGADGH (77) plasmids, respectively.

In total, 53.8 million clones (5.4-fold the complexity of the library) were screened using a mating approach with Y187 (matα) and L40ΔGal4 (mata) yeast strains as previously described (78). Subsequently, 123 His+ colonies were selected on a medium lacking tryptophan, leucine, and histidine, and supplemented with 0.5 mM 3-aminotriazole to handle bait autoactivation. The prey fragments of the positive clones were amplified by PCR and sequenced at their 5′ and 3′ junctions. The resulting sequences were used to identify the corresponding interacting proteins in the GenBank database (NCBI) using a fully automated procedure. A confidence score (PBS) was attributed to each interaction as previously described (79).

### Further description of the confidence score

The PBS relies on two different levels of analysis. Firstly, a local score takes into account the redundancy and independency of prey fragments, as well as the distribution of reading frames and stop codons in overlapping fragments. Secondly, a global score takes into account the interactions found in all the screens performed using the same library. This global score represents the probability of an interaction being nonspecific. For practical use, the scores were divided into four categories, from A (highest confidence) to D (lowest confidence). A fifth category (E) specifically flags interactions involving highly connected prey domains previously found several times in screens performed on libraries derived from the same organism. Finally, several of these highly connected domains have been confirmed as false-positive of the technique and are now tagged as F. The PBS scores have been shown to positively correlate with the biological significance of interactions (80, 81).

### Cell culture, antibodies and reagents

HEK293T cells were cultured in Dulbecco’s modified Eagle’s medium supplied with 10% fetal bovine serum (FBS), 1% antibiotic-antimycotic and 1% GlutaMAX, SH-SY5Y neuroblastoma cells were cultured in Dulbecco’s modified Eagle’s medium/F12 medium supplied with 10% FBS, 1% antibiotic-antimycotic, HAP1 WT, and GGA1-KO cells were cultured in Iscove's Modified Dulbecco's Medium supplied with 10% FBS and 1% antibiotic-antimycotic at 37 °C and 5% CO2. Cell culture media and reagents were purchased from Invitrogen. Rabbit polyclonal anti-NHE6 antibody was customized made against isoform-specific sequences GDHELVIRGTRLVLPMDDSE (aa636–655) of the C terminus of NHE6.0 (4). Antisera were collected and affinity-purified (4). HA antibody (Cell Signaling Technology, 3724s and Santa Cruz, sc-7392), GFP antibody (Cell Signaling Technology, 2956s), GGA1 antibodies (Abcam (ab57247), Santa Cruz (sc-271927), Proteintech (25674-1-AP), Novus Biologicals, H00026088-M01 (3F11), and Pierce (PA5-12130)). GGA3 antibody (BD Biosciences, 612310), GM130 (BD Biosciences, 610822), LAMP1 (Abcam, ab25630), TrkB (BD Biosciences, 610101). Rab5 (Cell Signaling Technology, 3547s and 46449s), Rab7 (Abcam, ab137029 and Cell Signaling Technology, 9367s) and Rab11(BD Biosciences, 610656). Transferrin receptor (Invitrogen, 13–6890), NHE5 (PA5-37222), NHE7 (PA5-75424), and NHE9 (Morrow lab customized made through Covance, epitope located within the C-terminal tail of mouse Slc9a9: SPSPSSPTTKLALDQKSSGKC). Flag M2 (Sigma-Aldrich, F1804). a-Tubulin (Sigma-Aldrich, T6074), c-Myc antibody (Santa Cruz, sc-40). Protein G agarose beads (sc-2002) was purchased from Santa Cruz, and Dynabeads Protein G (10003D) and Anti-HA Magnetic Beads (#88836) from Thermo Fisher Scientific. Lysosome enrichment kit (Thermo Fisher Scientific, 89839) and Golgi isolation kit (Sigma-Aldrich, GL0010). Pierce cell surface protein biotinylation and isolation kit (Thermo Fisher Scientific, A44390). LysoSensor Yellow/Blue DND160 (Invitrogen, L7545). SYTOX Green Nucleic acid stain (Thermo Fisher Scientific, S7020) was purchased.

### GGA1 KO cell lines

Two GGA1 KO cell lines (KO1 and KO2) were customized and generated by Horizon Discovery. Detailed information is shown in Table S3.

### Plasmids constructs, PCR-mediated mutagenesis and transfection

pCMV-mGGA1 which was originally from Open Biosystems, GGA1 full length and a series of domains were amplified from pCMV-mGGA1 and then cloned into mammalian expression vector pcDNA3.1/CT-GFP-TOPO (Invitrogen). PCR-mediated GGA1-Hinge domain (299–505aa) mutagenesis was carried out according to the methods provided by Quickchange II site-directed mutagenesis kit. c-Myc-GGA1 was amplified from pCMV-mGGA1 and double digested by restriction enzymes of EcoRI +XhoI and then ligated to Clontech pCMV-c-Myc vector.

pmNHE9 was originally from Open Biosystems, and then cloned into mammalian expression vector pcDNA3.1/CT-GFP-TOPO (Invitrogen). HA-tagged hNHE6.2-FL and cytoplasmic domain were cloned by GeneCopoeia. hNHE1-HA (3× on c terminal) (pYN4+, #78715) was originally from Addgene with D720G mutation, mutagenesis was performed to correct this mutation back. hNHE5 (#132163), hNHE7 (#132187), which was originally obtained from Addgene, together with mNHE9-GFP, and mutagenesis (G720D) corrected hNHE1-HA (c-hNHE1) cloned into HA vector using HA-tagged hNHE6.2-FL as template and using in-fusion snap method (Takara, In-Fusion Snap Assembly Master Mix, 638947) to replace hNHE6 with c-hNHE1, hNHE5, hNHE7, or mNHE9.

In-fusion snap method was also used for chimeric NHE6/NHE1 plasmids construction. Snap NHE1N/NHE6C-HA was constructed by replacing hNHE6 N terminus (1–504aa) with hNHE1.1 N terminus (1–499aa). Snap NHE6N/NHE1C-HA was constructed by replacing hNHE6 C terminus (505–669aa) with hNHE1.1 C terminus (500–815aa).

Transfection of cells with various mammalian expression constructs by Lipofectamine 2000 (Invitrogen) was according to the methods provided by manufacturer’s specification.

All constructs were finally verified by DNA sequencing by Genewiz or Plasmidsaurus (Tables S2, S4, and S5). All primers were listed in Tables S6 and S7.

### Western blotting and coimmunoprecipitation

Cells/tissues were lysed in buffer containing the following: 50 mM Tris–HCl, pH 7.8, 137 mM NaCl, 1 mM NaF, 1 mM NaVO3, 1% Triton X-100, 0.2% sarkosyl, 1 mM DTT, and 10% glycerol; or buffer containing 50 mM Tris–HCl pH7.9, 100 mM NaCl, 20% glycerol, and 0.1% NP-40; or buffer containing 25 mM Tris–HCl pH 7.4, 150 mM NaCl, 1 mM EDTA, 1% NP-40, and 5% glycerol; or radioimmunoprecipitation assay buffer containing 1M Tris-HCl, pH 7.4, 5M NaCl, 0.5M EDTA, 10% Triton X-100, 1% sodium deoxycholate and 20% SDS supplemented with protease inhibitor cocktail and phosphatase inhibitor. All cells were lysed for 30 min on ice and were centrifuged at 13,200 rpm for 15 min at 4 °C to remove cell debris. Protein concentration was measured by bicinchoninic acid (BCA) assay using the Pierce BCA Kit (Thermo Fisher Scientific, 23225). For IP, antibody was conjugated to Dynabeads Protein G (Thermo Fisher Scientific, 10003D) at room temperature (RT) for 2 h. Cell lysates were then incubated with antibody-conjugated beads overnight (O/N) at 4 °C, or cell lysates were incubated with antibody for 2 h then conjugate Protein G plus-agarose (Santa Cruz Biotechnology, sc-2002) (O/N) at 4 °C, or cell lysates were incubated with Anti-HA Magnetic beads (Thermo Fisher Scientific, #88836) (O/N) at 4 °C. The following day, the beads were gently pelleted, cell lysates were removed, and the beads were washed three times with PBS-buffer containing 0.02% Tween 20. Pelleted beads were then boiled in sample buffer at 95 °C for 5 min before loading onto 4 to 12% SDS-PAGE gels (Novex #NP0321Box). Following separation of proteins by electrophoresis, gels were transferred to nitrocellulose membranes (Novex #LC2000). Western blots were performed using standard procedures (5, 82) and were analyzed with the LI-COR Odyssey Imaging System.

### Primary cultured neurons immunocytochemistry and confocal imaging

All animal experiments were approved by the Institutional Animal Care and Use Committee and carried out in accordance with the Brown University Animal Experimentation Regulations. Primary neurons were dissociated as described before (4). For hippocampal cultures, WT neurons were firstly washed three times with 1× PBS-buffer, then fixed with 4% paraformaldehyde for 10 min at RT and permeabilized for 10 min in PBS-buffer containing 0.25% Triton X-100. Nonspecific binding was blocked by incubation with 10% normal goat serum (Jackson ImmunoResearch #005–000–121) in PBS-buffer containing 0.1% Tween 20 (PBST) for 1 h. Cells were then incubated O/N at 4 °C with primary antibody diluted in PBST containing 2% normal donkey serum, washed 3 × 5 min with PBST, and incubated for 1 h at RT with secondary antibody diluted as for primary antibody. Nuclei were counterstained with Hoechst (1:1600 working dilution of 10 mg/ml stock; Invitrogen #33342). Cells were then washed 3 times with PBST and mounted on slides with Fluoromount-G (SouthernBiotech #0100–01). Images were captured with FV3000 Confocal or Zeiss LSM800 microscope.

For NHE6 and GGA1 colocalization, Z-series images were collected using a 60× oil objective and Mander’s colocalization were analyzed using ImageJ (https://imagej.net/ij/) software (National Institutes of Health).

### Colocalization of GGA1 and NHE6 by 5× expansion microscopy method

#### Cell culture expansion

Rat hippocampal neurons were cultured from WT rat pups (p0-p2) in neurobasal media supplemented with B27 and glutamax 1%. On 14 days *in vitro* (DIV 14), rat hippocampal neurons were fixed with 4% paraformaldehyde for 30 min and followed by post fixation and first round of gelation for hydrogel expansion (5×) as per the protocol mentioned in M'Saad O. and Bewersdorf J, 2020 (57).

#### Antibody labeling

For GGA1 and NHE6 colocalization with respect to Golgi in 5× expanded cells in hydrogels, the cells were labeled with a cocktail of primary antibodies (1:500) of mouse GGA1 (H00026088-M01, Novus Biologicals), rabbit NHE6 (Covance), and guinea pig Giantin (263004, Synaptic Systems) were incubated in Tris-buffered saline (TBS)-Tween (927–65001, LI-COR Biosciences) for 24 h on a rocking platform at RT. The expanded gels were later washed three times with TBS-Tween 15 min each followed by a secondary antibody labeling. Later the gels were incubated in the cocktail of secondary antibodies (1:500): Goat anti-guinea pig Alexa488, Donkey anti-mouse CF568, and Goat anti-rabbit Alexa647 in TBS-Tween for 16 to 20 h, followed by three 15 min washes with PBST. The nucleus was stained with 1:1500 Sytox Blue Nucleic acid stain (S11348, Thermo Fisher Scientific) in 1× PBS-buffer for 30 min followed by three 15 min washes in 1× PBS-buffer. The water was changed three times every 30 min and incubated in water O/N at RT.

#### Imaging

On the day of imaging, the gels were mounted onto glass bottomed MatTek dishes and sealed with dental glue as described in M'Saad O. and Bewersdorf J, 2020 (57). For cells expanded using hydrogels, z-stacks were acquired using Olympus FV3000 microscope. Images were collected with 1024 × 1024 pixel resolution using 60× water objective. Voxel size for the acquired images were ∼114 × 114 × 63 μm.

#### Analysis

For GGA1 and NHE6 colocalization with respect to Golgi in 5× expanded cells in hydrogels, IMARIS software (version 10.0.0.1, https://imaris.oxinst.com/) was used for analysis. Nucleus and Giantin were analyzed using surfaces visualization in the surpass tree of IMARIS software. GGA1 and NHE6 localizations were analyzed using spots visualization. The overall and shortest distance statistics were generated and extracted from the software to plot using GraphPad Prism version 7 (https://www.graphpad.com/). The statistics were generated from the perspective of both GGA1 and NHE6 and plotted in the same graph. For measuring expansion factor contour was drawn around the nucleus (max projection) using the magic wand tool in FIJI (https://imagej.net/software/fiji/). The area of the nucleus was measured using measure plugin from FIJI. The expansion factor (EF) was calculated by measuring square root of area of expanded neurons, (a2) by area of unexpanded neurons, (a1) from the max projection. EF = √(a2/a1).

### EE and LE subcellular fractionation

Early and late endosomal fractions were prepared as described (62, 63). Subsequently, 6 ∼ 8 × 10^7^ cells used in this study were placed on ice, washed, scraped into microfuge tubes and homogenized (sucrose 250 mM, imidazole (pH7.4) 3 mM, EDTA 1 mM, cycloheximide 0.03 mM with protease inhibitors, and phosphatase inhibitors added), and then a post nuclear supernatant was prepared. The post nuclear supernatant was adjusted to 40.6% sucrose (refractometry was used to accurately measure the % of sucrose) in 3 mM imidazole, pH 7.4, loaded at the bottom of an ultra-thin centrifuge tube, and overload sequentially with 1.5 volumes of 35% and 1 volume of 25% sucrose solutions in 3 mM imidazole, pH 7.4, and then homogenization buffer (250 mM sucrose in 3 mM imidazole, pH 7.4). The gradient was centrifuged for 2-3 h at 41,600 rpm using a SW55Ti rotor (Table S8). Early and late endosomal fractions were collected at the 35/25% and 25%/homogenization buffer interfaces, respectively. Remove the bands by needle puncture on the tube wall or use large/blue tip to collect the interface to get EE and LE fractionation. BCA assay was applied, and equal amount of protein was loaded for Western blotting (WB) assay and different fractionation markers were used to monitor the purity of EE/LE fractionation.

### Lysosome subcellular fractionation

Lysosome Enrichment Kit (Thermo Fisher Scientific, 89839) was used for subcellular fractionation of lysosomes from tissue or cultured cells, following the manual’s instructions. TLA-110 rotor was used for ultracentrifuge at 145,000*g* for 2 h (Table S8) at 4 °C. Refractometry was used to accurately measure the % of sucrose.

The transparent lysosome band is on the top 2 ml of the gradient and under thick lipid. Carefully remove the lysosome band and save on ice. BCA assay was applied, and equal amount of protein was loaded for WB assay and different fractionation markers were tested to monitor the purity of lysosome fractionation.

### Golgi subcellular fractionation

Golgi isolation kit (Sigma-Aldrich, GL0010) was used with some modifications for cultured Golgi subcellular fractionation cells. The cells were homogenized in 0.25 M sucrose solution and centrifuge to get supernatant. Adjust the sucrose concentration in the sample (supernatant) to 1.25 M by adding the volume of 2.3 M sucrose solution according to formula provided by kit. A discontinuous gradient was built and the order of sucrose gradient fractions in the ultracentrifuge tube (from bottom to top) are: 1.84 M sucrose solution; sample (sucrose concentration adjusted to 1.25 M); 1.1 M sucrose solution; and 0.25 M sucrose solution. The gradient was centrifuged for 3 h at 120,000*g* using a SW55Ti rotor for 3 h (Table S8) at 2 to 8 °C and then the Golgi enriched fraction was withdrawn from the 1.1 M/0.25 M sucrose interphase. BCA assay was applied, and equal amount of protein was loaded for WB assay, and different fractionation markers were tested to monitor the purity of Golgi fractionation.

### LysoSensor Yellow/Blue DND160 lysosomal pH measurement

HAP1 WT and GGA1 KO cells were incubated at 37 °C in prewarmed, Iscove's modified Dulbecco's medium containing 1 μM of LysoSensor Yellow/Blue DND-160 for 1 min. For those cells prepared for pH calibration curve, the cells were rinsed twice with 1XPBS-buffer and once with pH calibration curve buffer (3.5, 4, 4.5, 5, and 5.5) and then incubated for 10 min in 100 μl of respective pH calibration curve buffer prepared for reading in SpectraMax M5 Microplate Reader. Light emitted at 440 and 540 nm in response to excitation at 329 and 384 nm was measured. The ratio of light emitted at 340 and 380 nm excitation was plotted against the pH values, and the pH calibration curve for the fluorescence probe was generated from the plot using Microsoft Excel. The pH was calculated using the equation from pH calibration curve (64).

### Golgi compartment pH measurement

HAP1 WT and GGA1 KO cells were seeded onto 35 mm glass bottom dishes and transiently transfected with TGN38-pHluorin plasmid the following day. Images were taken 24 h post-transfection. For standard curve making, the cells were rinsed once with the most alkalized pH standard buffer for imaging, and the appropriate region of Golgi pHluorin-expressing cells for imaging based on excitations at 405 nm and 488 nm was found and both emissions collected at 530 nm. Then the cells were rinsed once with 1× PBS-buffer followed by rinses twice with the next pH calibration curve buffer from alkalized to acidic. The images for each pH calibration curve were collected. During image taking, the cells were maintained on a heated stage held at 37 °C in a CO2 chamber. For sample image taking, the cells were rinsed once with PBS-buffer and kept in phenol-free medium. ImageJ (https://imagej.net/ij/) was then used to analyze data and regions of interest within cells containing pHluorin-labeled Golgi were selected. The florescence intensity measurement data were exported to excel and the fluorescence intensity ratio of two excitation wavelengths for each region of interest was calculated. The Golgi pH was calculated using the equation generated from making pH calibration curve (64).

### Cell surface protein biotinylation and isolation

HAP1 WT and GGA1 KO cell surface protein biotinylation and isolation were performed by following the Pierce cell surface protein biotinylation and isolation kit’s manual instruction (Thermo Fisher Scientific, A44390). In brief, cells were washed with PBS-buffer before labeling with Sulfo-NHS-SS-Biotin for 10 min at RT. Then the label was removed, washed, and cells were harvested for lysis. An equal amount of supernatant fraction from lysed cells was incubated with NeutrAvidin agarose slurry for 30 min at RT. After 4 times of washing, biotinylated protein was eluted by incubating with elution buffer for 30 min at RT. The biotin-labeled surface proteins were separated by SDS-PAGE and analyzed by immunoblotting. The intensity of the immunoreactive bands was quantified using LI-COR analysis software (https://www.licor.com/bio/image-studio/).

### Statistical analysis

Unpaired two-tailed Student’s *t* tests with Welch’s correction were performed unless mentioned separately in Figure legend. Data are presented as mean ± SD and graphs were plotted using GraphPad Prism software.

## Data availability

All data are included in this article and the supporting information.

## Supporting information

This article contains supporting information.

## Conflict of interest

The authors declare that they have no conflicts of interest with the contents of this article.
