# Supplementary material for: GGA1 interacts with the endosomal Na+/H+ exchanger NHE6 governing localization to the endosome compartment
Source: J Biol Chem. 2024 Jul 11;300(8):107552. doi: 10.1016/j.jbc.2024.107552 (PMC11375261; doi:10.1016/j.jbc.2024.107552)
Supplement: Supporting Figure S1-S8 [file mmc1.pdf]

Supporting Information

GGA1 interacts with the endosomal Na+/H+ Exchanger NHE6 governing localization to the endosome compartment

Li Ma[1,2], Ravi Kiran Kasula[1,2], Qing Ouyang[1,2], Michael Schmidt[1,2] and Eric M. Morrow[1,2#]

[1]Department of Molecular Biology, Cell Biology and Biochemistry, Brown University, Providence, RI 02912, USA; and [2]Center for Translational Neuroscience, Brown University, Providence, RI 02912, USA

[#]To whom correspondence should be addressed:
Eric M. Morrow MD PhD
Brown University, Laboratories for Molecular Medicine,
70 Ship Street, Providence, RI 02912, USA
Tel: 401-863-9778, Fax: 401-432-1607
E-mail: eric_morrow@brown.edu

This PDF file includes:

Supplementary figures

SUPPLEMENTAL FIGURE LEGENDS

Figure S1. GGA1 interaction with NHEs.

(A) Cell lysates from HEK293T cells expressing HA-tagged NHE5, NHE7, and NHE9 were subjected to western blot analysis with anti-HA to detect expression of NHE5, NHE7, and NHE9 constructs. NHE6-HA was used as a control. Red asterisks denote the monomer and dimer of NHE5, NHE7, NHE9 and NHE6.

(B) Cell lysates from HEK293T cells expressing HA-tagged NHE5, NHE7, and NHE9 were subjected to western blot analysis with anti-NHE5, NHE7, and NHE9 to detect expression of NHE5, NHE7, and NHE9 constructs. Red asterisks denote the monomer and dimer of NHE5, NHE7, NHE9 and NHE6.

(C) Cell lysates from HEK293T cells expressing c-Myc-GGA1 with HA-tagged NHE5, NHE7, NHE9, and NHE6 were immunoprecipitated with an anti-c-Myc antibody. The precipitates were probed with HA and c-Myc antibodies. Western blot analysis was performed with anti-HA to detect the expression of NHEs. Red asterisks denote the monomer and dimer of NHE5, NHE7, NHE9 and NHE6.

Figure S2. GGA3 interaction with NHE6 and NHE9.

(A) Cell lysates from HEK293T cells expressing Flag-hGGA3 with GFP-tagged hNHE6, mNHE9, and mGGA1 were immunoprecipitated with anti-Flag antibody. The precipitates were probed with GFP and Flag antibodies. Western blot analysis was performed with anti-GFP to detect the expression of NHEs and mGGA1. Red asterisks denote NHE6, NHE9 and GGA1 bands.

Figure S3. The specificity of GGA1 and NHE6 antibodies

(A) Representative image of mouse anti-GGA1 antibody specificity test (1:500 dilution, magenta) using HAP1 GGA1 wild type (WT) and GGA1 knockout (KO) cells. Scale bar: 10 µm.

(B) Representative image of rabbit anti-NHE6 antibody specificity test (1:2500 dilution) using NHE6 wild type (WT) and NHE6 null mouse primary hippocampal neurons at 14 days *in vitro* (14 DIV). Scale bar: 10 µm.

Figure S4. Specificity of NHE6 primary antibody in 5x expanded rat neuronal cell cultures.

Hippocampal neuronal rat cell cultures of DIV 14 were imaged after 5x hydrogel expansion of cells nuclear stained with sytox green, shown in green and the cells were immuno-stained with **(A)** secondary only in wild type cells with anti-rabbit Alexa 647 (blue), **(B)** shows stained for rabbit NHE6 primary antibody with anti-rabbit Alexa 647 (blue) in wild type rat hippocampal neurons, and **(C)** stained for rabbit NHE6 primary antibody with anti-rabbit Alexa 647 (blue) in NHE6 KO rat hippocampal neurons. The left panels show a single frame, and right panels show the average intensity projections of z-stacks imaged on confocal microscope. Intensities were adjusted to similar thresholds for better visualization. Scale bar: 10 µm.

Figure S5. Shortest distance of NHE6 and GGA1 in rat primary hippocampal neurons.

(A) Representative max projection z-stack images of unexpanded **(a)** or expanded **(b)** primary rat hippocampal neurons at 14 days *in vitro* (14 DIV) stained for nucleus using Sytox^{TM} Green Nucleic acid stain.

(B) Measurement of the shortest distance of GGA1 to NHE6 or vice versa based on the classifications shown in Figure **5C**. (n=14 neurons from 4 unique neuronal cultures).

Figure S6. Sanger sequencing of HAP1 GGA1 Knockout (KO) cell lines.

(A, B) Sanger sequencing from extracted DNA from HAP1 knockout (KO) and wildtype (WT) cell line PCR products. HAP1-GGA1-KO1, double knockout, has 2bp deletion in exon3 (*c*. 197_198delCT, *p*. L67DfsX14) (NM_001001561) (**A**) and 134bp deletion (**B**) in exon 4 (NM_013365).

(C) HAP1-GGA1-KO2 has 1bp insertion in exon7 (*c*.564_565insT, *p*.D189X) (NM_013365).

Figure S7. GGA1 Knockout (KO) lines do not express GGA1 protein.

(A) Western blot analysis of cell lysates harvested from HAP1 knockout (KO) and wildtype (WT) cell lines using GGA1 antibody. Tubulin was used as a loading control and GGA3 was used for the specificity of GGA1 KO.

Figure S8. TGN38-pHluorin construct colocalizes with Golgi markers in HEK cells. Representative confocal microscopy images of HEK cells transfected with TGN38-pHluorin (green) and stained with **(A)** anti-GM130 (cis-Golgi) or **(B)** anti-TGN38 (trans-Golgi) antibodies (red). Hoechst=blue. Scale bar=10 μm. Arrows denote the spots that TGN38-pHluorin colocalizes with Golgi markers.

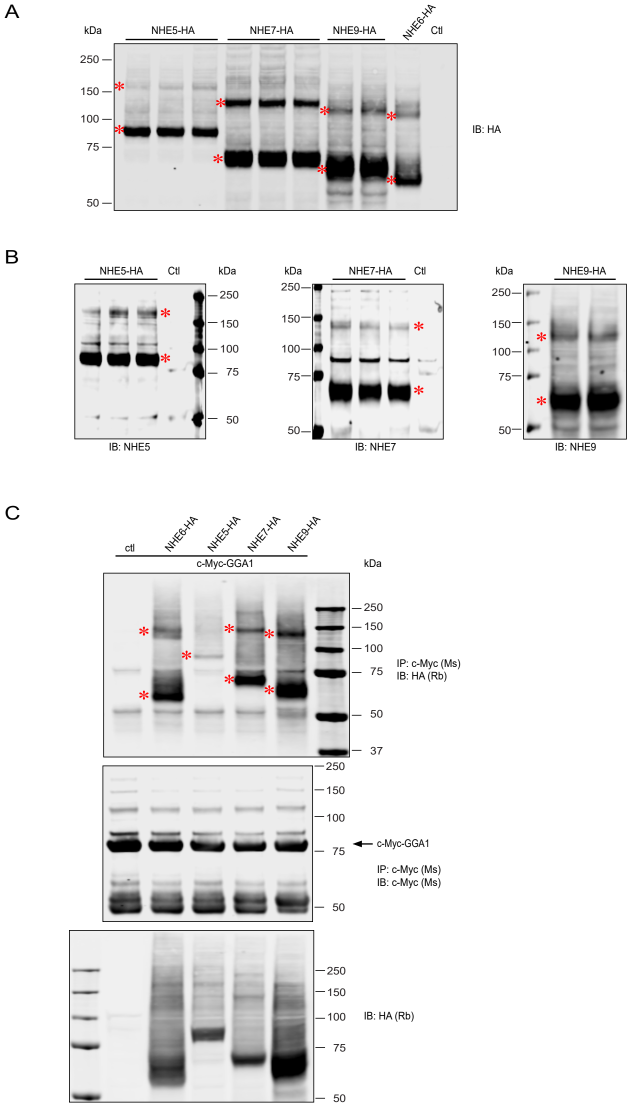

Figure S1. GGA1 interaction with NHEs

A

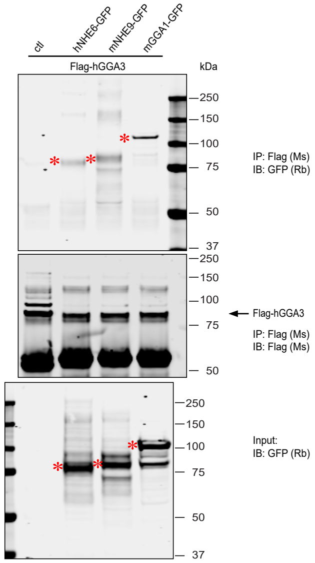

Figure S2. GGA3 interaction with NHE6 and NHE9

A

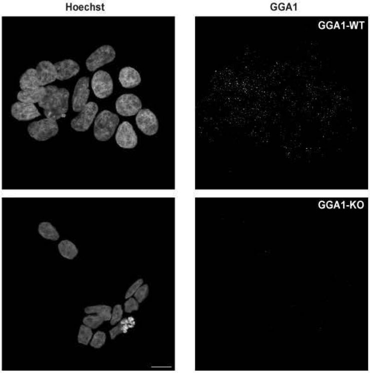

B

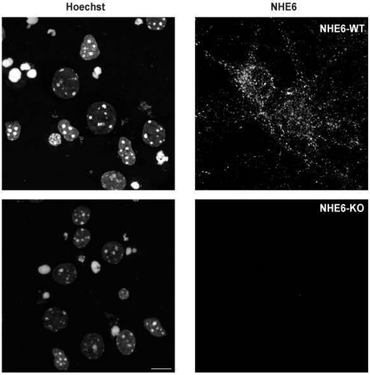

Figure S3. Specificity of GGA1 and NHE6 antibodies

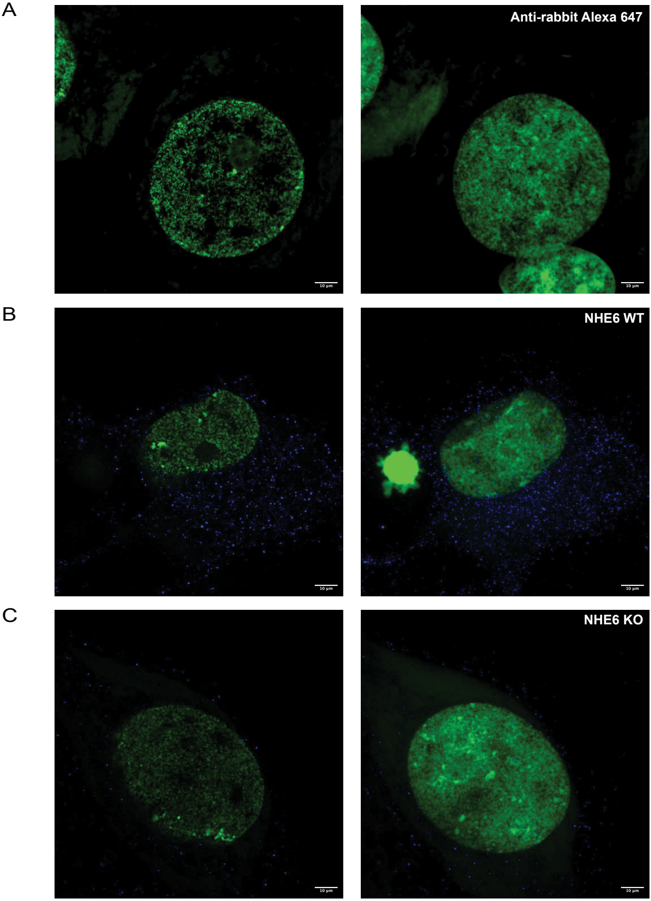

Figure S4. Specificity of NHE6 antibody in expansion gel

A

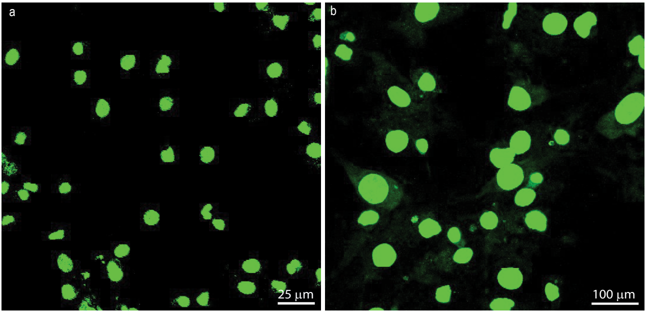

B

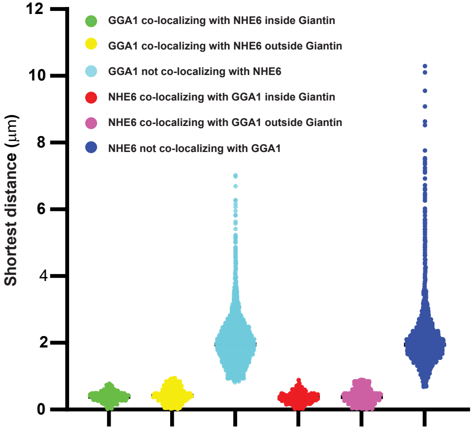

Figure S5. Shortest distance of NHE6 and GGA1 in rat primary neurons

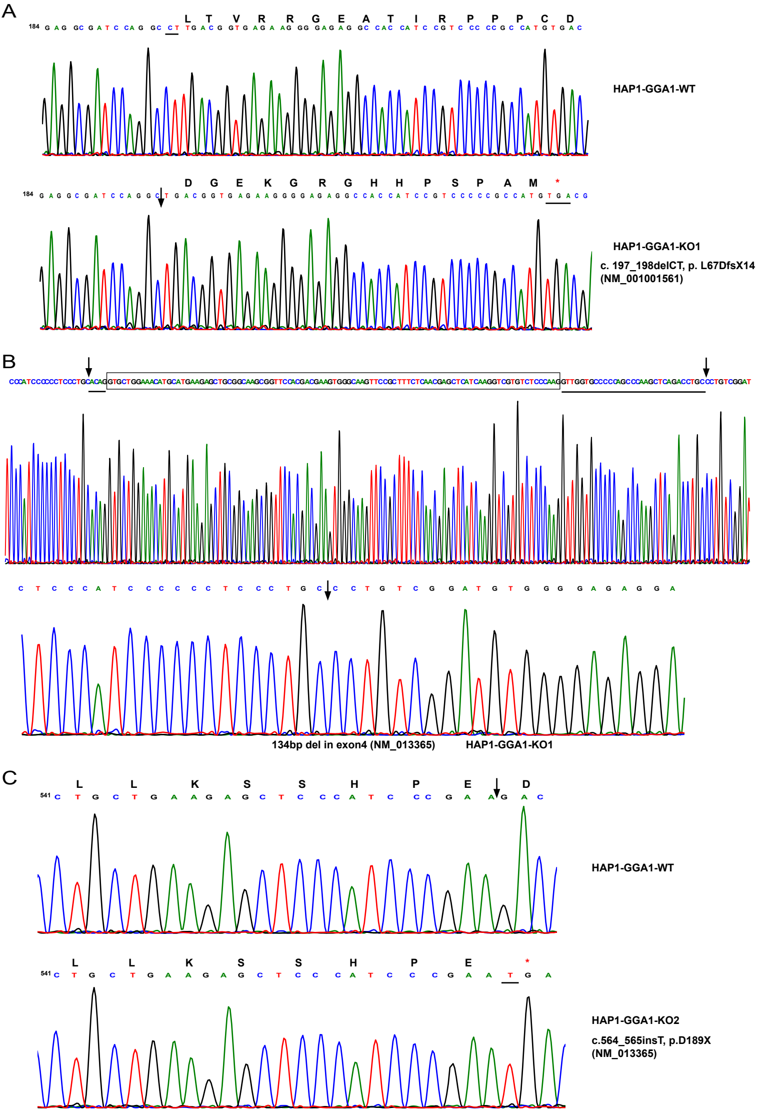

Figure S6. Sanger sequencing of HAP1 GGA1 KO cell lines

A

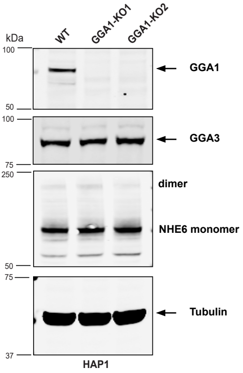

Figure S7. GGA1 KO lines do not express GGA1 protein

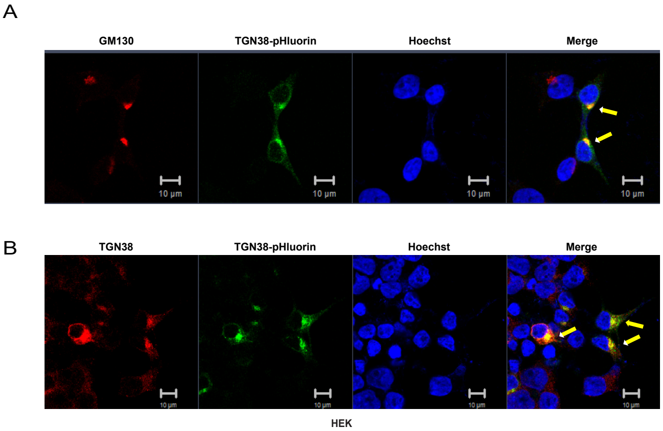

Figure S8. TGN38-pHluorin co-staining with Golgi markers
