# Supplementary material for: GGA1 interacts with the endosomal Na+/H+ exchanger NHE6 governing localization to the endosome compartment
Source: J Biol Chem. 2024 Jul 11;300(8):107552. doi: 10.1016/j.jbc.2024.107552 (PMC11375261; doi:10.1016/j.jbc.2024.107552)
Supplement: Supporting Table S2–S8 [file mmc3.pdf]

Table S2: NHE6/NHE1 fusion plasmids sequence

NHE6 (cyan), NHE1 (magenta), HA tag (yellow)

**Seq_ NHE6N/NHE1C-HA**
CACCCAACTGATCTTCAGCATCTTTTACTTTCACCAGCGTTTCTGGGTGAGCAAAAACAGGAAGG
CAAAATGCCGCAAAAAAGGGAATAAGGGCGACACGGAAATGTTGAATACTCATACTCTTCCTTTTT
CAATATTATTGAAGCATTTATCAGGGTTATTGTCTCATGAGCGGATACATATTTGAATGTATTTAGAA
AAATAAACAAATAGGGGTTCCGCGCACATTTCCCCGAAAAGTGCCACCTGACGTCGACGGATCG
GGAGATCTCCCGATCCCCTATGGTGCACTCTCAGTACAATCTGCTCTGATGCCGCATAGTTAAGC
CAGTATCTGCTCCCTGCTTGTGTGTTGGAGGTCGCTGAGTAGTGCGCGAGCAAAATTTAAGCTAC
AACAAGGCAAGGCTTGACCGACAATTGCATGAAGAATCTGCTTAGGGTTAGGCGTTTTGCGCTG
CTTCGCGATGTACGGGCCAGATATACGCGTTGACATTGATTATTGACTAGTTATTAATAGTAATCAA
TTACGGGGTCATTAGTTCATAGCCCATATATGGAGTTCCGCGTTACATAACTTACGGTAAATGGCC
CGCCTGGCTGACCGCCCAACGACCCCCGCCCATTGACGTCAATAATGACGTATGTTCCCATAGTA
ACGCCAATAGGGACTTTCCATTGACGTCAATGGGTGGAGTATTTACGGTAAACTGCCCACTTGGC
AGTACATCAAGTGTATCATATGCCAAGTACGCCCCCTATTGACGTCAATGACGGTAAATGGCCCGC
CTGGCATTATGCCCAGTACATGACCTTATGGGACTTTCCTACTTGGCAGTACATCTACGTATTAGT
CATCGCTATTACCATGGTGATGCGGTTTTGGCAGTACATCAATGGGCGTGGATAGCGGTTTGACT
CACGGGGATTTCCAAGTCTCCACCCCATTGACGTCAATGGGAGTTTGTTTTGGCACCAAAATCAA
CGGGACTTTCCAAAATGTCGTAACAACTCCGCCCCATTGACGCAAATGGGCGGTAGGCGTGTAC
GGTGGGAGGTCTATATAAGCAGAGCTCTCTGGCTAACTAGAGAACCCACTGCTTACTGGCTTATC
GAAATTAATACGACTCACTATAGGGAGACCCAAGCTGGCTAGTTAAGCTTGATCAAACAAGTTTGT
ACAAAAAAGCAGGCTTGAAGGAATTCGGTACCATGGCTCGGCGCGGCTGGCGGCGGGCACCCC
TCCGCCGTGGCGTCGGCAGCAGTCCCCGAGCCCGCAGGCTCATGCGGCCCCTTTGGTTGCTC
CTCGCAGTGGGCGTCTTTGACTGGGCAGGGGCTTCGGACGGCGGCGGCGGAGAGGCTAGAGC
CATGGACGAGGAGATCGTGTCCGAGAAGCAAGCCGAGGAGAGCCACCGGCAGGACAGCGCCA
ACCTGCTCATCTTCATCCTGCTGCTCACCCTCACCATTCTCACAATCTGGCTCTTCAAGCACCGC
CGGGCCCGCTTCCTGCACGAAACCGGCCTGGCTATGATTTATGGTCTTTTGGTGGGCCTTGTGC
TTCGGTATGGCATTCATGTTCCGAGTGATGTAAATAATGTGACCCTGAGCTGTGAAGTGCAGTCA
AGTCCAACTACCTTACTGGTTACTTTTGATCCAGAAGTATTTTTCAACATATTACTTCCTCCTATCAT
ATTTTATGCAGGTTATAGCCTGAAAAGGAGACATTTTTTTCGAAATCTTGGGTCTATCCTAGCATAC
GCTTTTCTTGGAACAGCAATTTCTTGTTTCGTTATTGGGTCAATAATGTATGGCTGTGTAACGCTG
ATGAAGGTAACGGGACAACTTGCAGGAGATTTTTACTTTACAGATTGCCTACTGTTTGGTGCCATT
GTATCAGCAACTGATCCAGTGACTGTTCTTGCTATATTCCACGAGCTTCAAGTTGATGTTGAACTC
TATGCACTTCTTTTTGGTGAAAGTGTCCTCAATGATGCTGTTGCCATAGTGCTGTCCTCCTCAATA
GTGGCATACCAGCCAGCTGGAGACAACAGTCACACCTTTGATGTCACAGCGATGTTCAAGTCTAT
TGGGATCTTCCTTGGAATCTTCAGTGGATCTTTTGCAATGGGTGCTGCTACTGGAGTGGTGACAG
CTTTAGTGACAAAGTTCACCAAATTACGGGAGTTCCAGTTGTTGGAGACAGGCCTGTTCTTCTTG
ATGTCCTGGAGTACCTTCCTCTTGGCTGAAGCATGGGGCTTCACAGGTGTAGTTGCAGTATTGTT
TTGTGGCATCACACAAGCACATTATACGTATAATAATTTGTCAACGGAGTCTCAGCATAGAACTAAA
CAGTTGTTTGAGCTTCTCAATTTCTTGGCAGAGAATTTCATCTTCTCCTACATGGGGCTGACACTG
TTCACCTTCCAGAACCATGTCTTTAACCCAACATTTGTAGTAGGAGCATTTGTTGCTATTTTCTTGG
GAAGAGCTGCCAATATTTACCCCTTGTCCCTCTTACTTAATTTGGGTAGAAGAAGTAAGATTGGAT
CAAATTTTCAACACATGATGATGTTTGCTGGCCTTCGTGGTGCAATGGCATTTGCCTTGGCCATTC
GAGATACTGCCACTTATGCACGGCAAATGATGTTCAGCACCACGCTTCTGATTGTGTTTTTTACCG
TGTGGGTATTTGGTGGTGGCACCACTGCAATGCTGTCATGCTTGCGGCCCCTGGTAGACCTGTT
GGCTGTGAAGAAAAAGCAAGAGACGAAGCGCTCCATCAACGAAGAGATCCACACACAGTTCCTG
GACCACCTTCTGACAGGCATCGAAGACATCTGTGGCCACTACGGTCACCACCACTGGAAGGACA
AGCTCAACCGGTTTAATAAGAAATATGTGAAGAAGTGTCTGATAGCTGGCGAGCGCTCCAAGGAG
CCCCAGCTCATTGCCTTCTACCACAAGATGGAGATGAAGCAGGCCATCGAGCTGGTGGAGAGC

GGGGGCATGGGCAAGATCCCCTCTGCCGTCTCCACCGTCTCCATGCAGAACATCCACCCCAAG
TCCCTGCCTTCCGAGCGCATCCTGCCAGCACTGTCCAAGGACAAGGAGGAGGAGATCCGCAAA
ATCCTGAGGAACAACTTGCAGAAGACCAGGCAGCGGCTGCGGTCCTACAACAGACACACGCTG
GTGGCAGACCCCTACGAGGAAGCCTGGAACCAGATGCTGCTCCGGAGGCAGAAGGCCCGGCA
GCTGGAGCAGAAGATCAACAACTACCTGACGGTGCCAGCCCACAAGCTGGACTCACCCACCAT
GTCTCGGGCCCGCATCGGCTCAGACCCACTGGCCTATGAGCCGAAGGAGGACCTGCCTGTCAT
CACCATCGACCCGGCTTCCCCGCAGTCACCCGAGTCTGTGGACCTGGTGAATGAGGAGCTGAA
GGGCAAAGTCTTAGGGTTGAGCCGGGATCCTGCAAAGGTGGCTGAGGAGGACGAGGACGACG
ATGGGGGCATCATGATGCGGAGCAAGGAGACTTCGTCCCCAGGAACCGACGATGTCTTCACCC
CCGCGCCCAGTGACAGCCCCAGCTCCCAGAGGATACAGCGCTGCCTCAGTGACCCAGGCCCA
CACCCTGAGCCTGGGGAGGGAGAACCGTTCTTCCCCAAGGGGCAGTACCTCGAGTGCGGCCG
CATGGCTAGCTACCCTTACGACGTCCCAGACTACGCTGGATCCTACCCTTACGACGTCCCAGACT
ACGCTTACCCTTACGACGTCCCAGACTACGCTTGATCAGCCTCGACTGTGCCTTCTAGTTGCCAG
CCATCTGTTGTTTGCCCCTCCCCCGTGCCTTCCTTGACCCTGGAAGGTGCCACTCCCACTGTCC
TTTCCTAATAAAATGAGGAAATTGCATCGCATTGTCTGAGTAGGTGTCATTCTATTCTGGGGGGTG
GGGTGGGGCAGGACAGCAAGGGGGAGGATTGGGAAGACAATAGCAGGCATGCTGGGGATGCG
GTGGGCTCTATGGCTTCTGAGGCGGAAAGAACCAGCTGGGGCTCTAGGGGGTATCCCCACGCG
CCCTGTAGCGGCGCATTAAGCGCGGCGGGTGTGGTGGTTACGCGCAGCGTGACCGCTACACTT
GCCAGCGCCCTAGCGCCCGCTCCTTTCGCTTTCTTCCCTTCCTTTCTCGCCACGTTCGCCGGCT
TTCCCCGTCAAGCTCTAAATCGGGGGCTCCCTTTAGGGTTCCGATTTAGTGCTTTACGGCACCTC
GACCCCAAAAAACTTGATTAGGGTGATGGTTCACGTAGTGGGCCATCGCCCTGATAGACGGTTTT
TCGCCCTTTGACGTTGGAGTCCACGTTCTTTAATAGTGGACTCTTGTTCCAAACTGGAACAACAC
TCAACCCTATCTCGGTCTATTCTTTTGATTTATAAGGGATTTTGCCGATTTCGGCCTATTGGTTAAA
AAATGAGCTGATTTAACAAAAATTTAACGCGAATTAATTCTGTGGAATGTGTGTCAGTTAGGGTGT
GGAAAGTCCCCAGGCTCCCCAGCAGGCAGAAGTATGCAAAGCATGCATCTCAATTAGTCAGCAA
CCAGGTGTGGAAAGTCCCCAGGCTCCCCAGCAGGCAGAAGTATGCAAAGCATGCATCTCAATTA
GTCAGCAACCATAGTCCCGCCCCTAACTCCGCCCATCCCGCCCCTAACTCCGCCCAGTTCCGCC
CATTCTCCGCCCCATGGCTGACTAATTTTTTTTATTTATGCAGAGGCCGAGGCCGCCTCTGCCTC
TGAGCTATTCCAGAAGTAGTGAGGAGGCTTTTTTGGAGGCCTAGGCTTTTGCAAAAAGCTCCCG
GGAGCTTGTATATCCATTTTCGGATCTGATCAAGAGACAGGATGAGGATCGTTTCGCATGATTGAA
CAAGATGGATTGCACGCAGGTTCTCCGGCCGCTTGGGTGGAGAGGCTATTCGGCTATGACTGG
GCACAACAGACAATCGGCTGCTCTGATGCCGCCGTGTTCCGGCTGTCAGCGCAGGGGCGCCC
GGTTCTTTTTGTCAAGACCGACCTGTCCGGTGCCCTGAATGAACTGCAGGACGAGGCAGCGCG
GCTATCGTGGCTGGCCACGACGGGCGTTCCTTGCGCAGCTGTGCTCGACGTTGTCACTGAAGC
GGGAAGGGACTGGCTGCTATTGGGCGAAGTGCCGGGGCAGGATCTCCTGTCATCTCACCTTGC
TCCTGCCGAGAAAGTATCCATCATGGCTGATGCAATGCGGCGGCTGCATACGCTTGATCCGGCTA
CCTGCCCATTCGACCACCAAGCGAAACATCGCATCGAGCGAGCACGTACTCGGATGGAAGCCG
GTCTTGTCGATCAGGATGATCTGGACGAAGAGCATCAGGGGCTCGCGCCAGCCGAACTGTTCG
CCAGGCTCAAGGCGCGCATGCCCGACGGCGAGGATCTCGTCGTGACCCATGGCGATGCCTGCT
TGCCGAATATCATGGTGGAAAATGGCCGCTTTTCTGGATTCATCGACTGTGGCCGGCTGGGTGT
GGCGGACCGCTATCAGGACATAGCGTTGGCTACCCGTGATATTGCTGAAGAGCTTGGCGGCGAA
TGGGCTGACCGCTTCCTCGTGCTTTACGGTATCGCCGCTCCCGATTCGCAGCGCATCGCCTTCT
ATCGCCTTCTTGACGAGTTCTTCTGAGCGGGACTCTGGGGTTCGCGAAATGACCGACCAAGCGA
CGCCCAACCTGCCATCACGAGATTTCGATTCCACCGCCGCCTTCTATGAAAGGTTGGGCTTCGG
AATCGTTTTCCGGGACGCCGGCTGGATGATCCTCCAGCGCGGGGATCTCATGCTGGAGTTCTTC
GCCCACCCCAACTTGTTTATTGCAGCTTATAATGGTTACAAATAAAGCAATAGCATCACAAATTTCA
CAAATAAAGCATTTTTTTCACTGCATTCTAGTTGTGGTTTGTCCAAACTCATCAATGTATCTTATCAT
GTCTGTATACCGTCGACCTCTAGCTAGAGCTTGGCGTAATCATGGTCATAGCTGTTTCCTGTGTGA
AATTGTTATCCGCTCACAATTCCACACAACATACGAGCCGGAAGCATAAAGTGTAAAGCCTGGGG
TGCCTAATGAGTGAGCTAACTCACATTAATTGCGTTGCGCTCACTGCCCGCTTTCCAGTCGGGAA
ACCTGTCGTGCCAGCTGCATTAATGAATCGGCCAACGCGCGGGGAGAGGCGGTTTGCGTATTG

GGCGCTCTTCCGCTTCCTCGCTCACTGACTCGCTGCGCTCGGTCGTTCGGCTGCGGCGAGCG<br>GTATCAGCTCACTCAAAGGCGGTAATACGGTTATCCACAGAATCAGGGGATAACGCAGGAAAGAA<br>CATGTGAGCAAAAGGCCAGCAAAAGGCCAGGAACCGTAAAAAGGCCGCGTTGCTGGCGTTTTT<br>CCATAGGCTCCGCCCCCCTGACGAGCATCACAAAAATCGACGCTCAAGTCAGAGGTGGCGAAA<br>CCCGACAGGACTATAAAGATACCAGGCGTTTCCCCCTGGAAGCTCCCTCGTGCGCTCTCCTGTT<br>CCGACCCTGCCGCTTACCGGATACCTGTCCGCCTTTCTCCCTTCGGGAAGCGTGGCGCTTTCTC<br>ATAGCTCACGCTGTAGGTATCTCAGTTCGGTGTAGGTCGTTCGCTCCAAGCTGGGCTGTGTGCA<br>CGAACCCCCCGTTCAGCCCGACCGCTGCGCCTTATCCGGTAACTATCGTCTTGAGTCCAACCCG<br>GTAAGACACGACTTATCGCCACTGGCAGCAGCCACTGGTAACAGGATTAGCAGAGCGAGGTATG<br>TAGGCGGTGCTACAGAGTTCTTGAAGTGGTGGCCTAACTACGGCTACACTAGAAGAACAGTATTT<br>GGTATCTGCGCTCTGCTGAAGCCAGTTACCTTCGGAAAAAGAGTTGGTAGCTCTTGATCCGGCA<br>AACAAACCACCGCTGGTAGCGGTGGTTTTTTTGTTTGCAAGCAGCAGATTACGCGCAGAAAAAAA<br>GGATCTCAAGAAGATCCTTTGATCTTTTCTACGGGGTCTGACGCTCAGTGGAACGAAAACTCACG<br>TTAAGGGATTTTGGTCATGAGATTATCAAAAAGGATCTTCACCTAGATCCTTTTAAATTAAAAATGAA<br>GTTTTAAATCAATCTAAAGTATATATGAGTAAACTTGGTCTGACAGTTACCAATGCTTAATCAGTGAG<br>GCACCTATCTCAGCGATCTGTCTATTTCGTTCATCCATAGTTGCCTGACTCCCCGTCGTGTAGATA<br>ACTACGATACGGGAGGGCTTACCATCTGGCCCCAGTGCTGCAATGATACCGCGAGACCCACGCT<br>CACCGGCTCCAGATTTATCAGCAATAAACCAGCCAGCCGGAAGGGCCGAGCGCAGAAGTGGTC<br>CTGCAACTTTATCCGCCTCCATCCAGTCTATTAATTGTTGCCGGGAAGCTAGAGTAAGTAGTTCGC<br>CAGTTAATAGTTTGCGCAACGTTGTTGCCATTGCTACAGGCATCGTGGTGTCACGCTCGTCGTTT<br>GGTATGGCTTCATTCAGCTCCGGTTCCCAACGATCAAGGCGAGTTACATGATCCCCCATGTTGTG<br>CAAAAAAGCGGTTAGCTCCTTCGGTCCTCCGATCGTTGTCAGAAGTAAGTTGGCCGCAGTGTTAT<br>CACTCATGGTTATGGCAGCACTGCATAATTCTCTTACTGTCATGCCATCCGTAAGATGCTTTTCTG<br>TGACTGGTGAGTACTCAACCAAGTCATTCTGAGAATAGTGTATGCGGCGACCGAGTTGCTCTTGC<br>CCGGCGTCAATACGGGATAATACCGCGCCACATAGCAGAACTTTAAAAGTGCTCATCATTGGAAA<br>ACGTTCTTCGGGGCGAAAACTCTCAAGGATCTTACCGCTGTTGAGATCCAGTTCGATGTAACCCA<br>CTCGTG

**Seq_ NHE1N/NHE6C-HA**
CCAACTGATCTTCAGCATCTTTTACTTTCACCAGCGTTTCTGGGTGAGCAAAAACAGGAAGGCAA<br>AATGCCGCAAAAAAGGGAATAAGGGCGACACGGAAATGTTGAATACTCATACTCTTCCTTTTTCAA<br>TATTATTGAAGCATTTATCAGGGTTATTGTCTCATGAGCGGATACATATTTGAATGTATTTAGAAAAA<br>TAAACAAATAGGGGTTCCGCGCACATTTCCCCGAAAAGTGCCACCTGACGTCGACGGATCGGGA<br>GATCTCCCGATCCCCTATGGTGCACTCTCAGTACAATCTGCTCTGATGCCGCATAGTTAAGCCAG<br>TATCTGCTCCCTGCTTGTGTGTTGGAGGTCGCTGAGTAGTGCGCGAGCAAAATTTAAGCTACAAC<br>AAGGCAAGGCTTGACCGACAATTGCATGAAGAATCTGCTTAGGGTTAGGCGTTTTGCGCTGCTT<br>CGCGATGTACGGGCCAGATATACGCGTTGACATTGATTATTGACTAGTTATTAATAGTAATCAATTA<br>CGGGGTCATTAGTTCATAGCCCATATATGGAGTTCCGCGTTACATAACTTACGGTAAATGGCCCGC<br>CTGGCTGACCGCCCAACGACCCCCGCCCATTGACGTCAATAATGACGTATGTTCCCATAGTAACG<br>CCAATAGGGACTTTCCATTGACGTCAATGGGTGGAGTATTTACGGTAAACTGCCCACTTGGCAGT<br>ACATCAAGTGTATCATATGCCAAGTACGCCCCCTATTGACGTCAATGACGGTAAATGGCCCGCCT<br>GGCATTATGCCCAGTACATGACCTTATGGGACTTTCCTACTTGGCAGTACATCTACGTATTAGTCAT<br>CGCTATTACCATGGTGATGCGGTTTTGGCAGTACATCAATGGGCGTGGATAGCGGTTTGACTCAC<br>GGGGATTTCCAAGTCTCCACCCCATTGACGTCAATGGGAGTTTGTTTTGGCACCAAAATCAACGG<br>GACTTTCCAAAATGTCGTAACAACTCCGCCCCATTGACGCAAATGGGCGGTAGGCGTGTACGGT<br>GGGAGGTCTATATAAGCAGAGCTCTCTGGCTAACTAGAGAACCCACTGCTTACTGGCTTATCGAA<br>ATTAATACGACTCACTATAGGGAGACCCAAGCTGGCTAGTTAAGCTTGATCAAACAAGTTTGTACA

AAAAAGCAGGCTTGAAGGAATTCGGTACCATGGTTCTGCGGTCTGGCATCTGTGGCCTCTCTCC
ACATCGGATCTTCCCTTCCTTACTCGTGGTGGTTGCTTTGGTGGGGCTGCTGCCTGTTCTCAGG
AGCCATGGCCTCCAGCTCAGCCCAACTGCCAGCACCATTCGAAGCTCAGAGCCACCACGAGAA
CGCTCGATTGGGGATGTCACCACCGCTCCACCGGAGGTCACCCCAGAGAGCCGCCCTGTTAAT
CATTCCGTCACTGATCATGGCATGAAGCCGCGCAAGGCCTTTCCAGTCCTGGGCATCGACTACA
CACACGTGCGCACCCCCTTCGAGATCTCCCTCTGGATCCTTCTGGCCTGCCTCATGAAGATAGG
TTTCCATGTGATCCCCACTATCTCAAGCATCGTCCCGGAGAGCTGCCTGCTGATCGTGGTGGGG
CTGCTGGTGGGGGGCCTGATCAAGGGTGTAGGCGAGACACCCCCCTTCCTGCAGTCCGACGTC
TTCTTCCTCTTCCTGCTGCCGCCCATCATCCTGGATGCGGGCTACTTCCTGCCACTGCGGCAGT
TCACAGAAAACCTGGGCACCATCCTGATCTTTGCCGTGGTGGGCACGCTGTGGAACGCCTTCTT
CCTGGGCGGCCTCATGTACGCCGTGTGCCTGGTGGGCGGTGAGCAGATCAACAACATCGGCCT
CCTGGACAACCTGCTCTTCGGCAGCATCATCTCGGCCGTGGACCCCGTGGCGGTTCTGGCTGT
CTTTGAGGAAATTCACATCAATGAGCTGCTGCACATCCTTGTTTTTGGGGAGTCCTTGCTCAATG
ACGCCGTCACTGTGGTCCTGTATCACCTCTTTGAGGAGTTTGCCAACTACGAACACGTGGGCAT
CGTGGACATCTTCCTCGGCTTCCTGAGCTTCTTCGTGGTGGCCCTGGGCGGGGTGCTTGTGGG
CGTGGTCTACGGGGTCATCGCAGCCTTCACCTCCCGATTTACCTCCCACATCCGGGTCATCGAG
CCGCTCTTCGTCTTCCTCTACAGCTACATGGCCTACTTGTCAGCCGAGCTCTTCCACCTGTCAGG
CATCATGGCGCTCATAGCCTCAGGAGTGGTGATGCGCCCCTATGTGGAGGCCAACATCTCCCAC
AAGTCCCACACCACCATCAAATACTTCCTGAAGATGTGGAGCAGCGTCAGCGAGACCCTCATCTT
CATCTTCCTCGGCGTCTCCACGGTGGCCGGCTCCCACCACTGGAACTGGACCTTCGTCATCAG
CACCCTGCTCTTCTGCCTCATCGCCCGCGTGCTGGGGGTGCTGGGCCTGACCTGGTTCATCAA
CAAGTTCCGTATCGTGAAGCTGACCCCCAAGGACCAGTTCATCATCGCCTATGGGGGCCTGCGA
GGGGCCATCGCCTTCTCTCTGGGCTACCTCCTGGACAAGAAGCACTTCCCCATGTGTGACCTGT
TCCTCACTGCCATCATCACTGTCATCTTCTTCACCGTCTTTGTGCAGGGCATGACCATTCATATCA
GGGTTGGTGTTGATTCAGACCAAGAACACTTGGGTGTTCCTGAAAATGAAAGGAGAACTACCAA
AGCAGAGAGTGCTTGGCTTTTCCGGATGTGGTACAACTTTGATCATAACTATCTGAAGCCTCTGC
TGACCCACAGCGGGCCTCCGCTGACAACAACACTCCCTGCCTGCTGTGGACCCATCGCCAGGT
GCCTCACCAGCCCCCAGGCTTACGAAAACCAGGAACAGTTGAAAGATGATGATTCTGATCTTATT
CTCAATGATGGTGACATCAGTTTGACATATGGAGATTCTACTGTGAACACTGAACCGGCCACATC
CAGCGCCCCAAGGAGATTTATGGGAAACAGTTCTGAAGATGCCTTGGATCGGGAGCTTGCATTT
GGGGACCATGAACTGGTCATTCGAGGAACACGCCTGGTTCTTCCAATGGATGATTCTGAACCCC
CGCTAAATTTGTTAGATAATACGAGACATGGTCCAGCCTACCTCGAGTGCGGCCGCATGGCTAGC
TACCCTTACGACGTCCCAGACTACGCTGGATCCTACCCTTACGACGTCCCAGACTACGCTTACCC
TTACGACGTCCCAGACTACGCTTGATCAGCCTCGACTGTGCCTTCTAGTTGCCAGCCATCTGTTG
TTTGCCCCTCCCCCGTGCCTTCCTTGACCCTGGAAGGTGCCACTCCCACTGTCCTTTCCTAATAA
AATGAGGAAATTGCATCGCATTGTCTGAGTAGGTGTCATTCTATTCTGGGGGGTGGGGTGGGGC
AGGACAGCAAGGGGGAGGATTGGGAAGACAATAGCAGGCATGCTGGGGATGCGGTGGGCTCTA
TGGCTTCTGAGGCGGAAAGAACCAGCTGGGGCTCTAGGGGGTATCCCCACGCGCCCTGTAGCG
GCGCATTAAGCGCGGCGGGTGTGGTGGTTACGCGCAGCGTGACCGCTACACTTGCCAGCGCCC
TAGCGCCCGCTCCTTTCGCTTTCTTCCCTTCCTTTCTCGCCACGTTCGCCGGCTTTCCCCGTCAA
GCTCTAAATCGGGGGCTCCCTTTAGGGTTCCGATTTAGTGCTTTACGGCACCTCGACCCCAAAAA
ACTTGATTAGGGTGATGGTTCACGTAGTGGGCCATCGCCCTGATAGACGGTTTTTCGCCCTTTGA
CGTTGGAGTCCACGTTCTTTAATAGTGGACTCTTGTTCCAAACTGGAACAACACTCAACCCTATCT
CGGTCTATTCTTTTGATTTATAAGGGATTTTGCCGATTTCGGCCTATTGGTTAAAAATGAGCTGAT

TTAACAAAAATTTAACGCGAATTAATTCTGTGGAATGTGTGTCAGTTAGGGTGTGGAAAGTCCCCA
GGCTCCCCAGCAGGCAGAAGTATGCAAAGCATGCATCTCAATTAGTCAGCAACCAGGTGTGGAA
AGTCCCCAGGCTCCCCAGCAGGCAGAAGTATGCAAAGCATGCATCTCAATTAGTCAGCAACCATA
GTCCCGCCCCTAACTCCGCCCATCCCGCCCCTAACTCCGCCCAGTTCCGCCCATTCTCCGCCCC
ATGGCTGACTAATTTTTTTTATTTATGCAGAGGCCGAGGCCGCCTCTGCCTCTGAGCTATTCCAGA
AGTAGTGAGGAGGCTTTTTTGGAGGCCTAGGCTTTTGCAAAAAGCTCCCGGGAGCTTGTATATCC
ATTTTCGGATCTGATCAAGAGACAGGATGAGGATCGTTTCGCATGATTGAACAAGATGGATTGCA
CGCAGGTTCTCCGGCCGCTTGGGTGGAGAGGCTATTCGGCTATGACTGGGCACAACAGACAAT
CGGCTGCTCTGATGCCGCCGTGTTCCGGCTGTCAGCGCAGGGGCGCCCGGTTCTTTTTGTCAA
GACCGACCTGTCCGGTGCCCTGAATGAACTGCAGGACGAGGCAGCGCGGCTATCGTGGCTGG
CCACGACGGGCGTTCCTTGCGCAGCTGTGCTCGACGTTGTCACTGAAGCGGGAAGGGACTGG
CTGCTATTGGGCGAAGTGCCGGGGCAGGATCTCCTGTCATCTCACCTTGCTCCTGCCGAGAAAG
TATCCATCATGGCTGATGCAATGCGGCGGCTGCATACGCTTGATCCGGCTACCTGCCCATTCGAC
CACCAAGCGAAACATCGCATCGAGCGAGCACGTACTCGGATGGAAGCCGGTCTTGTCGATCAG
GATGATCTGGACGAAGAGCATCAGGGGCTCGCGCCAGCCGAACTGTTCGCCAGGCTCAAGGCG
CGCATGCCCGACGGCGAGGATCTCGTCGTGACCCATGGCGATGCCTGCTTGCCGAATATCATGG
TGGAAAATGGCCGCTTTTCTGGATTCATCGACTGTGGCCGGCTGGGTGTGGCGGACCGCTATCA
GGACATAGCGTTGGCTACCCGTGATATTGCTGAAGAGCTTGGCGGCGAATGGGCTGACCGCTTC
CTCGTGCTTTACGGTATCGCCGCTCCCGATTCGCAGCGCATCGCCTTCTATCGCCTTCTTGACGA
GTTCTTCTGAGCGGGACTCTGGGGTTCGCGAAATGACCGACCAAGCGACGCCCAACCTGCCAT
CACGAGATTTCGATTCCACCGCCGCCTTCTATGAAAGGTTGGGCTTCGGAATCGTTTTCCGGGA
CGCCGGCTGGATGATCCTCCAGCGCGGGGATCTCATGCTGGAGTTCTTCGCCCACCCCAACTT
GTTTATTGCAGCTTATAATGGTTACAAATAAAGCAATAGCATCACAAATTTCACAAATAAAGCATTTT
TTTCACTGCATTCTAGTTGTGGTTTGTCCAAACTCATCAATGTATCTTATCATGTCTGTATACCGTC
GACCTCTAGCTAGAGCTTGGCGTAATCATGGTCATAGCTGTTTCCTGTGTGAAATTGTTATCCGCT
CACAATTCCACACAACATACGAGCCGGAAGCATAAAGTGTAAAGCCTGGGGTGCCTAATGAGTGA
GCTAACTCACATTAATTGCGTTGCGCTCACTGCCCGCTTTCCAGTCGGGAAACCTGTCGTGCCA
GCTGCATTAATGAATCGGCCAACGCGCGGGGAGAGGCGGTTTGCGTATTGGGCGCTCTTCCGC
TTCCTCGCTCACTGACTCGCTGCGCTCGGTCGTTCGGCTGCGGCGAGCGGTATCAGCTCACTC
AAAGGCGGTAATACGGTTATCCACAGAATCAGGGGATAACGCAGGAAAGAACATGTGAGCAAAA
GGCCAGCAAAAGGCCAGGAACCGTAAAAAGGCCGCGTTGCTGGCGTTTTTCCATAGGCTCCGC
CCCCCTGACGAGCATCACAAAAATCGACGCTCAAGTCAGAGGTGGCGAAACCCGACAGGACTAT
AAAGATACCAGGCGTTTCCCCCTGGAAGCTCCCTCGTGCGCTCTCCTGTTCCGACCCTGCCGCT
TACCGGATACCTGTCCGCCTTTCTCCCTTCGGGAAGCGTGGCGCTTTCTCATAGCTCACGCTGTA
GGTATCTCAGTTCGGTGTAGGTCGTTCGCTCCAAGCTGGGCTGTGTGCACGAACCCCCCGTTCA
GCCCGACCGCTGCGCCTTATCCGGTAACTATCGTCTTGAGTCCAACCCGGTAAGACACGACTTAT
CGCCACTGGCAGCAGCCACTGGTAACAGGATTAGCAGAGCGAGGTATGTAGGCGGTGCTACAG
AGTTCTTGAAGTGGTGGCCTAACTACGGCTACACTAGAAGAACAGTATTTGGTATCTGCGCTCTG
CTGAAGCCAGTTACCTTCGGAAAAAGAGTTGGTAGCTCTTGATCCGGCAAACAAACCACCGCTG
GTAGCGGTGGTTTTTTTGTTTGCAAGCAGCAGATTACGCGCAGAAAAAAAGGATCTCAAGAAGAT
CCTTTGATCTTTTCTACGGGGTCTGACGCTCAGTGGAACGAAAACTCACGTTAAGGGATTTTGGT
CATGAGATTATCAAAAAGGATCTTCACCTAGATCCTTTTAAATTAAAAATGAAGTTTTAAATCAATCT
AAAGTATATATGAGTAAACTTGGTCTGACAGTTACCAATGCTTAATCAGTGAGGCACCTATCTCAG
CGATCTGTCTATTTCGTTCATCCATAGTTGCCTGACTCCCCGTCGTGTAGATAACTACGATACGGG

AGGGCTTACCATCTGGCCCCAGTGCTGCAATGATACCGCGAGACCCACGCTCACCGGCTCCAG
ATTTATCAGCAATAAACCAGCCAGCCGGAAGGGCCGAGCGCAGAAGTGGTCCTGCAACTTTATC
CGCCTCCATCCAGTCTATTAATTGTTGCCGGGAAGCTAGAGTAAGTAGTTCGCCAGTTAATAGTTT
GCGCAACGTTGTTGCCATTGCTACAGGCATCGTGGTGTCACGCTCGTCGTTTGGTATGGCTTCAT
TCAGCTCCGGTTCCCAACGATCAAGGCGAGTTACATGATCCCCCATGTTGTGCAAAAAAGCGGT
TAGCTCCTTCGGTCCTCCGATCGTTGTCAGAAGTAAGTTGGCCGCAGTGTTATCACTCATGGTTA
TGGCAGCACTGCATAATTCTCTTACTGTCATGCCATCCGTAAGATGCTTTTCTGTGACTGGTGAGT
ACTCAACCAAGTCATTCTGAGAATAGTGTATGCGGCGACCGAGTTGCTCTTGCCCGGCGTCAATA
CGGGATAATACCGCGCCACATAGCAGAACTTTAAAAGTGCTCATCATTGGAAAACGTTCTTCGGG
GCGAAAACTCTCAAGGATCTTACCGCTGTTGAGATCCAGTTCGATGTAACCCACTCGTGCAC

Table S3: HAP1-GGA1 Knockout cell line Engineered using CRISPR/Cas9

Cell line	gRNA	Targeted exon	Mutation	PCR primers	Sequencing primer
GGA1-KO1 (GGA1/GGA1double knockout)	AAGCGGTTCCACGACGAAGT	Exon 4 (NM_013365)	134bp deletion in exon 4, causing a frameshift	PCR_fwd: TATGTAGGTCGCCTGACCCTATCTC PCR_bwd: CAATCTGAAGGTCCTCTTTCCTCTC	TATGTAGGTCGCCTGACCCTATCTC
	CCCTTCTCACCGTCAAGGCC	Exon 3 (NM_001001561)	2bp deletion in exon 3, causing a frameshift	PCR_fwd: TTTGCATATGCCTGCAGAAACCTTA PCR_bwd: CCATCTTAGCACAACTCTCTTGGG	TTTGCATATGCCTGCAGAAACCTTA
GGA1-KO2	TTGGCTGCGCGGAGGTCTTC	Exon 7 (NM_013365)	1bp insertion in exon 7, causing a frameshift	PCR_fwd: TTCTCACCATTTCACAGAGAGCTTA PCR_bwd: TCTAACTTTTGACGAAGAGGTAGCC	TTCTCACCATTTCACAGAGAGCTTA

Table S4: GGA1 plasmids sequence

GGA1 (cyan, magenta), c-Myc or GFP tag (green)

Seq_mGGA1-FL-GFP

TACAGCGTCGCCAGCGCAGCTCTCTCTAGCGACGGCCGCATCTTCACTGGTGTCAATGTATATCATTTTACT
GGGGGACCTTGTGCAGAACTCGTGGTGCTGGGCACTGCTGCTGCTGCGGCAGCTGGCAACCTGACTTGT
ATCGTCGCGATCGGAAATGAGAACAGGGGCATCTTGAGCCCCTGCGGACGGTGCCGACAGGTGCTTCTC
GATCTGCATCCTGGGATCAAAGCCATAGTGAAGGACAGTGATGGACAGCCGACGGCAGTTGGGATTCGTG
AATTGCTGCCCTCTGGTTATGTGTGGGAGGGCTAAGCACTTCGTGGCCGAGGAGCAGGACTGACACGTGC
TACGAGATTTCGATTCCACCGCCGCCTTCTATGAAAGGTTGGGCTTCGGAATCGTTTTCCGGGACGCCGGC
TGGATGATCCTCCAGCGCGGGGATCTCATGCTGGAGTTCTTCGCCCACCCCAACTTGTTTATTGCAGCTTAT
AATGGTTACAAATAAAGCAATAGCATCACAAATTTCACAAATAAAGCATTTTTTTCACTGCATTCTAGTTGTGG
TTTGTCCAAACTCATCAATGTATCTTATCATGTCTGTATACCGTCGACCTCTAGCTAGAGCTTGGCGTAATCAT
GGTCATAGCTGTTTCCTGTGTGAAATTGTTATCCGCTCACAATTCCACACAACATACGAGCCGGAAGCATAA
AGTGTAAAGCCTGGGGTGCCTAATGAGTGAGCTAACTCACATTAATTGCGTTGCGCTCACTGCCCGCTTTC
CAGTCGGGAAACCTGTCGTGCCAGCTGCATTAATGAATCGGCCAACGCGCGGGGAGAGGCGGTTTGCGT
ATTGGGCGCTCTTCCGCTTCCTCGCTCACTGACTCGCTGCGCTCGGTCGTTCGGCTGCGGCGAGCGGTAT
CAGCTCACTCAAAGGCGGTAATACGGTTATCCACAGAATCAGGGGATAACGCAGGAAAGAACATGTGAGCA
AAAGGCCAGCAAAAGGCCAGGAACCGTAAAAAGGCCGCGTTGCTGGCGTTTTTCCATAGGCTCCGCCCCC
CTGACGAGCATCACAAAAATCGACGCTCAAGTCAGAGGTGGCGAAACCCGACAGGACTATAAAGATACCAG
GCGTTTCCCCCTGGAAGCTCCCTCGTGCGCTCTCCTGTTCCGACCCTGCCGCTTACCGGATACCTGTCCG
CCTTTCTCCCTTCGGGAAGCGTGGCGCTTTCTCATAGCTCACGCTGTAGGTATCTCAGTTCGGTGTAGGTC
GTTCGCTCCAAGCTGGGCTGTGTGCACGAACCCCCCGTTCAGCCCGACCGCTGCGCCTTATCCGGTAACT
ATCGTCTTGAGTCCAACCCGGTAAGACACGACTTATCGCCACTGGCAGCAGCCACTGGTAACAGGATTAGC
AGAGCGAGGTATGTAGGCGGTGCTACAGAGTTCTTGAAGTGGTGGCCTAACTACGGCTACACTAGAAGAAC
AGTATTTGGTATCTGCGCTCTGCTGAAGCCAGTTACCTTCGGAAAAAGAGTTGGTAGCTCTTGATCCGGCAA
ACAAACCACCGCTGGTAGCGGTGGTTTTTTTGTTTGCAAGCAGCAGATTACGCGCAGAAAAAAAGGATCTC
AAGAAGATCCTTTGATCTTTTCTACGGGGTCTGACGCTCAGTGGAACGAAAACTCACGTTAAGGGATTTTG
GTCATGAGATTATCAAAAAGGATCTTCACCTAGATCCTTTTAAATTAAAAATGAAGTTTTAAATCAATCTAAAGT
ATATATGAGTAAACTTGGTCTGACAGTTACCAATGCTTAATCAGTGAGGCACCTATCTCAGCGATCTGTCTAT
TTCGTTCATCCATAGTTGCCTGACTCCCCGTCGTGTAGATAACTACGATACGGGAGGGCTTACCATCTGGCC
CCAGTGCTGCAATGATACCGCGAGACCCACGCTCACCGGCTCCAGATTTATCAGCAATAAACCAGCCAGCC
GGAAGGGCCGAGCGCAGAAGTGGTCCTGCAACTTTATCCGCCTCCATCCAGTCTATTAATTGTTGCCGGGA
AGCTAGAGTAAGTAGTTCGCCAGTTAATAGTTTGCGCAACGTTGTTGCCATTGCTACAGGCATCGTGGTGTC
ACGCTCGTCGTTTGGTATGGCTTCATTCAGCTCCGGTTCCCAACGATCAAGGCGAGTTACATGATCCCCCA
TGTTGTGCAAAAAAGCGGTTAGCTCCTTCGGTCCTCCGATCGTTGTCAGAAGTAAGTTGGCCGCAGTGTTA
TCACTCATGGTTATGGCAGCACTGCATAATTCTCTTACTGTCATGCCATCCGTAAGATGCTTTTCTGTGACTG
GTGAGTACTCAACCAAGTCATTCTGAGAATAGTGTATGCGGCGACCGAGTTGCTCTTGCCCGGCGTCAATA
CGGGATAATACCGCGCCACATAGCAGAACTTTAAAAGTGCTCATCATTGGAAAACGTTCTTCGGGGCGAAA
ACTCTCAAGGATCTTACCGCTGTTGAGATCCAGTTCGATGTAACCCACTCGTGCACCCAACTGATCTTCAGC
ATCTTTTACTTTCACCAGCGTTTCTGGGTGAGCAAAAACAGGAAGGCAAAATGCCGCAAAAAAGGGAATAA
GGGCGACACGGAAATGTTGAATACTCATACTCTTCCTTTTTCAATATTATTGAAGCATTTATCAGGGTTATTGT
CTCATGAGCGGATACATATTTGAATGTATTTAGAAAAATAAACAAATAGGGGTTCCGCGCACATTTCCCCGAA
AAGTGCCACCTGACGTCGACGGATCGGGAGATCTCCCGATCCCCTATGGTGCACTCTCAGTACAATCTGCT
CTGATGCCGCATAGTTAAGCCAGTATCTGCTCCCTGCTTGTGTGTTGGAGGTCGCTGAGTAGTGCGCGAGC
AAAATTTAAGCTACAACAAGGCAAGGCTTGACCGACAATTGCATGAAGAATCTGCTTAGGGTTAGGCGTTTT
GCGCTGCTTCGCGATGTACGGGCCAGATATACGCGTTGACATTGATTATTGACTAGTTATTAATAGTAATCAAT

TACGGGGTCATTAGTTCATAGCCCATATATGGAGTTCCGCGTTACATAACTTACGGTAAATGGCCCGCCTGG
CTGACCGCCCAACGACCCCCGCCCATTGACGTCAATAATGACGTATGTTCCCATAGTAACGCCAATAGGGA
CTTTCCATTGACGTCAATGGGTGGAGTATTTACGGTAAACTGCCCACTTGGCAGTACATCAAGTGTATCATAT
GCCAAGTACGCCCCCTATTGACGTCAATGACGGTAAATGGCCCGCCTGGCATTATGCCCAGTACATGACCT
TATGGGACTTTCCTACTTGGCAGTACATCTACGTATTAGTCATCGCTATTACCATGGTGATGCGGTTTTGGCA
GTACATCAATGGGCGTGGATAGCGGTTTGACTCACGGGGATTTCCAAGTCTCCACCCCATTGACGTCAATG
GGAGTTTGTTTTGGCACCAAAATCAACGGGACTTTCCAAAATGTCGTAACAACTCCGCCCCATTGACGCAA
ATGGGCGGTAGGCGTGTACGGTGGGAGGTCTATATAAGCAGAGCTCTCTGGCTAACTAGAGAACCCACTGC
TTACTGGCTTATCGAAATTAATACGACTCACTATAGGGAGACCCAAGCTGGCTAGTTAAGCTGAGCATCAACA
AGTTTGTACAAAAAAGCAGGCTCCGAATTCGCCCTTGCCGCCATGGAGCCCGCGATGGAGCCGGAGACTC
TGGAGGCACGAATCAACAGAGCCACAAATCCCCTGAACAAGGAGCTGAACTGGGCCAGCATCAACAGTTT
CTGCGAGCAGCTCAACGAAGACTTTGAGGGGCCTCCACTTGCCACTCGCTTGCTGGCCCACAAGATCCAG
TCCCCACAGGAATGGGAAGCCATCCAGGCCTTGACGGTTCTGGAGACGTGCATGAAGAGCTGCGGCAAG
AGGTTCCATGATGAGGTGGGCAAGTTCCGCTTCCTCAACGAGCTCATCAAGGTTGTGTCTCCCAAGTACTT
GGGCTCCCGGACATCTGAGAAGGTGAAGAGTAAGATCTTGGAGCTGCTGTACAGCTGGACGGTTTGCCTG
CCTGAGGAGGTGAAGATTGCAGAAGCCTACCAGATGCTGAAGAAGCAGGGGATTGTGAAGTCGGACCCCA
AGCTTCCAGAGGATGCCATCTTTCCCCTCCCCCCTCCCCGGCCCAAGAATGTGATCTTTGAAGATGAGGAG
AAGTCCAAGATGCTGGCCCGCCTGCTGAAGAGCTCACACCCTGAGGACCTCCGGGCTGCCAATAAGCTCA
TCAAAGAGATGGTGCAGGAGGACCAGAAGCGGATGGAAAAGATCTCCAAGCGGGTGAATGCCATCGAGGA
GGTCAACAACAATGTGAAGTTGCTGACAGAGATGGTGATGAGCCACAGCCAGGGTGCTGCATCCAGCAGC
AGTGAGGACCTCATGAAGGAACTGTACCAGCGCTGTGAGCGCATGCGACCCACACTCTTCCGACTGGCCA
GTGACACGGAAGACAATGATGAGGCCTTAGCTGAGATCCTGCAGGCTAATGACAATCTCACCCAGGTGATC
AACCTGTACAAGCAGCTGGTCCGGGGCGAGGAGGTCAACGGTGATGCCACAGCCAGCTCCATTCCTGGA
AGCACGTCAGCCCTGCTGGACCTCTCAGGCCTGGACCTCCCTCCCCCGGGCACCACCCAGCCAGCCACG
CCCACCCGCCCTGGCAACCAGAGCAGTCCTGAGCAGCTCAGTGCCTCGGTGTCCCTGCTTGATGACGAG
CTCATGTCTCTGGGCCTAAGTGACCCGACACCACCTTCAGGCACCAGCTCAGATAGTGTGGGGTGGGACA
ACTTCCAGTCATCAGATGGCACTGAATCCTCAGTCCCTCCTCCAGCCCAGGCCCCCAGCATGGACTGCCG
ACCCCCAGCCCAGGCCCCTCCACCAACGAGCAGTGGCCTGGACGACCTGGACCTCTTGGGGAAAACCCT
TATGCAGCAGGCTCTGCCTCCGGAAGCCCAGCAAGTGCGGTGGGAGAAGCAGCAGCCAGCCCCCCGGC
TCACCCTCCGTGACCTGCAGAGTAAGAGCAGCTCGCCCAGCCCAGGAGCCGCCAGCCTCCTCCACACCA
CGTCCCCAGAGCCCCCTGGGCCTCCACCTCAGGCCACACCCACTGAGTTCTCCCTAACCAGCATCACTGT
GCCCCTGGAGTCTATCAAACCCAGCAGCATCTTGCCAGTGACCGTTTATGACCAGCATGGCTTCCGTGTCC
TCTTCCATTTTGCTCGGGACCCACTGCCAGGGCGCTCCGATGTGCTGGTGGTGGTGGTCTCTATGCTGAG
CACGGCGCCCCAGCCCATCCGGAACATCGTTTTCCAGTCAGCCGTCCCCAAGGTCATGAAGGTGAGGCT
GCAGCCACCTTCCGGCACAGAGCTGCCAGCGTTCAACCCCATCGTCCACCCCTCAGCCATCACCCAGGTC
CTGCTCCTTGCTAACCCCCAGAAGGAGAAGGTTCGCCTCCGCTACAAGCTCATCTTCACTATGGGCGACCA
GACCTACAATGAGATGGGAGATGTGGATCAGTTCCCCCCACCAGAGACCTGGGGGAGCCTCAAGGGCGA
ATTCGACCCAGCTTTCTTGTACAAAGTGGTTGATGCTGTTAACATGGTGAGCAAGGGCGAGGAGCTGTTCA
CCGGGGTGGTGCCCATCCTGGTCGAGCTGGACGGCGACGTAAACGGCCACAAGTTCAGCGTGTCCGGC
GAGGGCGAGGGCGATGCCACCTACGGCAAGCTGACCCTGAAGTTCATCTGCACCACCGGCAAGCTGCCC
GTGCCCTGGCCCACCCTCGTGACCACCTTCACCTACGGCGTGCAGTGCTTCGCCCGCTACCCCGACCAC
ATGAAGCAGCACGACTTCTTCAAGTCCGCCATGCCCGAAGGCTACGTCCAGGAGCGCACCATCTTCTTCAA
GGACGACGGCAACTACAAGACCCGCGCCGAGGTGAAGTTCGAGGGCGACACCCTGGTGAACCGCATCGA
GCTGAAGGGCATCGACTTCAAGGAGGACGGCAACATCCTGGGGCACAAGCTGGAGTACAACTACAACAGC
CACAAGGTCTATATCACCGCCGACAAGCAGAAGAACGGCATCAAGGTGAACTTCAAGACCCGCCACAACAT
CGAGGACGGCAGCGTGCAGCTCGCCGACCACTACCAGCAGAACACCCCCATCGGCGACGGCCCCGTGC
TGCTGCCCGACAACCACTACCTGAGCACCCAGTCCGCCCTGAGCAAAGACCCCAACGAGAAGCGCGATC
ACATGGTCCTGCTGGAGTTCGTGACCGCCGCCGGGATCACTCTCGGCATGGACGAGCTGTACAAGTAATG

ATAAGTTTAAACGGGGGAGGCTAACTGAAACACGGAAGGAGACAATACCGGAAGGAACCCGCGCTATGAC
GGCAATAAAAAGACAGAATAAAACGCACGGGTGTTGGGTCGTTTGTTCATAAACGCGGGGTTCGGTCCCAG
GGCTGGCACTCTGTCGATACCCCACCGAGACCCCATTGGGGCCAATACGCCCGCGTTTCTTCCTTTTCCC
CACCCCACCCCCCAAGTTCGGGTGAAGGCCCAGGGCTCGCAGCCAACGTCGGGGCGGCAGGCCCTGCC
ATAGCAGATCTGCGCAGCTGGGGCTCTAGGGGGTATCCCCACGCGCCCTGTAGCGGCGCATTAAGCGCG
GCGGGTGTGGTGGTTACGCGCAGCGTGACCGCTACACTTGCCAGCGCCCTAGCGCCCGCTCCTTTCGCT
TTCTTCCCTTCCTTTCTCGCCACGTTCGCCGGCTTTCCCCGTCAAGCTCTAAATCGGGGGCTCCCTTTAGG
GTTCCGATTTAGTGCTTTACGGCACCTCGACCCCAAAAAACTTGATTAGGGTGATGGTTCACGTAGTGGGC
CATCGCCCTGATAGACGGTTTTTCGCCCTTTGACGTTGGAGTCCACGTTCTTTAATAGTGGACTCTTGTTCC
AAACTGGAACAACACTCAACCCTATCTCGGTCTATTCTTTTGATTTATAAGGGATTTTGCCGATTTCGGCCTA
TTGGTTAAAAAATGAGCTGATTTAACAAAAATTTAACGCGAATTAATTCTGTGGAATGTGTGTCAGTTAGGGT
GTGGAAAGTCCCCAGGCTCCCCAGCAGGCAGAAGTATGCAAAGCATGCATCTCAATTAGTCAGCAACCAG
GTGTGGAAAGTCCCCAGGCTCCCCAGCAGGCAGAAGTATGCAAAGCATGCATCTCAATTAGTCAGCAACCA
TAGTCCCGCCCCTAACTCCGCCCATCCCGCCCCTAACTCCGCCCAGTTCCGCCCATTCTCCGCCCCATGG
CTGACTAATTTTTTTTATTTATGCAGAGGCCGAGGCCGCCTCTGCCTCTGAGCTATTCCAGAAGTAGTGAGG
AGGCTTTTTTGGAGGCCTAGGCTTTTGCAAAAAGCTCCCGGGAGCTTGTATATCCATTTTCGGATCTGATCA
GCACGTGTTGACAATTAATCATCGGCATAGTATATCGGCATAGTATAATACGACAAGGTGAGGAACTAAACCA
TGGCCAAGCCTTTGTCTCAAGAAGAATCCACCCTCATTGAAAGAGCAACGGCTACAATCAACAGCATCCCC
ATCTCTGAAGAC

Seq_mGGA1-VHS_GFP

TACAGCGTCGCCAGCGCAGCTCTCTCTAGCGACGGCCGCATCTTCACTGGTGTCAATGTATATCATTTTACT
GGGGGACCTTGTGCAGAACTCGTGGTGCTGGGCACTGCTGCTGCTGCGGCAGCTGGCAACCTGACTTGT
ATCGTCGCGATCGGAAATGAGAACAGGGGCATCTTGAGCCCCTGCGGACGGTGCCGACAGGTGCTTCTC
GATCTGCATCCTGGGATCAAAGCCATAGTGAAGGACAGTGATGGACAGCCGACGGCAGTTGGGATTCGTG
AATTGCTGCCCTCTGGTTATGTGTGGGAGGGCTAAGCACTTCGTGGCCGAGGAGCAGGACTGACACGTGC
TACGAGATTTCGATTCCACCGCCGCCTTCTATGAAAGGTTGGGCTTCGGAATCGTTTTCCGGGACGCCGGC
TGGATGATCCTCCAGCGCGGGGATCTCATGCTGGAGTTCTTCGCCCACCCCAACTTGTTTATTGCAGCTTAT
AATGGTTACAAATAAAGCAATAGCATCACAAATTTCACAAATAAAGCATTTTTTTCACTGCATTCTAGTTGTGG
TTTGTCCAAACTCATCAATGTATCTTATCATGTCTGTATACCGTCGACCTCTAGCTAGAGCTTGGCGTAATCAT
GGTCATAGCTGTTTCCTGTGTGAAATTGTTATCCGCTCACAATTCCACACAACATACGAGCCGGAAGCATAA
AGTGTAAAGCCTGGGGTGCCTAATGAGTGAGCTAACTCACATTAATTGCGTTGCGCTCACTGCCCGCTTTC
CAGTCGGGAAACCTGTCGTGCCAGCTGCATTAATGAATCGGCCAACGCGCGGGGAGAGGCGGTTTGCGT
ATTGGGCGCTCTTCCGCTTCCTCGCTCACTGACTCGCTGCGCTCGGTCGTTCGGCTGCGGCGAGCGGTAT
CAGCTCACTCAAAGGCGGTAATACGGTTATCCACAGAATCAGGGGATAACGCAGGAAAGAACATGTGAGCA
AAAGGCCAGCAAAAGGCCAGGAACCGTAAAAAGGCCGCGTTGCTGGCGTTTTTCCATAGGCTCCGCCCCC
CTGACGAGCATCACAAAAATCGACGCTCAAGTCAGAGGTGGCGAAACCCGACAGGACTATAAAGATACCAG
GCGTTTCCCCCTGGAAGCTCCCTCGTGCGCTCTCCTGTTCCGACCCTGCCGCTTACCGGATACCTGTCCG
CCTTTCTCCCTTCGGGAAGCGTGGCGCTTTCTCATAGCTCACGCTGTAGGTATCTCAGTTCGGTGTAGGTC
GTTCGCTCCAAGCTGGGCTGTGTGCACGAACCCCCCGTTCAGCCCGACCGCTGCGCCTTATCCGGTAACT
ATCGTCTTGAGTCCAACCCGGTAAGACACGACTTATCGCCACTGGCAGCAGCCACTGGTAACAGGATTAGC
AGAGCGAGGTATGTAGGCGGTGCTACAGAGTTCTTGAAGTGGTGGCCTAACTACGGCTACACTAGAAGAAC
AGTATTTGGTATCTGCGCTCTGCTGAAGCCAGTTACCTTCGGAAAAAGAGTTGGTAGCTCTTGATCCGGCAA
ACAAACCACCGCTGGTAGCGGTGGTTTTTTTGTTTGCAAGCAGCAGATTACGCGCAGAAAAAAAGGATCTC
AAGAAGATCCTTTGATCTTTTCTACGGGGTCTGACGCTCAGTGGAACGAAAACTCACGTTAAGGGATTTTG
GTCATGAGATTATCAAAAAGGATCTTCACCTAGATCCTTTTAAATTAAAAATGAAGTTTTAAATCAATCTAAAGT

ATATATGAGTAAACTTGGTCTGACAGTTACCAATGCTTAATCAGTGAGGCACCTATCTCAGCGATCTGTCTAT
TTCGTTCATCCATAGTTGCCTGACTCCCCGTCGTGTAGATAACTACGATACGGGAGGGCTTACCATCTGGCC
CCAGTGCTGCAATGATACCGCGAGACCCACGCTCACCGGCTCCAGATTTATCAGCAATAAACCAGCCAGCC
GGAAGGGCCGAGCGCAGAAGTGGTCCTGCAACTTTATCCGCCTCCATCCAGTCTATTAATTGTTGCCGGGA
AGCTAGAGTAAGTAGTTCGCCAGTTAATAGTTTGCGCAACGTTGTTGCCATTGCTACAGGCATCGTGGTGTC
ACGCTCGTCGTTTGGTATGGCTTCATTCAGCTCCGGTTCCCAACGATCAAGGCGAGTTACATGATCCCCCA
TGTTGTGCAAAAAAGCGGTTAGCTCCTTCGGTCCTCCGATCGTTGTCAGAAGTAAGTTGGCCGCAGTGTTA
TCACTCATGGTTATGGCAGCACTGCATAATTCTCTTACTGTCATGCCATCCGTAAGATGCTTTTCTGTGACTG
GTGAGTACTCAACCAAGTCATTCTGAGAATAGTGTATGCGGCGACCGAGTTGCTCTTGCCCGGCGTCAATA
CGGGATAATACCGCGCCACATAGCAGAACTTTAAAAGTGCTCATCATTGGAAAACGTTCTTCGGGGCGAAA
ACTCTCAAGGATCTTACCGCTGTTGAGATCCAGTTCGATGTAACCCACTCGTGCACCCAACTGATCTTCAGC
ATCTTTTACTTTCACCAGCGTTTCTGGGTGAGCAAAAACAGGAAGGCAAAATGCCGCAAAAAAGGGAATAA
GGGCGACACGGAAATGTTGAATACTCATACTCTTCCTTTTTCAATATTATTGAAGCATTTATCAGGGTTATTGT
CTCATGAGCGGATACATATTTGAATGTATTTAGAAAAATAAACAAATAGGGGTTCCGCGCACATTTCCCCGAA
AAGTGCCACCTGACGTCGACGGATCGGGAGATCTCCCGATCCCCTATGGTGCACTCTCAGTACAATCTGCT
CTGATGCCGCATAGTTAAGCCAGTATCTGCTCCCTGCTTGTGTGTTGGAGGTCGCTGAGTAGTGCGCGAGC
AAAATTTAAGCTACAACAAGGCAAGGCTTGACCGACAATTGCATGAAGAATCTGCTTAGGGTTAGGCGTTTT
GCGCTGCTTCGCGATGTACGGGCCAGATATACGCGTTGACATTGATTATTGACTAGTTATTAATAGTAATCAAT
TACGGGGTCATTAGTTCATAGCCCATATATGGAGTTCCGCGTTACATAACTTACGGTAAATGGCCCGCCTGG
CTGACCGCCCAACGACCCCCGCCCATTGACGTCAATAATGACGTATGTTCCCATAGTAACGCCAATAGGGA
CTTTCCATTGACGTCAATGGGTGGAGTATTTACGGTAAACTGCCCACTTGGCAGTACATCAAGTGTATCATAT
GCCAAGTACGCCCCCTATTGACGTCAATGACGGTAAATGGCCCGCCTGGCATTATGCCCAGTACATGACCT
TATGGGACTTTCCTACTTGGCAGTACATCTACGTATTAGTCATCGCTATTACCATGGTGATGCGGTTTTGGCA
GTACATCAATGGGCGTGGATAGCGGTTTGACTCACGGGGATTTCCAAGTCTCCACCCCATTGACGTCAATG
GGAGTTTGTTTTGGCACCAAAATCAACGGGACTTTCCAAAATGTCGTAACAACTCCGCCCCATTGACGCAA
ATGGGCGGTAGGCGTGTACGGTGGGAGGTCTATATAAGCAGAGCTCTCTGGCTAACTAGAGAACCCACTGC
TTACTGGCTTATCGAAATTAATACGACTCACTATAGGGAGACCCAAGCTGGCTAGTTAAGCTGAGCATCAACA
AGTTTGTACAAAAAAGCAGGCTCCGAATTCGCCCTTGCCGCCATGGCCACAAATCCCCTGAACAAGGAGCT
GAACTGGGCCAGCATCAACAGTTTCTGCGAGCAGCTCAACGAAGACTTTGAGGGGCCTCCACTTGCCACT
CGCTTGCTGGCCCACAAGATCCAGTCCCCACAGGAATGGGAAGCCATCCAGGCCTTGACGGTTCTGGAGA
CGTGCATGAAGAGCTGCGGCAAGAGGTTCCATGATGAGGTGGGCAAGTTCCGCTTCCTCAACGAGCTCAT
CAAGGTTGTGTCTCCCAAGTACTTGGGCTCCCGGACATCTGAGAAGGTGAAGAGTAAGATCTTGGAGCTG
CTGTACAGCTGGACGGTTTGCCTGCCTGAGGAGGTGAAGATTGCAGAAGCCTACCAGATGCTGAAGAAGC
AGGGGATTGTGAAGTCGAAGGGCGAATTCGACCCAGCTTTCTTGTACAAAGTGGTTGATGCTGTTAACATG
GTGAGCAAGGGCGAGGAGCTGTTCACCGGGGTGGTGCCCATCCTGGTCGAGCTGGACGGCGACGTAAA
CGGCCACAAGTTCAGCGTGTCCGGCGAGGGCGAGGGCGATGCCACCTACGGCAAGCTGACCCTGAAGTT
CATCTGCACCACCGGCAAGCTGCCCGTGCCCTGGCCCACCCTCGTGACCACCTTCACCTACGGCGTGCA
GTGCTTCGCCCGCTACCCCGACCACATGAAGCAGCACGACTTCTTCAAGTCCGCCATGCCCGAAGGCTAC
GTCCAGGAGCGCACCATCTTCTTCAAGGACGACGGCAACTACAAGACCCGCGCCGAGGTGAAGTTCGAG
GGCGACACCCTGGTGAACCGCATCGAGCTGAAGGGCATCGACTTCAAGGAGGACGGCAACATCCTGGGG
CACAAGCTGGAGTACAACTACAACAGCCACAAGGTCTATATCACCGCCGACAAGCAGAAGAACGGCATCAA
GGTGAACTTCAAGACCCGCCACAACATCGAGGACGGCAGCGTGCAGCTCGCCGACCACTACCAGCAGAA
CACCCCCATCGGCGACGGCCCCGTGCTGCTGCCCGACAACCACTACCTGAGCACCCAGTCCGCCCTGAG
CAAAGACCCCAACGAGAAGCGCGATCACATGGTCCTGCTGGAGTTCGTGACCGCCGCCGGGATCACTCT
CGGCATGGACGAGCTGTACAAGTAATGATAAGTTTAAACGGGGGAGGCTAACTGAAACACGGAAGGAGAC
AATACCGGAAGGAACCCGCGCTATGACGGCAATAAAAAGACAGAATAAAACGCACGGGTGTTGGGTCGTTT
GTTCATAAACGCGGGGTTCGGTCCCAGGGCTGGCACTCTGTCGATACCCCACCGAGACCCCATTGGGGCC
AATACGCCCGCGTTTCTTCCTTTTCCCCACCCCACCCCCCAAGTTCGGGTGAAGGCCCAGGGCTCGCAGC

CAACGTCGGGGCGGCAGGCCCTGCCATAGCAGATCTGCGCAGCTGGGGCTCTAGGGGGTATCCCCACGC
GCCCTGTAGCGGCGCATTAAGCGCGGCGGGTGTGGTGGTTACGCGCAGCGTGACCGCTACACTTGCCAG
CGCCCTAGCGCCCGCTCCTTTCGCTTTCTTCCCTTCCTTTCTCGCCACGTTCGCCGGCTTTCCCCGTCAAG
CTCTAAATCGGGGGCTCCCTTTAGGGTTCCGATTTAGTGCTTTACGGCACCTCGACCCCAAAAAACTTGATT
AGGGTGATGGTTCACGTAGTGGGCCATCGCCCTGATAGACGGTTTTTCGCCCTTTGACGTTGGAGTCCAC
GTTCTTTAATAGTGGACTCTTGTTCCAAACTGGAACAACACTCAACCCTATCTCGGTCTATTCTTTTGATTTAT
AAGGGATTTTGCCGATTTCGGCCTATTGGTTAAAAAATGAGCTGATTTAACAAAAATTTAACGCGAATTAATTC
TGTGGAATGTGTGTCAGTTAGGGTGTGGAAAGTCCCCAGGCTCCCCAGCAGGCAGAAGTATGCAAAGCAT
GCATCTCAATTAGTCAGCAACCAGGTGTGGAAAGTCCCCAGGCTCCCCAGCAGGCAGAAGTATGCAAAGC
ATGCATCTCAATTAGTCAGCAACCATAGTCCCGCCCCTAACTCCGCCCATCCCGCCCCTAACTCCGCCCAG
TTCCGCCCATTCTCCGCCCCATGGCTGACTAATTTTTTTTATTTATGCAGAGGCCGAGGCCGCCTCTGCCTC
TGAGCTATTCCAGAAGTAGTGAGGAGGCTTTTTTGGAGGCCTAGGCTTTTGCAAAAAGCTCCCGGGAGCTT
GTATATCCATTTTCGGATCTGATCAGCACGTGTTGACAATTAATCATCGGCATAGTATATCGGCATAGTATAATA
CGACAAGGTGAGGAACTAAACCATGGCCAAGCCTTTGTCTCAAGAAGAATCCACCCTCATTGAAAGAGCAA
CGGCTACAATCAACAGCATCCCCATCTCTGAAGAC

Seq_mGGA1-GAT_GFP

CGTGAATTGCTGCCCTCTGGTTATGTGTGGGAGGGCTAAGCACTTCGTGGCCGAGGAGCAGGACTGACAC
GTGCTACGAGATTTCGATTCCACCGCCGCCTTCTATGAAAGGTTGGGCTTCGGAATCGTTTTCCGGGACGC
CGGCTGGATGATCCTCCAGCGCGGGGATCTCATGCTGGAGTTCTTCGCCCACCCCAACTTGTTTATTGCAG
CTTATAATGGTTACAAATAAAGCAATAGCATCACAAATTTCACAAATAAAGCATTTTTTTCACTGCATTCTAGTT
GTGGTTTGTCCAAACTCATCAATGTATCTTATCATGTCTGTATACCGTCGACCTCTAGCTAGAGCTTGGCGTA
ATCATGGTCATAGCTGTTTCCTGTGTGAAATTGTTATCCGCTCACAATTCCACACAACATACGAGCCGGAAG
CATAAAGTGTAAAGCCTGGGGTGCCTAATGAGTGAGCTAACTCACATTAATTGCGTTGCGCTCACTGCCCG
CTTTCCAGTCGGGAAACCTGTCGTGCCAGCTGCATTAATGAATCGGCCAACGCGCGGGGAGAGGCGGTTT
GCGTATTGGGCGCTCTTCCGCTTCCTCGCTCACTGACTCGCTGCGCTCGGTCGTTCGGCTGCGGCGAGC
GGTATCAGCTCACTCAAAGGCGGTAATACGGTTATCCACAGAATCAGGGGATAACGCAGGAAAGAACATGT
GAGCAAAAGGCCAGCAAAAGGCCAGGAACCGTAAAAAGGCCGCGTTGCTGGCGTTTTTCCATAGGCTCCG
CCCCCCTGACGAGCATCACAAAAATCGACGCTCAAGTCAGAGGTGGCGAAACCCGACAGGACTATAAAGA
TACCAGGCGTTTCCCCCTGGAAGCTCCCTCGTGCGCTCTCCTGTTCCGACCCTGCCGCTTACCGGATACC
TGTCCGCCTTTCTCCCTTCGGGAAGCGTGGCGCTTTCTCATAGCTCACGCTGTAGGTATCTCAGTTCGGTG
TAGGTCGTTCGCTCCAAGCTGGGCTGTGTGCACGAACCCCCCGTTCAGCCCGACCGCTGCGCCTTATCCG
GTAACTATCGTCTTGAGTCCAACCCGGTAAGACACGACTTATCGCCACTGGCAGCAGCCACTGGTAACAGG
ATTAGCAGAGCGAGGTATGTAGGCGGTGCTACAGAGTTCTTGAAGTGGTGGCCTAACTACGGCTACACTAG
AAGAACAGTATTTGGTATCTGCGCTCTGCTGAAGCCAGTTACCTTCGGAAAAAGAGTTGGTAGCTCTTGATC
CGGCAAACAAACCACCGCTGGTAGCGGTGGTTTTTTTGTTTGCAAGCAGCAGATTACGCGCAGAAAAAAAG
GATCTCAAGAAGATCCTTTGATCTTTTCTACGGGGTCTGACGCTCAGTGGAACGAAAACTCACGTTAAGGG
ATTTTGGTCATGAGATTATCAAAAAGGATCTTCACCTAGATCCTTTTAAATTAAAAATGAAGTTTTAAATCAATC
TAAAGTATATATGAGTAAACTTGGTCTGACAGTTACCAATGCTTAATCAGTGAGGCACCTATCTCAGCGATCT
GTCTATTTCGTTCATCCATAGTTGCCTGACTCCCCGTCGTGTAGATAACTACGATACGGGAGGGCTTACCAT
CTGGCCCCAGTGCTGCAATGATACCGCGAGACCCACGCTCACCGGCTCCAGATTTATCAGCAATAAACCAG
CCAGCCGGAAGGGCCGAGCGCAGAAGTGGTCCTGCAACTTTATCCGCCTCCATCCAGTCTATTAATTGTTG
CCGGGAAGCTAGAGTAAGTAGTTCGCCAGTTAATAGTTTGCGCAACGTTGTTGCCATTGCTACAGGCATCG
TGGTGTCACGCTCGTCGTTTGGTATGGCTTCATTCAGCTCCGGTTCCCAACGATCAAGGCGAGTTACATGA
TCCCCCATGTTGTGCAAAAAAGCGGTTAGCTCCTTCGGTCCTCCGATCGTTGTCAGAAGTAAGTTGGCCGC
AGTGTTATCACTCATGGTTATGGCAGCACTGCATAATTCTCTTACTGTCATGCCATCCGTAAGATGCTTTTCT

GTGACTGGTGAGTACTCAACCAAGTCATTCTGAGAATAGTGTATGCGGCGACCGAGTTGCTCTTGCCCGGC
GTCAATACGGGATAATACCGCGCCACATAGCAGAACTTTAAAAGTGCTCATCATTGGAAAACGTTCTTCGGG
GCGAAAACTCTCAAGGATCTTACCGCTGTTGAGATCCAGTTCGATGTAACCCACTCGTGCACCCAACTGAT
CTTCAGCATCTTTTACTTTCACCAGCGTTTCTGGGTGAGCAAAAACAGGAAGGCAAAATGCCGCAAAAAAG
GGAATAAGGGCGACACGGAAATGTTGAATACTCATACTCTTCCTTTTTCAATATTATTGAAGCATTTATCAGGG
TTATTGTCTCATGAGCGGATACATATTTGAATGTATTTAGAAAAATAAACAAATAGGGGTTCCGCGCACATTTC
CCCGAAAAGTGCCACCTGACGTCGACGGATCGGGAGATCTCCCGATCCCCTATGGTGCACTCTCAGTACA
ATCTGCTCTGATGCCGCATAGTTAAGCCAGTATCTGCTCCCTGCTTGTGTGTTGGAGGTCGCTGAGTAGTG
CGCGAGCAAAATTTAAGCTACAACAAGGCAAGGCTTGACCGACAATTGCATGAAGAATCTGCTTAGGGTTA
GGCGTTTTGCGCTGCTTCGCGATGTACGGGCCAGATATACGCGTTGACATTGATTATTGACTAGTTATTAATA
GTAATCAATTACGGGGTCATTAGTTCATAGCCCATATATGGAGTTCCGCGTTACATAACTTACGGTAAATGGC
CCGCCTGGCTGACCGCCCAACGACCCCCGCCCATTGACGTCAATAATGACGTATGTTCCCATAGTAACGCC
AATAGGGACTTTCCATTGACGTCAATGGGTGGAGTATTTACGGTAAACTGCCCACTTGGCAGTACATCAAGT
GTATCATATGCCAAGTACGCCCCCTATTGACGTCAATGACGGTAAATGGCCCGCCTGGCATTATGCCCAGTA
CATGACCTTATGGGACTTTCCTACTTGGCAGTACATCTACGTATTAGTCATCGCTATTACCATGGTGATGCGG
TTTTGGCAGTACATCAATGGGCGTGGATAGCGGTTTGACTCACGGGGATTTCCAAGTCTCCACCCCATTGA
CGTCAATGGGAGTTTGTTTTGGCACCAAAATCAACGGGACTTTCCAAAATGTCGTAACAACTCCGCCCCATT
GACGCAAATGGGCGGTAGGCGTGTACGGTGGGAGGTCTATATAAGCAGAGCTCTCTGGCTAACTAGAGAA
CCCACTGCTTACTGGCTTATCGAAATTAATACGACTCACTATAGGGAGACCCAAGCTGGCTAGTTAAGCTGA
GCATCAACAAGTTTGTACAAAAAAGCAGGCTCCGAATTCGCCCTTGCCGCCATGGATGAGGAGAAGTCCAA
GATGCTGGCCCGCCTGCTGAAGAGCTCACACCCTGAGGACCTCCGGGCTGCCAATAAGCTCATCAAAGAG
ATGGTGCAGGAGGACCAGAAGCGGATGGAAAAGATCTCCAAGCGGGTGAATGCCATCGAGGAGGTCAAC
AACAATGTGAAGTTGCTGACAGAGATGGTGATGAGCCACAGCCAGGGTGCTGCATCCAGCAGCAGTGAGG
ACCTCATGAAGGAACTGTACCAGCGCTGTGAGCGCATGCGACCCACACTCTTCCGACTGGCCAGTGACAC
GGAAGACAATGATGAGGCCTTAGCTGAGATCCTGCAGGCTAATGACAATCTCACCCAGGTGATCAACCTGT
ACAAGCAGCTGGTCCGGAAGGGCGAATTCGACCCAGCTTTCTTGTACAAAGTGGTTGATGCTGTTAACATG
GTGAGCAAGGGCGAGGAGCTGTTCACCGGGGTGGTGCCCATCCTGGTCGAGCTGGACGGCGACGTAAA
CGGCCACAAGTTCAGCGTGTCCGGCGAGGGCGAGGGCGATGCCACCTACGGCAAGCTGACCCTGAAGTT
CATCTGCACCACCGGCAAGCTGCCCGTGCCCTGGCCCACCCTCGTGACCACCTTCACCTACGGCGTGCA
GTGCTTCGCCCGCTACCCCGACCACATGAAGCAGCACGACTTCTTCAAGTCCGCCATGCCCGAAGGCTAC
GTCCAGGAGCGCACCATCTTCTTCAAGGACGACGGCAACTACAAGACCCGCGCCGAGGTGAAGTTCGAG
GGCGACACCCTGGTGAACCGCATCGAGCTGAAGGGCATCGACTTCAAGGAGGACGGCAACATCCTGGGG
CACAAGCTGGAGTACAACTACAACAGCCACAAGGTCTATATCACCGCCGACAAGCAGAAGAACGGCATCAA
GGTGAACTTCAAGACCCGCCACAACATCGAGGACGGCAGCGTGCAGCTCGCCGACCACTACCAGCAGAA
CACCCCCATCGGCGACGGCCCCGTGCTGCTGCCCGACAACCACTACCTGAGCACCCAGTCCGCCCTGAG
CAAAGACCCCAACGAGAAGCGCGATCACATGGTCCTGCTGGAGTTCGTGACCGCCGCCGGGATCACTCT
CGGCATGGACGAGCTGTACAAGTAATGATAAGTTTAAACGGGGGAGGCTAACTGAAACACGGAAGGAGAC
AATACCGGAAGGAACCCGCGCTATGACGGCAATAAAAAGACAGAATAAAACGCACGGGTGTTGGGTCGTTT
GTTCATAAACGCGGGGTTCGGTCCCAGGGCTGGCACTCTGTCGATACCCCACCGAGACCCCATTGGGGCC
AATACGCCCGCGTTTCTTCCTTTTCCCCACCCCACCCCCCAAGTTCGGGTGAAGGCCCAGGGCTCGCAGC
CAACGTCGGGGCGGCAGGCCCTGCCATAGCAGATCTGCGCAGCTGGGGCTCTAGGGGGTATCCCCACGC
GCCCTGTAGCGGCGCATTAAGCGCGGCGGGTGTGGTGGTTACGCGCAGCGTGACCGCTACACTTGCCAG
CGCCCTAGCGCCCGCTCCTTTCGCTTTCTTCCCTTCCTTTCTCGCCACGTTCGCCGGCTTTCCCCGTCAAG
CTCTAAATCGGGGGCTCCCTTTAGGGTTCCGATTTAGTGCTTTACGGCACCTCGACCCCAAAAAACTTGATT
AGGGTGATGGTTCACGTAGTGGGCCATCGCCCTGATAGACGGTTTTTCGCCCTTTGACGTTGGAGTCCAC
GTTCTTTAATAGTGGACTCTTGTTCCAAACTGGAACAACACTCAACCCTATCTCGGTCTATTCTTTTGATTTAT
AAGGGATTTTGCCGATTTCGGCCTATTGGTTAAAAAATGAGCTGATTTAACAAAAATTTAACGCGAATTAATTC
TGTGGAATGTGTGTCAGTTAGGGTGTGGAAAGTCCCCAGGCTCCCCAGCAGGCAGAAGTATGCAAAGCAT

GCATCTCAATTAGTCAGCAACCAGGTGTGGAAAGTCCCCAGGCTCCCCAGCAGGCAGAAGTATGCAAAGC
ATGCATCTCAATTAGTCAGCAACCATAGTCCCGCCCCTAACTCCGCCCATCCCGCCCCTAACTCCGCCCAG
TTCCGCCCATTCTCCGCCCCATGGCTGACTAATTTTTTTTATTTATGCAGAGGCCGAGGCCGCCTCTGCCTC
TGAGCTATTCCAGAAGTAGTGAGGAGGCTTTTTTGGAGGCCTAGGCTTTTGCAAAAAGCTCCCGGGAGCTT
GTATATCCATTTTCGGATCTGATCAGCACGTGTTGACAATTAATCATCGGCATAGTATATCGGCATAGTATAATA
CGACAAGGTGAGGAACTAAACCATGGCCAAGCCTTTGTCTCAAGAAGAATCCACCCTCATTGAAAGAGCAA
CGGCTACAATCAACAGCATCCCCATCTCTGAAGACTACAGCGTCGCCAGCGCAGCTCTCTCTAGCGACGG
CCGCATCTTCACTGGTGTCAATGTATATCATTTTACTGGGGGACCTTGTGCAGAACTCGTGGTGCTGGGCA
CTGCTGCTGCTGCGGCAGCTGGCAACCTGACTTGTATCGTCGCGATCGGAAATGAGAACAGGGGCATCTT
GAGCCCCTGCGGACGGTGCCGACAGGTGCTTCTCGATCTGCATCCTGGGATCAAAGCCATAGTGAAGGAC
AGTGATGGACAGCCGACGGCAGTTGGGATT

Seq_mGGA1-Hinge_GFP

AGGAGCAGGACTGACACGTGCTACGAGATTTCGATTCCACCGCCGCCTTCTATGAAAGGTTGGGCTTCGG
AATCGTTTTCCGGGACGCCGGCTGGATGATCCTCCAGCGCGGGGATCTCATGCTGGAGTTCTTCGCCCAC
CCCAACTTGTTTATTGCAGCTTATAATGGTTACAAATAAAGCAATAGCATCACAAATTTCACAAATAAAGCATTT
TTTTCACTGCATTCTAGTTGTGGTTTGTCCAAACTCATCAATGTATCTTATCATGTCTGTATACCGTCGACCTC
TAGCTAGAGCTTGGCGTAATCATGGTCATAGCTGTTTCCTGTGTGAAATTGTTATCCGCTCACAATTCCACAC
AACATACGAGCCGGAAGCATAAAGTGTAAAGCCTGGGGTGCCTAATGAGTGAGCTAACTCACATTAATTGCG
TTGCGCTCACTGCCCGCTTTCCAGTCGGGAAACCTGTCGTGCCAGCTGCATTAATGAATCGGCCAACGCG
CGGGGAGAGGCGGTTTGCGTATTGGGCGCTCTTCCGCTTCCTCGCTCACTGACTCGCTGCGCTCGGTCG
TTCGGCTGCGGCGAGCGGTATCAGCTCACTCAAAGGCGGTAATACGGTTATCCACAGAATCAGGGGATAAC
GCAGGAAAGAACATGTGAGCAAAAGGCCAGCAAAAGGCCAGGAACCGTAAAAAGGCCGCGTTGCTGGCG
TTTTTCCATAGGCTCCGCCCCCCTGACGAGCATCACAAAAATCGACGCTCAAGTCAGAGGTGGCGAAACC
CGACAGGACTATAAAGATACCAGGCGTTTCCCCCTGGAAGCTCCCTCGTGCGCTCTCCTGTTCCGACCCT
GCCGCTTACCGGATACCTGTCCGCCTTTCTCCCTTCGGGAAGCGTGGCGCTTTCTCATAGCTCACGCTGTA
GGTATCTCAGTTCGGTGTAGGTCGTTCGCTCCAAGCTGGGCTGTGTGCACGAACCCCCCGTTCAGCCCGA
CCGCTGCGCCTTATCCGGTAACTATCGTCTTGAGTCCAACCCGGTAAGACACGACTTATCGCCACTGGCAG
CAGCCACTGGTAACAGGATTAGCAGAGCGAGGTATGTAGGCGGTGCTACAGAGTTCTTGAAGTGGTGGCC
TAACTACGGCTACACTAGAAGAACAGTATTTGGTATCTGCGCTCTGCTGAAGCCAGTTACCTTCGGAAAAAG
AGTTGGTAGCTCTTGATCCGGCAAACAAACCACCGCTGGTAGCGGTGGTTTTTTTGTTTGCAAGCAGCAGA
TTACGCGCAGAAAAAAAGGATCTCAAGAAGATCCTTTGATCTTTTCTACGGGGTCTGACGCTCAGTGGAAC
GAAAACTCACGTTAAGGGATTTTGGTCATGAGATTATCAAAAAGGATCTTCACCTAGATCCTTTTAAATTAAAA
ATGAAGTTTTAAATCAATCTAAAGTATATATGAGTAAACTTGGTCTGACAGTTACCAATGCTTAATCAGTGAGG
CACCTATCTCAGCGATCTGTCTATTTCGTTCATCCATAGTTGCCTGACTCCCCGTCGTGTAGATAACTACGAT
ACGGGAGGGCTTACCATCTGGCCCCAGTGCTGCAATGATACCGCGAGACCCACGCTCACCGGCTCCAGAT
TTATCAGCAATAAACCAGCCAGCCGGAAGGGCCGAGCGCAGAAGTGGTCCTGCAACTTTATCCGCCTCCAT
CCAGTCTATTAATTGTTGCCGGGAAGCTAGAGTAAGTAGTTCGCCAGTTAATAGTTTGCGCAACGTTGTTGC
CATTGCTACAGGCATCGTGGTGTCACGCTCGTCGTTTGGTATGGCTTCATTCAGCTCCGGTTCCCAACGAT
CAAGGCGAGTTACATGATCCCCCATGTTGTGCAAAAAAGCGGTTAGCTCCTTCGGTCCTCCGATCGTTGTC
AGAAGTAAGTTGGCCGCAGTGTTATCACTCATGGTTATGGCAGCACTGCATAATTCTCTTACTGTCATGCCAT
CCGTAAGATGCTTTTCTGTGACTGGTGAGTACTCAACCAAGTCATTCTGAGAATAGTGTATGCGGCGACCGA
GTTGCTCTTGCCCGGCGTCAATACGGGATAATACCGCGCCACATAGCAGAACTTTAAAAGTGCTCATCATTG
GAAAACGTTCTTCGGGGCGAAAACTCTCAAGGATCTTACCGCTGTTGAGATCCAGTTCGATGTAACCCACT
CGTGCACCCAACTGATCTTCAGCATCTTTTACTTTCACCAGCGTTTCTGGGTGAGCAAAAACAGGAAGGCA
AAATGCCGCAAAAAAGGGAATAAGGGCGACACGGAAATGTTGAATACTCATACTCTTCCTTTTTCAATATTAT

TGAAGCATTTATCAGGGTTATTGTCTCATGAGCGGATACATATTTGAATGTATTTAGAAAAATAAACAAATAGG
GGTTCCGCGCACATTTCCCCGAAAAGTGCCACCTGACGTCGACGGATCGGGAGATCTCCCGATCCCCTAT
GGTGCACTCTCAGTACAATCTGCTCTGATGCCGCATAGTTAAGCCAGTATCTGCTCCCTGCTTGTGTGTTGG
AGGTCGCTGAGTAGTGCGCGAGCAAAATTTAAGCTACAACAAGGCAAGGCTTGACCGACAATTGCATGAAG
AATCTGCTTAGGGTTAGGCGTTTTGCGCTGCTTCGCGATGTACGGGCCAGATATACGCGTTGACATTGATTA
TTGACTAGTTATTAATAGTAATCAATTACGGGGTCATTAGTTCATAGCCCATATATGGAGTTCCGCGTTACATAA
CTTACGGTAAATGGCCCGCCTGGCTGACCGCCCAACGACCCCCGCCCATTGACGTCAATAATGACGTATGT
TCCCATAGTAACGCCAATAGGGACTTTCCATTGACGTCAATGGGTGGAGTATTTACGGTAAACTGCCCACTT
GGCAGTACATCAAGTGTATCATATGCCAAGTACGCCCCCTATTGACGTCAATGACGGTAAATGGCCCGCCTG
GCATTATGCCCAGTACATGACCTTATGGGACTTTCCTACTTGGCAGTACATCTACGTATTAGTCATCGCTATTA
CCATGGTGATGCGGTTTTGGCAGTACATCAATGGGCGTGGATAGCGGTTTGACTCACGGGGATTTCCAAGT
CTCCACCCCATTGACGTCAATGGGAGTTTGTTTTGGCACCAAAATCAACGGGACTTTCCAAAATGTCGTAAC
AACTCCGCCCCATTGACGCAAATGGGCGGTAGGCGTGTACGGTGGGAGGTCTATATAAGCAGAGCTCTCT
GGCTAACTAGAGAACCCACTGCTTACTGGCTTATCGAAATTAATACGACTCACTATAGGGAGACCCAAGCTG
GCTAGTTAAGCTGAGCATCAACAAGTTTGTACAAAAAAGCAGGCTCCGAATTCGCCCTTGCCGCCATGGGC
GAGGAGGTCAACGGTGATGCCACAGCCAGCTCCATTCCTGGAAGCACGTCAGCCCTGCTGGACCTCTCA
GGCCTGGACCTCCCTCCCCCGGGCACCACCCAGCCAGCCACGCCCACCCGCCCTGGCAACCAGAGCAG
TCCTGAGCAGCTCAGTGCCTCGGTGTCCCTGCTTGATGACGAGCTCATGTCTCTGGGCCTAAGTGACCCG
ACACCACCTTCAGGCACCAGCTCAGATAGTGTGGGGTGGGACAACTTCCAGTCATCAGATGGCACTGAAT
CCTCAGTCCCTCCTCCAGCCCAGGCCCCCAGTATGGACTGCCGACCCCCAGCCCAGGCCCCTCCACCAA
CGAGCAGTGGCCTGGACGACCTGGACCTCTTGGGGAAAACCCTTATGCAGCAGGCTCTGCCTCCGGAAG
CCCAGCAAGTGCGGTGGGAGAAGCAGCAGCCAGCCCCCCGGCTCACCCTCCGTGACCTGCAGAGTAAGA
GCAGCTCGCCCAGCCCAGGAGCCGCCAGCCTCCTCCACACCACGTCCCCAGAGCCCCCTGGCCTCCA
CCTCAGGCCACACCCACTGAGTTCTCCCTAACCAGCATCACTGTGCCCCTGGAGTCTATCAAACCCAAGG
GCGAATTCGACCCAGCTTTCTTGTACAAAGTGGTTGATGCTGTTAACATGGTGAGCAAGGGCGAGGAGCTG
TTCACCGGGGTGGTGCCCATCCTGGTCGAGCTGGACGGCGACGTAAACGGCCACAAGTTCAGCGTGTCC
GGCGAGGGCGAGGGCGATGCCACCTACGGCAAGCTGACCCTGAAGTTCATCTGCACCACCGGCAAGCTG
CCCGTGCCCTGGCCCACCCTCGTGACCACCTTCACCTACGGCGTGCAGTGCTTCGCCCGCTACCCCGAC
CACATGAAGCAGCACGACTTCTTCAAGTCCGCCATGCCCGAAGGCTACGTCCAGGAGCGCACCATCTTCT
TCAAGGACGACGGCAACTACAAGACCCGCGCCGAGGTGAAGTTCGAGGGCGACACCCTGGTGAACCGCA
TCGAGCTGAAGGGCATCGACTTCAAGGAGGACGGCAACATCCTGGGGCACAAGCTGGAGTACAACTACAA
CAGCCACAAGGTCTATATCACCGCCGACAAGCAGAAGAACGGCATCAAGGTGAACTTCAAGACCCGCCAC
AACATCGAGGACGGCAGCGTGCAGCTCGCCGACCACTACCAGCAGAACACCCCCATCGGCGACGGCCCC
GTGCTGCTGCCCGACAACCACTACCTGAGCACCCAGTCCGCCCTGAGCAAAGACCCCAACGAGAAGCGC
GATCACATGGTCCTGCTGGAGTTCGTGACCGCCGCCGGGATCACTCTCGGCATGGACGAGCTGTACAAGT
AATGATAAGTTTAAACGGGGGAGGCTAACTGAAACACGGAAGGAGACAATACCGGAAGGAACCCGCGCTAT
GACGGCAATAAAAAGACAGAATAAAACGCACGGGTGTTGGGTCGTTTGTTCATAAACGCGGGGTTCGGTCC
CAGGGCTGGCACTCTGTCGATACCCCACCGAGACCCCATTGGGGCCAATACGCCCGCGTTTCTTCCTTTT
CCCCACCCCACCCCCCAAGTTCGGGTGAAGGCCCAGGGCTCGCAGCCAACGTCGGGGCGGCAGGCCCT
GCCATAGCAGATCTGCGCAGCTGGGGCTCTAGGGGGTATCCCCACGCGCCCTGTAGCGGCGCATTAAGCG
CGGCGGGTGTGGTGGTTACGCGCAGCGTGACCGCTACACTTGCCAGCGCCCTAGCGCCCGCTCCTTTCG
CTTTCTTCCCTTCCTTTCTCGCCACGTTCGCCGGCTTTCCCCGTCAAGCTCTAAATCGGGGGCTCCCTTTA
GGGTTCCGATTTAGTGCTTTACGGCACCTCGACCCCAAAAAACTTGATTAGGGTGATGGTTCACGTAGTGG
GCCATCGCCCTGATAGACGGTTTTTCGCCCTTTGACGTTGGAGTCCACGTTCTTTAATAGTGGACTCTTGTT
CCAAACTGGAACAACACTCAACCCTATCTCGGTCTATTCTTTTGATTTATAAGGGATTTTGCCGATTTCGGCC
TATTGGTTAAAAAATGAGCTGATTTAACAAAAATTTAACGCGAATTAATTCTGTGGAATGTGTGTCAGTTAGGG
TGTGGAAAGTCCCCAGGCTCCCCAGCAGGCAGAAGTATGCAAAGCATGCATCTCAATTAGTCAGCAACCAG
GTGTGGAAAGTCCCCAGGCTCCCCAGCAGGCAGAAGTATGCAAAGCATGCATCTCAATTAGTCAGCAACCA

TAGTCCCGCCCCTAACTCCGCCCATCCCGCCCCTAACTCCGCCCAGTTCCGCCCATTCTCCGCCCCATGG
CTGACTAATTTTTTTTATTTATGCAGAGGCCGAGGCCGCCTCTGCCTCTGAGCTATTCCAGAAGTAGTGAGG
AGGCTTTTTTGGAGGCCTAGGCTTTTGCAAAAAGCTCCCGGGAGCTTGTATATCCATTTTCGGATCTGATCA
GCACGTGTTGACAATTAATCATCGGCATAGTATATCGGCATAGTATAATACGACAAGGTGAGGAACTAAACCA
TGGCCAAGCCTTTGTCTCAAGAAGAATCCACCCTCATTGAAAGAGCAACGGCTACAATCAACAGCATCCCC
ATCTCTGAAGACTACAGCGTCGCCAGCGCAGCTCTCTCTAGCGACGGCCGCATCTTCACTGGTGTCAATGT
ATATCATTTTACTGGGGGACCTTGTGCAGAACTCGTGGTGCTGGGCACTGCTGCTGCTGCGGCAGCTGGC
AACCTGACTTGTATCGTCGCGATCGGAAATGAGAACAGGGGCATCTTGAGCCCCTGCGGACGGTGCCGAC
AGGTGCTTCTCGATCTGCATCCTGGGATCAAAGCCATAGTGAAGGACAGTGATGGACAGCCGACGGCAGT
TGGGATTCGTGAATTGCTGCCCTCTGGTTATGTGTGGGAGGGCTAAGCACTTCGTGGCCG

Seq_mGGA1-GAE_GFP

TTCGTGAATTGCTGCCCTCTGGTTATGTGTGGGAGGGCTAAGCACTTCGTGGCCGAGGAGCAGGACTGAC
ACGTGCTACGAGATTTCGATTCCACCGCCGCCTTCTATGAAAGGTTGGGCTTCGGAATCGTTTTCCGGGAC
GCCGGCTGGATGATCCTCCAGCGCGGGGATCTCATGCTGGAGTTCTTCGCCCACCCCAACTTGTTTATTGC
AGCTTATAATGGTTACAAATAAAGCAATAGCATCACAAATTTCACAAATAAAGCATTTTTTTCACTGCATTCTAG
TTGTGGTTTGTCCAAACTCATCAATGTATCTTATCATGTCTGTATACCGTCGACCTCTAGCTAGAGCTTGGCG
TAATCATGGTCATAGCTGTTTCCTGTGTGAAATTGTTATCCGCTCACAATTCCACACAACATACGAGCCGGAA
GCATAAAGTGTAAAGCCTGGGGTGCCTAATGAGTGAGCTAACTCACATTAATTGCGTTGCGCTCACTGCCC
GCTTTCCAGTCGGGAAACCTGTCGTGCCAGCTGCATTAATGAATCGGCCAACGCGCGGGGAGAGGCGGTT
TGCGTATTGGGCGCTCTTCCGCTTCCTCGCTCACTGACTCGCTGCGCTCGGTCGTTCGGCTGCGGCGAG
CGGTATCAGCTCACTCAAAGGCGGTAATACGGTTATCCACAGAATCAGGGGATAACGCAGGAAAGAACATG
TGAGCAAAAGGCCAGCAAAAGGCCAGGAACCGTAAAAAGGCCGCGTTGCTGGCGTTTTTCCATAGGCTCC
GCCCCCCTGACGAGCATCACAAAAATCGACGCTCAAGTCAGAGGTGGCGAAACCCGACAGGACTATAAAG
ATACCAGGCGTTTCCCCCTGGAAGCTCCCTCGTGCGCTCTCCTGTTCCGACCCTGCCGCTTACCGGATAC
CTGTCCGCCTTTCTCCCTTCGGGAAGCGTGGCGCTTTCTCATAGCTCACGCTGTAGGTATCTCAGTTCGGT
GTAGGTCGTTCGCTCCAAGCTGGGCTGTGTGCACGAACCCCCCGTTCAGCCCGACCGCTGCGCCTTATCC
GGTAACTATCGTCTTGAGTCCAACCCGGTAAGACACGACTTATCGCCACTGGCAGCAGCCACTGGTAACAG
GATTAGCAGAGCGAGGTATGTAGGCGGTGCTACAGAGTTCTTGAAGTGGTGGCCTAACTACGGCTACACTA
GAAGAACAGTATTTGGTATCTGCGCTCTGCTGAAGCCAGTTACCTTCGGAAAAAGAGTTGGTAGCTCTTGAT
CCGGCAAACAAACCACCGCTGGTAGCGGTGGTTTTTTTGTTTGCAAGCAGCAGATTACGCGCAGAAAAAAA
GGATCTCAAGAAGATCCTTTGATCTTTTCTACGGGGTCTGACGCTCAGTGGAACGAAAACTCACGTTAAGG
GATTTTGGTCATGAGATTATCAAAAAGGATCTTCACCTAGATCCTTTTAAATTAAAAATGAAGTTTTAAATCAAT
CTAAAGTATATATGAGTAAACTTGGTCTGACAGTTACCAATGCTTAATCAGTGAGGCACCTATCTCAGCGATC
TGTCTATTTCGTTCATCCATAGTTGCCTGACTCCCCGTCGTGTAGATAACTACGATACGGGAGGGCTTACCAT
CTGGCCCCAGTGCTGCAATGATACCGCGAGACCCACGCTCACCGGCTCCAGATTTATCAGCAATAAACCAG
CCAGCCGGAAGGGCCGAGCGCAGAAGTGGTCCTGCAACTTTATCCGCCTCCATCCAGTCTATTAATTGTTG
CCGGGAAGCTAGAGTAAGTAGTTCGCCAGTTAATAGTTTGCGCAACGTTGTTGCCATTGCTACAGGCATCG
TGGTGTCACGCTCGTCGTTTGGTATGGCTTCATTCAGCTCCGGTTCCCAACGATCAAGGCGAGTTACATGA
TCCCCCATGTTGTGCAAAAAAGCGGTTAGCTCCTTCGGTCCTCCGATCGTTGTCAGAAGTAAGTTGGCCGC
AGTGTTATCACTCATGGTTATGGCAGCACTGCATAATTCTCTTACTGTCATGCCATCCGTAAGATGCTTTTCT
GTGACTGGTGAGTACTCAACCAAGTCATTCTGAGAATAGTGTATGCGGCGACCGAGTTGCTCTTGCCCGGC
GTCAATACGGGATAATACCGCGCCACATAGCAGAACTTTAAAAGTGCTCATCATTGGAAAACGTTCTTCGGG
GCGAAAACTCTCAAGGATCTTACCGCTGTTGAGATCCAGTTCGATGTAACCCACTCGTGCACCCAACTGAT
CTTCAGCATCTTTTACTTTCACCAGCGTTTCTGGGTGAGCAAAAACAGGAAGGCAAAATGCCGCAAAAAAG
GGAATAAGGGCGACACGGAAATGTTGAATACTCATACTCTTCCTTTTTCAATATTATTGAAGCATTTATCAGGG

TTATTGTCTCATGAGCGGATACATATTTGAATGTATTTAGAAAAATAAACAAATAGGGGTTCCGCGCACATTTC
CCCGAAAAGTGCCACCTGACGTCGACGGATCGGGAGATCTCCCGATCCCCTATGGTGCACTCTCAGTACA
ATCTGCTCTGATGCCGCATAGTTAAGCCAGTATCTGCTCCCTGCTTGTGTGTTGGAGGTCGCTGAGTAGTG
CGCGAGCAAAATTTAAGCTACAACAAGGCAAGGCTTGACCGACAATTGCATGAAGAATCTGCTTAGGGTTA
GGCGTTTTGCGCTGCTTCGCGATGTACGGGCCAGATATACGCGTTGACATTGATTATTGACTAGTTATTAATA
GTAATCAATTACGGGGTCATTAGTTCATAGCCCATATATGGAGTTCCGCGTTACATAACTTACGGTAAATGGC
CCGCCTGGCTGACCGCCCAACGACCCCCGCCCATTGACGTCAATAATGACGTATGTTCCCATAGTAACGCC
AATAGGGACTTTCCATTGACGTCAATGGGTGGAGTATTTACGGTAAACTGCCCACTTGGCAGTACATCAAGT
GTATCATATGCCAAGTACGCCCCCTATTGACGTCAATGACGGTAAATGGCCCGCCTGGCATTATGCCCAGTA
CATGACCTTATGGGACTTTCCTACTTGGCAGTACATCTACGTATTAGTCATCGCTATTACCATGGTGATGCGG
TTTTGGCAGTACATCAATGGGCGTGGATAGCGGTTTGACTCACGGGGATTTCCAAGTCTCCACCCCATTGA
CGTCAATGGGAGTTTGTTTTGGCACCAAAATCAACGGGACTTTCCAAAATGTCGTAACAACTCCGCCCCATT
GACGCAAATGGGCGGTAGGCGTGTACGGTGGGAGGTCTATATAAGCAGAGCTCTCTGGCTAACTAGAGAA
CCCACTGCTTACTGGCTTATCGAAATTAATACGACTCACTATAGGGAGACCCAAGCTGGCTAGTTAAGCTGA
GCATCAACAAGTTTGTACAAAAAAGCAGGCTCCGAATTCGCCCTTGCCGCCATGGCCAGCAGCATCTTGCC
AGTGACCGTTTATGACCAGCATGGCTTCCGTGTCCTCTTCCATTTTGCTCGGGACCCACTGCCAGGGCGCT
CCGATGTGCTGGTGGTGGTGGTCTCTATGCTGAGCACGGCGCCCCAGCCCATCCGGAACATCGTTTTCCA
GTCAGCCGTCCCCAAGGTCATGAAGGTGAGGCTGCAGCCACCTTCCGGCACAGAGCTGCCAGCGTTCAA
CCCCATCGTCCACCCCTCAGCCATCACCCAGGTCCTGCTCCTTGCTAACCCCCAGAAGGAGAAGGTTCGC
CTCCGCTACAAGCTCATCTTCACTATGGGCGACCAGACCTACAATGAGATGGGAGATGTGGATCAGTTCCC
CAAGGGCGAATTCGACCCAGCTTTCTTGTACAAAGTGGTTGATGCTGTTAACATGGTGAGCAAGGGCGAG
GAGCTGTTCACCGGGGTGGTGCCCATCCTGGTCGAGCTGGACGGCGACGTAAACGGCCACAAGTTCAGC
GTGTCCGGCGAGGGCGAGGGCGATGCCACCTACGGCAAGCTGACCCTGAAGTTCATCTGCACCACCGGC
AAGCTGCCCGTGCCCTGGCCCACCCTCGTGACCACCTTCACCTACGGCGTGCAGTGCTTCGCCCGCTAC
CCCGACCACATGAAGCAGCACGACTTCTTCAAGTCCGCCATGCCCGAAGGCTACGTCCAGGAGCGCACCA
TCTTCTTCAAGGACGACGGCAACTACAAGACCCGCGCCGAGGTGAAGTTCGAGGGCGACACCCTGGTGA
ACCGCATCGAGCTGAAGGGCATCGACTTCAAGGAGGACGGCAACATCCTGGGGCACAAGCTGGAGTACA
ACTACAACAGCCACAAGGTCTATATCACCGCCGACAAGCAGAAGAACGGCATCAAGGTGAACTTCAAGACC
CGCCACAACATCGAGGACGGCAGCGTGCAGCTCGCCGACCACTACCAGCAGAACACCCCCATCGGCGAC
GGCCCCGTGCTGCTGCCCGACAACCACTACCTGAGCACCCAGTCCGCCCTGAGCAAAGACCCCAACGAG
AAGCGCGATCACATGGTCCTGCTGGAGTTCGTGACCGCCGCCGGGATCACTCTCGGCATGGACGAGCTG
TACAAGTAATGATAAGTTTAAACGGGGGAGGCTAACTGAAACACGGAAGGAGACAATACCGGAAGGAACCC
GCGCTATGACGGCAATAAAAAGACAGAATAAAACGCACGGGTGTTGGGTCGTTTGTTCATAAACGCGGGGT
TCGGTCCCAGGGCTGGCACTCTGTCGATACCCCACCGAGACCCCATTGGGGCCAATACGCCCGCGTTTCT
TCCTTTTCCCCACCCCACCCCCCAAGTTCGGGTGAAGGCCCAGGGCTCGCAGCCAACGTCGGGGCGGCA
GGCCCTGCCATAGCAGATCTGCGCAGCTGGGGCTCTAGGGGGTATCCCCACGCGCCCTGTAGCGGCGCA
TTAAGCGCGGCGGGTGTGGTGGTTACGCGCAGCGTGACCGCTACACTTGCCAGCGCCCTAGCGCCCGCT
CCTTTCGCTTTCTTCCCTTCCTTTCTCGCCACGTTCGCCGGCTTTCCCCGTCAAGCTCTAAATCGGGGGCT
CCCTTTAGGGTTCCGATTTAGTGCTTTACGGCACCTCGACCCCAAAAAACTTGATTAGGGTGATGGTTCACG
TAGTGGGCCATCGCCCTGATAGACGGTTTTTCGCCCTTTGACGTTGGAGTCCACGTTCTTTAATAGTGGACT
CTTGTTCCAAACTGGAACAACACTCAACCCTATCTCGGTCTATTCTTTTGATTTATAAGGGATTTTGCCGATTT
CGGCCTATTGGTTAAAAAATGAGCTGATTTAACAAAAATTTAACGCGAATTAATTCTGTGGAATGTGTGTCAG
TTAGGGTGTGGAAAGTCCCCAGGCTCCCCAGCAGGCAGAAGTATGCAAAGCATGCATCTCAATTAGTCAGC
AACCAGGTGTGGAAAGTCCCCAGGCTCCCCAGCAGGCAGAAGTATGCAAAGCATGCATCTCAATTAGTCA
GCAACCATAGTCCCGCCCCTAACTCCGCCCATCCCGCCCCTAACTCCGCCCAGTTCCGCCCATTCTCCGC
CCCATGGCTGACTAATTTTTTTTATTTATGCAGAGGCCGAGGCCGCCTCTGCCTCTGAGCTATTCCAGAAGT
AGTGAGGAGGCTTTTTTGGAGGCCTAGGCTTTTGCAAAAAGCTCCCGGGAGCTTGTATATCCATTTTCGGAT
CTGATCAGCACGTGTTGACAATTAATCATCGGCATAGTATATCGGCATAGTATAATACGACAAGGTGAGGAAC

TAAACCATGGCCAAGCCTTTGTCTCAAGAAGAATCCACCCTCATTGAAAGAGCAACGGCTACAATCAACAG
CATCCCCATCTCTGAAGACTACAGCGTCGCCAGCGCAGCTCTCTCTAGCGACGGCCGCATCTTCACTGGT
GTCAATGTATATCATTTTACTGGGGGACCTTGTGCAGAACTCGTGGTGCTGGGCACTGCTGCTGCTGCGGC
AGCTGGCAACCTGACTTGTATCGTCGCGATCGGAAATGAGAACAGGGGCATCTTGAGCCCCTGCGGACGG
TGCCGACAGGTGCTTCTCGATCTGCATCCTGGGATCAAAGCCATAGTGAAGGACAGTGATGGACAGCCGA
CGGCAGTTGGGA

Seq_mGGA1-delGAE-GFP

ACAGCGTCGCCAGCGCAGCTCTCTCTAGCGACGGCCGCATCTTCACTGGTGTCAATGTATATCATTTTACTG
GGGGACCTTGTGCAGAACTCGTGGTGCTGGGCACTGCTGCTGCTGCGGCAGCTGGCAACCTGACTTGTA
TCGTCGCGATCGGAAATGAGAACAGGGGCATCTTGAGCCCCTGCGGACGGTGCCGACAGGTGCTTCTCG
ATCTGCATCCTGGGATCAAAGCCATAGTGAAGGACAGTGATGGACAGCCGACGGCAGTTGGGATTCGTGA
ATTGCTGCCCTCTGGTTATGTGTGGGAGGGCTAAGCACTTCGTGGCCGAGGAGCAGGACTGACACGTGCT
ACGAGATTTCGATTCCACCGCCGCCTTCTATGAAAGGTTGGGCTTCGGAATCGTTTTCCGGGACGCCGGCT
GGATGATCCTCCAGCGCGGGGATCTCATGCTGGAGTTCTTCGCCCACCCCAACTTGTTTATTGCAGCTTATA
ATGGTTACAAATAAAGCAATAGCATCACAAATTTCACAAATAAAGCATTTTTTTCACTGCATTCTAGTTGTGGT
TTGTCCAAACTCATCAATGTATCTTATCATGTCTGTATACCGTCGACCTCTAGCTAGAGCTTGGCGTAATCAT
GGTCATAGCTGTTTCCTGTGTGAAATTGTTATCCGCTCACAATTCCACACAACATACGAGCCGGAAGCATAA
AGTGTAAAGCCTGGGGTGCCTAATGAGTGAGCTAACTCACATTAATTGCGTTGCGCTCACTGCCCGCTTTC
CAGTCGGGAAACCTGTCGTGCCAGCTGCATTAATGAATCGGCCAACGCGCGGGGAGAGGCGGTTTGCGT
ATTGGGCGCTCTTCCGCTTCCTCGCTCACTGACTCGCTGCGCTCGGTCGTTCGGCTGCGGCGAGCGGTAT
CAGCTCACTCAAAGGCGGTAATACGGTTATCCACAGAATCAGGGGATAACGCAGGAAAGAACATGTGAGCA
AAAGGCCAGCAAAAGGCCAGGAACCGTAAAAAGGCCGCGTTGCTGGCGTTTTTCCATAGGCTCCGCCCCC
CTGACGAGCATCACAAAAATCGACGCTCAAGTCAGAGGTGGCGAAACCCGACAGGACTATAAAGATACCAG
GCGTTTCCCCCTGGAAGCTCCCTCGTGCGCTCTCCTGTTCCGACCCTGCCGCTTACCGGATACCTGTCCG
CCTTTCTCCCTTCGGGAAGCGTGGCGCTTTCTCATAGCTCACGCTGTAGGTATCTCAGTTCGGTGTAGGTC
GTTCGCTCCAAGCTGGGCTGTGTGCACGAACCCCCCGTTCAGCCCGACCGCTGCGCCTTATCCGGTAACT
ATCGTCTTGAGTCCAACCCGGTAAGACACGACTTATCGCCACTGGCAGCAGCCACTGGTAACAGGATTAGC
AGAGCGAGGTATGTAGGCGGTGCTACAGAGTTCTTGAAGTGGTGGCCTAACTACGGCTACACTAGAAGAAC
AGTATTTGGTATCTGCGCTCTGCTGAAGCCAGTTACCTTCGGAAAAAGAGTTGGTAGCTCTTGATCCGGCAA
ACAAACCACCGCTGGTAGCGGTGGTTTTTTTGTTTGCAAGCAGCAGATTACGCGCAGAAAAAAAGGATCTC
AAGAAGATCCTTTGATCTTTTCTACGGGGTCTGACGCTCAGTGGAACGAAAACTCACGTTAAGGGATTTTG
GTCATGAGATTATCAAAAAGGATCTTCACCTAGATCCTTTTAAATTAAAAATGAAGTTTTAAATCAATCTAAAGT
ATATATGAGTAAACTTGGTCTGACAGTTACCAATGCTTAATCAGTGAGGCACCTATCTCAGCGATCTGTCTAT
TTCGTTCATCCATAGTTGCCTGACTCCCCGTCGTGTAGATAACTACGATACGGGAGGGCTTACCATCTGGCC
CCAGTGCTGCAATGATACCGCGAGACCCACGCTCACCGGCTCCAGATTTATCAGCAATAAACCAGCCAGCC
GGAAGGGCCGAGCGCAGAAGTGGTCCTGCAACTTTATCCGCCTCCATCCAGTCTATTAATTGTTGCCGGGA
AGCTAGAGTAAGTAGTTCGCCAGTTAATAGTTTGCGCAACGTTGTTGCCATTGCTACAGGCATCGTGGTGTC
ACGCTCGTCGTTTGGTATGGCTTCATTCAGCTCCGGTTCCCAACGATCAAGGCGAGTTACATGATCCCCCA
TGTTGTGCAAAAAAGCGGTTAGCTCCTTCGGTCCTCCGATCGTTGTCAGAAGTAAGTTGGCCGCAGTGTTA
TCACTCATGGTTATGGCAGCACTGCATAATTCTCTTACTGTCATGCCATCCGTAAGATGCTTTTCTGTGACTG
GTGAGTACTCAACCAAGTCATTCTGAGAATAGTGTATGCGGCGACCGAGTTGCTCTTGCCCGGCGTCAATA
CGGGATAATACCGCGCCACATAGCAGAACTTTAAAAGTGCTCATCATTGGAAAACGTTCTTCGGGGCGAAA
ACTCTCAAGGATCTTACCGCTGTTGAGATCCAGTTCGATGTAACCCACTCGTGCACCCAACTGATCTTCAGC
ATCTTTTACTTTCACCAGCGTTTCTGGGTGAGCAAAAACAGGAAGGCAAAATGCCGCAAAAAAGGGAATAA
GGGCGACACGGAAATGTTGAATACTCATACTCTTCCTTTTTCAATATTATTGAAGCATTTATCAGGGTTATTGT

CTCATGAGCGGATACATATTTGAATGTATTTAGAAAAATAAACAAATAGGGGTTCCGCGCACATTTCCCCGAA
AAGTGCCACCTGACGTCGACGGATCGGGAGATCTCCCGATCCCCTATGGTGCACTCTCAGTACAATCTGCT
CTGATGCCGCATAGTTAAGCCAGTATCTGCTCCCTGCTTGTGTGTTGGAGGTCGCTGAGTAGTGCGCGAGC
AAAATTTAAGCTACAACAAGGCAAGGCTTGACCGACAATTGCATGAAGAATCTGCTTAGGGTTAGGCGTTTT
GCGCTGCTTCGCGATGTACGGGCCAGATATACGCGTTGACATTGATTATTGACTAGTTATTAATAGTAATCAAT
TACGGGGTCATTAGTTCATAGCCCATATATGGAGTTCCGCGTTACATAACTTACGGTAAATGGCCCGCCTGG
CTGACCGCCCAACGACCCCCGCCCATTGACGTCAATAATGACGTATGTTCCCATAGTAACGCCAATAGGGA
CTTTCCATTGACGTCAATGGGTGGAGTATTTACGGTAAACTGCCCACTTGGCAGTACATCAAGTGTATCATAT
GCCAAGTACGCCCCCTATTGACGTCAATGACGGTAAATGGCCCGCCTGGCATTATGCCCAGTACATGACCT
TATGGGACTTTCCTACTTGGCAGTACATCTACGTATTAGTCATCGCTATTACCATGGTGATGCGGTTTTGGCA
GTACATCAATGGGCGTGGATAGCGGTTTGACTCACGGGGATTTCCAAGTCTCCACCCCATTGACGTCAATG
GGAGTTTGTTTTGGCACCAAAATCAACGGGACTTTCCAAAATGTCGTAACAACTCCGCCCCATTGACGCAA
ATGGGCGGTAGGCGTGTACGGTGGGAGGTCTATATAAGCAGAGCTCTCTGGCTAACTAGAGAACCCACTGC
TTACTGGCTTATCGAAATTAATACGACTCACTATAGGGAGACCCAAGCTGGCTAGTTAAGCTGAGCATCAACA
AGTTTGTACAAAAAAGCAGGCTCCGAATTCGCCCTTGCCGCCATGGAGCCCGCGATGGAGCCGGAGACTC
TGGAGGCACGAATCAACAGAGCCACAAATCCCCTGAACAAGGAGCTGAACTGGGCCAGCATCAACAGTTT
CTGCGAGCAGCTCAACGAAGACTTTGAGGGGCCTCCACTTGCCACTCGCTTGCTGGCCCACAAGATCCAG
TCCCCACAGGAATGGGAAGCCATCCAGGCCTTGACGGTTCTGGAGACGTGCATGAAGAGCTGCGGCAAG
AGGTTCCATGATGAGGTGGGCAAGTTCCGCTTCCTCAACGAGCTCATCAAGGTTGTGTCTCCCAAGTACTT
GGGCTCCCGGACATCTGAGAAGGTGAAGAGTAAGATCTTGGAGCTGCTGTACAGCTGGACGGTTTGCCTG
CCTGAGGAGGTGAAGATTGCAGAAGCCTACCAGATGCTGAAGAAGCAGGGGATTGTGAAGTCGGACCCCA
AGCTTCCAGAGGATGCCATCTTTCCCCTCCCCCCTCCCCGGCCCAAGAATGTGATCTTTGAAGATGAGGAG
AAGTCCAAGATGCTGGCCCGCCTGCTGAAGAGCTCACACCCTGAGGACCTCCGGGCTGCCAATAAGCTCA
TCAAAGAGATGGTGCAGGAGGACCAGAAGCGGATGGAAAAGATCTCCAAGCGGGTGAATGCCATCGAGGA
GGTCAACAACAATGTGAAGTTGCTGACAGAGATGGTGATGAGCCACAGCCAGGGTGCTGCATCCAGCAGC
AGTGAGGACCTCATGAAGGAACTGTACCAGCGCTGTGAGCGCATGCGACCCACACTCTTCCGACTGGCCA
GTGACACGGAAGACAATGATGAGGCCTTAGCTGAGATCCTGCAGGCTAATGACAATCTCACCCAGGTGATC
AACCTGTACAAGCAGCTGGTCCGGGGCGAGGAGGTCAACGGTGATGCCACAGCCAGCTCCATTCCTGGA
AGCACGTCAGCCCTGCTGGACCTCTCAGGCCTGGACCTCCCTCCCCCGGGCACCACCCAGCCAGCCACG
CCCACCCGCCCTGGCAACCAGAGCAGTCCTGAGCAGCTCAGTGCCTCGGTGTCCCTGCTTGATGACGAG
CTCATGTCTCTGGGCCTAAGTGACCCGACACCACCTTCAGGCACCAGCTCAGATAGTGTGGGGTGGGACA
ACTTCCAGTCATCAGATGGCACTGAATCCTCAGTCCCTCCTCCAGCCCAGGCCCCCAGCATGGACTGCCG
ACCCCCAGCCCAGGCCCCTCCACCAACGAGCAGTGGCCTGGACGACCTGGACCTCTTGGGGAAAACCCT
TATGCAGCAGGCTCTGCCTCCGGAAGCCCAGCAAGTGCGGTGGGAGAAGCAGCAGCCAGCCCCCCGGC
TCACCCTCCGTGACCTGCAGAGTAAGAGCAGCTCGCCCAGCCCAGGAGCCGCCAGCCTCCTCCACACCA
CGTCCCCAGAGCCCCCTGGGCCTCCACCTCAGGCCACACCCACTGAGTTCTCCCTAACCAGCATCACTGT
GCCCCTGGAGTCTATCAAACCCAAGGGCGAATTCGACCCAGCTTTCTTGTACAAAGTGGTTGATGCTGTTA
ACATGGTGAGCAAGGGCGAGGAGCTGTTCACCGGGGTGGTGCCCATCCTGGTCGAGCTGGACGGCGAC
GTAAACGGCCACAAGTTCAGCGTGTCCGGCGAGGGCGAGGGCGATGCCACCTACGGCAAGCTGACCCTG
AAGTTCATCTGCACCACCGGCAAGCTGCCCGTGCCCTGGCCCACCCTCGTGACCACCTTCACCTACGGCG
TGCAGTGCTTCGCCCGCTACCCCGACCACATGAAGCAGCACGACTTCTTCAAGTCCGCCATGCCCGAAGG
CTACGTCCAGGAGCGCACCATCTTCTTCAAGGACGACGGCAACTACAAGACCCGCGCCGAGGTGAAGTTC
GAGGGCGACACCCTGGTGAACCGCATCGAGCTGAAGGGCATCGACTTCAAGGAGGACGGCAACATCCTG
GGGCACAAGCTGGAGTACAACTACAACAGCCACAAGGTCTATATCACCGCCGACAAGCAGAAGAACGGCA
TCAAGGTGAACTTCAAGACCCGCCACAACATCGAGGACGGCAGCGTGCAGCTCGCCGACCACTACCAGC
AGAACACCCCCATCGGCGACGGCCCCGTGCTGCTGCCCGACAACCACTACCTGAGCACCCAGTCCGCCC
TGAGCAAAGACCCCAACGAGAAGCGCGATCACATGGTCCTGCTGGAGTTCGTGACCGCCGCCGGGATCA
CTCTCGGCATGGACGAGCTGTACAAGTAATGATAAGTTTAAACGGGGGAGGCTAACTGAAACACGGAAGGA

GACAATACCGGAAGGAACCCGCGCTATGACGGCAATAAAAAGACAGAATAAAACGCACGGGTGTTGGGTC
GTTTGTTCATAAACGCGGGGTTCGGTCCCAGGGCTGGCACTCTGTCGATACCCCACCGAGACCCCATTGG
GGCCAATACGCCCGCGTTTCTTCCTTTTCCCCACCCCACCCCCCAAGTTCGGGTGAAGGCCCAGGGCTCG
CAGCCAACGTCGGGGCGGCAGGCCCTGCCATAGCAGATCTGCGCAGCTGGGGCTCTAGGGGGTATCCCC
ACGCGCCCTGTAGCGGCGCATTAAGCGCGGCGGGTGTGGTGGTTACGCGCAGCGTGACCGCTACACTTG
CCAGCGCCCTAGCGCCCGCTCCTTTCGCTTTCTTCCCTTCCTTTCTCGCCACGTTCGCCGGCTTTCCCCGT
CAAGCTCTAAATCGGGGGCTCCCTTTAGGGTTCCGATTTAGTGCTTTACGGCACCTCGACCCCAAAAAACT
TGATTAGGGTGATGGTTCACGTAGTGGGCCATCGCCCTGATAGACGGTTTTTCGCCCTTTGACGTTGGAGT
CCACGTTCTTTAATAGTGGACTCTTGTTCCAAACTGGAACAACACTCAACCCTATCTCGGTCTATTCTTTTGA
TTTATAAGGGATTTTGCCGATTTCGGCCTATTGGTTAAAAAATGAGCTGATTTAACAAAAATTTAACGCGAATT
AATTCTGTGGAATGTGTGTCAGTTAGGGTGTGGAAAGTCCCCAGGCTCCCCAGCAGGCAGAAGTATGCAA
AGCATGCATCTCAATTAGTCAGCAACCAGGTGTGGAAAGTCCCCAGGCTCCCCAGCAGGCAGAAGTATGC
AAAGCATGCATCTCAATTAGTCAGCAACCATAGTCCCGCCCCTAACTCCGCCCATCCCGCCCCTAACTCCG
CCCAGTTCCGCCCATTCTCCGCCCCATGGCTGACTAATTTTTTTTATTTATGCAGAGGCCGAGGCCGCCTCT
GCCTCTGAGCTATTCCAGAAGTAGTGAGGAGGCTTTTTTGGAGGCCTAGGCTTTTGCAAAAAGCTCCCGGG
AGCTTGTATATCCATTTTCGGATCTGATCAGCACGTGTTGACAATTAATCATCGGCATAGTATATCGGCATAGT
ATAATACGACAAGGTGAGGAACTAAACCATGGCCAAGCCTTTGTCTCAAGAAGAATCCACCCTCATTGAAAG
AGCAACGGCTACAATCAACAGCATCCCCATCTCTGAAGACT

Seq_mGGA1-delVHS_GFP

GAGGAGCAGGACTGACACGTGCTACGAGATTTCGATTCCACCGCCGCCTTCTATGAAAGGTTGGGCTTCG
GAATCGTTTTCCGGGACGCCGGCTGGATGATCCTCCAGCGCGGGGATCTCATGCTGGAGTTCTTCGCCCA
CCCCAACTTGTTTATTGCAGCTTATAATGGTTACAAATAAAGCAATAGCATCACAAATTTCACAAATAAAGCAT
TTTTTTCACTGCATTCTAGTTGTGGTTTGTCCAAACTCATCAATGTATCTTATCATGTCTGTATACCGTCGACC
TCTAGCTAGAGCTTGGCGTAATCATGGTCATAGCTGTTTCCTGTGTGAAATTGTTATCCGCTCACAATTCCAC
ACAACATACGAGCCGGAAGCATAAAGTGTAAAGCCTGGGGTGCCTAATGAGTGAGCTAACTCACATTAATTG
CGTTGCGCTCACTGCCCGCTTTCCAGTCGGGAAACCTGTCGTGCCAGCTGCATTAATGAATCGGCCAACG
CGCGGGGAGAGGCGGTTTGCGTATTGGGCGCTCTTCCGCTTCCTCGCTCACTGACTCGCTGCGCTCGGT
CGTTCGGCTGCGGCGAGCGGTATCAGCTCACTCAAAGGCGGTAATACGGTTATCCACAGAATCAGGGGATA
ACGCAGGAAAGAACATGTGAGCAAAAGGCCAGCAAAAGGCCAGGAACCGTAAAAAGGCCGCGTTGCTGG
CGTTTTTCCATAGGCTCCGCCCCCCTGACGAGCATCACAAAAATCGACGCTCAAGTCAGAGGTGGCGAAA
CCCGACAGGACTATAAAGATACCAGGCGTTTCCCCCTGGAAGCTCCCTCGTGCGCTCTCCTGTTCCGACC
CTGCCGCTTACCGGATACCTGTCCGCCTTTCTCCCTTCGGGAAGCGTGGCGCTTTCTCATAGCTCACGCTG
TAGGTATCTCAGTTCGGTGTAGGTCGTTCGCTCCAAGCTGGGCTGTGTGCACGAACCCCCCGTTCAGCCC
GACCGCTGCGCCTTATCCGGTAACTATCGTCTTGAGTCCAACCCGGTAAGACACGACTTATCGCCACTGGC
AGCAGCCACTGGTAACAGGATTAGCAGAGCGAGGTATGTAGGCGGTGCTACAGAGTTCTTGAAGTGGTGG
CCTAACTACGGCTACACTAGAAGAACAGTATTTGGTATCTGCGCTCTGCTGAAGCCAGTTACCTTCGGAAAA
AGAGTTGGTAGCTCTTGATCCGGCAAACAAACCACCGCTGGTAGCGGTGGTTTTTTTGTTTGCAAGCAGCA
GATTACGCGCAGAAAAAAAGGATCTCAAGAAGATCCTTTGATCTTTTCTACGGGGTCTGACGCTCAGTGGA
ACGAAAACTCACGTTAAGGGATTTTGGTCATGAGATTATCAAAAAGGATCTTCACCTAGATCCTTTTAAATTAA
AAATGAAGTTTTAAATCAATCTAAAGTATATATGAGTAAACTTGGTCTGACAGTTACCAATGCTTAATCAGTGA
GGCACCTATCTCAGCGATCTGTCTATTTCGTTCATCCATAGTTGCCTGACTCCCCGTCGTGTAGATAACTAC
GATACGGGAGGGCTTACCATCTGGCCCCAGTGCTGCAATGATACCGCGAGACCCACGCTCACCGGCTCCA
GATTTATCAGCAATAAACCAGCCAGCCGGAAGGGCCGAGCGCAGAAGTGGTCCTGCAACTTTATCCGCCTC
CATCCAGTCTATTAATTGTTGCCGGGAAGCTAGAGTAAGTAGTTCGCCAGTTAATAGTTTGCGCAACGTTGTT
GCCATTGCTACAGGCATCGTGGTGTCACGCTCGTCGTTTGGTATGGCTTCATTCAGCTCCGGTTCCCAACG

ATCAAGGCGAGTTACATGATCCCCCATGTTGTGCAAAAAAGCGGTTAGCTCCTTCGGTCCTCCGATCGTTG
TCAGAAGTAAGTTGGCCGCAGTGTTATCACTCATGGTTATGGCAGCACTGCATAATTCTCTTACTGTCATGC
CATCCGTAAGATGCTTTTCTGTGACTGGTGAGTACTCAACCAAGTCATTCTGAGAATAGTGTATGCGGCGAC
CGAGTTGCTCTTGCCCGGCGTCAATACGGGATAATACCGCGCCACATAGCAGAACTTTAAAAGTGCTCATCA
TTGGAAAACGTTCTTCGGGGCGAAAACTCTCAAGGATCTTACCGCTGTTGAGATCCAGTTCGATGTAACCC
ACTCGTGCACCCAACTGATCTTCAGCATCTTTTACTTTCACCAGCGTTTCTGGGTGAGCAAAAACAGGAAG
GCAAAATGCCGCAAAAAAGGGAATAAGGGCGACACGGAAATGTTGAATACTCATACTCTTCCTTTTTCAATAT
TATTGAAGCATTTATCAGGGTTATTGTCTCATGAGCGGATACATATTTGAATGTATTTAGAAAAATAAACAAATA
GGGGTTCCGCGCACATTTCCCCGAAAAGTGCCACCTGACGTCGACGGATCGGGAGATCTCCCGATCCCCT
ATGGTGCACTCTCAGTACAATCTGCTCTGATGCCGCATAGTTAAGCCAGTATCTGCTCCCTGCTTGTGTGTT
GGAGGTCGCTGAGTAGTGCGCGAGCAAAATTTAAGCTACAACAAGGCAAGGCTTGACCGACAATTGCATG
AAGAATCTGCTTAGGGTTAGGCGTTTTGCGCTGCTTCGCGATGTACGGGCCAGATATACGCGTTGACATTG
ATTATTGACTAGTTATTAATAGTAATCAATTACGGGGTCATTAGTTCATAGCCCATATATGGAGTTCCGCGTTAC
ATAACTTACGGTAAATGGCCCGCCTGGCTGACCGCCCAACGACCCCCGCCCATTGACGTCAATAATGACGT
ATGTTCCCATAGTAACGCCAATAGGGACTTTCCATTGACGTCAATGGGTGGAGTATTTACGGTAAACTGCCC
ACTTGGCAGTACATCAAGTGTATCATATGCCAAGTACGCCCCCTATTGACGTCAATGACGGTAAATGGCCCG
CCTGGCATTATGCCCAGTACATGACCTTATGGGACTTTCCTACTTGGCAGTACATCTACGTATTAGTCATCGC
TATTACCATGGTGATGCGGTTTTGGCAGTACATCAATGGGCGTGGATAGCGGTTTGACTCACGGGGATTTCC
AAGTCTCCACCCCATTGACGTCAATGGGAGTTTGTTTTGGCACCAAAATCAACGGGACTTTCCAAAATGTC
GTAACAACTCCGCCCCATTGACGCAAATGGGCGGTAGGCGTGTACGGTGGGAGGTCTATATAAGCAGAGCT
CTCTGGCTAACTAGAGAACCCACTGCTTACTGGCTTATCGAAATTAATACGACTCACTATAGGGAGACCCAA
GCTGGCTAGTTAAGCTGAGCATCAACAAGTTTGTACAAAAAAGCAGGCTCCGAATTCGCCCTTGCCGCCAT
GGATGAGGAGAAGTCCAAGATGCTGGCCCGCCTGCTGAAGAGCTCACACCCTGAGGACCTCCGGGCTGC
CAATAAGCTCATCAAAGAGATGGTGCAGGAGGACCAGAAGCGGATGGAAAAGATCTCCAAGCGGGTGAAT
GCCATCGAGGAGGTCAACAACAATGTGAAGTTGCTGACAGAGATGGTGATGAGCCACAGCCAGGGTGCTG
CATCCAGCAGCAGTGAGGACCTCATGAAGGAACTGTACCAGCGCTGTGAGCGCATGCGACCCACACTCTT
CCGACTGGCCAGTGACACGGAAGACAATGATGAGGCCTTAGCTGAGATCCTGCAGGCTAATGACAATCTCA
CCCAGGTGATCAACCTGTACAAGCAGCTGGTCCGGGGCGAGGAGGTCAACGGTGATGCCACAGCCAGCT
CCATTCCTGGAAGCACGTCAGCCCTGCTGGACCTCTCAGGCCTGGACCTCCCTCCCCCGGGCACCACCC
AGCCAGCCACGCCCACCCGCCCTGGCAACCAGAGCAGTCCTGAGCAGCTCAGTGCCTCGGTGTCCCTGC
TTGATGACGAGCTCATGTCTCTGGGCCTAAGTGACCCGACACCACCTTCAGGCACCAGCTCAGATAGTGTG
GGGTGGGACAACTTCCAGTCATCAGATGGCACTGAATCCTCAGTCCCTCCTCCAGCCCAGGCCCCCAGCA
TGGACTGCCGACCCCCAGCCCAGGCCCCTCCACCAACGAGCAGTGGCCTGGACGACCTGGACCTCTTGG
GGAAAACCCTTATGCAGCAGGCTCTGCCTCCGGAAGCCCAGCAAGTGCGGTGGGAGAAGCAGCAGCCAG
CCCCCCGGCTCACCCTCCGTGACCTGCAGAGTAAGAGCAGCTCGCCCAGCCCAGGAGCCGCCAGCCTCC
TCCACACCACGTCCCCAGAGCCCCCTGGGCCTCCACCTCAGGCCACACCCACTGAGTTCTCCCTAACCAG
CATCACTGTGCCCCTGGAGTCTATCAAACCCAGCAGCATCTTGCCAGTGACCGTTTATGACCAGCATGGCT
TCCGTGTCCTCTTCCATTTTGCTCGGGACCCACTGCCAGGGCGCTCCGATGTGCTGGTGGTGGTGGTCTC
TATGCTGAGCACGGCGCCCCAGCCCATCCGGAACATCGTTTTCCAGTCAGCCGTCCCCAAGGTCATGAAG
GTGAGGCTGCAGCCACCTTCCGGCACAGAGCTGCCAGCGTTCAACCCCATCGTCCACCCCTCAGCCATCA
CCCAGGTCCTGCTCCTTGCTAACCCCCAGAAGGAGAAGGTTCGCCTCCGCTACAAGCTCATCTTCACTATG
GGCGACCAGACCTACAATGAGATGGAGATGTGGATCAGTTCCCCCCACCAGAGACCTGGGGGAGCCTC
AAGGGCGAATTCGACCCAGCTTTCTTGTACAAAGTGGTTGATGCTGTTAACATGGTGAGCAAGGGCGAGGA
GCTGTTCACCGGGGTGGTGCCCATCCTGGTCGAGCTGGACGGCGACGTAAACGGCCACAAGTTCAGCGT
GTCCGGCGAGGGCGAGGGCGATGCCACCTACGGCAAGCTGACCCTGAAGTTCATCTGCACCACCGGCAA
GCTGCCCGTGCCCTGGCCCACCCTCGTGACCACCTTCACCTACGGCGTGCAGTGCTTCGCCCGCTACCC
CGACCACATGAAGCAGCACGACTTCTTCAAGTCCGCCATGCCCGAAGGCTACGTCCAGGAGCGCACCATC
TTCTTCAAGGACGACGGCAACTACAAGACCCGCGCCGAGGTGAAGTTCGAGGGCGACACCCTGGTGAAC

CGCATCGAGCTGAAGGGCATCGACTTCAAGGAGGACGGCAACATCCTGGGGCACAAGCTGGAGTACAACT
ACAACAGCCACAAGGTCTATATCACCGCCGACAAGCAGAAGAACGGCATCAAGGTGAACTTCAAGACCCG
CCACAACATCGAGGACGGCAGCGTGCAGCTCGCCGACCACTACCAGCAGAACACCCCCATCGGCGACGG
CCCCGTGCTGCTGCCCGACAACCACTACCTGAGCACCCAGTCCGCCCTGAGCAAAGACCCCAACGAGAA
GCGCGATCACATGGTCCTGCTGGAGTTCGTGACCGCCGCCGGGATCACTCTCGGCATGGACGAGCTGTA
CAAGTAATGATAAGTTTAAACGGGGGAGGCTAACTGAAACACGGAAGGAGACAATACCGGAAGGAACCCG
CGCTATGACGGCAATAAAAAGACAGAATAAAACGCACGGGTGTTGGGTCGTTTGTTCATAAACGCGGGGTT
CGGTCCCAGGGCTGGCACTCTGTCGATACCCCACCGAGACCCCATTGGGGCCAATACGCCCGCGTTTCTT
CCTTTTCCCCACCCCACCCCCCAAGTTCGGGTGAAGGCCCAGGGCTCGCAGCCAACGTCGGGGCGGCA
GGCCCTGCCATAGCAGATCTGCGCAGCTGGGGCTCTAGGGGGTATCCCCACGCGCCCTGTAGCGGCGCA
TTAAGCGCGGCGGGTGTGGTGGTTACGCGCAGCGTGACCGCTACACTTGCCAGCGCCCTAGCGCCCGCT
CCTTTCGCTTTCTTCCCTTCCTTTCTCGCCACGTTCGCCGGCTTTCCCCGTCAAGCTCTAAATCGGGGGCT
CCCTTTAGGGTTCCGATTTAGTGCTTTACGGCACCTCGACCCCAAAAAACTTGATTAGGGTGATGGTTCACG
TAGTGGGCCATCGCCCTGATAGACGGTTTTTCGCCCTTTGACGTTGGAGTCCACGTTCTTTAATAGTGGACT
CTTGTTCCAAACTGGAACAACACTCAACCCTATCTCGGTCTATTCTTTTGATTTATAAGGGATTTTGCCGATTT
CGGCCTATTGGTTAAAAAATGAGCTGATTTAACAAAAATTTAACGCGAATTAATTCTGTGGAATGTGTGTCAG
TTAGGGTGTGGAAAGTCCCCAGGCTCCCCAGCAGGCAGAAGTATGCAAAGCATGCATCTCAATTAGTCAGC
AACCAGGTGTGGAAAGTCCCCAGGCTCCCCAGCAGGCAGAAGTATGCAAAGCATGCATCTCAATTAGTCA
GCAACCATAGTCCCGCCCCTAACTCCGCCCATCCCGCCCCTAACTCCGCCCAGTTCCGCCCATTCTCCGC
CCCATGGCTGACTAATTTTTTTTATTTATGCAGAGGCCGAGGCCGCCTCTGCCTCTGAGCTATTCCAGAAGT
AGTGAGGAGGCTTTTTTGGAGGCCTAGGCTTTTGCAAAAAGCTCCCGGGAGCTTGTATATCCATTTTCGGAT
CTGATCAGCACGTGTTGACAATTAATCATCGGCATAGTATATCGGCATAGTATAATACGACAAGGTGAGGAAC
TAAACCATGGCCAAGCCTTTGTCTCAAGAAGAATCCACCCTCATTGAAAGAGCAACGGCTACAATCAACAG
CATCCCCATCTCTGAAGACTACAGCGTCGCCAGCGCAGCTCTCTCTAGCGACGGCCGCATCTTCACTGGT
GTCAATGTATATCATTTTACTGGGGGACCTTGTGCAGAACTCGTGGTGCTGGGCACTGCTGCTGCTGCGGC
AGCTGGCAACCTGACTTGTATCGTCGCGATCGGAAATGAGAACAGGGGCATCTTGAGCCCCTGCGGACGG
TGCCGACAGGTGCTTCTCGATCTGCATCCTGGGATCAAAGCCATAGTGAAGGACAGTGATGGACAGCCGA
CGGCAGTTGGGATTCGTGAATTGCTGCCCTCTGGTTATGTGTGGGAGGGCTAAGCACTTCGTGGCC

Seq_mGGA1-VHS+GAT_GFP

ACTGATCTTCAGCATCTTTTACTTTCACCAGCGTTTCTGGGTGAGCAAAAACAGGAAGGCAAAATGCCGCA
AAAAAGGGAATAAGGGCGACACGGAAATGTTGAATACTCATACTCTTCCTTTTTCAATATTATTGAAGCATTTA
TCAGGGTTATTGTCTCATGAGCGGATACATATTTGAATGTATTTAGAAAAATAAACAAATAGGGGTTCCGCGC
ACATTTCCCCGAAAAGTGCCACCTGACGTCGACGGATCGGGAGATCTCCCGATCCCCTATGGTGCACTCTC
AGTACAATCTGCTCTGATGCCGCATAGTTAAGCCAGTATCTGCTCCCTGCTTGTGTGTTGGAGGTCGCTGA
GTAGTGCGCGAGCAAAATTTAAGCTACAACAAGGCAAGGCTTGACCGACAATTGCATGAAGAATCTGCTTA
GGGTTAGGCGTTTTGCGCTGCTTCGCGATGTACGGGCCAGATATACGCGTTGACATTGATTATTGACTAGTT
ATTAATAGTAATCAATTACGGGGTCATTAGTTCATAGCCCATATATGGAGTTCCGCGTTACATAACTTACGGTA
AATGGCCCGCCTGGCTGACCGCCCAACGACCCCCGCCCATTGACGTCAATAATGACGTATGTTCCCATAGT
AACGCCAATAGGGACTTTCCATTGACGTCAATGGGTGGAGTATTTACGGTAAACTGCCCACTTGGCAGTACA
TCAAGTGTATCATATGCCAAGTACGCCCCCTATTGACGTCAATGACGGTAAATGGCCCGCCTGGCATTATGC
CCAGTACATGACCTTATGGGACTTTCCTACTTGGCAGTACATCTACGTATTAGTCATCGCTATTACCATGGTG
ATGCGGTTTTGGCAGTACATCAATGGGCGTGGATAGCGGTTTGACTCACGGGGATTTCCAAGTCTCCACCC
CATTGACGTCAATGGGAGTTTGTTTTGGCACCAAAATCAACGGGACTTTCCAAAATGTCGTAACAACTCCGC
CCCATTGACGCAAATGGGCGGTAGGCGTGTACGGTGGGAGGTCTATATAAGCAGAGCTCTCTGGCTAACTA
GAGAACCCACTGCTTACTGGCTTATCGAAATTAATACGACTCACTATAGGGAGACCCAAGCTGGCTAGTTAA

GCTGAGCATCAACAAGTTTGTACAAAAAAGCAGGCTCCGAATTCGCCCTTGCCGCCATGGAGCCCGCGAT
GGAGCCGGAGACTCTGGAGGCACGAATCAACAGAGCCACAAATCCCCTGAACAAGGAGCTGAACTGGGC
CAGCATCAACAGTTTCTGCGAGCAGCTCAACGAAGACTTTGAGGGGCCTCCACTTGCCACTCGCTTGCTG
GCCCACAAGATCCAGTCCCCACAGGAATGGGAAGCCATCCAGGCCTTGACGGTTCTGGAGACGTGCATGA
AGAGCTGCGGCAAGAGGTTCCATGATGAGGTGGGCAAGTTCCGCTTCCTCAACGAGCTCATCAAGGTTGT
GTCTCCCAAGTACTTGGGCTCCCGGACATCTGAGAAGGTGAAGAGTAAGATCTTGGAGCTGCTGTACAGCT
GGACGGTTTGCCTGCCTGAGGAGGTGAAGATTGCAGAAGCCTACCAGATGCTGAAGAAGCAGGGGATTGT
GAAGTCGGACCCCAAGCTTCCAGAGGATGCCATCTTTCCCCTCCCCCCTCCCCGGCCCAAGAATGTGATC
TTTGAAGATGAGGAGAAGTCCAAGATGCTGGCCCGCCTGCTGAAGAGCTCACACCCTGAGGACCTCCGG
GCTGCCAATAAGCTCATCAAAGAGATGGTGCAGGAGGACCAGAAGCGGATGGAAAAGATCTCCAAGCGGG
TGAATGCCATCGAGGAGGTCAACAACAATGTGAAGTTGCTGACAGAGATGGTGATGAGCCACAGCCAGGG
TGCTGCATCCAGCAGCAGTGAGGACCTCATGAAGGAACTGTACCAGCGCTGTGAGCGCATGCGACCCACA
CTCTTCCGACTGGCCAGTGACACGGAAGACAATGATGAGGCCTTAGCTGAGATCCTGCAGGCTAATGACAA
TCTCACCCAGGTGATCAACCTGTACAAGCAGCTGGTCCGGAAGGGCGAATTCGACCCAGCTTTCTTGTACA
AAGTGGTTGATGCTGTTAACATGGTGAGCAAGGGCGAGGAGCTGTTCACCGGGGTGGTGCCCATCCTGGT
CGAGCTGGACGGCGACGTAAACGGCCACAAGTTCAGCGTGTCCGGCGAGGGCGAGGGCGATGCCACCT
ACGGCAAGCTGACCCTGAAGTTCATCTGCACCACCGGCAAGCTGCCCGTGCCCTGGCCCACCCTCGTGA
CCACCTTCACCTACGGCGTGCAGTGCTTCGCCCGCTACCCCGACCACATGAAGCAGCACGACTTCTTCAA
GTCCGCCATGCCCGAAGGCTACGTCCAGGAGCGCACCATCTTCTTCAAGGACGACGGCAACTACAAGACC
CGCGCCGAGGTGAAGTTCGAGGGCGACACCCTGGTGAACCGCATCGAGCTGAAGGGCATCGACTTCAAG
GAGGACGGCAACATCCTGGGGCACAAGCTGGAGTACAACTACAACAGCCACAAGGTCTATATCACCGCCG
ACAAGCAGAAGAACGGCATCAAGGTGAACTTCAAGACCCGCCACAACATCGAGGACGGCAGCGTGCAGC
TCGCCGACCACTACCAGCAGAACACCCCCATCGGCGACGGCCCCGTGCTGCTGCCCGACAACCACTACC
TGAGCACCCAGTCCGCCCTGAGCAAAGACCCCAACGAGAAGCGCGATCACATGGTCCTGCTGGAGTTCG
TGACCGCCGCCGGGATCACTCTCGGCATGGACGAGCTGTACAAGTAATGATAAGTTTAAACGGGGGAGGC
TAACTGAAACACGGAAGGAGACAATACCGGAAGGAACCCGCGCTATGACGGCAATAAAAAGACAGAATAAA
ACGCACGGGTGTTGGGTCGTTTGTTCATAAACGCGGGGTTCGGTCCCAGGGCTGGCACTCTGTCGATACC
CCACCGAGACCCCATTGGGGCCAATACGCCCGCGTTTCTTCCTTTTCCCCACCCCACCCCCCAAGTTCGG
GTGAAGGCCCAGGGCTCGCAGCCAACGTCGGGGCGGCAGGCCCTGCCATAGCAGATCTGCGCAGCTGG
GGCTCTAGGGGGTATCCCCACGCGCCCTGTAGCGGCGCATTAAGCGCGGCGGGTGTGGTGGTTACGCGC
AGCGTGACCGCTACACTTGCCAGCGCCCTAGCGCCCGCTCCTTTCGCTTTCTTCCCTTCCTTTCTCGCCAC
GTTCGCCGGCTTTCCCCGTCAAGCTCTAAATCGGGGGCTCCCTTTAGGGTTCCGATTTAGTGCTTTACGGC
ACCTCGACCCCAAAAAACTTGATTAGGGTGATGGTTCACGTAGTGGGCCATCGCCCTGATAGACGGTTTTT
CGCCCTTTGACGTTGGAGTCCACGTTCTTTAATAGTGGACTCTTGTTCCAAACTGGAACAACACTCAACCCT
ATCTCGGTCTATTCTTTTGATTTATAAGGGATTTTGCCGATTTCGGCCTATTGGTTAAAAAATGAGCTGATTTA
ACAAAAATTTAACGCGAATTAATTCTGTGGAATGTGTGTCAGTTAGGGTGTGGAAAGTCCCCAGGCTCCCCA
GCAGGCAGAAGTATGCAAAGCATGCATCTCAATTAGTCAGCAACCAGGTGTGGAAAGTCCCCAGGCTCCC
CAGCAGGCAGAAGTATGCAAAGCATGCATCTCAATTAGTCAGCAACCATAGTCCCGCCCCTAACTCCGCCC
ATCCCGCCCCTAACTCCGCCCAGTTCCGCCCATTCTCCGCCCCATGGCTGACTAATTTTTTTTATTTATGCA
GAGGCCGAGGCCGCCTCTGCCTCTGAGCTATTCCAGAAGTAGTGAGGAGGCTTTTTTGGAGGCCTAGGCT
TTTGCAAAAAGCTCCCGGGAGCTTGTATATCCATTTTCGGATCTGATCAGCACGTGTTGACAATTAATCATCG
GCATAGTATATCGGCATAGTATAATACGACAAGGTGAGGAACTAAACCATGGCCAAGCCTTTGTCTCAAGAA
GAATCCACCCTCATTGAAAGAGCAACGGCTACAATCAACAGCATCCCCATCTCTGAAGACTACAGCGTCGC
CAGCGCAGCTCTCTCTAGCGACGGCCGCATCTTCACTGGTGTCAATGTATATCATTTTACTGGGGGACCTT
GTGCAGAACTCGTGGTGCTGGGCACTGCTGCTGCTGCGGCAGCTGGCAACCTGACTTGTATCGTCGCGAT
CGGAAATGAGAACAGGGGCATCTTGAGCCCCTGCGGACGGTGCCGACAGGTGCTTCTCGATCTGCATCCT
GGGATCAAAGCCATAGTGAAGGACAGTGATGGACAGCCGACGGCAGTTGGGATTCGTGAATTGCTGCCCT
CTGGTTATGTGTGGGAGGGCTAAGCACTTCGTGGCCGAGGAGCAGGACTGACACGTGCTACGAGATTTCG

ATTCCACCGCCGCCTTCTATGAAAGGTTGGGCTTCGGAATCGTTTTCCGGGACGCCGGCTGGATGATCCTC
CAGCGCGGGGATCTCATGCTGGAGTTCTTCGCCCACCCCAACTTGTTTATTGCAGCTTATAATGGTTACAAA
TAAAGCAATAGCATCACAAATTTCACAAATAAAGCATTTTTTTCACTGCATTCTAGTTGTGGTTTGTCCAAACT
CATCAATGTATCTTATCATGTCTGTATACCGTCGACCTCTAGCTAGAGCTTGGCGTAATCATGGTCATAGCTG
TTTCCTGTGTGAAATTGTTATCCGCTCACAATTCCACACAACATACGAGCCGGAAGCATAAAGTGTAAAGCC
TGGGGTGCCTAATGAGTGAGCTAACTCACATTAATTGCGTTGCGCTCACTGCCCGCTTTCCAGTCGGGAAA
CCTGTCGTGCCAGCTGCATTAATGAATCGGCCAACGCGCGGGGAGAGGCGGTTTGCGTATTGGGCGCTCT
TCCGCTTCCTCGCTCACTGACTCGCTGCGCTCGGTCGTTCGGCTGCGGCGAGCGGTATCAGCTCACTCAA
AGGCGGTAATACGGTTATCCACAGAATCAGGGGATAACGCAGGAAAGAACATGTGAGCAAAAGGCCAGCAA
AAGGCCAGGAACCGTAAAAAGGCCGCGTTGCTGGCGTTTTTCCATAGGCTCCGCCCCCCTGACGAGCATC
ACAAAAATCGACGCTCAAGTCAGAGGTGGCGAAACCCGACAGGACTATAAAGATACCAGGCGTTTCCCCCT
GGAAGCTCCCTCGTGCGCTCTCCTGTTCCGACCCTGCCGCTTACCGGATACCTGTCCGCCTTTCTCCCTT
CGGGAAGCGTGGCGCTTTCTCATAGCTCACGCTGTAGGTATCTCAGTTCGGTGTAGGTCGTTCGCTCCAAG
CTGGGCTGTGTGCACGAACCCCCCGTTCAGCCCGACCGCTGCGCCTTATCCGGTAACTATCGTCTTGAGT
CCAACCCGGTAAGACACGACTTATCGCCACTGGCAGCAGCCACTGGTAACAGGATTAGCAGAGCGAGGTA
TGTAGGCGGTGCTACAGAGTTCTTGAAGTGGTGGCCTAACTACGGCTACACTAGAAGAACAGTATTTGGTAT
CTGCGCTCTGCTGAAGCCAGTTACCTTCGGAAAAAGAGTTGGTAGCTCTTGATCCGGCAAACAAACCACC
GCTGGTAGCGGTGGTTTTTTTGTTTGCAAGCAGCAGATTACGCGCAGAAAAAAAGGATCTCAAGAAGATCC
TTTGATCTTTTCTACGGGGTCTGACGCTCAGTGGAACGAAAACTCACGTTAAGGGATTTTGGTCATGAGATT
ATCAAAAAGGATCTTCACCTAGATCCTTTTAAATTAAAAATGAAGTTTTAAATCAATCTAAAGTATATATGAGTAA
ACTTGGTCTGACAGTTACCAATGCTTAATCAGTGAGGCACCTATCTCAGCGATCTGTCTATTTCGTTCATCCA
TAGTTGCCTGACTCCCCGTCGTGTAGATAACTACGATACGGGAGGGCTTACCATCTGGCCCCAGTGCTGCA
ATGATACCGCGAGACCCACGCTCACCGGCTCCAGATTTATCAGCAATAAACCAGCCAGCCGGAAGGGCCG
AGCGCAGAAGTGGTCCTGCAACTTTATCCGCCTCCATCCAGTCTATTAATTGTTGCCGGGAAGCTAGAGTAA
GTAGTTCGCCAGTTAATAGTTTGCGCAACGTTGTTGCCATTGCTACAGGCATCGTGGTGTCACGCTCGTCG
TTTGGTATGGCTTCATTCAGCTCCGGTTCCCAACGATCAAGGCGAGTTACATGATCCCCCATGTTGTGCAAA
AAAGCGGTTAGCTCCTTCGGTCCTCCGATCGTTGTCAGAAGTAAGTTGGCCGCAGTGTTATCACTCATGGT
TATGGCAGCACTGCATAATTCTCTTACTGTCATGCCATCCGTAAGATGCTTTTCTGTGACTGGTGAGTACTCA
ACCAAGTCATTCTGAGAATAGTGTATGCGGCGACCGAGTTGCTCTTGCCCGGCGTCAATACGGGATAATAC
CGCGCCACATAGCAGAACTTTAAAAGTGCTCATCATTGGAAAACGTTCTTCGGGGCGAAAACTCTCAAGGA
TCTTACCGCTGTTGAGATCCAGTTCGATGTAACCCACTCGTGCACCCA

Seq_mGGA1-Hinge+GAE_GFP

TACAGCGTCGCCAGCGCAGCTCTCTCTAGCGACGGCCGCATCTTCACTGGTGTCAATGTATATCATTTTACT
GGGGGACCTTGTGCAGAACTCGTGGTGCTGGGCACTGCTGCTGCTGCGGCAGCTGGCAACCTGACTTGT
ATCGTCGCGATCGGAAATGAGAACAGGGGCATCTTGAGCCCCTGCGGACGGTGCCGACAGGTGCTTCTC
GATCTGCATCCTGGGATCAAAGCCATAGTGAAGGACAGTGATGGACAGCCGACGGCAGTTGGGATTCGTG
AATTGCTGCCCTCTGGTTATGTGTGGGAGGGCTAAGCACTTCGTGGCCGAGGAGCAGGACTGACACGTGC
TACGAGATTTCGATTCCACCGCCGCCTTCTATGAAAGGTTGGGCTTCGGAATCGTTTTCCGGGACGCCGGC
TGGATGATCCTCCAGCGCGGGGATCTCATGCTGGAGTTCTTCGCCCACCCCAACTTGTTTATTGCAGCTTAT
AATGGTTACAAATAAAGCAATAGCATCACAAATTTCACAAATAAAGCATTTTTTTCACTGCATTCTAGTTGTGG
TTTGTCCAAACTCATCAATGTATCTTATCATGTCTGTATACCGTCGACCTCTAGCTAGAGCTTGGCGTAATCAT
GGTCATAGCTGTTTCCTGTGTGAAATTGTTATCCGCTCACAATTCCACACAACATACGAGCCGGAAGCATAA
AGTGTAAAGCCTGGGGTGCCTAATGAGTGAGCTAACTCACATTAATTGCGTTGCGCTCACTGCCCGCTTTC
CAGTCGGGAAACCTGTCGTGCCAGCTGCATTAATGAATCGGCCAACGCGCGGGGAGAGGCGGTTTGCGT
ATTGGGCGCTCTTCCGCTTCCTCGCTCACTGACTCGCTGCGCTCGGTCGTTCGGCTGCGGCGAGCGGTAT

CAGCTCACTCAAAGGCGGTAATACGGTTATCCACAGAATCAGGGGATAACGCAGGAAAGAACATGTGAGCA
AAAGGCCAGCAAAAGGCCAGGAACCGTAAAAAGGCCGCGTTGCTGGCGTTTTTCCATAGGCTCCGCCCCC
CTGACGAGCATCACAAAAATCGACGCTCAAGTCAGAGGTGGCGAAACCCGACAGGACTATAAAGATACCAG
GCGTTTCCCCCTGGAAGCTCCCTCGTGCGCTCTCCTGTTCCGACCCTGCCGCTTACCGGATACCTGTCCG
CCTTTCTCCCTTCGGGAAGCGTGGCGCTTTCTCATAGCTCACGCTGTAGGTATCTCAGTTCGGTGTAGGTC
GTTCGCTCCAAGCTGGGCTGTGTGCACGAACCCCCCGTTCAGCCCGACCGCTGCGCCTTATCCGGTAACT
ATCGTCTTGAGTCCAACCCGGTAAGACACGACTTATCGCCACTGGCAGCAGCCACTGGTAACAGGATTAGC
AGAGCGAGGTATGTAGGCGGTGCTACAGAGTTCTTGAAGTGGTGGCCTAACTACGGCTACACTAGAAGAAC
AGTATTTGGTATCTGCGCTCTGCTGAAGCCAGTTACCTTCGGAAAAAGAGTTGGTAGCTCTTGATCCGGCAA
ACAAACCACCGCTGGTAGCGGTGGTTTTTTTGTTTGCAAGCAGCAGATTACGCGCAGAAAAAAAGGATCTC
AAGAAGATCCTTTGATCTTTTCTACGGGGTCTGACGCTCAGTGGAACGAAAACTCACGTTAAGGGATTTTG
GTCATGAGATTATCAAAAAGGATCTTCACCTAGATCCTTTTAAATTAAAAATGAAGTTTTAAATCAATCTAAAGT
ATATATGAGTAAACTTGGTCTGACAGTTACCAATGCTTAATCAGTGAGGCACCTATCTCAGCGATCTGTCTAT
TTCGTTCATCCATAGTTGCCTGACTCCCCGTCGTGTAGATAACTACGATACGGGAGGGCTTACCATCTGGCC
CCAGTGCTGCAATGATACCGCGAGACCCACGCTCACCGGCTCCAGATTTATCAGCAATAAACCAGCCAGCC
GGAAGGGCCGAGCGCAGAAGTGGTCCTGCAACTTTATCCGCCTCCATCCAGTCTATTAATTGTTGCCGGGA
AGCTAGAGTAAGTAGTTCGCCAGTTAATAGTTTGCGCAACGTTGTTGCCATTGCTACAGGCATCGTGGTGTC
ACGCTCGTCGTTTGGTATGGCTTCATTCAGCTCCGGTTCCCAACGATCAAGGCGAGTTACATGATCCCCCA
TGTTGTGCAAAAAAGCGGTTAGCTCCTTCGGTCCTCCGATCGTTGTCAGAAGTAAGTTGGCCGCAGTGTTA
TCACTCATGGTTATGGCAGCACTGCATAATTCTCTTACTGTCATGCCATCCGTAAGATGCTTTTCTGTGACTG
GTGAGTACTCAACCAAGTCATTCTGAGAATAGTGTATGCGGCGACCGAGTTGCTCTTGCCCGGCGTCAATA
CGGGATAATACCGCGCCACATAGCAGAACTTTAAAAGTGCTCATCATTGGAAAACGTTCTTCGGGGCGAAA
ACTCTCAAGGATCTTACCGCTGTTGAGATCCAGTTCGATGTAACCCACTCGTGCACCCAACTGATCTTCAGC
ATCTTTTACTTTCACCAGCGTTTCTGGGTGAGCAAAAACAGGAAGGCAAAATGCCGCAAAAAAGGGAATAA
GGGCGACACGGAAATGTTGAATACTCATACTCTTCCTTTTTCAATATTATTGAAGCATTTATCAGGGTTATTGT
CTCATGAGCGGATACATATTTGAATGTATTTAGAAAAATAAACAAATAGGGGTTCCGCGCACATTTCCCCGAA
AAGTGCCACCTGACGTCGACGGATCGGGAGATCTCCCGATCCCCTATGGTGCACTCTCAGTACAATCTGCT
CTGATGCCGCATAGTTAAGCCAGTATCTGCTCCCTGCTTGTGTGTTGGAGGTCGCTGAGTAGTGCGCGAGC
AAAATTTAAGCTACAACAAGGCAAGGCTTGACCGACAATTGCATGAAGAATCTGCTTAGGGTTAGGCGTTTT
GCGCTGCTTCGCGATGTACGGGCCAGATATACGCGTTGACATTGATTATTGACTAGTTATTAATAGTAATCAAT
TACGGGGTCATTAGTTCATAGCCCATATATGGAGTTCCGCGTTACATAACTTACGGTAAATGGCCCGCCTGG
CTGACCGCCCAACGACCCCCGCCCATTGACGTCAATAATGACGTATGTTCCCATAGTAACGCCAATAGGGA
CTTTCCATTGACGTCAATGGGTGGAGTATTTACGGTAAACTGCCCACTTGGCAGTACATCAAGTGTATCATAT
GCCAAGTACGCCCCCTATTGACGTCAATGACGGTAAATGGCCCGCCTGGCATTATGCCCAGTACATGACCT
TATGGGACTTTCCTACTTGGCAGTACATCTACGTATTAGTCATCGCTATTACCATGGTGATGCGGTTTTGGCA
GTACATCAATGGGCGTGGATAGCGGTTTGACTCACGGGGATTTCCAAGTCTCCACCCCATTGACGTCAATG
GGAGTTTGTTTTGGCACCAAAATCAACGGGACTTTCCAAAATGTCGTAACAACTCCGCCCCATTGACGCAA
ATGGGCGGTAGGCGTGTACGGTGGGAGGTCTATATAAGCAGAGCTCTCTGGCTAACTAGAGAACCCACTGC
TTACTGGCTTATCGAAATTAATACGACTCACTATAGGGAGACCCAAGCTGGCTAGTTAAGCTGAGCATCAACA
AGTTTGTACAAAAAAGCAGGCTCCGAATTCGCCCTTGCCGCCATGGGCGAGGAGGTCAACGGTGATGCCA
CAGCCAGCTCCATTCCTGGAAGCACGTCAGCCCTGCTGGACCTCTCAGGCCTGGACCTCCCTCCCCCGG
GCACCACCCAGCCAGCCACGCCCACCCGCCCTGGCAACCAGAGCAGTCCTGAGCAGCTCAGTGCCTCG
GTGTCCCTGCTTGATGACGAGCTCATGTCTCTGGGCCTAAGTGACCCGACACCACCTTCAGGCACCAGCT
CAGATAGTGTGGGGTGGGACAACTTCCAGTCATCAGATGGCACTGAATCCTCAGTCCCTCCTCCAGCCCA
GGCCCCCAGCATGGACTGCCGACCCCCAGCCCAGGCCCCTCCACCAACGAGCAGTGGCCTGGACGACC
TGGACCTCTTGGGGAAAACCCTTATGCAGCAGGCTCTGCCTCCGGAAGCCCAGCAAGTGCGGTGGGAGA
AGCAGCAGCCAGCCCCCCGGCTCACCCTCCGTGACCTGCAGAGTAAGAGCAGCTCGCCCAGCCCAGGA
GCCGCCAGCCTCCTCCACACCACGTCCCCAGAGCCCCCTGGGCCTCCACCTCAGGCCACACCCACTGAG

TTCTCCCTAACCAGCATCACTGTGCCCCTGGAGTCTATCAAACCCAGCAGCATCTTGCCAGTGACCGTTTAT
GACCAGCATGGCTTCCGTGTCCTCTTCCATTTTGCTCGGGACCCACTGCCAGGGCGCTCCGATGTGCTGG
TGGTGGTGGTCTCTATGCTGAGCACGGCGCCCCAGCCCATCCGGAACATCGTTTTCCAGTCAGCCGTCCC
CAAGGTCATGAAGGTGAGGCTGCAGCCACCTTCCGGCACAGAGCTGCCAGCGTTCAACCCCATCGTCCA
CCCCTCAGCCATCACCCAGGTCCTGCTCCTTGCTAACCCCCAGAAGGAGAAGGTTCGCCTCCGCTACAAG
CTCATCTTCACTATGGGCGACCAGACCTACAATGAGATGGGAGATGTGGATCAGTTCCCCAAGGGCGAATT
CGACCCAGCTTTCTTGTACAAAGTGGTTGATGCTGTTAACATGGTGAGCAAGGGCGAGGAGCTGTTCACC
GGGGTGGTGCCCATCCTGGTCGAGCTGGACGGCGACGTAAACGGCCACAAGTTCAGCGTGTCCGGCGA
GGGCGAGGGCGATGCCACCTACGGCAAGCTGACCCTGAAGTTCATCTGCACCACCGGCAAGCTGCCCGT
GCCCTGGCCCACCCTCGTGACCACCTTCACCTACGGCGTGCAGTGCTTCGCCCGCTACCCCGACCACAT
GAAGCAGCACGACTTCTTCAAGTCCGCCATGCCCGAAGGCTACGTCCAGGAGCGCACCATCTTCTTCAAG
GACGACGGCAACTACAAGACCCGCGCCGAGGTGAAGTTCGAGGGCGACACCCTGGTGAACCGCATCGAG
CTGAAGGGCATCGACTTCAAGGAGGACGGCAACATCCTGGGGCACAAGCTGGAGTACAACTACAACAGCC
ACAAGGTCTATATCACCGCCGACAAGCAGAAGAACGGCATCAAGGTGAACTTCAAGACCCGCCACAACATC
GAGGACGGCAGCGTGCAGCTCGCCGACCACTACCAGCAGAACACCCCCATCGGCGACGGCCCCGTGCT
GCTGCCCGACAACCACTACCTGAGCACCCAGTCCGCCCTGAGCAAAGACCCCAACGAGAAGCGCGATCA
CATGGTCCTGCTGGAGTTCGTGACCGCCGCCGGGATCACTCTCGGCATGGACGAGCTGTACAAGTAATGA
TAAGTTTAAACGGGGGAGGCTAACTGAAACACGGAAGGAGACAATACCGGAAGGAACCCGCGCTATGACG
GCAATAAAAAGACAGAATAAAACGCACGGGTGTTGGGTCGTTTGTTCATAAACGCGGGGTTCGGTCCCAGG
GCTGGCACTCTGTCGATACCCCACCGAGACCCCATTGGGGCCAATACGCCCGCGTTTCTTCCTTTTCCCCA
CCCCACCCCCCAAGTTCGGGTGAAGGCCCAGGGCTCGCAGCCAACGTCGGGGCGGCAGGCCCTGCCAT
AGCAGATCTGCGCAGCTGGGGCTCTAGGGGGTATCCCCACGCGCCCTGTAGCGGCGCATTAAGCGCGGC
GGGTGTGGTGGTTACGCGCAGCGTGACCGCTACACTTGCCAGCGCCCTAGCGCCCGCTCCTTTCGCTTTC
TTCCCTTCCTTTCTCGCCACGTTCGCCGGCTTTCCCCGTCAAGCTCTAAATCGGGGGCTCCCTTTAGGGTT
CCGATTTAGTGCTTTACGGCACCTCGACCCCAAAAAACTTGATTAGGGTGATGGTTCACGTAGTGGGCCAT
CGCCCTGATAGACGGTTTTTCGCCCTTTGACGTTGGAGTCCACGTTCTTTAATAGTGGACTCTTGTTCCAAA
CTGGAACAACACTCAACCCTATCTCGGTCTATTCTTTTGATTTATAAGGGATTTTGCCGATTTCGGCCTATTG
GTTAAAAAATGAGCTGATTTAACAAAAATTTAACGCGAATTAATTCTGTGGAATGTGTGTCAGTTAGGGTGTG
GAAAGTCCCCAGGCTCCCCAGCAGGCAGAAGTATGCAAAGCATGCATCTCAATTAGTCAGCAACCAGGTGT
GGAAAGTCCCCAGGCTCCCCAGCAGGCAGAAGTATGCAAAGCATGCATCTCAATTAGTCAGCAACCATAGT
CCCGCCCCTAACTCCGCCCATCCCGCCCCTAACTCCGCCCAGTTCCGCCCATTCTCCGCCCCATGGCTGA
CTAATTTTTTTTATTTATGCAGAGGCCGAGGCCGCCTCTGCCTCTGAGCTATTCCAGAAGTAGTGAGGAGGC
TTTTTTGGAGGCCTAGGCTTTTGCAAAAAGCTCCCGGGAGCTTGTATATCCATTTTCGGATCTGATCAGCAC
GTGTTGACAATTAATCATCGGCATAGTATATCGGCATAGTATAATACGACAAGGTGAGGAACTAAACCATGGC
CAAGCCTTTGTCTCAAGAAGAATCCACCCTCATTGAAAGAGCAACGGCTACAATCAACAGCATCCCCATCTC
TGAAGAC

Seq_mGGA1-GAT+Hinge_GFP

CCCAACTGATCTTCAGCATCTTTTACTTTCACCAGCGTTTCTGGGTGAGCAAAAACAGGAAGGCAAAATGC
CGCAAAAAAGGGAATAAGGGCGACACGGAAATGTTGAATACTCATACTCTTCCTTTTTCAATATTATTGAAGC
ATTTATCAGGGTTATTGTCTCATGAGCGGATACATATTTGAATGTATTTAGAAAAATAAACAAATAGGGGTTCC
GCGCACATTTCCCCGAAAAGTGCCACCTGACGTCGACGGATCGGGAGATCTCCCGATCCCCTATGGTGCA
CTCTCAGTACAATCTGCTCTGATGCCGCATAGTTAAGCCAGTATCTGCTCCCTGCTTGTGTGTTGGAGGTCG
CTGAGTAGTGCGCGAGCAAAATTTAAGCTACAACAAGGCAAGGCTTGACCGACAATTGCATGAAGAATCTG
CTTAGGGTTAGGCGTTTTGCGCTGCTTCGCGATGTACGGGCCAGATATACGCGTTGACATTGATTATTGACT
AGTTATTAATAGTAATCAATTACGGGGTCATTAGTTCATAGCCCATATATGGAGTTCCGCGTTACATAACTTACG

GTAAATGGCCCGCCTGGCTGACCGCCCAACGACCCCCGCCCATTGACGTCAATAATGACGTATGTTCCCAT
AGTAACGCCAATAGGGACTTTCCATTGACGTCAATGGGTGGAGTATTTACGGTAAACTGCCCACTTGGCAGT
ACATCAAGTGTATCATATGCCAAGTACGCCCCCTATTGACGTCAATGACGGTAAATGGCCCGCCTGGCATTA
TGCCCAGTACATGACCTTATGGGACTTTCCTACTTGGCAGTACATCTACGTATTAGTCATCGCTATTACCATG
GTGATGCGGTTTTGGCAGTACATCAATGGGCGTGGATAGCGGTTTGACTCACGGGGATTTCCAAGTCTCCA
CCCCATTGACGTCAATGGGAGTTTGTTTTGGCACCAAAATCAACGGGACTTTCCAAAATGTCGTAACAACTC
CGCCCCATTGACGCAAATGGGCGGTAGGCGTGTACGGTGGGAGGTCTATATAAGCAGAGCTCTCTGGCTA
ACTAGAGAACCCACTGCTTACTGGCTTATCGAAATTAATACGACTCACTATAGGGAGACCCAAGCTGGCTAG
TTAAGCTGAGCATCAACAAGTTTGTACAAAAAAGCAGGCTCCGAATTCGCCCTTGCCGCCATGGATGAGGA
GAAGTCCAAGATGCTGGCCCGCCTGCTGAAGAGCTCACACCCTGAGGACCTCCGGGCTGCCAATAAGCT
CATCAAAGAGATGGTGCAGGAGGACCAGAAGCGGATGGAAAAGATCTCCAAGCGGGTGAATGCCATCGAG
GAGGTCAACAACAATGTGAAGTTGCTGACAGAGATGGTGATGAGCCACAGCCAGGGTGCTGCATCCAGCA
GCAGTGAGGACCTCATGAAGGAACTGTACCAGCGCTGTGAGCGCATGCGACCCACACTCTTCCGACTGGC
CAGTGACACGGAAGACAATGATGAGGCCTTAGCTGAGATCCTGCAGGCTAATGACAATCTCACCCAGGTGA
TCAACCTGTACAAGCAGCTGGTCCGGGGCGAGGAGGTCAACGGTGATGCCACAGCCAGCTCCATTCCTG
GAAGCACGTCAGCCCTGCTGGACCTCTCAGGCCTGGACCTCCCTCCCCCGGGCACCACCCAGCCAGCCA
CGCCCACCCGCCCTGGCAACCAGAGCAGTCCTGAGCAGCTCAGTGCCTCGGTGTCCCTGCTTGATGACG
AGCTCATGTCTCTGGGCCTAAGTGACCCGACACCACCTTCAGGCACCAGCTCAGATAGTGTGGGGTGGGA
CAACTTCCAGTCATCAGATGGCACTGAATCCTCAGTCCCTCCTCCAGCCCAGGCCCCCAGCATGGACTGC
CGACCCCCAGCCCAGGCCCCTCCACCAACGAGCAGTGGCCTGGACGACCTGGACCTCTTGGGGAAAACC
CTTATGCAGCAGGCTCTGCCTCCGGAAGCCCAGCAAGTGCGGTGGGAGAAGCAGCAGCCAGCCCCCCG
GCTCACCCTCCGTGACCTGCAGAGTAAGAGCAGCTCGCCCAGCCCAGGAGCCGCCAGCCTCCTCCACAC
CACGTCCCCAGAGCCCCCTGGGCCTCCACCTCAGGCCACACCCACTGAGTTCTCCCTAACCAGCATCACT
GTGCCCCTGGAGTCTATCAAACCCAAGGGCGAATTCGACCCAGCTTTCTTGTACAAAGTGGTTGATGCTGT
TAACATGGTGAGCAAGGGCGAGGAGCTGTTCACCGGGGTGGTGCCCATCCTGGTCGAGCTGGACGGCGA
CGTAAACGGCCACAAGTTCAGCGTGTCCGGCGAGGGCGAGGGCGATGCCACCTACGGCAAGCTGACCCT
GAAGTTCATCTGCACCACCGGCAAGCTGCCCGTGCCCTGGCCCACCCTCGTGACCACCTTCACCTACGGC
GTGCAGTGCTTCGCCCGCTACCCCGACCACATGAAGCAGCACGACTTCTTCAAGTCCGCCATGCCCGAAG
GCTACGTCCAGGAGCGCACCATCTTCTTCAAGGACGACGGCAACTACAAGACCCGCGCCGAGGTGAAGTT
CGAGGGCGACACCCTGGTGAACCGCATCGAGCTGAAGGGCATCGACTTCAAGGAGGACGGCAACATCCT
GGGGCACAAGCTGGAGTACAACTACAACAGCCACAAGGTCTATATCACCGCCGACAAGCAGAAGAACGGC
ATCAAGGTGAACTTCAAGACCCGCCACAACATCGAGGACGGCAGCGTGCAGCTCGCCGACCACTACCAG
CAGAACACCCCCATCGGCGACGGCCCCGTGCTGCTGCCCGACAACCACTACCTGAGCACCCAGTCCGCC
CTGAGCAAAGACCCCAACGAGAAGCGCGATCACATGGTCCTGCTGGAGTTCGTGACCGCCGCCGGGATC
ACTCTCGGCATGGACGAGCTGTACAAGTAATGATAAGTTTAAACGGGGGAGGCTAACTGAAACACGGAAGG
AGACAATACCGGAAGGAACCCGCGCTATGACGGCAATAAAAAGACAGAATAAAACGCACGGGTGTTGGGT
CGTTTGTTCATAAACGCGGGGTTCGGTCCCAGGGCTGGCACTCTGTCGATACCCCACCGAGACCCCATTG
GGGCCAATACGCCCGCGTTTCTTCCTTTTCCCCACCCCACCCCCCAAGTTCGGGTGAAGGCCCAGGGCTC
GCAGCCAACGTCGGGGCGGCAGGCCCTGCCATAGCAGATCTGCGCAGCTGGGGCTCTAGGGGGTATCCC
CACGCGCCCTGTAGCGGCGCATTAAGCGCGGCGGGTGTGGTGGTTACGCGCAGCGTGACCGCTACACTT
GCCAGCGCCCTAGCGCCCGCTCCTTTCGCTTTCTTCCCTTCCTTTCTCGCCACGTTCGCCGGCTTTCCCC
GTCAAGCTCTAAATCGGGGGCTCCCTTTAGGGTTCCGATTTAGTGCTTTACGGCACCTCGACCCCAAAAA
CTTGATTAGGGTGATGGTTCACGTAGTGGGCCATCGCCCTGATAGACGGTTTTTCGCCCTTTGACGTTGGA
GTCCACGTTCTTTAATAGTGGACTCTTGTTCCAAACTGGAACAACACTCAACCCTATCTCGGTCTATTCTTTT
GATTTATAAGGGATTTTGCCGATTTCGGCCTATTGGTTAAAAAATGAGCTGATTTAACAAAAATTTAACGCGAA
TTAATTCTGTGGAATGTGTGTCAGTTAGGGTGTGGAAAGTCCCCAGGCTCCCCAGCAGGCAGAAGTATGCA
AAGCATGCATCTCAATTAGTCAGCAACCAGGTGTGGAAAGTCCCCAGGCTCCCCAGCAGGCAGAAGTATG
CAAAGCATGCATCTCAATTAGTCAGCAACCATAGTCCCGCCCCTAACTCCGCCCATCCCGCCCCTAACTCC

GCCCAGTTCCGCCCATTCTCCGCCCCATGGCTGACTAATTTTTTTTATTTATGCAGAGGCCGAGGCCGCCT
CTGCCTCTGAGCTATTCCAGAAGTAGTGAGGAGGCTTTTTTGGAGGCCTAGGCTTTTGCAAAAAGCTCCCG
GGAGCTTGTATATCCATTTTCGGATCTGATCAGCACGTGTTGACAATTAATCATCGGCATAGTATATCGGCATA
GTATAATACGACAAGGTGAGGAACTAAACCATGGCCAAGCCTTTGTCTCAAGAAGAATCCACCCTCATTGAA
AGAGCAACGGCTACAATCAACAGCATCCCCATCTCTGAAGACTACAGCGTCGCCAGCGCAGCTCTCTCTAG
CGACGGCCGCATCTTCACTGGTGTCAATGTATATCATTTTACTGGGGGACCTTGTGCAGAACTCGTGGTGC
TGGGCACTGCTGCTGCTGCGGCAGCTGGCAACCTGACTTGTATCGTCGCGATCGGAAATGAGAACAGGG
GCATCTTGAGCCCCTGCGGACGGTGCCGACAGGTGCTTCTCGATCTGCATCCTGGGATCAAAGCCATAGT
GAAGGACAGTGATGGACAGCCGACGGCAGTTGGGATTCGTGAATTGCTGCCCTCTGGTTATGTGTGGGAG
GGCTAAGCACTTCGTGGCCGAGGAGCAGGACTGACACGTGCTACGAGATTTCGATTCCACCGCCGCCTTC
TATGAAAGGTTGGGCTTCGGAATCGTTTTCCGGGACGCCGGCTGGATGATCCTCCAGCGCGGGGATCTCA
TGCTGGAGTTCTTCGCCCACCCCAACTTGTTTATTGCAGCTTATAATGGTTACAAATAAAGCAATAGCATCAC
AAATTTCACAAATAAAGCATTTTTTTCACTGCATTCTAGTTGTGGTTTGTCCAAACTCATCAATGTATCTTATCA
TGTCTGTATACCGTCGACCTCTAGCTAGAGCTTGGCGTAATCATGGTCATAGCTGTTTCCTGTGTGAAATTGT
TATCCGCTCACAATTCCACACAACATACGAGCCGGAAGCATAAAGTGTAAAGCCTGGGGTGCCTAATGAGT
GAGCTAACTCACATTAATTGCGTTGCGCTCACTGCCCGCTTTCCAGTCGGGAAACCTGTCGTGCCAGCTGC
ATTAATGAATCGGCCAACGCGCGGGGAGAGGCGGTTTGCGTATTGGGCGCTCTTCCGCTTCCTCGCTCAC
TGACTCGCTGCGCTCGGTCGTTCGGCTGCGGCGAGCGGTATCAGCTCACTCAAAGGCGGTAATACGGTTA
TCCACAGAATCAGGGGATAACGCAGGAAAGAACATGTGAGCAAAAGGCCAGCAAAAGGCCAGGAACCGTA
AAAAGGCCGCGTTGCTGGCGTTTTTCCATAGGCTCCGCCCCCCTGACGAGCATCACAAAAATCGACGCTC
AAGTCAGAGGTGGCGAAACCCGACAGGACTATAAAGATACCAGGCGTTTCCCCCTGGAAGCTCCCTCGTG
CGCTCTCCTGTTCCGACCCTGCCGCTTACCGGATACCTGTCCGCCTTTCTCCCTTCGGGAAGCGTGGCGC
TTTCTCATAGCTCACGCTGTAGGTATCTCAGTTCGGTGTAGGTCGTTCGCTCCAAGCTGGGCTGTGTGCAC
GAACCCCCCGTTCAGCCCGACCGCTGCGCCTTATCCGGTAACTATCGTCTTGAGTCCAACCCGGTAAGAC
ACGACTTATCGCCACTGGCAGCAGCCACTGGTAACAGGATTAGCAGAGCGAGGTATGTAGGCGGTGCTAC
AGAGTTCTTGAAGTGGTGGCCTAACTACGGCTACACTAGAAGAACAGTATTTGGTATCTGCGCTCTGCTGAA
GCCAGTTACCTTCGGAAAAAGAGTTGGTAGCTCTTGATCCGGCAAACAAACCACCGCTGGTAGCGGTGGT
TTTTTTGTTTGCAAGCAGCAGATTACGCGCAGAAAAAAAGGATCTCAAGAAGATCCTTTGATCTTTTCTACG
GGGTCTGACGCTCAGTGGAACGAAAACTCACGTTAAGGGATTTTGGTCATGAGATTATCAAAAAGGATCTTC
ACCTAGATCCTTTTAAATTAAAAATGAAGTTTTAAATCAATCTAAAGTATATATGAGTAAACTTGGTCTGACAGT
TACCAATGCTTAATCAGTGAGGCACCTATCTCAGCGATCTGTCTATTTCGTTCATCCATAGTTGCCTGACTCC
CCGTCGTGTAGATAACTACGATACGGGAGGGCTTACCATCTGGCCCCAGTGCTGCAATGATACCGCGAGAC
CCACGCTCACCGGCTCCAGATTTATCAGCAATAAACCAGCCAGCCGGAAGGGCCGAGCGCAGAAGTGGTC
CTGCAACTTTATCCGCCTCCATCCAGTCTATTAATTGTTGCCGGGAAGCTAGAGTAAGTAGTTCGCCAGTTA
ATAGTTTGCGCAACGTTGTTGCCATTGCTACAGGCATCGTGGTGTCACGCTCGTCGTTTGGTATGGCTTCAT
TCAGCTCCGGTTCCCAACGATCAAGGCGAGTTACATGATCCCCCATGTTGTGCAAAAAAGCGGTTAGCTCC
TTCGGTCCTCCGATCGTTGTCAGAAGTAAGTTGGCCGCAGTGTTATCACTCATGGTTATGGCAGCACTGCAT
AATTCTCTTACTGTCATGCCATCCGTAAGATGCTTTTCTGTGACTGGTGAGTACTCAACCAAGTCATTCTGAG
AATAGTGTATGCGGCGACCGAGTTGCTCTTGCCCGGCGTCAATACGGGATAATACCGCGCCACATAGCAGA
ACTTTAAAAGTGCTCATCATTGGAAAACGTTCTTCGGGGCGAAAACTCTCAAGGATCTTACCGCTGTTGAGA
TCCAGTTCGATGTAACCCACTCGTGCA

Seq_mGGA1-delHinge_GFP

CAACTGATCTTCAGCATCTTTTACTTTCACCAGCGTTTCTGGGTGAGCAAAAACAGGAAGGCAAAATGCCG
CAAAAAAGGGAATAAGGGCGACACGGAAATGTTGAATACTCATACTCTTCCTTTTTCAATATTATTGAAGCATT
TATCAGGGTTATTGTCTCATGAGCGGATACATATTTGAATGTATTTAGAAAAATAAACAAATAGGGGTTCCGCG

CACATTTCCCCGAAAAGTGCCACCTGACGTCGACGGATCGGGAGATCTCCCGATCCCCTATGGTGCACTCT
CAGTACAATCTGCTCTGATGCCGCATAGTTAAGCCAGTATCTGCTCCCTGCTTGTGTGTTGGAGGTCGCTG
AGTAGTGCGCGAGCAAAATTTAAGCTACAACAAGGCAAGGCTTGACCGACAATTGCATGAAGAATCTGCTTA
GGGTTAGGCGTTTTGCGCTGCTTCGCGATGTACGGGCCAGATATACGCGTTGACATTGATTATTGACTAGTT
ATTAATAGTAATCAATTACGGGGTCATTAGTTCATAGCCCATATATGGAGTTCCGCGTTACATAACTTACGGTA
AATGGCCCGCCTGGCTGACCGCCCAACGACCCCCGCCCATTGACGTCAATAATGACGTATGTTCCCATAGT
AACGCCAATAGGGACTTTCCATTGACGTCAATGGGTGGAGTATTTACGGTAAACTGCCCACTTGGCAGTACA
TCAAGTGTATCATATGCCAAGTACGCCCCCTATTGACGTCAATGACGGTAAATGGCCCGCCTGGCATTATGC
CCAGTACATGACCTTATGGGACTTTCCTACTTGGCAGTACATCTACGTATTAGTCATCGCTATTACCATGGTG
ATGCGGTTTTGGCAGTACATCAATGGGCGTGGATAGCGGTTTGACTCACGGGGATTTCCAAGTCTCCACCC
CATTGACGTCAATGGGAGTTTGTTTTGGCACCAAAATCAACGGGACTTTCCAAAATGTCGTAACAACTCCGC
CCCATTGACGCAAATGGGCGGTAGGCGTGTACGGTGGGAGGTCTATATAAGCAGAGCTCTCTGGCTAACTA
GAGAACCCACTGCTTACTGGCTTATCGAAATTAATACGACTCACTATAGGGAGACCCAAGCTGGCTAGTTAA
GCTGAGCATCAACAAGTTTGTACAAAAAAGCAGGCTCCGAATTCGCCCTTGCCGCCATGGAGCCCGCGAT
GGAGCCGGAGACTCTGGAGGCACGAATCAACAGAGCCACAAATCCCCTGAACAAGGAGCTGAACTGGGC
CAGCATCAACAGTTTCTGCGAGCAGCTCAACGAAGACTTTGAGGGGCCTCCACTTGCCACTCGCTTGCTG
GCCCACAAGATCCAGTCCCCACAGGAATGGGAAGCCATCCAGGCCTTGACGGTTCTGGAGACGTGCATGA
AGAGCTGCGGCAAGAGGTTCCATGATGAGGTGGGCAAGTTCCGCTTCCTCAACGAGCTCATCAAGGTTGT
GTCTCCCAAGTACTTGGGCTCCCGGACATCTGAGAAGGTGAAGAGTAAGATCTTGGAGCTGCTGTACAGCT
GGACGGTTTGCCTGCCTGAGGAGGTGAAGATTGCAGAAGCCTACCAGATGCTGAAGAAGCAGGGGATTGT
GAAGTCGGACCCCAAGCTTCCAGAGGATGCCATCTTTCCCCTCCCCCCTCCCCGGCCCAAGAATGTGATC
TTTGAAGATGAGGAGAAGTCCAAGATGCTGGCCCGCCTGCTGAAGAGCTCACACCCTGAGGACCTCCGG
GCTGCCAATAAGCTCATCAAAGAGATGGTGCAGGAGGACCAGAAGCGGATGGAAAAGATCTCCAAGCGG
TGAATGCCATCGAGGAGGTCAACAACAATGTGAAGTTGCTGACAGAGATGGTGATGAGCCACAGCCAGGG
TGCTGCATCCAGCAGCAGTGAGGACCTCATGAAGGAACTGTACCAGCGCTGTGAGCGCATGCGACCCACA
CTCTTCCGACTGGCCAGTGACACGGAAGACAATGATGAGGCCTTAGCTGAGATCCTGCAGGCTAATGACAA
TCTCACCCAGGTGATCAACCTGTACAAGCAGCTGGTCCGGAGCAGCATCTTGCCAGTGACCGTTTATGACC
AGCATGGCTTCCGTGTCCTCTTCCATTTTGCTCGGGACCCACTGCCAGGGCGCTCCGATGTGCTGGTGGT
GGTGGTCTCTATGCTGAGCACGGCGCCCCAGCCCATCCGGAACATCGTTTTCCAGTCAGCCGTCCCCAAG
GTCATGAAGGTGAGGCTGCAGCCACCTTCCGGCACAGAGCTGCCAGCGTTCAACCCCATCGTCCACCCCT
CAGCCATCACCCAGGTCCTGCTCCTTGCTAACCCCCAGAAGGAGAAGGTTCGCCTCCGCTACAAGCTCAT
CTTCACTATGGGCGACCAGACCTACAATGAGATGGGAGATGTGGATCAGTTCCCCCCACCAGAGACCTGG
GGGAGCCTCAAGGGCGAATTCGACCCAGCTTTCTTGTACAAAGTGGTTGATGCTGTTAACATGGTGAGCAA
GGGCGAGGAGCTGTTCACCGGGGTGGTGCCCATCCTGGTCGAGCTGGACGGCGACGTAAACGGCCACA
AGTTCAGCGTGTCCGGCGAGGGCGAGGGCGATGCCACCTACGGCAAGCTGACCCTGAAGTTCATCTGCA
CCACCGGCAAGCTGCCCGTGCCCTGGCCCACCCTCGTGACCACCTTCACCTACGGCGTGCAGTGCTTCG
CCCGCTACCCCGACCACATGAAGCAGCACGACTTCTTCAAGTCCGCCATGCCCGAAGGCTACGTCCAGGA
GCGCACCATCTTCTTCAAGGACGACGGCAACTACAAGACCCGCGCCGAGGTGAAGTTCGAGGGCGACAC
CCTGGTGAACCGCATCGAGCTGAAGGGCATCGACTTCAAGGAGGACGGCAACATCCTGGGGCACAAGCT
GGAGTACAACTACAACAGCCACAAGGTCTATATCACCGCCGACAAGCAGAAGAACGGCATCAAGGTGAACT
TCAAGACCCGCCACAACATCGAGGACGGCAGCGTGCAGCTCGCCGACCACTACCAGCAGAACACCCCCA
TCGGCGACGGCCCCGTGCTGCTGCCCGACAACCACTACCTGAGCACCCAGTCCGCCCTGAGCAAAGACC
CCAACGAGAAGCGCGATCACATGGTCCTGCTGGAGTTCGTGACCGCCGCCGGGATCACTCTCGGCATGG
ACGAGCTGTACAAGTAATGATAAGTTTAAACGGGGGAGGCTAACTGAAACACGGAAGGAGACAATACCGGA
AGGAACCCGCGCTATGACGGCAATAAAAAGACAGAATAAAACGCACGGGTGTTGGGTCGTTTGTTCATAAA
CGCGGGGTTCGGTCCCAGGGCTGGCACTCTGTCGATACCCCACCGAGACCCCATTGGGGCCAATACGCC
CGCGTTTCTTCCTTTTCCCCACCCCACCCCCCAAGTTCGGGTGAAGGCCCAGGGCTCGCAGCCAACGTC
GGGGCGGCAGGCCCTGCCATAGCAGATCTGCGCAGCTGGGGCTCTAGGGGGTATCCCCACGCGCCCTGT

AGCGGCGCATTAAGCGCGGCGGGTGTGGTGGTTACGCGCAGCGTGACCGCTACACTTGCCAGCGCCCTA
GCGCCCGCTCCTTTCGCTTTCTTCCCTTCCTTTCTCGCCACGTTCGCCGGCTTTCCCCGTCAAGCTCTAAA
TCGGGGGCTCCCTTTAGGGTTCCGATTTAGTGCTTTACGGCACCTCGACCCCAAAAAACTTGATTAGGGTG
ATGGTTCACGTAGTGGGCCATCGCCCTGATAGACGGTTTTTCGCCCTTTGACGTTGGAGTCCACGTTCTTT
AATAGTGGACTCTTGTTCCAAACTGGAACAACACTCAACCCTATCTCGGTCTATTCTTTTGATTTATAAGGGA
TTTTGCCGATTTCGGCCTATTGGTTAAAAAATGAGCTGATTTAACAAAAATTTAACGCGAATTAATTCTGTGGA
ATGTGTGTCAGTTAGGGTGTGGAAAGTCCCCAGGCTCCCCAGCAGGCAGAAGTATGCAAAGCATGCATCT
CAATTAGTCAGCAACCAGGTGTGGAAAGTCCCCAGGCTCCCCAGCAGGCAGAAGTATGCAAAGCATGCAT
CTCAATTAGTCAGCAACCATAGTCCCGCCCCTAACTCCGCCCATCCCGCCCCTAACTCCGCCCAGTTCCGC
CCATTCTCCGCCCCATGGCTGACTAATTTTTTTTATTTATGCAGAGGCCGAGGCCGCCTCTGCCTCTGAGCT
ATTCCAGAAGTAGTGAGGAGGCTTTTTTGGAGGCCTAGGCTTTTGCAAAAAGCTCCCGGGAGCTTGTATAT
CCATTTTCGGATCTGATCAGCACGTGTTGACAATTAATCATCGGCATAGTATATCGGCATAGTATAATACGACA
AGGTGAGGAACTAAACCATGGCCAAGCCTTTGTCTCAAGAAGAATCCACCCTCATTGAAAGAGCAACGGCT
ACAATCAACAGCATCCCCATCTCTGAAGACTACAGCGTCGCCAGCGCAGCTCTCTCTAGCGACGGCCGCA
TCTTCACTGGTGTCAATGTATATCATTTTACTGGGGGACCTTGTGCAGAACTCGTGGTGCTGGGCACTGCT
GCTGCTGCGGCAGCTGGCAACCTGACTTGTATCGTCGCGATCGGAAATGAGAACAGGGGCATCTTGAGCC
CCTGCGGACGGTGCCGACAGGTGCTTCTCGATCTGCATCCTGGGATCAAAGCCATAGTGAAGGACAGTGA
TGGACAGCCGACGGCAGTTGGGATTCGTGAATTGCTGCCCTCTGGTTATGTGTGGGAGGGCTAAGCACTT
CGTGGCCGAGGAGCAGGACTGACACGTGCTACGAGATTTCGATTCCACCGCCGCCTTCTATGAAAGGTTG
GGCTTCGGAATCGTTTTCCGGGACGCCGGCTGGATGATCCTCCAGCGCGGGGATCTCATGCTGGAGTTCT
TCGCCCACCCCAACTTGTTTATTGCAGCTTATAATGGTTACAAATAAAGCAATAGCATCACAAATTTCACAAAT
AAAGCATTTTTTTCACTGCATTCTAGTTGTGGTTTGTCCAAACTCATCAATGTATCTTATCATGTCTGTATACC
GTCGACCTCTAGCTAGAGCTTGGCGTAATCATGGTCATAGCTGTTTCCTGTGTGAAATTGTTATCCGCTCAC
AATTCCACACAACATACGAGCCGGAAGCATAAAGTGTAAAGCCTGGGGTGCCTAATGAGTGAGCTAACTCA
CATTAATTGCGTTGCGCTCACTGCCCGCTTTCCAGTCGGGAAACCTGTCGTGCCAGCTGCATTAATGAATC
GGCCAACGCGCGGGGAGAGGCGGTTTGCGTATTGGGCGCTCTTCCGCTTCCTCGCTCACTGACTCGCTG
CGCTCGGTCGTTCGGCTGCGGCGAGCGGTATCAGCTCACTCAAAGGCGGTAATACGGTTATCCACAGAAT
CAGGGGATAACGCAGGAAAGAACATGTGAGCAAAAGGCCAGCAAAAGGCCAGGAACCGTAAAAAGGCCG
CGTTGCTGGCGTTTTTCCATAGGCTCCGCCCCCCTGACGAGCATCACAAAAATCGACGCTCAAGTCAGAG
GTGGCGAAACCCGACAGGACTATAAAGATACCAGGCGTTTCCCCCTGGAAGCTCCCTCGTGCGCTCTCCT
GTTCCGACCCTGCCGCTTACCGGATACCTGTCCGCCTTTCTCCCTTCGGGAAGCGTGGCGCTTTCTCATAG
CTCACGCTGTAGGTATCTCAGTTCGGTGTAGGTCGTTCGCTCCAAGCTGGGCTGTGTGCACGAACCCCCC
GTTCAGCCCGACCGCTGCGCCTTATCCGGTAACTATCGTCTTGAGTCCAACCCGGTAAGACACGACTTATC
GCCACTGGCAGCAGCCACTGGTAACAGGATTAGCAGAGCGAGGTATGTAGGCGGTGCTACAGAGTTCTTG
AAGTGGTGGCCTAACTACGGCTACACTAGAAGAACAGTATTTGGTATCTGCGCTCTGCTGAAGCCAGTTAC
CTTCGGAAAAAGAGTTGGTAGCTCTTGATCCGGCAAACAAACCACCGCTGGTAGCGGTGGTTTTTTTGTTT
GCAAGCAGCAGATTACGCGCAGAAAAAAAGGATCTCAAGAAGATCCTTTGATCTTTTCTACGGGGTCTGAC
GCTCAGTGGAACGAAAACTCACGTTAAGGGATTTTGGTCATGAGATTATCAAAAAGGATCTTCACCTAGATC
CTTTTAAATTAAAAATGAAGTTTTAAATCAATCTAAAGTATATATGAGTAAACTTGGTCTGACAGTTACCAATGC
TTAATCAGTGAGGCACCTATCTCAGCGATCTGTCTATTTCGTTCATCCATAGTTGCCTGACTCCCCGTCGTGT
AGATAACTACGATACGGGAGGGCTTACCATCTGGCCCCAGTGCTGCAATGATACCGCGAGACCCACGCTCA
CCGGCTCCAGATTTATCAGCAATAAACCAGCCAGCCGGAAGGGCCGAGCGCAGAAGTGGTCCTGCAACTT
TATCCGCCTCCATCCAGTCTATTAATTGTTGCCGGGAAGCTAGAGTAAGTAGTTCGCCAGTTAATAGTTTGCG
CAACGTTGTTGCCATTGCTACAGGCATCGTGGTGTCACGCTCGTCGTTTGGTATGGCTTCATTCAGCTCCG
GTTCCCAACGATCAAGGCGAGTTACATGATCCCCCATGTTGTGCAAAAAAGCGGTTAGCTCCTTCGGTCCT
CCGATCGTTGTCAGAAGTAAGTTGGCCGCAGTGTTATCACTCATGGTTATGGCAGCACTGCATAATTCTCTT
ACTGTCATGCCATCCGTAAGATGCTTTTCTGTGACTGGTGAGTACTCAACCAAGTCATTCTGAGAATAGTGT
ATGCGGCGACCGAGTTGCTCTTGCCCGGCGTCAATACGGGATAATACCGCGCCACATAGCAGAACTTTAAA

AGTGCTCATCATTGGAAAACGTTCTTCGGGGCGAAAACTCTCAAGGATCTTACCGCTGTTGAGATCCAGTT
CGATGTAACCCACTCGTGCACC

Seq_c-Myc-mGGA1-FL

CTTTTATTTCAGGTCCCGGATCCGGTGGTGGTGCAAATCAAAGAACTGCTCCTCAGTGGATGTTGCCTTTAC
TTCTAGGCCTGTACGGAAGTGTTACTTCTGCTCTAAAAGCTGCGGAATTGTACCCGCGGGCCCACCATGGC
ATCAATGCAGAAGCTGATCTCAGAGGAGGACCTGCTTATGGCCATGGAGGCCCGAATTCCGATGGAGCCC
GCGATGGAGCCGGAGACTCTGGAGGCACGAATCAACAGAGCCACAAATCCCCTGAACAAGGAGCTGAAC
TGGGCCAGCATCAACAGTTTCTGCGAGCAGCTCAACGAAGACTTTGAGGGGCCTCCACTTGCCACTCGCT
TGCTGGCCCACAAGATCCAGTCCCCACAGGAATGGGAAGCCATCCAGGCCTTGACGGTTCTGGAGACGT
GCATGAAGAGCTGCGGCAAGAGGTTCCATGATGAGGTGGGCAAGTTCCGCTTCCTCAACGAGCTCATCAA
GGTTGTGTCTCCCAAGTACTTGGGCTCCCGGACATCTGAGAAGGTGAAGAGTAAGATCTTGGAGCTGCTG
TACAGCTGGACGGTTTGCCTGCCTGAGGAGGTGAAGATTGCAGAAGCCTACCAGATGCTGAAGAAGCAGG
GGATTGTGAAGTCGGACCCCAAGCTTCCAGAGGATGCCATCTTTCCCCTCCCCCCTCCCCGGCCCAAGAA
TGTGATCTTTGAAGATGAGGAGAAGTCCAAGATGCTGGCCCGCCTGCTGAAGAGCTCACACCCTGAGGAC
CTCCGGGCTGCCAATAAGCTCATCAAAGAGATGGTGCAGGAGGACCAGAAGCGGATGGAAAAGATCTCCA
AGCGGGTGAATGCCATCGAGGAGGTCAACAACAATGTGAAGTTGCTGACAGAGATGGTGATGAGCCACAG
CCAGGGTGCTGCATCCAGCAGCAGTGAGGACCTCATGAAGGAACTGTACCAGCGCTGTGAGCGCATGCG
ACCCACACTCTTCCGACTGGCCAGTGACACGGAAGACAATGATGAGGCCTTAGCTGAGATCCTGCAGGCT
AATGACAATCTCACCCAGGTGATCAACCTGTACAAGCAGCTGGTCCGGGGCGAGGAGGTCAACGGTGATG
CCACAGCCAGCTCCATTCCTGGAAGCACGTCAGCCCTGCTGGACCTCTCAGGCCTGGACCTCCCTCCCC
CGGGCACCACCCAGCCAGCCACGCCCACCCGCCCTGGCAACCAGAGCAGTCCTGAGCAGCTCAGTGCC
TCGGTGTCCCTGCTTGATGACGAGCTCATGTCTCTGGGCCTAAGTGACCCGACACCACCTTCAGGCACCA
GCTCAGATAGTGTGGGGTGGGACAACTTCCAGTCATCAGATGGCACTGAATCCTCAGTCCCTCCTCCAGC
CCAGGCCCCCAGCATGGACTGCCGACCCCCAGCCCAGGCCCCTCCACCAACGAGCAGTGGCCTGGACG
ACCTGGACCTCTTGGGGAAAACCCTTATGCAGCAGGCTCTGCCTCCGGAAGCCCAGCAAGTGCGGTGGG
AGAAGCAGCAGCCAGCCCCCCGGCTCACCCTCCGTGACCTGCAGAGTAAGAGCAGCTCGCCCAGCCCAG
GAGCCGCCAGCCTCCTCCACACCACGTCCCCAGAGCCCCCTGGGCCTCCACCTCAGGCCACACCCACTG
AGTTCTCCCTAACCAGCATCACTGTGCCCCTGGAGTCTATCAAACCCAGCAGCATCTTGCCAGTGACCGTT
TATGACCAGCATGGCTTCCGTGTCCTCTTCCATTTTGCTCGGGACCCACTGCCAGGGCGCTCCGATGTGCT
GGTGGTGGTGGTCTCTATGCTGAGCACGGCGCCCCAGCCCATCCGGAACATCGTTTTCCAGTCAGCCGTC
CCCAAGGTCATGAAGGTGAGGCTGCAGCCACCTTCCGGCACAGAGCTGCCAGCGTTCAACCCCATCGTC
CACCCCTCAGCCATCACCCAGGTCCTGCTCCTTGCTAACCCCCAGAAGGAGAAGGTTCGCCTCCGCTACA
AGCTCATCTTCACTATGGGCGACCAGACCTACAATGAGATGGGAGATGTGGATCAGTTCCCCCCACCAGAG
ACCTGGGGGAGCCTCTAGCTCGAGGTACCGCGGCCGCGGGGATCCAGACATGATAAGATACATTGATGAG
TTTGGACAAACCACAACTAGAATGCAGTGAAAAAAATGCTTTATTTGTGAAATTTGTGATGCTATTGCTTTATT
TGTAACCATTATAAGCTGCAATAAACAAGTTAACAACAACAATTGCATTCATTTTATGTTTCAGGTTCAGGGGG
AGGTGTGGGAGGTTTTTTCGGATCCTCTAGAGTCGATCTGCAGGCATGCTAGCTTGGCGTAATCATGGTCA
TAGCTGTTTCCTGTGTGAAATTGTTATCCGCTCACAATTCCACACAACATACGAGCCGGAAGCATAAAGTGT
AAAGCCTGGGGTGCCTAATGAGTGAGCTAACTCACATTAATTGCGTTGCGCTCACTGCCCGCTTTCCAGTC
GGGAAACCTGTCGTGCCAGCTGCATTAATGAATCGGCCAACGCGCGGGGAGAGGCGGTTTGCGTATTGG
GCGCTCTTCCGCTTCCTCGCTCACTGACTCGCTGCGCTCGGTCGTTCGGCTGCGGCGAGCGGTATCAGC
TCACTCAAAGGCGGTAATACGGTTATCCACAGAATCAGGGGATAACGCAGGAAAGAACATGTGAGCAAAAG
GCCAGCAAAAGGCCAGGAACCGTAAAAAGGCCGCGTTGCTGGCGTTTTTCCATAGGCTCCGCCCCCCTGA

CGAGCATCACAAAAATCGACGCTCAAGTCAGAGGTGGCGAAACCCGACAGGACTATAAAGATACCAGGCG
TTTCCCCCTGGAAGCTCCCTCGTGCGCTCTCCTGTTCCGACCCTGCCGCTTACCGGATACCTGTCCGCCT
TTCTCCCTTCGGGAAGCGTGGCGCTTTCTCATAGCTCACGCTGTAGGTATCTCAGTTCGGTGTAGGTCGTT
CGCTCCAAGCTGGGCTGTGTGCACGAACCCCCCGTTCAGCCCGACCGCTGCGCCTTATCCGGTAACTATC
GTCTTGAGTCCAACCCGGTAAGACACGACTTATCGCCACTGGCAGCAGCCACTGGTAACAGGATTAGCAG
AGCGAGGTATGTAGGCGGTGCTACAGAGTTCTTGAAGTGGTGGCCTAACTACGGCTACACTAGAAGAACAG
TATTTGGTATCTGCGCTCTGCTGAAGCCAGTTACCTTCGGAAAAAGAGTTGGTAGCTCTTGATCCGGCAAAC
AAACCACCGCTGGTAGCGGTGGTTTTTTTGTTTGCAAGCAGCAGATTACGCGCAGAAAAAAAGGATCTCAA
GAAGATCCTTTGATCTTTTCTACGGGGTCTGACGCTCAGTGGAACGAAAACTCACGTTAAGGGATTTTGGT
CATGAGATTATCAAAAAGGATCTTCACCTAGATCCTTTTAAATTAAAAATGAAGTTTTAAATCAATCTAAAGTAT
ATATGAGTAAACTTGGTCTGACAGTTACCAATGCTTAATCAGTGAGGCACCTATCTCAGCGATCTGTCTATTT
CGTTCATCCATAGTTGCCTGACTCCCCGTCGTGTAGATAACTACGATACGGGAGGGCTTACCATCTGGCCC
CAGTGCTGCAATGATACCGCGAGACCCACGCTCACCGGCTCCAGATTTATCAGCAATAAACCAGCCAGCCG
GAAGGGCCGAGCGCAGAAGTGGTCCTGCAACTTTATCCGCCTCCATCCAGTCTATTAATTGTTGCCGGGAA
GCTAGAGTAAGTAGTTCGCCAGTTAATAGTTTGCGCAACGTTGTTGCCATTGCTACAGGCATCGTGGTGTCA
CGCTCGTCGTTTGGTATGGCTTCATTCAGCTCCGGTTCCCAACGATCAAGGCGAGTTACATGATCCCCCAT
GTTGTGCAAAAAAGCGGTTAGCTCCTTCGGTCCTCCGATCGTTGTCAGAAGTAAGTTGGCCGCAGTGTTAT
CACTCATGGTTATGGCAGCACTGCATAATTCTCTTACTGTCATGCCATCCGTAAGATGCTTTTCTGTGACTGG
TGAGTACTCAACCAAGTCATTCTGAGAATAGTGTATGCGGCGACCGAGTTGCTCTTGCCCGGCGTCAATAC
GGGATAATACCGCGCCACATAGCAGAACTTTAAAAGTGCTCATCATTGGAAAACGTTCTTCGGGGCGAAAA
CTCTCAAGGATCTTACCGCTGTTGAGATCCAGTTCGATGTAACCCACTCGTGCACCCAACTGATCTTCAGCA
TCTTTTACTTTCACCAGCGTTTCTGGGTGAGCAAAAACAGGAAGGCAAAATGCCGCAAAAAAGGGAATAAG
GGCGACACGGAAATGTTGAATACTCATACTCTTCCTTTTTCAATATTATTGAAGCATTTATCAGGGTTATTGTC
TCATGAGCGGATACATATTTGAATGTATTTAGAAAAATAAACAAATAGGGGTTCCGCGCACATTTCCCCGAAA
AGTGCCACCTGACGTCTAAGAAACCATTATTATCATGACATTAACCTATAAAAATAGGCGTATCACGAGGCCC
TTTCGTCTCGCGCGTTTCGGTGATGACGGTGAAAACCTCTGACACATGCAGCTCCCGGAGACGGTCACAG
CTTGTCTGTAAGCGGATGCCGGGAGCAGACAAGCCCGTCAGGGCGCGTCAGCGGGTGTTGGCGGGTGT
CGGGGCTGGCTTAACTATGCGGCATCAGAGCAGATTGTACTGAGAGTGCACCATATGCGGTGTGAAATACC
GCACAGATGCGTAAGGAGAAAATACCGCATCAGGCGCCATTCGCCATTCAGGCTGCGCAACTGTTGGGAA
GGGCGATCGGTGCGGGCCTCTTCGCTATTACGCCAGCTGGCGAAAGGGGGATGTGCTGCAAGGCGATTAA
GTTGGGTAACGCCAGGGTTTTCCCAGTCACGACGTTGTAAAACGACGGCCAGTGAGTTCGAGCTTGCATG
CCTGCAGGTCGTTACATAACTTACGGTAAATGGCCCGCCTGGCTGACCGCCCAACGACCCCCGCCCATTG
ACGTCAATAATGACGTATGTTCCCATAGTAACGCCAATAGGGACTTTCCATTGACGTCAATGGGTGGAGTATT
TACGGTAAACTGCCCACTTGGCAGTACATCAAGTGTATCATATGCCAAGTACGCCCCCTATTGACGTCAATG
ACGGTAAATGGCCCGCCTGGCATTATGCCCAGTACATGACCTTATGGGACTTTCCTACTTGGCAGTACATCT
ACGTATTAGTCATCGCTATTACCATGGTGATGCGGTTTTGGCAGTACATCAATGGGCGTGGATAGCGGTTTG
ACTCACGGGGATTTCCAAGTCTCCACCCCATTGACGTCAATGGGAGTTTGTTTTGGCACCAAAATCAACGG
GACTTTCCAAAATGTCGTAACAACTCCGCCCCATTGACGCAAATGGGCGGTAGGCGTGTACGGTGGGAGG
TCTATATAAGCAGAGCTCGTTTAGTGAACCGTCAGATCGCCTGGAGACGCCATCCACGCTGTTTTGACCTC
CATAGAAGACACCGGGACCGATCCAGCCTCCGGACTCTAGAGGATCCGGTACTAGAGGAACTGAAAAACC
AGAAAGTTAACTGGTAAGTTTAGTCTTTTTGT

Seq_mGGA1-FL

GCTGGTTTATTGCTGATAAATCTGGAGCCGGTGAGCGTGGGTCTCGCGGTATCATTGCAGCACTGGGGCC
AGATGGTAAGCCCTCCCGTATCGTAGTTATCTACACGACGGGGAGTCAGGCAACTATGGATGAACGAAATAG
ACAGATCGCTGAGATAGGTGCCTCACTGATTAAGCATTGGTAACTGTCAGACCAAGTTTACTCATATATACTT

TAGATTGATTTAAAACTTCATTTTTAATTTAAAAGGATCTAGGTGAAGATCCTTTTTGATAATCTCATGCCATAA
CTTCGTATAATGTATGCTATACGAAGTTATGGCATGACCAAAATCCCTTAACGTGAGTTTTCGTTCCACTGAG
CGTCAGACCCCGTAGAAAAGATCAAAGGATCTTCTTGAGATCCTTTTTTTCTGCGCGTAATCTGCTGCTTGC
AAACAAAAAAACCACCGCTACCAGCGGTGGTTTGTTTGCCGGATCAAGAGCTACCAACTCTTTTTCCGAAG
GTAACTGGCTTCAGCAGAGCGCAGATACCAAATACTGTTCTTCTAGTGTAGCCGTAGTTAGGCCACCACTTC
AAGAACTCTGTAGCACCGCCTACATACCTCGCTCTGCTAATCCTGTTACCAGTGGCTGCTGCCAGTGGCGA
TAAGTCGTGTCTTACCGGGTTGGACTCAAGACGATAGTTACCGGATAAGGCGCAGCGGTCGGGCTGAACG
GGGGGTTCGTGCACACAGCCCAGCTTGGAGCGAACGACCTACACCGAACTGAGATACCTACAGCGTGAG
CTATGAGAAAGCGCCACGCTTCCCGAAGGGAGAAAGGCGGACAGGTATCCGGTAAGCGGCAGGGTCGGA
ACAGGAGAGCGCACGAGGGAGCTTCCAGGGGGAAACGCCTGGTATCTTTATAGTCCTGTCGGGTTTCGCC
ACCTCTGACTTGAGCGTCGATTTTTGTGATGCTCGTCAGGGGGGCGGAGCCTATGGAAAAACGCCAGCAA
CGCGGCCTTTTTACGGTTCCTGGCCTTTTGCTGGCCTTTTGCTCACATGTTCTTTCCTGCGTTATCCCCTGA
TTCTGTGGATAACCGTATTACCGCCTTTGAGTGAGCTGATACCGCTCGCCGCAGCCGAACGACCGAGCGC
AGCGAGTCAGTGAGCGAGGAAGCGGAAGAGCGCCCAATACGCAAACCGCCTCTCCCCGCGCGTTGGCC
GATTCATTAATGCAGAGCTTGCAATTCGCGCTTTTTCAATATTATTGAAGCATTTATCAGGGTTATTGTCTCAT
GAGCGGATACATATTTGAATGTATTTAGAAAAATAAACAAATAGGGGTTCCGCGCACATTTCCCCGAAAAGTG
CCACCTGACGTCTAAGAAACCATTATTATCATGACATTAACCTATAAAAATAGGCGTATTACGAGGCCCTTTCA
CGCATTAGATGCATGTCGTTACATAACTTACGGTAAATGGCCCGCCTGGCTGACCGCCCAACGACCCCCGC
CCATTGACGTCAATAATGACGTATGTTCCCATAGTAACGCCAATAGGGACTTTCCATTGACGTCAATGGGTG
GAGTATTTACGGTAAACTGCCCACTTGGCAGTACATCAAGTGTATCATATGCCAAGTACGCCCCCTATTGAC
GTCAATGACGGTAAATGGCCCGCCTGGCATTATGCCCAGTACATGACCTTATGGGACTTTCCTACTTGGCAG
TACATCTACGTATTAGTCATCGCTATTACCATGGTGATGCGGTTTTGGCAGTACATCAATGGGCGTGGATAGC
GGTTTGACTCACGGGGATTTCCAAGTCTCCACCCCATTGACGTCAATGGGAGTTTGTTTTGGCACCAAAAT
CAACGGGACTTTCCAAAATGTCGTAACAACTCCGCCCCATTGACGCAAATGGGCGGTAGGCGTGTACGGT
GGGAGGTCTATATAAGCAGAGCTCGTTTAGTGAACCGTCAGATCGCCTGGAGACGCCATCCACGCTGTTTT
GACCTCCATAGAAGACACCGGGACCGATCCAGCCTCCGGACTCTAGCCTAGGCCGCGGGACGGATAACAA
TTTCACACAGGAAACAGCTATGACCATTAGGCCTATTTAGGTGACACTATAGAACAAGTTTGTACAAAAAAGC
AGGCTGGTACCGGTCCGGAATTCCCGGGATATCGTCGACCCACGCGTCCGGAAACATGGCTCCGGAGCG
TTGTCCCGGAAGCGGTCACGGGTCTCTGCGTGACGCTAACTGAGTTAGAGCGGTCCCGCGGGGTTTCTAT
GGTGCCGCCCGCCACCCCCAGGCTCTCCTCTTCCTCTCCCGCCCGAGCGCCCGGAGCTGCGAGGCCGC
CGACCAGCCCCACCGGCGACTCCGCCAGCCGCCAGGGGGAGGGGCGCCGACGAGGCGACGCTCTAGG
CGGCTCCACCCGCGTCTCGATGGTGCCCGGCGTCCCGGTGCCGCGTTCCTACTCCGCGGGGTGGACGC
GGCCCCTTTAACCGGCCGAGGGCGGAGCCGAGGAGTTCCCGCGAGAGCGGCCGGGGGGCGGGGGGCG
GCGCCGAGGCCGGGGGCCCGTGGCGGATGGAGCCCGCGATGGAGCCGGAGACTCTGGAGGCACGAAT
CAACAGAGCCACAAATCCCCTGAACAAGGAGCTGAACTGGGCCAGCATCAACAGTTTCTGCGAGCAGCTC
AACGAAGACTTTGAGGGGCCTCCACTTGCCACTCGCTTGCTGGCCCACAAGATCCAGTCCCCACAGGAAT
GGGAAGCCATCCAGGCCTTGACGGTTCTGGAGACGTGCATGAAGAGCTGCGGCAAGAGGTTCCATGATGA
GGTGGGCAAGTTCCGCTTCCTCAACGAGCTCATCAAGGTTGTGTCTCCCAAGTACTTGGGCTCCCGGACA
TCTGAGAAGGTGAAGAGTAAGATCTTGGAGCTGCTGTACAGCTGGACGGTTTGCCTGCCTGAGGAGGTGA
AGATTGCAGAAGCCTACCAGATGCTGAAGAAGCAGGGGATTGTGAAGTCGGACCCCAAGCTTCCAGAGGA
TGCCATCTTTCCCCTCCCCCCTCCCCGGCCCAAGAATGTGATCTTTGAAGATGAGGAGAAGTCCAAGATGC
TGGCCCGCCTGCTGAAGAGCTCACACCCTGAGGACCTCCGGGCTGCCAATAAGCTCATCAAAGAGATGGT
GCAGGAGGACCAGAAGCGGATGGAAAAGATCTCCAAGCGGGTGAATGCCATCGAGGAGGTCAACAACAAT
GTGAAGTTGCTGACAGAGATGGTGATGAGCCACAGCCAGGGTGCTGCATCCAGCAGCAGTGAGGACCTC
ATGAAGGAACTGTACCAGCGCTGTGAGCGCATGCGACCCACACTCTTCCGACTGGCCAGTGACACGGAAG
ACAATGATGAGGCCTTAGCTGAGATCCTGCAGGCTAATGACAATCTCACCCAGGTGATCAACCTGTACAAG
CAGCTGGTCCGGGGCGAGGAGGTCAACGGTGATGCCACAGCCAGCTCCATTCCTGGAAGCACGTCAGCC
CTGCTGGACCTCTCAGGCCTGGACCTCCCTCCCCCGGGCACCACCCAGCCAGCCACGCCCACCCGCCCT

GGCAACCAGAGCAGTCCTGAGCAGCTCAGTGCCTCGGTGTCCCTGCTTGATGACGAGCTCATGTCTCTGG
GCCTAAGTGACCCGACACCACCTTCAGGCACCAGCTCAGATAGTGTGGGGTGGGACAACTTCCAGTCATC
AGATGGCACTGAATCCTCAGTCCCTCCTCCAGCCCAGGCCCCCAGCATGGACTGCCGACCCCCAGCCCA
GGCCCCTCCACCAACGAGCAGTGGCCTGGACGACCTGGACCTCTTGGGGAAAACCCTTATGCAGCAGGC
TCTGCCTCCGGAAGCCCAGCAAGTGCGGTGGGAGAAGCAGCAGCCAGCCCCCCGGCTCACCCTCCGTG
ACCTGCAGAGTAAGAGCAGCTCGCCCAGCCCAGGAGCCGCCAGCCTCCTCCACACCACGTCCCCAGAGC
CCCCTGGGCCTCCACCTCAGGCCACACCCACTGAGTTCTCCCTAACCAGCATCACTGTGCCCCTGGAGTC
TATCAAACCCAGCAGCATCTTGCCAGTGACCGTTTATGACCAGCATGGCTTCCGTGTCCTCTTCCATTTTGC
TCGGGACCCACTGCCAGGGCGCTCCGATGTGCTGGTGGTGGTGGTCTCTATGCTGAGCACGGCGCCCCA
GCCCATCCGGAACATCGTTTTCCAGTCAGCCGTCCCCAAGGTCATGAAGGTGAGGCTGCAGCCACCTTCC
GGCACAGAGCTGCCAGCGTTCAACCCCATCGTCCACCCCTCAGCCATCACCCAGGTCCTGCTCCTTGCTA
ACCCCCAGAAGGAGAAGGTTCGCCTCCGCTACAAGCTCATCTTCACTATGGGCGACCAGACCTACAATGA
GATGGGAGATGTGGATCAGTTCCCCCCACCAGAGACCTGGGGGAGCCTCTAGGACAGAGGGGCTGGAGA
GAGGAGGGGCAGCAGGACCTGGACCTGTTCATCCTGTGCTTCTCTCCCTCCTGTGGCCCCCAGTGACTCT
TCCCCCTCCCTCTTCCCCTGCTGAGCCAAACCCAGCAGGAGGCTGCGCCTGGGTCTGCCATAGCTGCTG
GGACCTCCATCACCAGTGGGAGCCTGGAGCAGGGAGGGGCTGTATGCCCTGGAAGGACTCTGGGGTGA
GGGAAGGAGGGGGGCTGTGAGGCCCAGCCCTGAACCCCAGCCTGAGGTGGGGTCGTCCTCACCTGTCT
TTTATGCCTTATGGAAAGGCCCAGCCATAACGTGGAGGCCATGCTGGAAACTGGGACCAGCCCAGGCCTC
CTCTGCGGGGACCCAGTGACTGGGTGGGGGTGACGCCCGCGCCCTAGCTGTGTGCACTTGGTGTGTGGT
CTGGCTCGTTGCTTTTCTTTTGGAATGGCCCTGTGGTCACAGAGCTGAAGGAGCTCTCCACTCAGCCCCC
GGGGCCTCCAGAGGCCTCCCTTTGCTCTCAGGCTCCCACGGGCCATGGAAACTTGAAAGCAGAGCCAGA
GGACATGCCCCCTGGAGGGCCATGGAACTCCCAGAGTTCCATGCTCCATTATCACCTTCTGCTGGGGCCT
CCCGGGAGCACCTCACATCTGCCCACCCTCCTGTGGTCAAGTAGGGTCAGGATGGGTGGCAGTGGGAGT
ATTTATGAAAATAAAGTGACTTTTTTCCTGGAAAAAAAAAAAAAAAAAAAAAAAAAAAAAAAAAAAAAAGGGC
GGCCGCTCTAGAGTATCCCTCGAGGGGCCCAAGCTTACGCGTACCCAGCTTTCTTGTACAAAGTGGTCCCT
ATAGTGAGTCGTATTATAAGCTAGGCACTGGCCGTCGTTTTACAACGTCGTGACTGGGAAAACTGCTAGCTT
GGGATCTTTGTGAAGGAACCTTACTTCTGTGGTGTGACATAATTGGACAAACTACCTACAGAGATTTAAAGC
TCTAAGGTAAATATAAAATTTTTAAGTGTATAATGTGTTAAACTAGCTGCATATGCTTGCTGCTTGAGAGTTTTG
CTTACTGAGTATGATTTATGAAAATATTATACACAGGAGCTAGTGATTCTAATTGTTTGTGTATTTTAGATTCAC
AGTCCCAAGGCTCATTTCAGGCCCCTCAGTCCTCACAGTCTGTTCATGATCATAATCAGCCATACCACATTT
GTAGAGGTTTTACTTGCTTTAAAAAACCTCCCACACCTCCCCCTGAACCTGAAACATAAAATGAATGCAATTG
TTGTTGTTAACTTGTTTATTGCAGCTTATAATGGTTACAAATAAAGCAATAGCATCACAAATTTCACAAATAAAG
CATTTTTTTCACTGCATTCTAGTTGTGGTTTGTCCAAACTCATCAATGTATCTTATCATGTCTGGATCGATCCT
GCATTAATGAATCGGCCAACGCGCGGGGAGAGGCGGTTTGCGTATTGGCTGGCGTAATAGCGAAGAGGCC
CGCACCGATCGCCCTTCCCAACAGTTGCGCAGCCTGAATGGCGAATGGGACGCGCCCTGTAGCGGCGCA
TTAAGCGCGGCGGGTGTGGTGGTTACGCGCAGCGTGACCGCTACACTTGCCAGCGCCCTAGCGCCCGCT
CCTTTCGCTTTCTTCCCTTCCTTTCTCGCCACGTTCGCCGGCTTTCCCCGTCAAGCTCTAAATCGGGGGCT
CCCTTTAGGGTTCCGATTTAGTGCTTTACGGCACCTCGACCCCAAAAAACTTGATTAGGGTGATGGTTCACG
TAGTGGGCCATCGCCCTGATAGACGGTTTTTCGCCCTTTGACGTTGGAGTCCACGTTCTTAATAGTGGACT
CTTGTTCCAAACTGGAACAACACTCAACCCTATCTCGGTCTATTCTTTTGATTTATAAGGGATTTTGCCGATTT
CGGCCTATTGGTTAAAAAATGAGCTGATTTAACAAAAATTTAACGCGAATTTTAACAAAATATTAACGCTTACA
ATTTAGGTGGCACTTTTCGGGGAAATGTGCGCGGAACCCCTATTTGTTTATTTTTCTAAATACATTCAAATATG
TATCCGCTCATGCCAGGTCTTGGACTGGTGAGAACGGCTTGCTCGGCAGCTTCGATGTGTGCTGGAGGGA
GAATAAAGGTCTAAGATGTGCGATAGAGGGAAGTCGCATTGAATTATGTGCTGTGTAGGGATCGCTGGTATC
AAATATGTGTGCCCACCCCTGGCATGAGACAATAACCCTGATAAATGCTTCAATAATATTGAAAAAGGAAGAG
TATGAGTATTCAACATTTCCGTGTCGCCCTTATTCCCTTTTTTGCGGCATTTTGCCTTCCTGTTTTTGCTCAC
CCAGAAACGCTGGTGAAAGTAAAAGATGCTGAAGATCAGTTGGGTGCACGAGTGGGTTACATCGAACTGG
ATCTCAACAGCGGTAAGATCCTTGAGAGTTTTCGCCCCGAAGAACGTTTTCCAATGATGAGCACTTTTAAAG

TTCTGCTATGTGGCGCGGTATTATCCCGTATTGACGCCGGGCAAGAGCAACTCGGTCGCCGCATACACTAT
TCTCAGAATGACTTGGTTGAGTACTCACCAGTCACAGAAAAGCATCTTACGGATGGCATGACAGTAAGAGAA
TTATGCAGTGCTGCCATAACCATGAGTGATAACACTGCGGCCAACTTACTTCTGACAACGATCGGAGGACC
GAAGGAGCTAACCGCTTTTTTGCACAACATGGGGGATCATGTAACTCGCCTTGATCGTTGGGAACCGGAGC
TGAATGAAGCCATACCAAACGACGAGCGTGACACCACGATGCCTGTAGCAATGGCAACAACGTTGCGCAA
ACTATTAACTGGCGAACTACTTACTCTAGCTTCCCGGCAACAATTAATAGACTGGATGGAGGCGGATAAAGTT
GCAGGACCACTTCTGCGCTCGGCCCTTCCGGCTG

Table S5: NHEs, GGA3 plasmids sequence

NHEs, GGA3 (cyan), HA, GFP, Flag tag (green)

**Seq_hNHE1-HA**
ATTTATGCAGAGGCCGAGGCCGCCTCTGCCTCTGAGCTATTCCAGAAGTAGTGAGGAGGCTTTTTTGGAGG<br>CCTAGGCTTTTGCAAAAAGCTCCCGGGAGCTTGTATATCCATTTTCGGATCTGATCAAGAGACAGGATGAGG<br>ATCGTTTCGCATGATTGAACAAGATGGATTGCACGCAGGTTCTCCGGCCGCTTGGGTGGAGAGGCTATTCG<br>GCTATGACTGGGCACAACAGACAATCGGCTGCTCTGATGCCGCCGTGTTCCGGCTGTCAGCGCAGGGGC<br>GCCCGGTTCTTTTTGTCAAGACCGACCTGTCCGGTGCCCTGAATGAACTGCAGGACGAGGCAGCGCGGC<br>TATCGTGGCTGGCCACGACGGGCGTTCCTTGCGCAGCTGTGCTCGACGTTGTCACTGAAGCGGGAAGGG<br>ACTGGCTGCTATTGGGCGAAGTGCCGGGGCAGGATCTCCTGTCATCTCACCTTGCTCCTGCCGAGAAAGT<br>ATCCATCATGGCTGATGCAATGCGGCGGCTGCATACGCTTGATCCGGCTACCTGCCCATTCGACCACCAAG<br>CGAAACATCGCATCGAGCGAGCACGTACTCGGATGGAAGCCGGTCTTGTCGATCAGGATGATCTGGACGA<br>AGAGCATCAGGGGCTCGCGCCAGCCGAACTGTTCGCCAGGCTCAAGGCGCGCATGCCCGACGGCGAGG<br>ATCTCGTCGTGACCCATGGCGATGCCTGCTTGCCGAATATCATGGTGGAAAATGGCCGCTTTTCTGGATTC<br>ATCGACTGTGGCCGGCTGGGTGTGGCGGACCGCTATCAGGACATAGCGTTGGCTACCCGTGATATTGCTG<br>AAGAGCTTGGCGGCGAATGGGCTGACCGCTTCCTCGTGCTTTACGGTATCGCCGCTCCCGATTCGCAGCG<br>CATCGCCTTCTATCGCCTTCTTGACGAGTTCTTCTGAGCGGGACTCTGGGGTTCGCGAAATGACCGACCAA<br>GCGACGCCCAACCTGCCATCACGAGATTTCGATTCCACCGCCGCCTTCTATGAAAGGTTGGGCTTCGGAAT<br>CGTTTTCCGGGACGCCGGCTGGATGATCCTCCAGCGCGGGGATCTCATGCTGGAGTTCTTCGCCCACCCC<br>AACTTGTTTATTGCAGCTTATAATGGTTACAAATAAAGCAATAGCATCACAAATTTCACAAATAAAGCATTTTTT<br>TCACTGCATTCTAGTTGTGGTTTGTCCAAACTCATCAATGTATCTTATCATGTCTGTATACCGTCGACCTCTAG<br>CTAGAGCTTGGCGTAATCATGGTCATAGCTGTTTCCTGTGTGAAATTGTTATCCGCTCACAATTCCACACAAC<br>ATACGAGCCGGAAGCATAAAGTGTAAAGCCTGGGGTGCCTAATGAGTGAGCTAACTCACATTAATTGCGTTG<br>CGCTCACTGCCCGCTTTCCAGTCGGGAAACCTGTCGTGCCAGCTGCATTAATGAATCGGCCAACGCGCGG<br>GGAGAGGCGGTTTGCGTATTGGGCGCTCTTCCGCTTCCTCGCTCACTGACTCGCTGCGCTCGGTCGTTCG<br>GCTGCGGCGAGCGGTATCAGCTCACTCAAAGGCGGTAATACGGTTATCCACAGAATCAGGGGATAACGCA<br>GGAAAGAACATGTGAGCAAAAGGCCAGCAAAAGGCCAGGAACCGTAAAAAGGCCGCGTTGCTGGCGTTTT<br>TCCATAGGCTCCGCCCCCCTGACGAGCATCACAAAAATCGACGCTCAAGTCAGAGGTGGCGAAACCCGAC<br>AGGACTATAAAGATACCAGGCGTTTCCCCCTGGAAGCTCCCTCGTGCGCTCTCCTGTTCCGACCCTGCCG<br>CTTACCGGATACCTGTCCGCCTTTCTCCCTTCGGGAAGCGTGGCGCTTTCTCATAGCTCACGCTGTAGGTA<br>TCTCAGTTCGGTGTAGGTCGTTCGCTCCAAGCTGGGCTGTGTGCACGAACCCCCCGTTCAGCCCGACCG<br>CTGCGCCTTATCCGGTAACTATCGTCTTGAGTCCAACCCGGTAAGACACGACTTATCGCCACTGGCAGCAG<br>CCACTGGTAACAGGATTAGCAGAGCGAGGTATGTAGGCGGTGCTACAGAGTTCTTGAAGTGGTGGCCTAAC<br>TACGGCTACACTAGAAGAACAGTATTTGGTATCTGCGCTCTGCTGAAGCCAGTTACCTTCGGAAAAAGAGTT<br>GGTAGCTCTTGATCCGGCAAACAAACCACCGCTGGTAGCGGTGGTTTTTTTGTTTGCAAGCAGCAGATTAC<br>GCGCAGAAAAAAAGGATCTCAAGAAGATCCTTTGATCTTTTCTACGGGGTCTGACGCTCAGTGGAACGAAA<br>ACTCACGTTAAGGGATTTTGGTCATGAGATTATCAAAAAGGATCTTCACCTAGATCCTTTTAAATTAAAAATGA<br>AGTTTTAAATCAATCTAAAGTATATATGAGTAAACTTGGTCTGACAGTTACCAATGCTTAATCAGTGAGGCACC<br>TATCTCAGCGATCTGTCTATTTCGTTCATCCATAGTTGCCTGACTCCCCGTCGTGTAGATAACTACGATACGG<br>GAGGGCTTACCATCTGGCCCCAGTGCTGCAATGATACCGCGAGACCCACGCTCACCGGCTCCAGATTTATC<br>AGCAATAAACCAGCCAGCCGGAAGGGCCGAGCGCAGAAGTGGTCCTGCAACTTTATCCGCCTCCATCCAG<br>TCTATTAATTGTTGCCGGGAAGCTAGAGTAAGTAGTTCGCCAGTTAATAGTTTGCGCAACGTTGTTGCCATTG<br>CTACAGGCATCGTGGTGTCACGCTCGTCGTTTGGTATGGCTTCATTCAGCTCCGGTTCCCAACGATCAAGG<br>CGAGTTACATGATCCCCCATGTTGTGCAAAAAAGCGGTTAGCTCCTTCGGTCCTCCGATCGTTGTCAGAAG<br>TAAGTTGGCCGCAGTGTTATCACTCATGGTTATGGCAGCACTGCATAATTCTCTTACTGTCATGCCATCCGTA<br>AGATGCTTTTCTGTGACTGGTGAGTACTCAACCAAGTCATTCTGAGAATAGTGTATGCGGCGACCGAGTTG

CTCTTGCCCGGCGTCAATACGGGATAATACCGCGCCACATAGCAGAACTTTAAAAGTGCTCATCATTGGAAA
ACGTTCTTCGGGGCGAAAACTCTCAAGGATCTTACCGCTGTTGAGATCCAGTTCGATGTAACCCACTCGTG
CACCCAACTGATCTTCAGCATCTTTTACTTTCACCAGCGTTTCTGGGTGAGCAAAAACAGGAAGGCAAAATG
CCGCAAAAAAGGGAATAAGGGCGACACGGAAATGTTGAATACTCATACTCTTCCTTTTTCAATATTATTGAAG
CATTTATCAGGGTTATTGTCTCATGAGCGGATACATATTTGAATGTATTTAGAAAAATAAACAAATAGGGGTTC
CGCGCACATTTCCCCGAAAAGTGCCACCTGACGTCGACGGATCGGGAGATCTCCCGATCCCCTATGGTGC
ACTCTCAGTACAATCTGCTCTGATGCCGCATAGTTAAGCCAGTATCTGCTCCCTGCTTGTGTGTTGGAGGTC
GCTGAGTAGTGCGCGAGCAAAATTTAAGCTACAACAAGGCAAGGCTTGACCGACAATTGCATGAAGAATCT
GCTTAGGGTTAGGCGTTTTGCGCTGCTTCGCGATGTACGGGCCAGATATACGCGTTGACATTGATTATTGAC
TAGTTATTAATAGTAATCAATTACGGGGTCATTAGTTCATAGCCCATATATGGAGTTCCGCGTTACATAACTTAC
GGTAAATGGCCCGCCTGGCTGACCGCCCAACGACCCCCGCCCATTGACGTCAATAATGACGTATGTTCCCA
TAGTAACGCCAATAGGGACTTTCCATTGACGTCAATGGGTGGAGTATTTACGGTAAACTGCCCACTTGGCAG
TACATCAAGTGTATCATATGCCAAGTACGCCCCCTATTGACGTCAATGACGGTAAATGGCCCGCCTGGCATT
ATGCCCAGTACATGACCTTATGGGACTTTCCTACTTGGCAGTACATCTACGTATTAGTCATCGCTATTACCATG
GTGATGCGGTTTTGGCAGTACATCAATGGGCGTGGATAGCGGTTTGACTCACGGGGATTTCCAAGTCTCCA
CCCCATTGACGTCAATGGGAGTTTGTTTTGGCACCAAAATCAACGGGACTTTCCAAAATGTCGTAACAACTC
CGCCCCATTGACGCAAATGGGCGGTAGGCGTGTACGGTGGGAGGTCTATATAAGCAGAGCTCTCTGGCTA
ACTAGAGAACCCACTGCTTACTGGCTTATCGAAATTAATACGACTCACTATAGGGAGACCCAAGCTGGCTAG
TTAAGCTTGATCAAACAAGTTTGTACAAAAAAGCAGGCTTGAAGGAATTCGGTACCATGGTTCTGCGGTCTG
GCATCTGTGGCCTCTCTCCACATCGGATCTTCCCTTCCTTACTCGTGGTGGTTGCTTTGGTGGGGCTGCTG
CCTGTTCTCAGGAGCCATGGCCTCCAGCTCAGCCCAACTGCCAGCACCATTCGAAGCTCAGAGCCACCAC
GAGAACGCTCGATTGGGGATGTCACCACCGCTCCACCGGAGGTCACCCCAGAGAGCCGCCCTGTTAATCA
TTCCGTCACTGATCATGGCATGAAGCCGCGCAAGGCCTTTCCAGTCCTGGGCATCGACTACACACACGTG
CGCACCCCCTTCGAGATCTCCCTCTGGATCCTTCTGGCCTGCCTCATGAAGATAGGTTTCCATGTGATCCC
CACTATCTCAAGCATCGTCCCGGAGAGCTGCCTGCTGATCGTGGTGGGGCTGCTGGTGGGGGGCCTGAT
CAAGGGTGTAGGCGAGACACCCCCCTTCCTGCAGTCCGACGTCTTCTTCCTCTTCCTGCTGCCGCCCATC
ATCCTGGATGCGGGCTACTTCCTGCCACTGCGGCAGTTCACAGAAAACCTGGGCACCATCCTGATCTTTGC
CGTGGTGGGCACGCTGTGGAACGCCTTCTTCCTGGGCGGCCTCATGTACGCCGTGTGCCTGGTGGGCGG
TGAGCAGATCAACAACATCGGCCTCCTGGACAACCTGCTCTTCGGCAGCATCATCTCGGCCGTGGACCCC
GTGGCGGTTCTGGCTGTCTTTGAGGAAATTCACATCAATGAGCTGCTGCACATCCTTGTTTTTGGGGAGTC
CTTGCTCAATGACGCCGTCACTGTGGTCCTGTATCACCTCTTTGAGGAGTTTGCCAACTACGAACACGTGG
GCATCGTGGACATCTTCCTCGGCTTCCTGAGCTTCTTCGTGGTGGCCCTGGGCGGGGTGCTTGTGGGCG
TGGTCTACGGGGTCATCGCAGCCTTCACCTCCCGATTTACCTCCCACATCCGGGTCATCGAGCCGCTCTTC
GTCTTCCTCTACAGCTACATGGCCTACTTGTCAGCCGAGCTCTTCCACCTGTCAGGCATCATGGCGCTCATA
GCCTCAGGAGTGGTGATGCGCCCCTATGTGGAGGCCAACATCTCCCACAAGTCCCACACCACCATCAAATA
CTTCCTGAAGATGTGGAGCAGCGTCAGCGAGACCCTCATCTTCATCTTCCTCGGCGTCTCCACGGTGGCC
GGCTCCCACCACTGGAACTGGACCTTCGTCATCAGCACCCTGCTCTTCTGCCTCATCGCCCGCGTGCTGG
GGGTGCTGGGCCTGACCTGGTTCATCAACAAGTTCCGTATCGTGAAGCTGACCCCCAAGGACCAGTTCAT
CATCGCCTATGGGGGCCTGCGAGGGGCCATCGCCTTCTCTCTGGGCTACCTCCTGGACAAGAAGCACTTC
CCCATGTGTGACCTGTTCCTCACTGCCATCATCACTGTCATCTTCTTCACCGTCTTTGTGCAGGGCATGACC
ATTCGGCCCCTGGTAGACCTGTTGGCTGTGAAGAAAAAGCAAGAGACGAAGCGCTCCATCAACGAAGAGA
TCCACACACAGTTCCTGGACCACCTTCTGACAGGCATCGAAGACATCTGTGGCCACTACGGTCACCACCA
CTGGAAGGACAAGCTCAACCGGTTTAATAAGAAATATGTGAAGAAGTGTCTGATAGCTGGCGAGCGCTCCA
AGGAGCCCCAGCTCATTGCCTTCTACCACAAGATGGAGATGAAGCAGGCCATCGAGCTGGTGGAGAGCGG
GGGCATGGGCAAGATCCCCTCTGCCGTCTCCACCGTCTCCATGCAGAACATCCACCCCAAGTCCCTGCCT
TCCGAGCGCATCCTGCCAGCACTGTCCAAGGACAAGGAGGAGGAGATCCGCAAAATCCTGAGGAACAACT
TGCAGAAGACCAGGCAGCGGCTGCGGTCCTACAACAGACACACGCTGGTGGCAGACCCCTACGAGGAAG
CCTGGAACCAGATGCTGCTCCGGAGGCAGAAGGCCCGGCAGCTGGAGCAGAAGATCAACAACTACCTGA

CGGTGCCAGCCCACAAGCTGGACTCACCCACCATGTCTCGGGCCCGCATCGGCTCAGACCCACTGGCCT
ATGAGCCGAAGGAGGACCTGCCTGTCATCACCATCGACCCGGCTTCCCCGCAGTCACCCGAGTCTGTGG
ACCTGGTGAATGAGGAGCTGAAGGGCAAAGTCTTAGGGTTGAGCCGGGATCCTGCAAAGGTGGCTGAGG
AGGACGAGGACGACGATGGGGGCATCATGATGCGGAGCAAGGAGACTTCGTCCCCAGGAACCGACGATG
TCTTCACCCCCGCGCCCAGTGACAGCCCCAGCTCCCAGAGGATACAGCGCTGCCTCAGTGACCCAGGCC
CACACCCTGAGCCTGGGGAGGGAGAACCGTTCTTCCCCAAGGGGCAGTACCTCGAGTGCGGCCGCATGG
CTAGCTACCCTTACGACGTCCCAGACTACGCTGGATCCTACCCTTACGACGTCCCAGACTACGCTTACCCTT
ACGACGTCCCAGACTACGCTTGATCAGCCTCGACTGTGCCTTCTAGTTGCCAGCCATCTGTTGTTTGCCCC
TCCCCCGTGCCTTCCTTGACCCTGGAAGGTGCCACTCCCACTGTCCTTTCCTAATAAAATGAGGAAATTGC
ATCGCATTGTCTGAGTAGGTGTCATTCTATTCTGGGGGGTGGGGTGGGGCAGGACAGCAAGGGGGAGGAT
TGGGAAGACAATAGCAGGCATGCTGGGGATGCGGTGGGCTCTATGGCTTCTGAGGCGGAAAGAACCAGCT
GGGGCTCTAGGGGGTATCCCCACGCGCCCTGTAGCGGCGCATTAAGCGCGGCGGGTGTGGTGGTTACGC
GCAGCGTGACCGCTACACTTGCCAGCGCCCTAGCGCCCGCTCCTTTCGCTTTCTTCCCTTCCTTTCTCGC
CACGTTCGCCGGCTTTCCCCGTCAAGCTCTAAATCGGGGGCTCCCTTTAGGGTTCCGATTTAGTGCTTTAC
GGCACCTCGACCCCAAAAAACTTGATTAGGGTGATGGTTCACGTAGTGGGCCATCGCCCTGATAGACGGTT
TTTCGCCCTTTGACGTTGGAGTCCACGTTCTTTAATAGTGGACTCTTGTTCCAAACTGGAACAACACTCAAC
CCTATCTCGGTCTATTCTTTTGATTTATAAGGGATTTTGCCGATTTCGGCCTATTGGTTAAAAAATGAGCTGAT
TTAACAAAAATTTAACGCGAATTAATTCTGTGGAATGTGTGTCAGTTAGGGTGTGGAAAGTCCCCAGGCTCC
CCAGCAGGCAGAAGTATGCAAAGCATGCATCTCAATTAGTCAGCAACCAGGTGTGGAAAGTCCCCAGGCT
CCCCAGCAGGCAGAAGTATGCAAAGCATGCATCTCAATTAGTCAGCAACCATAGTCCCGCCCCTAACTCCG
CCCATCCCGCCCCTAACTCCGCCCAGTTCCGCCCATTCTCCGCCCCATGGCTGACTAATTTTTTTT

Seq_hNHE5-HA

ACTGATCTTCAGCATCTTTTACTTTCACCAGCGTTTCTGGGTGAGCAAAAACAGGAAGGCAAAATGCCGCA
AAAAAGGGAATAAGGGCGACACGGAAATGTTGAATACTCATACTCTTCCTTTTTCAATATTATTGAAGCATTTA
TCAGGGTTATTGTCTCATGAGCGGATACATATTTGAATGTATTTAGAAAAATAAACAAATAGGGGTTCCGCGC
ACATTTCCCCGAAAAGTGCCACCTGACGTCGACGGATCGGGAGATCTCCCGATCCCCTATGGTGCACTCTC
AGTACAATCTGCTCTGATGCCGCATAGTTAAGCCAGTATCTGCTCCCTGCTTGTGTGTTGGAGGTCGCTGA
GTAGTGCGCGAGCAAAATTTAAGCTACAACAAGGCAAGGCTTGACCGACAATTGCATGAAGAATCTGCTTA
GGGTTAGGCGTTTTGCGCTGCTTCGCGATGTACGGGCCAGATATACGCGTTGACATTGATTATTGACTAGTT
ATTAATAGTAATCAATTACGGGGTCATTAGTTCATAGCCCATATATGGAGTTCCGCGTTACATAACTTACGGTA
AATGGCCCGCCTGGCTGACCGCCCAACGACCCCCGCCCATTGACGTCAATAATGACGTATGTTCCCATAGT
AACGCCAATAGGGACTTTCCATTGACGTCAATGGGTGGAGTATTTACGGTAAACTGCCCACTTGGCAGTACA
TCAAGTGTATCATATGCCAAGTACGCCCCCTATTGACGTCAATGACGGTAAATGGCCCGCCTGGCATTATGC
CCAGTACATGACCTTATGGGACTTTCCTACTTGGCAGTACATCTACGTATTAGTCATCGCTATTACCATGGTG
ATGCGGTTTTGGCAGTACATCAATGGGCGTGGATAGCGGTTTGACTCACGGGGATTTCCAAGTCTCCACCC
CATTGACGTCAATGGGAGTTTGTTTTGGCACCAAAATCAACGGGACTTTCCAAAATGTCGTAACAACTCCGC
CCCATTGACGCAAATGGGCGGTAGGCGTGTACGGTGGGAGGTCTATATAAGCAGAGCTCTCTGGCTAACTA
GAGAACCCACTGCTTACTGGCTTATCGAAATTAATACGACTCACTATAGGGAGACCCAAGCTGGCTAGTTAA
GCTTGATCAAACAAGTTTGTACAAAAAAGCAGGCTTGAAGGAATTCGGTACCATGCTGAGAGCCGCCCTGT
CTCTGCTGGCCCTGCCACTGGCAGGAGCAGCAGAGGAGCCTACCCAGAAGCCAGAGAGCCCAGGAGAG
CCACCTCCAGGACTGGAGCTGTTCAGATGGCAGTGGCACGAGGTGGAGGCACCATACCTGGTGGCCCTG
TGGATCCTGGTGGCAAGCCTGGCCAAGATCGTGTTTCACCTGTCTAGGAAGGTGACCAGCCTGGTGCCTG
AGTCCTGCCTGCTGATCCTGCTGGGACTGGTGCTGGGAGGAATCGTGCTGGCCGTGGCCAAGAAGGCCG
AGTATCAGCTGGAGCCAGGCACATTCTTTCTGTTCCTGCTGCCCCCTATCGTGCTGGACTCCGGCTACTTT
ATGCCCTCTCGCCTGTTCTTTGATAACCTGGGCGCCATCCTGACCTACGCAGTGGTTGGGACTTTGTGGAA
TGCCTTCACCACAGGAGCCGCCCTGTGGGGACTGCAGCAGGCAGGCCTGGTGGCCCCTAGGGTGCAGG
CAGGACTGCTGGACTTCCTGCTGTTTGGCAGCCTGATCTCCGCCGTGGATCCAGTGGCCGTGCTGGCCG
TGTTTGAGGAGGTGCACGTGAACGAGACCCTGTTCATCATCGTGTTTGGCGAGAGCCTGCTGAATGACGC
CGTGACAGTGGTGCTGTACAAGGTGTGCAACAGCTTCGTGGAGATGGGCTCCGCCAATGTGCAGGCCAC

CGATTATCTGAAGGGCGTGGCCTCTCTGTTTGTGGTGAGCCTGGGAGGAGCAGCAGTGGGACTGGTGTTC
GCCTTTCTGCTGGCCCTGACCACAAGATTCACAAAGAGAGTGAGGATCATCGAGCCTCTGCTGGTGTTTCT
GCTGGCCTACGCCGCCTATCTGACCGCCGAGATGGCCTCTCTGAGCGCCATCCTGGCCGTGACAATGTGC
GGCCTGGGCTGTAAGAAGTACGTGGAGGCCAACATCTCTCACAAGAGCAGGACCACAGTGAAGTATACCA
TGAAGACACTGGCCTCCTGTGCCGAGACCGTGATCTTCATGCTGCTGGGCATCTCCGCCGTGGACAGCTC
CAAGTGGGCATGGGATTCTGGCCTGGTGCTGGGCACCCTGATCTTCATCCTGTTCTTTCGCGCCCTGGGA
GTGGTGCTGCAGACATGGGTGCTGAATCAGTTTCGGCTGGTGCCACTGGACAAGATCGATCAGGTGGTCA
TGAGCTACGGAGGACTGAGGGGAGCAGTGGCCTTCGCCCTGGTCATCCTGCTGGACCGGACCAAGGTGC
CCGCCAAGGATTATTTTGTGGCCACCACAATCGTGGTGGTGTTCTTTACCGTGATCGTGCAGGGCCTGACA
ATCAAGCCCCTGGTGAAGTGGCTGAAGGTGAAGCGGTCCGAGCACCACAAGCCTACCCTGAACCAGGAG
CTGCACGAGCACACATTCGACCACATCCTGGCAGCAGTGGAGGATGTGGTGGGACACCACGGCTACCACT
ATTGGCGCGACCGGTGGGAGCAGTTTGATAAGAAGTACCTGTCCCAGCTGCTGATGCGGAGATCTGCCTAT
AGAATCAGGGACCAGATCTGGGACGTGTACTATCGCCTGAATATCCGGGACGCCATCAGCTTCGTGGATCA
GGGAGGACACGTGCTGTCTAGCACCGGACTGACACTGCCTTCCATGCCATCTAGAAACAGCGTGGCCGAG
ACCTCCGTGACAAATCTGCTGAGGGAGTCCGGATCTGGAGCATGCCTGGACCTGCAGGTCATCGATACCG
TGCGCTCTGGAAGGGACAGGGAGGATGCCGTGATGCACCACCTGCTGTGCGGAGGACTGTACAAGCCAA
GGCGCCGGTATAAGGCCAGCTGTTCCAGACACTTCATCTCTGAGGACGCCCAGGAGAGGCAGGATAAGGA
GGTGTTTCAGCAGAACATGAAGAGAAGGCTGGAGTCCTTCAAGTCTACCAAGCACAATATCTGTTTTACAAA
GTCCAAGCCTAGACCAAGGAAGACCGGCCGCCGGAAGAAGGACGGAGTGGCAAACGCAGAGGCAACAA
ATGGCAAGCACAGGGGACTGGGATTCCAGGACACCGCAGCCGTGATCCTGACAGTGGAGAGCGAGGAGG
AGGAGGAGGAGTCCGATTCCTCTGAGACCGAGAAGGAGGACGATGAGGGCATCATCTTTGTGGCAAGGG
CCACAAGCGAGGTGCTGCAGGAGGGCAAGGTGTCTGGAAGCCTGGAGGTGTGCCCATCTCCTAGGATCA
TCCCACCCAGCCCAACCTGTGCCGAGAAGGAGCTGCCATGGAAGAGCGGACAGGGCGACCTGGCCGTG
TACGTGAGCTCCGAGACCACAAAGATCGTGCCTGTGGATATGCAGACAGGCTGGAACCAGTCCATCTCTAG
CCTGGAGAGCCTGGCCTCCCCTCCATGCAATCAGGCCCCTATCCTGACCTGTCTGCCCCCTCACCCAAGA
GGCACAGAGGAGCCCCAGGTGCCTCTGCACCTGCCAAGCGACCCCAGGTCCTCTTTCGCCTTTCCACCAT
CCCTGGCAAAGGCAGGCCGCTCCCGGTCTGAGAGCTCCGCCGACCTGCCTCAGCAGCAGGAGCTGCAG
CCACTGATGGGCCACAAGGATCACACCCACCTGTCTCCAGGCACCGCCACAAGCCACTGGTGTATCCAGT
TCAACAGAGGCTCCAGGCTGTACCTCGAGTGCGGCCGCATGGCTAGCTACCCTTACGACGTCCCAGACTA
CGCTGGATCCTACCCTTACGACGTCCCAGACTACGCTTACCCTTACGACGTCCCAGACTACGCTTGATCAG
CCTCGACTGTGCCTTCTAGTTGCCAGCCATCTGTTGTTTGCCCCTCCCCCGTGCCTTCCTTGACCCTGGAA
GGTGCCACTCCCACTGTCCTTTCCTAATAAAATGAGGAAATTGCATCGCATTGTCTGAGTAGGTGTCATTCTA
TTCTGGGGGGTGGGGTGGGGCAGGACAGCAAGGGGGAGGATTGGGAAGACAATAGCAGGCATGCTGGG
GATGCGGTGGGCTCTATGGCTTCTGAGGCGGAAAGAACCAGCTGGGGCTCTAGGGGGTATCCCCACGCG
CCCTGTAGCGGCGCATTAAGCGCGGCGGGTGTGGTGGTTACGCGCAGCGTGACCGCTACACTTGCCAGC
GCCCTAGCGCCCGCTCCTTTCGCTTTCTTCCCTTCCTTTCTCGCCACGTTCGCCGGCTTTCCCCGTCAAGC
TCTAAATCGGGGGCTCCCTTTAGGGTTCCGATTTAGTGCTTTACGGCACCTCGACCCCAAAAAACTTGATTA
GGGTGATGGTTCACGTAGTGGGCCATCGCCCTGATAGACGGTTTTTCGCCCTTTGACGTTGGAGTCCACG
TTCTTTAATAGTGGACTCTTGTTCCAAACTGGAACAACACTCAACCCTATCTCGGTCTATTCTTTTGATTTATA
AGGGATTTTGCCGATTTCGGCCTATTGGTTAAAAAATGAGCTGATTTAACAAAAATTTAACGCGAATTAATTCT
GTGGAATGTGTGTCAGTTAGGGTGTGGAAAGTCCCCAGGCTCCCCAGCAGGCAGAAGTATGCAAAGCATG
CATCTCAATTAGTCAGCAACCAGGTGTGGAAAGTCCCCAGGCTCCCCAGCAGGCAGAAGTATGCAAAGCAT
GCATCTCAATTAGTCAGCAACCATAGTCCCGCCCCTAACTCCGCCCATCCCGCCCCTAACTCCGCCCAGTT
CCGCCCATTCTCCGCCCCATGGCTGACTAATTTTTTTTATTTATGCAGAGGCCGAGGCCGCCTCTGCCTCTG
AGCTATTCCAGAAGTAGTGAGGAGGCTTTTTTGGAGGCCTAGGCTTTTGCAAAAAGCTCCCGGGAGCTTGT
ATATCCATTTTCGGATCTGATCAAGAGACAGGATGAGGATCGTTTCGCATGATTGAACAAGATGGATTGCAC
GCAGGTTCTCCGGCCGCTTGGGTGGAGAGGCTATTCGGCTATGACTGGGCACAACAGACAATCGGCTGCT
CTGATGCCGCCGTGTTCCGGCTGTCAGCGCAGGGGCGCCCGGTTCTTTTTGTCAAGACCGACCTGTCCG
GTGCCCTGAATGAACTGCAGGACGAGGCAGCGCGGCTATCGTGGCTGGCCACGACGGGCGTTCCTTGCG
CAGCTGTGCTCGACGTTGTCACTGAAGCGGGAAGGGACTGGCTGCTATTGGGCGAAGTGCCGGGGCAGG
ATCTCCTGTCATCTCACCTTGCTCCTGCCGAGAAAGTATCCATCATGGCTGATGCAATGCGGCGGCTGCATA
CGCTTGATCCGGCTACCTGCCCATTCGACCACCAAGCGAAACATCGCATCGAGCGAGCACGTACTCGGAT
GGAAGCCGGTCTTGTCGATCAGGATGATCTGGACGAAGAGCATCAGGGGCTCGCGCCAGCCGAACTGTT
CGCCAGGCTCAAGGCGCGCATGCCCGACGGCGAGGATCTCGTCGTGACCCATGGCGATGCCTGCTTGCC
GAATATCATGGTGGAAAATGGCCGCTTTTCTGGATTCATCGACTGTGGCCGGCTGGGTGTGGCGGACCGC
TATCAGGACATAGCGTTGGCTACCCGTGATATTGCTGAAGAGCTTGGCGGCGAATGGGCTGACCGCTTCCT

CGTGCTTTACGGTATCGCCGCTCCCGATTCGCAGCGCATCGCCTTCTATCGCCTTCTTGACGAGTTCTTCT
GAGCGGGACTCTGGGGTTCGCGAAATGACCGACCAAGCGACGCCCAACCTGCCATCACGAGATTTCGATT
CCACCGCCGCCTTCTATGAAAGGTTGGGCTTCGGAATCGTTTTCCGGGACGCCGGCTGGATGATCCTCCA
GCGCGGGGATCTCATGCTGGAGTTCTTCGCCCACCCCAACTTGTTTATTGCAGCTTATAATGGTTACAAATA
AAGCAATAGCATCACAAATTTCACAAATAAAGCATTTTTTTCACTGCATTCTAGTTGTGGTTTGTCCAAACTCA
TCAATGTATCTTATCATGTCTGTATACCGTCGACCTCTAGCTAGAGCTTGGCGTAATCATGGTCATAGCTGTTT
CCTGTGTGAAATTGTTATCCGCTCACAATTCCACACAACATACGAGCCGGAAGCATAAAGTGTAAAGCCTGG
GGTGCCTAATGAGTGAGCTAACTCACATTAATTGCGTTGCGCTCACTGCCCGCTTTCCAGTCGGGAAACCT
GTCGTGCCAGCTGCATTAATGAATCGGCCAACGCGCGGGGAGAGGCGGTTTGCGTATTGGGCGCTCTTCC
GCTTCCTCGCTCACTGACTCGCTGCGCTCGGTCGTTCGGCTGCGGCGAGCGGTATCAGCTCACTCAAAG
GCGGTAATACGGTTATCCACAGAATCAGGGGATAACGCAGGAAAGAACATGTGAGCAAAAGGCCAGCAAAA
GGCCAGGAACCGTAAAAAGGCCGCGTTGCTGGCGTTTTTCCATAGGCTCCGCCCCCCTGACGAGCATCAC
AAAAATCGACGCTCAAGTCAGAGGTGGCGAAACCCGACAGGACTATAAAGATACCAGGCGTTTCCCCCTG
GAAGCTCCCTCGTGCGCTCTCCTGTTCCGACCCTGCCGCTTACCGGATACCTGTCCGCCTTTCTCCCTTC
GGGAAGCGTGGCGCTTTCTCATAGCTCACGCTGTAGGTATCTCAGTTCGGTGTAGGTCGTTCGCTCCAAGC
TGGGCTGTGTGCACGAACCCCCCGTTCAGCCCGACCGCTGCGCCTTATCCGGTAACTATCGTCTTGAGTC
CAACCCGGTAAGACACGACTTATCGCCACTGGCAGCAGCCACTGGTAACAGGATTAGCAGAGCGAGGTAT
GTAGGCGGTGCTACAGAGTTCTTGAAGTGGTGGCCTAACTACGGCTACACTAGAAGAACAGTATTTGGTATC
TGCGCTCTGCTGAAGCCAGTTACCTTCGGAAAAAGAGTTGGTAGCTCTTGATCCGGCAAACAAACCACCG
CTGGTAGCGGTGGTTTTTTTGTTTGCAAGCAGCAGATTACGCGCAGAAAAAAAGGATCTCAAGAAGATCCT
TTGATCTTTTCTACGGGGTCTGACGCTCAGTGGAACGAAAACTCACGTTAAGGGATTTTGGTCATGAGATTA
TCAAAAAGGATCTTCACCTAGATCCTTTTAAATTAAAAATGAAGTTTTAAATCAATCTAAAGTATATATGAGTAA
ACTTGGTCTGACAGTTACCAATGCTTAATCAGTGAGGCACCTATCTCAGCGATCTGTCTATTTCGTTCATCCA
TAGTTGCCTGACTCCCCGTCGTGTAGATAACTACGATACGGGAGGGCTTACCATCTGGCCCCAGTGCTGCA
ATGATACCGCGAGACCCACGCTCACCGGCTCCAGATTTATCAGCAATAAACCAGCCAGCCGGAAGGGCCG
AGCGCAGAAGTGGTCCTGCAACTTTATCCGCCTCCATCCAGTCTATTAATTGTTGCCGGGAAGCTAGAGTAA
GTAGTTCGCCAGTTAATAGTTTGCGCAACGTTGTTGCCATTGCTACAGGCATCGTGGTGTCACGCTCGTCG
TTTGGTATGGCTTCATTCAGCTCCGGTTCCCAACGATCAAGGCGAGTTACATGATCCCCCATGTTGTGCAAA
AAAGCGGTTAGCTCCTTCGGTCCTCCGATCGTTGTCAGAAGTAAGTTGGCCGCAGTGTTATCACTCATGGT
TATGGCAGCACTGCATAATTCTCTTACTGTCATGCCATCCGTAAGATGCTTTTCTGTGACTGGTGAGTACTCA
ACCAAGTCATTCTGAGAATAGTGTATGCGGCGACCGAGTTGCTCTTGCCCGGCGTCAATACGGGATAATAC
CGCGCCACATAGCAGAACTTTAAAAGTGCTCATCATTGGAAAACGTTCTTCGGGGCGAAAACTCTCAAGGA
TCTTACCGCTGTTGAGATCCAGTTCGATGTAACCCACTCGTGCACCCA

Seq_hNHE6-HA

TCAATACGGGATAATACCGCGCCACATAGCAGAACTTTAAAAGTGCTCATCATTGGAAAACGTTCTTCGGGG
CGAAAACTCTCAAGGATCTTACCGCTGTTGAGATCCAGTTCGATGTAACCCACTCGTGCACCCAACTGATCT
TCAGCATCTTTTACTTTCACCAGCGTTTCTGGGTGAGCAAAAACAGGAAGGCAAAATGCCGCAAAAAAGGG
AATAAGGGCGACACGGAAATGTTGAATACTCATACTCTTCCTTTTTCAATATTATTGAAGCATTTATCAGGGTT
ATTGTCTCATGAGCGGATACATATTTGAATGTATTTAGAAAAATAAACAAATAGGGGTTCCGCGCACATTTCCC
CGAAAAGTGCCACCTGACGTCGACGGATCGGGAGATCTCCCGATCCCCTATGGTGCACTCTCAGTACAATC
TGCTCTGATGCCGCATAGTTAAGCCAGTATCTGCTCCCTGCTTGTGTGTTGGAGGTCGCTGAGTAGTGCGC
GAGCAAAATTTAAGCTACAACAAGGCAAGGCTTGACCGACAATTGCATGAAGAATCTGCTTAGGGTTAGGC
GTTTTGCGCTGCTTCGCGATGTACGGGCCAGATATACGCGTTGACATTGATTATTGACTAGTTATTAATAGTA
ATCAATTACGGGGTCATTAGTTCATAGCCCATATATGGAGTTCCGCGTTACATAACTTACGGTAAATGGCCCG
CCTGGCTGACCGCCCAACGACCCCCGCCCATTGACGTCAATAATGACGTATGTTCCCATAGTAACGCCAAT
AGGGACTTTCCATTGACGTCAATGGGTGGAGTATTTACGGTAAACTGCCCACTTGGCAGTACATCAAGTGTA
TCATATGCCAAGTACGCCCCCTATTGACGTCAATGACGGTAAATGGCCCGCCTGGCATTATGCCCAGTACAT
GACCTTATGGGACTTTCCTACTTGGCAGTACATCTACGTATTAGTCATCGCTATTACCATGGTGATGCGGTTT
TGGCAGTACATCAATGGGCGTGGATAGCGGTTTGACTCACGGGGATTTCCAAGTCTCCACCCCATTGACGT
CAATGGGAGTTTGTTTTGGCACCAAAATCAACGGGACTTTCCAAAATGTCGTAACAACTCCGCCCCATTGAC

GCAAATGGGCGGTAGGCGTGTACGGTGGGAGGTCTATATAAGCAGAGCTCTCTGGCTAACTAGAGAACCCA
CTGCTTACTGGCTTATCGAAATTAATACGACTCACTATAGGGAGACCCAAGCTGGCTAGTTAAGCTTGATCAA
ACAAGTTTGTACAAAAAAGCAGGCTTGAAGGAATTCGGTACCATGGCTCGGCGCGGCTGGCGGCGGGCAC
CCCTCCGCCGTGGCGTCGGCAGCAGTCCCCGAGCCCGCAGGCTCATGCGGCCCCTTTGGTTGCTCCTCG
CAGTGGGCGTCTTTGACTGGGCAGGGGCTTCGGACGGCGGCGGCGGAGAGGCTAGAGCCATGGACGAG
GAGATCGTGTCCGAGAAGCAAGCCGAGGAGAGCCACCGGCAGGACAGCGCCAACCTGCTCATCTTCATC
CTGCTGCTCACCCTCACCATTCTCACAATCTGGCTCTTCAAGCACCGCCGGGCCCGCTTCCTGCACGAAA
CCGGCCTGGCTATGATTTATGGTCTTTTGGTGGGCCTTGTGCTTCGGTATGGCATTCATGTTCCGAGTGATG
TAAATAATGTGACCCTGAGCTGTGAAGTGCAGTCAAGTCCAACTACCTTACTGGTTACTTTTGATCCAGAAG
TATTTTTCAACATATTACTTCCTCCTATCATATTTTATGCAGGTTATAGCCTGAAAAGGAGACATTTTTTTCGAA
ATCTTGGGTCTATCCTAGCATACGCTTTTCTTGGAACAGCAATTTCTTGTTTCGTTATTGGGTCAATAATGTAT
GGCTGTGTAACGCTGATGAAGGTAACGGGACAACTTGCAGGAGATTTTTACTTTACAGATTGCCTACTGTTT
GGTGCCATTGTATCAGCAACTGATCCAGTGACTGTTCTTGCTATATTCCACGAGCTTCAAGTTGATGTTGAA
CTCTATGCACTTCTTTTTGGTGAAAGTGTCCTCAATGATGCTGTTGCCATAGTGCTGTCCTCCTCAATAGTG
GCATACCAGCCAGCTGGAGACAACAGTCACACCTTTGATGTCACAGCGATGTTCAAGTCTATTGGGATCTT
CCTTGGAATCTTCAGTGGATCTTTTGCAATGGGTGCTGCTACTGGAGTGGTGACAGCTTTAGTGACAAAGT
TCACCAAATTACGGGAGTTCCAGTTGTTGGAGACAGGCCTGTTCTTCTTGATGTCCTGGAGTACCTTCCTCT
TGGCTGAAGCATGGGGCTTCACAGGTGTAGTTGCAGTATTGTTTTGTGGCATCACACAAGCACATTATACGT
ATAATAATTTGTCAACGGAGTCTCAGCATAGAACTAAACAGTTGTTTGAGCTTCTCAATTTCTTGGCAGAGAA
TTTCATCTTCTCCTACATGGGGCTGACACTGTTCACCTTCCAGAACCATGTCTTTAACCCAACATTTGTAGTA
GGAGCATTTGTTGCTATTTTCTTGGGAAGAGCTGCCAATATTTACCCCTTGTCCCTCTTACTTAATTTGGGTA
GAAGAAGTAAGATTGGATCAAATTTTCAACACATGATGATGTTTGCTGGCCTTCGTGGTGCAATGGCATTTG
CCTTGGCCATTCGAGATACTGCCACTTATGCACGGCAAATGATGTTCAGCACCACGCTTCTGATTGTGTTTT
TTACCGTGTGGGTATTTGGTGGTGGCACCACTGCAATGCTGTCATGCTTGCATATCAGGGTTGGTGTTGATT
CAGACCAAGAACACTTGGGTGTTCCTGAAAATGAAAGGAGAACTACCAAAGCAGAGAGTGCTTGGCTTTTC
CGGATGTGGTACAACTTTGATCATAACTATCTGAAGCCTCTGCTGACCCACAGCGGGCCTCCGCTGACAAC
AACACTCCCTGCCTGCTGTGGACCCATCGCCAGGTGCCTCACCAGCCCCCAGGCTTACGAAAACCAGGAA
CAGTTGAAAGATGATGATTCTGATCTTATTCTCAATGATGGTGACATCAGTTTGACATATGGAGATTCTACTGT
GAACACTGAACCGGCCACATCCAGCGCCCCAAGGAGATTTATGGGAAACAGTTCTGAAGATGCCTTGGATC
GGGAGCTTGCATTTGGGGACCATGAACTGGTCATTCGAGGAACACGCCTGGTTCTTCCAATGGATGATTCT
GAACCCCCGCTAAATTTGTTAGATAATACGAGACATGGTCCAGCCTACCTCGAGTGCGGCCGCATGGCTAG
CTACCCTTACGACGTCCCAGACTACGCTGGATCCTACCCTTACGACGTCCCAGACTACGCTTACCCTTACG
ACGTCCCAGACTACGCTTGATCAGCCTCGACTGTGCCTTCTAGTTGCCAGCCATCTGTTGTTTGCCCCTCC
CCCGTGCCTTCCTTGACCCTGGAAGGTGCCACTCCCACTGTCCTTTCCTAATAAAATGAGGAAATTGCATC
GCATTGTCTGAGTAGGTGTCATTCTATTCTGGGGGGTGGGGTGGGGCAGGACAGCAAGGGGGAGGATTG
GGAAGACAATAGCAGGCATGCTGGGGATGCGGTGGGCTCTATGGCTTCTGAGGCGGAAAGAACCAGCTG
GGGCTCTAGGGGGTATCCCCACGCGCCCTGTAGCGGCGCATTAAGCGCGGCGGGTGTGGTGGTTACGCG
CAGCGTGACCGCTACACTTGCCAGCGCCCTAGCGCCCGCTCCTTTCGCTTTCTTCCCTTCCTTTCTCGCCA
CGTTCGCCGGCTTTCCCCGTCAAGCTCTAAATCGGGGGCTCCCTTTAGGGTTCCGATTTAGTGCTTTACGG
CACCTCGACCCCAAAAAACTTGATTAGGGTGATGGTTCACGTAGTGGGCCATCGCCCTGATAGACGGTTTT
TCGCCCTTTGACGTTGGAGTCCACGTTCTTTAATAGTGGACTCTTGTTCCAAACTGGAACAACACTCAACCC
TATCTCGGTCTATTCTTTTGATTTATAAGGGATTTTGCCGATTTCGGCCTATTGGTTAAAAAATGAGCTGATTTA
ACAAAAATTTAACGCGAATTAATTCTGTGGAATGTGTGTCAGTTAGGGTGTGGAAAGTCCCCAGGCTCCCCA
GCAGGCAGAAGTATGCAAAGCATGCATCTCAATTAGTCAGCAACCAGGTGTGGAAAGTCCCCAGGCTCCC
CAGCAGGCAGAAGTATGCAAAGCATGCATCTCAATTAGTCAGCAACCATAGTCCCGCCCCTAACTCCGCCC
ATCCCGCCCCTAACTCCGCCCAGTTCCGCCCATTCTCCGCCCCATGGCTGACTAATTTTTTTTATTTATGCA
GAGGCCGAGGCCGCCTCTGCCTCTGAGCTATTCCAGAAGTAGTGAGGAGGCTTTTTTGGAGGCCTAGGCT
TTTGCAAAAAGCTCCCGGGAGCTTGTATATCCATTTTCGGATCTGATCAAGAGACAGGATGAGGATCGTTTC

GCATGATTGAACAAGATGGATTGCACGCAGGTTCTCCGGCCGCTTGGGTGGAGAGGCTATTCGGCTATGA
CTGGGCACAACAGACAATCGGCTGCTCTGATGCCGCCGTGTTCCGGCTGTCAGCGCAGGGGCGCCCGGT
TCTTTTTGTCAAGACCGACCTGTCCGGTGCCCTGAATGAACTGCAGGACGAGGCAGCGCGGCTATCGTGG
CTGGCCACGACGGGCGTTCCTTGCGCAGCTGTGCTCGACGTTGTCACTGAAGCGGGAAGGGACTGGCTG
CTATTGGGCGAAGTGCCGGGGCAGGATCTCCTGTCATCTCACCTTGCTCCTGCCGAGAAAGTATCCATCAT
GGCTGATGCAATGCGGCGGCTGCATACGCTTGATCCGGCTACCTGCCCATTCGACCACCAAGCGAAACAT
CGCATCGAGCGAGCACGTACTCGGATGGAAGCCGGTCTTGTCGATCAGGATGATCTGGACGAAGAGCATC
AGGGGCTCGCGCCAGCCGAACTGTTCGCCAGGCTCAAGGCGCGCATGCCCGACGGCGAGGATCTCGTC
GTGACCCATGGCGATGCCTGCTTGCCGAATATCATGGTGGAAAATGGCCGCTTTTCTGGATTCATCGACTG
TGGCCGGCTGGGTGTGGCGGACCGCTATCAGGACATAGCGTTGGCTACCCGTGATATTGCTGAAGAGCTT
GGCGGCGAATGGGCTGACCGCTTCCTCGTGCTTTACGGTATCGCCGCTCCCGATTCGCAGCGCATCGCCT
TCTATCGCCTTCTTGACGAGTTCTTCTGAGCGGGACTCTGGGGTTCGCGAAATGACCGACCAAGCGACGC
CCAACCTGCCATCACGAGATTTCGATTCCACCGCCGCCTTCTATGAAAGGTTGGGCTTCGGAATCGTTTTC
CGGGACGCCGGCTGGATGATCCTCCAGCGCGGGGATCTCATGCTGGAGTTCTTCGCCCACCCCAACTTGT
TTATTGCAGCTTATAATGGTTACAAATAAAGCAATAGCATCACAAATTTCACAAATAAAGCATTTTTTTCACTGC
ATTCTAGTTGTGGTTTGTCCAAACTCATCAATGTATCTTATCATGTCTGTATACCGTCGACCTCTAGCTAGAGC
TTGGCGTAATCATGGTCATAGCTGTTTCCTGTGTGAAATTGTTATCCGCTCACAATTCCACACAACATACGAG
CCGGAAGCATAAAGTGTAAAGCCTGGGGTGCCTAATGAGTGAGCTAACTCACATTAATTGCGTTGCGCTCA
CTGCCCGCTTTCCAGTCGGGAAACCTGTCGTGCCAGCTGCATTAATGAATCGGCCAACGCGCGGGGAGAG
GCGGTTTGCGTATTGGGCGCTCTTCCGCTTCCTCGCTCACTGACTCGCTGCGCTCGGTCGTTCGGCTGCG
GCGAGCGGTATCAGCTCACTCAAAGGCGGTAATACGGTTATCCACAGAATCAGGGGATAACGCAGGAAAGA
ACATGTGAGCAAAAGGCCAGCAAAAGGCCAGGAACCGTAAAAAGGCCGCGTTGCTGGCGTTTTTCCATAG
GCTCCGCCCCCCTGACGAGCATCACAAAAATCGACGCTCAAGTCAGAGGTGGCGAAACCCGACAGGACTA
TAAAGATACCAGGCGTTTCCCCCTGGAAGCTCCCTCGTGCGCTCTCCTGTTCCGACCCTGCCGCTTACCG
GATACCTGTCCGCCTTTCTCCCTTCGGGAAGCGTGGCGCTTTCTCATAGCTCACGCTGTAGGTATCTCAGT
TCGGTGTAGGTCGTTCGCTCCAAGCTGGGCTGTGTGCACGAACCCCCCGTTCAGCCCGACCGCTGCGCC
TTATCCGGTAACTATCGTCTTGAGTCCAACCCGGTAAGACACGACTTATCGCCACTGGCAGCAGCCACTGG
TAACAGGATTAGCAGAGCGAGGTATGTAGGCGGTGCTACAGAGTTCTTGAAGTGGTGGCCTAACTACGGCT
ACACTAGAAGAACAGTATTTGGTATCTGCGCTCTGCTGAAGCCAGTTACCTTCGGAAAAAGAGTTGGTAGCT
CTTGATCCGGCAAACAAACCACCGCTGGTAGCGGTGGTTTTTTTGTTTGCAAGCAGCAGATTACGCGCAGA
AAAAAAGGATCTCAAGAAGATCCTTTGATCTTTTCTACGGGGTCTGACGCTCAGTGGAACGAAAACTCACG
TTAAGGGATTTTGGTCATGAGATTATCAAAAAGGATCTTCACCTAGATCCTTTTAAATTAAAAATGAAGTTTTAA
ATCAATCTAAAGTATATATGAGTAAACTTGGTCTGACAGTTACCAATGCTTAATCAGTGAGGCACCTATCTCAG
CGATCTGTCTATTTCGTTCATCCATAGTTGCCTGACTCCCCGTCGTGTAGATAACTACGATACGGGAGGGCT
TACCATCTGGCCCCAGTGCTGCAATGATACCGCGAGACCCACGCTCACCGGCTCCAGATTTATCAGCAATA
AACCAGCCAGCCGGAAGGGCCGAGCGCAGAAGTGGTCCTGCAACTTTATCCGCCTCCATCCAGTCTATTA
ATTGTTGCCGGGAAGCTAGAGTAAGTAGTTCGCCAGTTAATAGTTTGCGCAACGTTGTTGCCATTGCTACAG
GCATCGTGGTGTCACGCTCGTCGTTTGGTATGGCTTCATTCAGCTCCGGTTCCCAACGATCAAGGCGAGTT
ACATGATCCCCCATGTTGTGCAAAAAAGCGGTTAGCTCCTTCGGTCCTCCGATCGTTGTCAGAAGTAAGTT
GGCCGCAGTGTTATCACTCATGGTTATGGCAGCACTGCATAATTCTCTTACTGTCATGCCATCCGTAAGATG
CTTTTCTGTGACTGGTGAGTACTCAACCAAGTCATTCTGAGAATAGTGTATGCGGCGACCGAGTTGCTCTTG
CCCGGCG

Seq_hNHE7-HA

ACGGGATAATACCGCGCCACATAGCAGAACTTTAAAAGTGCTCATCATTGGAAAACGTTCTTCGGGGCGAA
AACTCTCAAGGATCTTACCGCTGTTGAGATCCAGTTCGATGTAACCCACTCGTGCACCCAACTGATCTTCAG

CATCTTTTACTTTCACCAGCGTTTCTGGGTGAGCAAAAACAGGAAGGCAAAATGCCGCAAAAAAGGGAATA
AGGGCGACACGGAAATGTTGAATACTCATACTCTTCCTTTTTCAATATTATTGAAGCATTTATCAGGGTTATTG
TCTCATGAGCGGATACATATTTGAATGTATTTAGAAAAATAAACAAATAGGGGTTCCGCGCACATTTCCCCGA
AAAGTGCCACCTGACGTCGACGGATCGGGAGATCTCCCGATCCCCTATGGTGCACTCTCAGTACAATCTGC
TCTGATGCCGCATAGTTAAGCCAGTATCTGCTCCCTGCTTGTGTGTTGGAGGTCGCTGAGTAGTGCGCGAG
CAAAATTTAAGCTACAACAAGGCAAGGCTTGACCGACAATTGCATGAAGAATCTGCTTAGGGTTAGGCGTTT
TGCGCTGCTTCGCGATGTACGGGCCAGATATACGCGTTGACATTGATTATTGACTAGTTATTAATAGTAATCA
ATTACGGGGTCATTAGTTCATAGCCCATATATGGAGTTCCGCGTTACATAACTTACGGTAAATGGCCCGCCTG
GCTGACCGCCCAACGACCCCCGCCCATTGACGTCAATAATGACGTATGTTCCCATAGTAACGCCAATAGGG
ACTTTCCATTGACGTCAATGGGTGGAGTATTTACGGTAAACTGCCCACTTGGCAGTACATCAAGTGTATCATA
TGCCAAGTACGCCCCCTATTGACGTCAATGACGGTAAATGGCCCGCCTGGCATTATGCCCAGTACATGACC
TTATGGGACTTTCCTACTTGGCAGTACATCTACGTATTAGTCATCGCTATTACCATGGTGATGCGGTTTTGGC
AGTACATCAATGGGCGTGGATAGCGGTTTGACTCACGGGGATTTCCAAGTCTCCACCCCATTGACGTCAAT
GGGAGTTTGTTTTGGCACCAAAATCAACGGGACTTTCCAAAATGTCGTAACAACTCCGCCCCATTGACGCA
AATGGGCGGTAGGCGTGTACGGTGGGAGGTCTATATAAGCAGAGCTCTCTGGCTAACTAGAGAACCCACTG
CTTACTGGCTTATCGAAATTAATACGACTCACTATAGGGAGACCCAAGCTGGCTAGTTAAGCTTGATCAAACA
AGTTTGTACAAAAAAGCAGGCTTGAAGGAATTCGGTACCATGGAGCCTGGCGATGCCGCCAGGCCAGGAA
GCGGAAGGGCAACCGGAGCACCCCCTCCAAGGCTGCTGCTGCCACTGCTGCTGGGATGGGGACTG
AGAGTGGCAGCAGCAGCCTCCGCCAGCTCCTCTGGCGCCGCCGCCGAGGATAGCTCCGCCATGGAGGA
GCTGGCCACCGAGAAGGAGGCCGAGGAGTCTCACAGGCAGGACTCTGTGAGCCTGCTGACATTCATCCT
GCTGCTGACCCTGACAATCCTGACCATCTGGCTGTTTAAGCACCGGAGAGTGCGCTTCCTGCACGAGACC
GGACTGGCAATGATCTACGGCCTGATCGTGGGCGTGATCCTGAGATATGGAACCCCAGCCACATCCGGAA
GGGATAAGTCCCTGTCTTGCACCCAGGAGGACAGGGCCTTTTCCACACTGCTGGTGAACGTGTCTGGCAA
GTTCTTTGAGTACACACTGAAGGGCGAGATCTCTCCCGGCAAGATCAACAGCGTGGAGCAGAATGATATGC
TGCGCAAGGTGACCTTCGACCCTGAGGTGTTCTTTAATATCCTGCTGCCCCCTATCATCTTTCACGCCGGCT
ACTCCCTGAAGAAGCGGCACTTCTTTAGAAACCTGGGCTCTATCCTGGCCTATGCCTTTCTGGGCACAGCC
GTGAGCTGCTTCATCATCGGCAATCTGATGTACGGCGTGGTGAAGCTGATGAAGATCATGGGCCAGCTGTC
CGATAAGTTCTACTATACCGACTGTCTGTTCTTTGGCGCCATCATCTCTGCCACCGACCCCGTGACAGTGCT
GGCCATCTTCAACGAGCTGCACGCCGACGTGGATCTGTATGCCCTGCTGTTTGGCGAGAGCGTGCTGAAT
GATGCCGTGGCCATCGTGCTGTCTAGCTCCATCGTGGCATACCAGCCTGCAGGCCTGAACACCCACGCCT
TCGACGCAGCCGCCTTCTTTAAGTCCGTGGGCATCTTCCTGGGCATCTTTAGCGGCTCCTTCACCATGGGC
GCCGTGACAGGCGTGAACGCCAATGTGACCAAGTTTACAAAGCTGCACTGTTTCCCTCTGCTGGAGACCG
CCCTGTTCTTTCTGATGTCTTGGAGCACATTTCTGCTGGCAGAGGCATGCGGCTTCACCGGAGTGGTGGC
CGTGCTGTTTTGTGGCATCACCCAGGCCCACTACACATATAACAATCTGAGCGTGGAGTCCAGGTCTCGCA
CAAAGCAGCTGTTTGAGGTGCTGCACTTCCTGGCCGAGAATTTCATCTTTAGCTATATGGGCCTGGCCCTG
TTCACCTTTCAGAAGCACGTGTTTTCCCCTATCTTCATCATCGGCGCCTTTGTGGCAATCTTCCTGGGAAGG
GCAGCACACATCTACCCACTGTCCTTCTTTCTGAACCTGGGCAGGCGCCACAAGATCGGCTGGAATTTTCA
GCACATGATGATGTTCAGCGGACTGAGAGGAGCAATGGCCTTCGCCCTGGCAATCAGGGATACAGCCTCC
TATGCCAGACAGATGATGTTTACCACAACCCTGCTGATCGTGTTCTTTACCGTGTGGATCATCGGCGGCGG
CACAACCCCAATGCTGTCCTGGCTGAACATCAGGGTGGGAGTGGAGGAGCCATCTGAGGAGGATCAGAAT
GAGCACCACTGGCAGTACTTCAGAGTGGGAGTGGACCCAGATCAGGACCCACCACCTAACAATGACTCTT
TTCAGGTGCTGCAGGGCGATGGACCAGACAGCGCCAGGGGAAACAGAACAAAGCAGGAGAGCGCCTGG
ATCTTTCGGCTGTGGTACTCCTTCGATCACAATTATCTGAAGCCTATCCTGACCCACAGCGGCCCACCCCTG
ACAACCACACTGCCAGCATGGTGCGGACTGCTGGCAAGGTGTCTGACATCTCCCCAGGTGTATGACAACC
AGGAGCCTCTGCGCGAGGAGGATAGCGACTTCATCCTGACCGAGGGCGATCTGACCCTGACATACGGCG
ACTCTACCGTGACAGCCAATGGCTCTAGCTCCTCTCACACCGCAAGCACATCCCTGGAGGGAAGCCGGAG
AACAAAGAGCTCCTCTGAGGAGGTGCTGGAGCGGATCTGGGCATGGGCGACCAGAAGGTGAGCTCCAG
GGGAACCAGGCTGGTGTTCCCACTGGAGGACAACGCCTACCTCGAGTGCGGCCGCATGGCTAGCTACCC

TTACGACGTCCCAGACTACGCTGGATCCTACCCTTACGACGTCCCAGACTACGCTTACCCTTACGACGTCC
CAGACTACGCTTGATCAGCCTCGACTGTGCCTTCTAGTTGCCAGCCATCTGTTGTTTGCCCCTCCCCCGTG
CCTTCCTTGACCCTGGAAGGTGCCACTCCCACTGTCCTTTCCTAATAAAATGAGGAAATTGCATCGCATTGT
CTGAGTAGGTGTCATTCTATTCTGGGGGGTGGGGTGGGGCAGGACAGCAAGGGGGAGGATTGGGAAGAC
AATAGCAGGCATGCTGGGGATGCGGTGGGCTCTATGGCTTCTGAGGCGGAAAGAACCAGCTGGGGCTCTA
GGGGGTATCCCCACGCGCCCTGTAGCGGCGCATTAAGCGCGGCGGGTGTGGTGGTTACGCGCAGCGTGA
CCGCTACACTTGCCAGCGCCCTAGCGCCCGCTCCTTTCGCTTTCTTCCCCTCCTTTCTCGCCACGTTCGCC
GGCTTTCCCCGTCAAGCTCTAAATCGGGGGCTCCCTTTAGGGTTCCGATTTAGTGCTTTACGGCACCTCGA
CCCCAAAAAACTTGATTAGGGTGATGGTTCACGTAGTGGGCCATCGCCCTGATAGACGGTTTTTCGCCCTT
TGACGTTGGAGTCCACGTTCTTTAATAGTGGACTCTTGTTCCAAACTGGAACAACACTCAACCCTATCTCGG
TCTATTCTTTTGATTTATAAGGGATTTTGCCGATTTCGGCCTATTGGTTAAAAAATGAGCTGATTTAACAAAAAT
TTAACGCGAATTAATTCTGTGGAATGTGTGTCAGTTAGGGTGTGGAAAGTCCCCAGGCTCCCCAGCAGGCA
GAAGTATGCAAAGCATGCATCTCAATTAGTCAGCAACCAGGTGTGGAAAGTCCCCAGGCTCCCCAGCAGG
CAGAAGTATGCAAAGCATGCATCTCAATTAGTCAGCAACCATAGTCCCGCCCCTAACTCCGCCCATCCCGC
CCCTAACTCCGCCCAGTTCCGCCCATTCTCCGCCCCATGGCTGACTAATTTTTTTTATTTATGCAGAGGCCG
AGGCCGCCTCTGCCTCTGAGCTATTCCAGAAGTAGTGAGGAGGCTTTTTTGGAGGCCTAGGCTTTTGCAAA
AAGCTCCCGGGAGCTTGTATATCCATTTTCGGATCTGATCAAGAGACAGGATGAGGATCGTTTCGCATGATT
GAACAAGATGGATTGCACGCAGGTTCTCCGGCCGCTTGGGTGGAGAGGCTATTCGGCTATGACTGGGCAC
AACAGACAATCGGCTGCTCTGATGCCGCCGTGTTCCGGCTGTCAGCGCAGGGGCGCCCGGTTCTTTTTGT
CAAGACCGACCTGTCCGGTGCCCTGAATGAACTGCAGGACGAGGCAGCGCGGCTATCGTGGCTGGCCAC
GACGGGCGTTCCTTGCGCAGCTGTGCTCGACGTTGTCACTGAAGCGGGAAGGGACTGGCTGCTATTGGG
CGAAGTGCCGGGGCAGGATCTCCTGTCATCTCACCTTGCTCCTGCCGAGAAAGTATCCATCATGGCTGATG
CAATGCGGCGGCTGCATACGCTTGATCCGGCTACCTGCCCATTCGACCACCAAGCGAAACATCGCATCGA
GCGAGCACGTACTCGGATGGAAGCCGGTCTTGTCGATCAGGATGATCTGGACGAAGAGCATCAGGGGCTC
GCGCCAGCCGAACTGTTCGCCAGGCTCAAGGCGCGCATGCCCGACGGCGAGGATCTCGTCGTGACCCAT
GGCGATGCCTGCTTGCCGAATATCATGGTGGAAAATGGCCGCTTTTCTGGATTCATCGACTGTGGCCGGCT
GGGTGTGGCGGACCGCTATCAGGACATAGCGTTGGCTACCCGTGATATTGCTGAAGAGCTTGGCGGCGAA
TGGGCTGACCGCTTCCTCGTGCTTTACGGTATCGCCGCTCCCGATTCGCAGCGCATCGCCTTCTATCGCCT
TCTTGACGAGTTCTTCTGAGCGGGACTCTGGGGTTCGCGAAATGACCGACCAAGCGACGCCCAACCTGCC
ATCACGAGATTTCGATTCCACCGCCGCCTTCTATGAAAGGTTGGGCTTCGGAATCGTTTTCCGGGACGCCG
GCTGGATGATCCTCCAGCGCGGGGATCTCATGCTGGAGTTCTTCGCCCACCCCAACTTGTTTATTGCAGCT
TATAATGGTTACAAATAAAGCAATAGCATCACAAATTTCACAAATAAAGCATTTTTTTCACTGCATTCTAGTTGT
GGTTTGTCCAAACTCATCAATGTATCTTATCATGTCTGTATACCGTCGACCTCTAGCTAGAGCTTGGCGTAAT
CATGGTCATAGCTGTTTCCTGTGTGAAATTGTTATCCGCTCACAATTCCACACAACATACGAGCCGGAAGCA
TAAAGTGTAAAGCCTGGGGTGCCTAATGAGTGAGCTAACTCACATTAATTGCGTTGCGCTCACTGCCCGCTT
TCCAGTCGGGAAACCTGTCGTGCCAGCTGCATTAATGAATCGGCCAACGCGCGGGGAGAGGCGGTTTGC
GTATTGGGCGCTCTTCCGCTTCCTCGCTCACTGACTCGCTGCGCTCGGTCGTTCGGCTGCGGCGAGCGG
TATCAGCTCACTCAAAGGCGGTAATACGGTTATCCACAGAATCAGGGGATAACGCAGGAAAGAACATGTGA
GCAAAAGGCCAGCAAAAGGCCAGGAACCGTAAAAAGGCCGCGTTGCTGGCGTTTTTCCATAGGCTCCGCC
CCCCTGACGAGCATCACAAAAATCGACGCTCAAGTCAGAGGTGGCGAAACCCGACAGGACTATAAAGATAC
CAGGCGTTTCCCCCTGGAAGCTCCCTCGTGCGCTCTCCTGTTCCGACCCTGCCGCTTACCGGATACCTGT
CCGCCTTTCTCCCTTCGGGAAGCGTGGCGCTTTCTCATAGCTCACGCTGTAGGTATCTCAGTTCGGTGTAG
GTCGTTCGCTCCAAGCTGGGCTGTGTGCACGAACCCCCCGTTCAGCCCGACCGCTGCGCCTTATCCGGTA
ACTATCGTCTTGAGTCCAACCCGGTAAGACACGACTTATCGCCACTGGCAGCAGCCACTGGTAACAGGATT
AGCAGAGCGAGGTATGTAGGCGGTGCTACAGAGTTCTTGAAGTGGTGGCCTAACTACGGCTACACTAGAA
GAACAGTATTTGGTATCTGCGCTCTGCTGAAGCCAGTTACCTTCGGAAAAAGAGTTGGTAGCTCTTGATCCG
GCAAACAAACCACCGCTGGTAGCGGTGGTTTTTTTGTTTGCAAGCAGCAGATTACGCGCAGAAAAAAAGGA
TCTCAAGAAGATCCTTTGATCTTTTCTACGGGGTCTGACGCTCAGTGGAACGAAAACTCACGTTAAGGGATT

TTGGTCATGAGATTATCAAAAAGGATCTTCACCTAGATCCTTTTAAATTAAAAATGAAGTTTTAAATCAATCTAA
AGTATATATGAGTAAACTTGGTCTGACAGTTACCAATGCTTAATCAGTGAGGCACCTATCTCAGCGATCTGTC
TATTTCGTTCATCCATAGTTGCCTGACTCCCCGTCGTGTAGATAACTACGATACGGGAGGGCTTACCATCTG
GCCCCAGTGCTGCAATGATACCGCGAGACCCACGCTCACCGGCTCCAGATTTATCAGCAATAAACCAGCCA
GCCGGAAGGGCCGAGCGCAGAAGTGGTCCTGCAACTTTATCCGCCTCCATCCAGTCTATTAATTGTTGCCG
GGAAGCTAGAGTAAGTAGTTCGCCAGTTAATAGTTTGCGCAACGTTGTTGCCATTGCTACAGGCATCGTGGT
GTCACGCTCGTCGTTTGGTATGGCTTCATTCAGCTCCGGTTCCCAACGATCAAGGCGAGTTACATGATCCC
CCATGTTGTGCAAAAAAGCGGTTAGCTCCTTCGGTCCTCCGATCGTTGTCAGAAGTAAGTTGGCCGCAGTG
TTATCACTCATGGTTATGGCAGCACTGCATAATTCTCTTACTGTCATGCCATCCGTAAGATGCTTTTCTGTGA
CTGGTGAGTACTCAACCAAGTCATTCTGAGAATAGTGTATGCGGCGACCGAGTTGCTCTTGCCCGGCGTCA
AT

Seq_mNHE9-HA

CAACTGATCTTCAGCATCTTTTACTTTCACCAGCGTTTCTGGGTGAGCAAAAACAGGAAGGCAAAATGCCG
CAAAAAAGGGAATAAGGGCGACACGGAAATGTTGAATACTCATACTCTTCCTTTTTCAATATTATTGAAGCATT
TATCAGGGTTATTGTCTCATGAGCGGATACATATTTGAATGTATTTAGAAAAATAAACAAATAGGGGTTCCGCG
CACATTTCCCCGAAAAGTGCCACCTGACGTCGACGGATCGGGAGATCTCCCGATCCCCTATGGTGCACTCT
CAGTACAATCTGCTCTGATGCCGCATAGTTAAGCCAGTATCTGCTCCCTGCTTGTGTGTTGGAGGTCGCTG
AGTAGTGCGCGAGCAAAATTTAAGCTACAACAAGGCAAGGCTTGACCGACAATTGCATGAAGAATCTGCTTA
GGGTTAGGCGTTTTGCGCTGCTTCGCGATGTACGGGCCAGATATACGCGTTGACATTGATTATTGACTAGTT
ATTAATAGTAATCAATTACGGGGTCATTAGTTCATAGCCCATATATGGAGTTCCGCGTTACATAACTTACGGTA
AATGGCCCGCCTGGCTGACCGCCCAACGACCCCCGCCCATTGACGTCAATAATGACGTATGTTCCCATAGT
AACGCCAATAGGGACTTTCCATTGACGTCAATGGGTGGAGTATTTACGGTAAACTGCCCACTTGGCAGTACA
TCAAGTGTATCATATGCCAAGTACGCCCCCTATTGACGTCAATGACGGTAAATGGCCCGCCTGGCATTATGC
CCAGTACATGACCTTATGGGACTTTCCTACTTGGCAGTACATCTACGTATTAGTCATCGCTATTACCATGGTG
ATGCGGTTTTGGCAGTACATCAATGGGCGTGGATAGCGGTTTGACTCACGGGGATTTCCAAGTCTCCACCC
CATTGACGTCAATGGGAGTTTGTTTTGGCACCAAAATCAACGGGACTTTCCAAAATGTCGTAACAACTCCGC
CCCATTGACGCAAATGGGCGGTAGGCGTGTACGGTGGGAGGTCTATATAAGCAGAGCTCTCTGGCTAACTA
GAGAACCCACTGCTTACTGGCTTATCGAAATTAATACGACTCACTATAGGGAGACCCAAGCTGGCTAGTTAA
GCTTGATCAAACAAGTTTGTACAAAAAAGCAGGCTTGAAGGAATTCGGTACCATGGCTGGGCAGCTTCGGT
TTACGTCAGGGAAGGATGAAGATCATTTTCAACACCAGGGAGCAGTGGAGCTACTCGCGTTTAATTTTTTGC
TCATCCTTACCATTTTAACAATCTGGTTATTTAAAAATCACCGATTCCGCTTCTTGCATGAGACTGGAGGGGC
GATGGTGTATGGCCTCATAATGGGACTAATTTTGCGATATGCCACAGCGCCGACTGATATTGATAGTGGAACT
GTCTACAACTGTGGGAACTTGTTCTTCAGCCCATCAACTCTACTAGTCAACATCACCGACCAAGTTTATGAG
TATAAGTACCAGAGAGAGATCAACCAGCACAATATCAGCCCCCACCAAGGCAACGCCATACTGGAGAAGAT
GACGTTTGACCCAGAAATCTTCTTCAACGTTTTGCTGCCTCCCATCATATTTCATGCAGGATACAGTCTGAA
GAAGAGACACTTTTTTCAAAACTTGGGATCCATTTTAACGTATGCCTTCTTGGGAACAGCCATCTCCTGTGT
GGTCATAGGGTTAATTATGTACGGCTTTGTGAAAGCCATGGTGCATGCTGGCCAGCTGAAGAGCGGAGACT
TCCACTTCACTGACTGTTTATTTTTTGGTTCACTGATGTCTGCTACAGATCCAGTGACAGTGCTGGCCATTTT
CCATGAACTCCACGTGGACCCAGACCTGTACACACTGCTGTTTGGAGAGAGTGTACTGAATGATGCGGTG
GCCATAGTCCTCACATACTCTATATCCATCTATAGTCCCAAGGAGAATCCCAACGCGTTTGACACGGCAGCG
TTCTTCCAATCCGTGGGCAATTTCCTAGGGATCTTTGCCGGCTCCTTTGCAATGGGCTCCGCATATGCAGTT
GTTACCGCACTGTTGACCAAATTTACCAAGCTCCGTGAGTTCCCGATGCTGGAGACAGGTCTGTTTTTCCT
CCTTTCTTGGAGTGCCTTCCTGTCTGCAGAGGCCGCCGGCTTAACAGGCATAGTTGCTGTTCTCTTCTGTG
GAGTCACACAGGCACATTACACCTACAACAATCTGTCATCGGATTCCAAACTGAGGACCAAACAGCTGTTT
GAATTTATGAACTTCCTGGCTGAAAACGTCATCTTCTGCTACATGGGCCTGGCTCTGTTCACCTTCCAGAAT

CACATTTTCAATGCTCTTTTTATACTCGGAGCCTTTCTAGCAATTTTTGTTGCCAGAGCCTGCAACATTTACC
CCCTCTCCTTCCTCCTGAATCTGGGCAGAAAGCAGAAGATTCCCTGGAACTTCCAGCACATGATGATGTTTT
CAGGTTTGCGAGGTGCCATTGCCTTCGCCCTGGCCATCCGCAACACGGAATCTCAGCCCAAACAGATGAT
GTTCACCACCACACTGCTCCTTGTGTTCTTCACTGTCTGGGTGTTTGGAGGAGGAACGACCCCCATGCTG
ACGTGGCTTCAGATCAGAGTTGGTGTGGACCTGGATGAAAGTCTGAAGGAAGAGCCCTCCTCACAGCAGG
AAGCAAATAAGTTGGACAAAAACATGACGAAGACAGAGAGTGCACAGCTCTTCAGAATGTGGTATGGATTT
GACCACAAATACCTGAAACCCATCCTAACACACTCAGGCCCTCCACTGACCACCACCTTGCCAGCGTGGTG
CGGCCCAGTCTCCAGGCTGCTCACCAGTCCGCAGGCCTATGGGGAACAACTGAAAGAGGATGACGTGGA
ATGTATCGTCAACCAAGATGAACTGGCCATGAATTACCAGGAGCAGAGCCCTTCACCCAGCAGTCCCACCA
CCAAGCTAGCTCTGGACCAGAAGTCTTCAGGCCAGACTCCAGGCAAGGAGAATATCTACGAGGGAGATCT
CGGCCTGGGAGGCTATGATCTCAAACTGGAGCAGACTCGGGGTCAACCCCAGATGGACTACCTCGAGTGC
GGCCGCATGGCTAGCTACCCTTACGACGTCCCAGACTACGCTGGATCCTACCCTTACGACGTCCCAGACTA
CGCTTACCCTTACGACGTCCCAGACTACGCTTGATCAGCCTCGACTGTGCCTTCTAGTTGCCAGCCATCTG
TTGTTTGCCCCTCCCCCGTGCCTTCCTTGACCCTGGAAGGTGCCACTCCCACTGTCCTTTCCTAATAAAAT
GAGGAAATTGCATCGCATTGTCTGAGTAGGTGTCATTCTATTCTGGGGGGTGGGGTGGGGCAGGACAGCA
AGGGGGAGGATTGGGAAGACAATAGCAGGCATGCTGGGGATGCGGTGGGCTCTATGGCTTCTGAGGCGG
AAAGAACCAGCTGGGGCTCTAGGGGGTATCCCCACGCGCCCTGTAGCGGCGCATTAAGCGCGGCGGGTG
TGGTGGTTACGCGCAGCGTGACCGCTACACTTGCCAGCGCCCTAGCGCCCGCTCCTTTCGCTTTCTTCCC
TTCCTTTCTCGCCACGTTCGCCGGCTTTCCCCGTCAAGCTCTAAATCGGGGGCTCCCTTTAGGGTTCCGAT
TTAGTGCTTTACGGCACCTCGACCCCAAAAAACTTGATTAGGGTGATGGTTCACGTAGTGGGCCATCGCCC
TGATAGACGGTTTTTCGCCCTTTGACGTTGGAGTCCACGTTCTTTAATAGTGGACTCTTGTTCCAAACTGGA
ACAACACTCAACCCTATCTCGGTCTATTCTTTTGATTTATAAGGGATTTTGCCGATTTCGGCCTATTGGTTAAA
AAATGAGCTGATTTAACAAAAATTTAACGCGAATTAATTCTGTGGAATGTGTGTCAGTTAGGGTGTGGAAAGT
CCCCAGGCTCCCCAGCAGGCAGAAGTATGCAAAGCATGCATCTCAATTAGTCAGCAACCAGGTGTGGAAA
GTCCCCAGGCTCCCCAGCAGGCAGAAGTATGCAAAGCATGCATCTCAATTAGTCAGCAACCATAGTCCCGC
CCCTAACTCCGCCCATCCCGCCCCTAACTCCGCCCAGTTCCGCCCATTCTCCGCCCCATGGCTGACTAATT
TTTTTTATTTATGCAGAGGCCGAGGCCGCCTCTGCCTCTGAGCTATTCCAGAAGTAGTGAGGAGGCTTTTTT
GGAGGCCTAGGCTTTTGCAAAAAGCTCCCGGGAGCTTGTATATCCATTTTCGGATCTGATCAAGAGACAGG
ATGAGGATCGTTTCGCATGATTGAACAAGATGGATTGCACGCAGGTTCTCCGGCCGCTTGGGTGGAGAGG
CTATTCGGCTATGACTGGGCACAACAGACAATCGGCTGCTCTGATGCCGCCGTGTTCCGGCTGTCAGCGC
AGGGGCGCCCGGTTCTTTTTGTCAAGACCGACCTGTCCGGTGCCCTGAATGAACTGCAGGACGAGGCAG
CGCGGCTATCGTGGCTGGCCACGACGGGCGTTCCTTGCGCAGCTGTGCTCGACGTTGTCACTGAAGCGG
GAAGGGACTGGCTGCTATTGGGCGAAGTGCCGGGGCAGGATCTCCTGTCATCTCACCTTGCTCCTGCCGA
GAAAGTATCCATCATGGCTGATGCAATGCGGCGGCTGCATACGCTTGATCCGGCTACCTGCCCATTCGACC
ACCAAGCGAAACATCGCATCGAGCGAGCACGTACTCGGATGGAAGCCGGTCTTGTCGATCAGGATGATCT
GGACGAAGAGCATCAGGGGCTCGCGCCAGCCGAACTGTTCGCCAGGCTCAAGGCGCGCATGCCCGACG
GCGAGGATCTCGTCGTGACCCATGGCGATGCCTGCTTGCCGAATATCATGGTGGAAAATGGCCGCTTTTCT
GGATTCATCGACTGTGGCCGGCTGGGTGTGGCGGACCGCTATCAGGACATAGCGTTGGCTACCCGTGATA
TTGCTGAAGAGCTTGGCGGCGAATGGGCTGACCGCTTCCTCGTGCTTTACGGTATCGCCGCTCCCGATTC
GCAGCGCATCGCCTTCTATCGCCTTCTTGACGAGTTCTTCTGAGCGGGACTCTGGGGTTCGCGAAATGAC
CGACCAAGCGACGCCCAACCTGCCATCACGAGATTTCGATTCCACCGCCGCCTTCTATGAAAGGTTGGGC
TTCGGAATCGTTTTCCGGGACGCCGGCTGGATGATCCTCCAGCGCGGGGATCTCATGCTGGAGTTCTTCG
CCCACCCCAACTTGTTTATTGCAGCTTATAATGGTTACAAATAAAGCAATAGCATCACAAATTTCACAAATAAA
GCATTTTTTTCACTGCATTCTAGTTGTGGTTTGTCCAAACTCATCAATGTATCTTATCATGTCTGTATACCGTC
GACCTCTAGCTAGAGCTTGGCGTAATCATGGTCATAGCTGTTTCCTGTGTGAAATTGTTATCCGCTCACAATT
CCACACAACATACGAGCCGGAAGCATAAAGTGTAAAGCCTGGGGTGCCTAATGAGTGAGCTAACTCACATT
AATTGCGTTGCGCTCACTGCCCGCTTTCCAGTCGGGAAACCTGTCGTGCCAGCTGCATTAATGAATCGGCC
AACGCGCGGGGAGAGGCGGTTTGCGTATTGGGCGCTCTTCCGCTTCCTCGCTCACTGACTCGCTGCGCT

CGGTCGTTCGGCTGCGGCGAGCGGTATCAGCTCACTCAAAGGCGGTAATACGGTTATCCACAGAATCAGG
GGATAACGCAGGAAAGAACATGTGAGCAAAAGGCCAGCAAAAGGCCAGGAACCGTAAAAAGGCCGCGTTG
CTGGCGTTTTTCCATAGGCTCCGCCCCCCTGACGAGCATCACAAAAATCGACGCTCAAGTCAGAGGTGGC
GAAACCCGACAGGACTATAAAGATACCAGGCGTTTCCCCCTGGAAGCTCCCTCGTGCGCTCTCCTGTTCC
GACCCTGCCGCTTACCGGATACCTGTCCGCCTTTCTCCCTTCGGGAAGCGTGGCGCTTTCTCATAGCTCAC
GCTGTAGGTATCTCAGTTCGGTGTAGGTCGTTCGCTCCAAGCTGGGCTGTGTGCACGAACCCCCCGTTCA
GCCCGACCGCTGCGCCTTATCCGGTAACTATCGTCTTGAGTCCAACCCGGTAAGACACGACTTATCGCCAC
TGGCAGCAGCCACTGGTAACAGGATTAGCAGAGCGAGGTATGTAGGCGGTGCTACAGAGTTCTTGAAGTG
GTGGCCTAACTACGGCTACACTAGAAGAACAGTATTTGGTATCTGCGCTCTGCTGAAGCCAGTTACCTTCG
GAAAAAGAGTTGGTAGCTCTTGATCCGGCAAACAAACCACCGCTGGTAGCGGTGGTTTTTTTGTTTGCAAG
CAGCAGATTACGCGCAGAAAAAAAGGATCTCAAGAAGATCCTTTGATCTTTTCTACGGGGTCTGACGCTCA
GTGGAACGAAAACTCACGTTAAGGGATTTTGGTCATGAGATTATCAAAAAGGATCTTCACCTAGATCCTTTTA
AATTAAAAATGAAGTTTTAAATCAATCTAAAGTATATATGAGTAAACTTGGTCTGACAGTTACCAATGCTTAATC
AGTGAGGCACCTATCTCAGCGATCTGTCTATTTCGTTCATCCATAGTTGCCTGACTCCCCGTCGTGTAGATA
ACTACGATACGGGAGGGCTTACCATCTGGCCCCAGTGCTGCAATGATACCGCGAGACCCACGCTCACCGG
CTCCAGATTTATCAGCAATAAACCAGCCAGCCGGAAGGGCCGAGCGCAGAAGTGGTCCTGCAACTTTATCC
GCCTCCATCCAGTCTATTAATTGTTGCCGGGAAGCTAGAGTAAGTAGTTCGCCAGTTAATAGTTTGCGCAAC
GTTGTTGCCATTGCTACAGGCATCGTGGTGTCACGCTCGTCGTTTGGTATGGCTTCATTCAGCTCCGGTTC
CCAACGATCAAGGCGAGTTACATGATCCCCCATGTTGTGCAAAAAAGCGGTTAGCTCCTTCGGTCCTCCGA
TCGTTGTCAGAAGTAAGTTGGCCGCAGTGTTATCACTCATGGTTATGGCAGCACTGCATAATTCTCTTACTGT
CATGCCATCCGTAAGATGCTTTTCTGTGACTGGTGAGTACTCAACCAAGTCATTCTGAGAATAGTGTATGCG
GCGACCGAGTTGCTCTTGCCCGGCGTCAATACGGGATAATACCGCGCCACATAGCAGAACTTTAAAAGTGC
TCATCATTGGAAAACGTTCTTCGGGGCGAAAACTCTCAAGGATCTTACCGCTGTTGAGATCCAGTTCGATGT
AACCCACTCGTGCACC

Seq_mNHE9-GFP

TTGCCCGGCGTCAATACGGGATAATACCGCGCCACATAGCAGAACTTTAAAAGTGCTCATCATTGGAAAACG
TTCTTCGGGGCGAAAACTCTCAAGGATCTTACCGCTGTTGAGATCCAGTTCGATGTAACCCACTCGTGCAC
CCAACTGATCTTCAGCATCTTTTACTTTCACCAGCGTTTCTGGGTGAGCAAAAACAGGAAGGCAAAATGCC
GCAAAAAAGGGAATAAGGGCGACACGGAAATGTTGAATACTCATACTCTTCCTTTTTCAATATTATTGAAGCA
TTTATCAGGGTTATTGTCTCATGAGCGGATACATATTTGAATGTATTTAGAAAAATAAACAAATAGGGGTTCCG
CGCACATTTCCCCGAAAAGTGCCACCTGACGTCGACGGATCGGGAGATCTCCCGATCCCCTATGGTGCAC
TCTCAGTACAATCTGCTCTGATGCCGCATAGTTAAGCCAGTATCTGCTCCCTGCTTGTGTGTTGGAGGTCGC
TGAGTAGTGCGCGAGCAAAATTTAAGCTACAACAAGGCAAGGCTTGACCGACAATTGCATGAAGAATCTGC
TTAGGGTTAGGCGTTTTGCGCTGCTTCGCGATGTACGGGCCAGATATACGCGTTGACATTGATTATTGACTA
GTTATTAATAGTAATCAATTACGGGGTCATTAGTTCATAGCCCATATATGGAGTTCCGCGTTACATAACTTACG
GTAAATGGCCCGCCTGGCTGACCGCCCAACGACCCCCGCCCATTGACGTCAATAATGACGTATGTTCCCAT
AGTAACGCCAATAGGGACTTTCCATTGACGTCAATGGGTGGAGTATTTACGGTAAACTGCCCACTTGGCAGT
ACATCAAGTGTATCATATGCCAAGTACGCCCCCTATTGACGTCAATGACGGTAAATGGCCCGCCTGGCATTA
TGCCCAGTACATGACCTTATGGGACTTTCCTACTTGGCAGTACATCTACGTATTAGTCATCGCTATTACCATG
GTGATGCGGTTTTGGCAGTACATCAATGGGCGTGGATAGCGGTTTGACTCACGGGGATTTCCAAGTCTCCA
CCCCATTGACGTCAATGGGAGTTTGTTTTGGCACCAAAATCAACGGGACTTTCCAAAATGTCGTAACAACTC
CGCCCCATTGACGCAAATGGGCGGTAGGCGTGTACGGTGGGAGGTCTATATAAGCAGAGCTCTCTGGCTA
ACTAGAGAACCCACTGCTTACTGGCTTATCGAAATTAATACGACTCACTATAGGGAGACCCAAGCTGGCTAG
TTAAGCTGAGCATCAACAAGTTTGTACAAAAAAGCAGGCTCCGAATTCGCCCTTGCGAAGTAGCAACAAAG
TAGCAGCGTTTATTCTGAAATTCTGTGGGCGCCAGTCTATCCTAGGCAACCCTCATCTAAGGGCTCATCCCT

TGTCCAGGGATTTCTCTGAGCATCACAGTCGGTCGAGTGGAGAATGGCTGGGCAGCTTCGGTTTACGTCA
GGGAAGGATGAAGATCATTTTCAACACCAGGGAGCAGTGGAGCTACTCGCGTTTAATTTTTTGCTCATCCTT
ACCATTTTAACAATCTGGTTATTTAAAAATCACCGATTCCGCTTCTTGCATGAGACTGGAGGGGCGATGGTG
TATGGCCTCATAATGGGACTAATTTTGCGATATGCCACAGCGCCGACTGATATTGATAGTGGAACTGTCTACA
ACTGTGGGAACTTGTTCTTCAGCCCATCAACTCTACTAGTCAACATCACCGACCAAGTTTATGAGTATAAGTA
CCAGAGAGAGATCAACCAGCACAATATCAGCCCCCACCAAGGCAACGCCATACTGGAGAAGATGACGTTTG
ACCCAGAAATCTTCTTCAACGTTTTGCTGCCTCCCATCATATTTCATGCAGGATACAGTCTGAAGAAGAGAC
ACTTTTTTCAAAACTTGGGATCCATTTTAACGTATGCCTTCTTGGGAACAGCCATCTCCTGTGTGGTCATAGG
GTTAATTATGTACGGCTTTGTGAAAGCCATGGTGCATGCTGGCCAGCTGAAGAGCGGAGACTTCCACTTCA
CTGACTGTTTATTTTTTGGTTCACTGATGTCTGCTACAGATCCAGTGACAGTGCTGGCCATTTTCCATGAACT
CCACGTGGACCCAGACCTGTACACACTGCTGTTTGGAGAGAGTGTACTGAATGATGCGGTGGCCATAGTC
CTCACATACTCTATATCCATCTATAGTCCCAAGGAGAATCCCAACGCGTTTGACACGGCAGCGTTCTTCCAAT
CCGTGGGCAATTTCCTAGGGATCTTTGCCGGCTCCTTTGCAATGGGCTCCGCATATGCAGTTGTTACCGCA
CTGTTGACCAAATTTACCAAGCTCCGTGAGTTCCCGATGCTGGAGACAGGTCTGTTTTTCCTCCTTTCTTGG
AGTGCCTTCCTGTCTGCAGAGGCCGCCGGCTTAACAGGCATAGTTGCTGTTCTCTTCTGTGGAGTCACACA
GGCACATTACACCTACAACAATCTGTCATCGGATTCCAAACTGAGGACCAAACAGCTGTTTGAATTTATGAA
CTTCCTGGCTGAAAACGTCATCTTCTGCTACATGGGCCTGGCTCTGTTCACCTTCCAGAATCACATTTTCAA
TGCTCTTTTTATACTCGGAGCCTTTCTAGCAATTTTTGTTGCCAGAGCCTGCAACATTTACCCCCTCTCCTTC
CTCCTGAATCTGGGCAGAAAGCAGAAGATTCCCTGGAACTTCCAGCACATGATGATGTTTTCAGGTTTGCG
AGGTGCCATTGCCTTCGCCCTGGCCATCCGCAACACGGAATCTCAGCCCAAACAGATGATGTTCACCACCA
CACTGCTCCTTGTGTTCTTCACTGTCTGGGTGTTTGGAGGAGGAACGACCCCCATGCTGACGTGGCTTCA
GATCAGAGTTGGTGTGGACCTGGATGAAAGTCTGAAGGAAGAGCCCTCCTCACAGCAGGAAGCAAATAAG
TTGGACAAAAACATGACGAAGACAGAGAGTGCACAGCTCTTCAGAATGTGGTATGGATTTGACCACAAATAC
CTGAAACCCATCCTAACACACTCAGGCCCTCCACTGACCACCACCTTGCCAGCGTGGTGCGGCCCAGTCT
CCAGGCTGCTCACCAGTCCGCAGGCCTATGGGGAACAACTGAAAGAGGATGACGTGGAATGTATCGTCAA
CCAAGATGAACTGGCCATGAATTACCAGGAGCAGAGCCCTTCACCCAGCAGTCCCACCACCAAGCTAGCT
CTGGACCAGAAGTCTTCAGGCCAGACTCCAGGCAAGGAGAATATCTACGAGGGAGATCTCGGCCTGGGAG
GCTATGATCTCAAACTGGAGCAGACTCGGGGTCAACCCCAGATGGACAAGGGCGAATTCGACCCAGCTTT
CTTGTACAAAGTGGTTGATGCTGTTAACATGGTGAGCAAGGGCGAGGAGCTGTTCACCGGGGTGGTGCCC
ATCCTGGTCGAGCTGGACGGCGACGTAAACGGCCACAAGTTCAGCGTGTCCGGCGAGGGCGAGGGCGAT
GCCACCTACGGCAAGCTGACCCTGAAGTTCATCTGCACCACCGGCAAGCTGCCCGTGCCCTGGCCCACC
CTCGTGACCACCTTCACCTACGGCGTGCAGTGCTTCGCCCGCTACCCCGACCACATGAAGCAGCACGACT
TCTTCAAGTCCGCCATGCCCGAAGGCTACGTCCAGGAGCGCACCATCTTCTTCAAGGACGACGGCAACTA
CAAGACCCGCGCCGAGGTGAAGTTCGAGGGCGACACCCTGGTGAACCGCATCGAGCTGAAGGGCATCGA
CTTCAAGGAGGACGGCAACATCCTGGGGCACAAGCTGGAGTACAACTACAACAGCCACAAGGTCTATATCA
CCGCCGACAAGCAGAAGAACGGCATCAAGGTGAACTTCAAGACCCGCCACAACATCGAGGACGGCAGCG
TGCAGCTCGCCGACCACTACCAGCAGAACACCCCCATCGGCGACGGCCCCGTGCTGCTGCCCGACAACC
ACTACCTGAGCACCCAGTCCGCCCTGAGCAAAGACCCCAACGAGAAGCGCGATCACATGGTCCTGCTGGA
GTTCGTGACCGCCGCCGGGATCACTCTCGGCATGGACGAGCTGTACAAGTAATGATAAGTTTAAACGGGG
GAGGCTAACTGAAACACGGAAGGAGACAATACCGGAAGGAACCCGCGCTATGACGGCAATAAAAAGACAG
AATAAAACGCACGGGTGTTGGGTCGTTTGTTCATAAACGCGGGGTTCGGTCCCAGGGCTGGCACTCTGTC
GATACCCCACCGAGACCCCATTGGGGCCAATACGCCCGCGTTTCTTCCTTTTCCCCACCCCACCCCCCAA
GTTCGGGTGAAGGCCCAGGGCTCGCAGCCAACGTCGGGGCGGCAGGCCCTGCCATAGCAGATCTGCGC
AGCTGGGGCTCTAGGGGGTATCCCCACGCGCCCTGTAGCGGCGCATTAAGCGCGGCGGGTGTGGTGGTT
ACGCGCAGCGTGACCGCTACACTTGCCAGCGCCCTAGCGCCCGCTCCTTTCGCTTTCTTCCCTTCCTTTCT
CGCCACGTTCGCCGGCTTTCCCCGTCAAGCTCTAAATCGGGGGCTCCCTTTAGGGTTCCGATTTAGTGCTT
TACGGCACCTCGACCCCAAAAAACTTGATTAGGGTGATGGTTCACGTAGTGGGCCATCGCCCTGATAGACG
GTTTTTCGCCCTTTGACGTTGGAGTCCACGTTCTTTAATAGTGGACTCTTGTTCCAAACTGGAACAACACTC

AACCCTATCTCGGTCTATTCTTTTGATTTATAAGGGATTTTGCCGATTTCGGCCTATTGGTTAAAAAATGAGCT
GATTTAACAAAAATTTAACGCGAATTAATTCTGTGGAATGTGTGTCAGTTAGGGTGTGGAAAGTCCCCAGGC
TCCCCAGCAGGCAGAAGTATGCAAAGCATGCATCTCAATTAGTCAGCAACCAGGTGTGGAAAGTCCCCAG
GCTCCCCAGCAGGCAGAAGTATGCAAAGCATGCATCTCAATTAGTCAGCAACCATAGTCCCGCCCCTAACT
CCGCCCATCCCGCCCCTAACTCCGCCCAGTTCCGCCCATTCTCCGCCCCATGGCTGACTAATTTTTTTTATT
TATGCAGAGGCCGAGGCCGCCTCTGCCTCTGAGCTATTCCAGAAGTAGTGAGGAGGCTTTTTTGGAGGCC
TAGGCTTTTGCAAAAAGCTCCCGGGAGCTTGTATATCCATTTTCGGATCTGATCAGCACGTGTTGACAATTAA
TCATCGGCATAGTATATCGGCATAGTATAATACGACAAGGTGAGGAACTAAACCATGGCCAAGCCTTTGTCTC
AAGAAGAATCCACCCTCATTGAAAGAGCAACGGCTACAATCAACAGCATCCCCATCTCTGAAGACTACAGC
GTCGCCAGCGCAGCTCTCTCTAGCGACGGCCGCATCTTCACTGGTGTCAATGTATATCATTTTACTGGGGG
ACCTTGTGCAGAACTCGTGGTGCTGGGCACTGCTGCTGCTGCGGCAGCTGGCAACCTGACTTGTATCGTC
GCGATCGGAAATGAGAACAGGGGCATCTTGAGCCCCTGCGGACGGTGCCGACAGGTGCTTCTCGATCTG
CATCCTGGGATCAAAGCCATAGTGAAGGACAGTGATGGACAGCCGACGGCAGTTGGGATTCGTGAATTGC
TGCCCTCTGGTTATGTGTGGGAGGGCTAAGCACTTCGTGGCCGAGGAGCAGGACTGACACGTGCTACGA
GATTTCGATTCCACCGCCGCCTTCTATGAAAGGTTGGGCTTCGGAATCGTTTTCCGGGACGCCGGCTGGAT
GATCCTCCAGCGCGGGGATCTCATGCTGGAGTTCTTCGCCCACCCCAACTTGTTTATTGCAGCTTATAATGG
TTACAAATAAAGCAATAGCATCACAAATTTCACAAATAAAGCATTTTTTTCACTGCATTCTAGTTGTGGTTTGT
CCAAACTCATCAATGTATCTTATCATGTCTGTATACCGTCGACCTCTAGCTAGAGCTTGGCGTAATCATGGTC
ATAGCTGTTTCCTGTGTGAAATTGTTATCCGCTCACAATTCCACACAACATACGAGCCGGAAGCATAAAGTG
TAAAGCCTGGGGTGCCTAATGAGTGAGCTAACTCACATTAATTGCGTTGCGCTCACTGCCCGCTTTCCAGT
CGGGAAACCTGTCGTGCCAGCTGCATTAATGAATCGGCCAACGCGCGGGGAGAGGCGGTTTGCGTATTGG
GCGCTCTTCCGCTTCCTCGCTCACTGACTCGCTGCGCTCGGTCGTTCGGCTGCGGCGAGCGGTATCAGC
TCACTCAAAGGCGGTAATACGGTTATCCACAGAATCAGGGGATAACGCAGGAAAGAACATGTGAGCAAAAG
GCCAGCAAAAGGCCAGGAACCGTAAAAAGGCCGCGTTGCTGGCGTTTTTCCATAGGCTCCGCCCCCCTGA
CGAGCATCACAAAAATCGACGCTCAAGTCAGAGGTGGCGAAACCCGACAGGACTATAAAGATACCAGGCG
TTTCCCCCTGGAAGCTCCCTCGTGCGCTCTCCTGTTCCGACCCTGCCGCTTACCGGATACCTGTCCGCCT
TTCTCCCTTCGGGAAGCGTGGCGCTTTCTCATAGCTCACGCTGTAGGTATCTCAGTTCGGTGTAGGTCGTT
CGCTCCAAGCTGGGCTGTGTGCACGAACCCCCCGTTCAGCCCGACCGCTGCGCCTTATCCGGTAACTATC
GTCTTGAGTCCAACCCGGTAAGACACGACTTATCGCCACTGGCAGCAGCCACTGGTAACAGGATTAGCAG
AGCGAGGTATGTAGGCGGTGCTACAGAGTTCTTGAAGTGGTGGCCTAACTACGGCTACACTAGAAGAACAG
TATTTGGTATCTGCGCTCTGCTGAAGCCAGTTACCTTCGGAAAAAGAGTTGGTAGCTCTTGATCCGGCAAAC
AAACCACCGCTGGTAGCGGTGGTTTTTTTGTTTGCAAGCAGCAGATTACGCGCAGAAAAAAAGGATCTCAA
GAAGATCCTTTGATCTTTTCTACGGGGTCTGACGCTCAGTGGAACGAAAACTCACGTTAAGGGATTTTGGT
CATGAGATTATCAAAAAGGATCTTCACCTAGATCCTTTTAAATTAAAAATGAAGTTTTAAATCAATCTAAAGTAT
ATATGAGTAAACTTGGTCTGACAGTTACCAATGCTTAATCAGTGAGGCACCTATCTCAGCGATCTGTCTATTT
CGTTCATCCATAGTTGCCTGACTCCCCGTCGTGTAGATAACTACGATACGGGAGGGCTTACCATCTGGCCC
CAGTGCTGCAATGATACCGCGAGACCCACGCTCACCGGCTCCAGATTTATCAGCAATAAACCAGCCAGCCG
GAAGGGCCGAGCGCAGAAGTGGTCCTGCAACTTTATCCGCCTCCATCCAGTCTATTAATTGTTGCCGGGAA
GCTAGAGTAAGTAGTTCGCCAGTTAATAGTTTGCGCAACGTTGTTGCCATTGCTACAGGCATCGTGGTGTCA
CGCTCGTCGTTTGGTATGGCTTCATTCAGCTCCGGTTCCCAACGATCAAGGCGAGTTACATGATCCCCCAT
GTTGTGCAAAAAAGCGGTTAGCTCCTTCGGTCCTCCGATCGTTGTCAGAAGTAAGTTGGCCGCAGTGTTAT
CACTCATGGTTATGGCAGCACTGCATAATTCTCTTACTGTCATGCCATCCGTAAGATGCTTTTCTGTGACTGG
TGAGTACTCAACCAAGTCATTCTGAGAATAGTGTATGCGGCGACCGAGTTGCTC

Seq_Flag-hGGA3
GCAATAAACCAGCCAGCCGGAAGGGCCGAGCGCAGAAGTGGTCCTGCAACTTTATCCGCCTCCATCCAGT<br>CTATTAATTGTTGCCGGGAAGCTAGAGTAAGTAGTTCGCCAGTTAATAGTTTGCGCAACGTTGTTGCCATTG<br>CTACAGGCATCGTGGTGTCACGCTCGTCGTTTGGTATGGCTTCATTCAGCTCCGGTTCCCAACGATCAAGG<br>CGAGTTACATGATCCCCCATGTTGTGCAAAAAAGCGGTTAGCTCCTTCGGTCCTCCGATCGTTGTCAGAAG<br>TAAGTTGGCCGCAGTGTTATCACTCATGGTTATGGCAGCACTGCATAATTCTCTTACTGTCATGCCATCCGTA<br>AGATGCTTTTCTGTGACTGGTGAGTACTCAACCAAGTCATTCTGAGAATAGTGTATGCGGCGACCGAGTTG<br>CTCTTGCCCGGCGTCAATACGGGATAATACCGCGCCACATAGCAGAACTTTAAAAGTGCTCATCATTGGAAA<br>ACGTTCTTCGGGGCGAAAACTCTCAAGGATCTTACCGCTGTTGAGATCCAGTTCGATGTAACCCACTCGTG<br>CACCCAACTGATCTTCAGCATCTTTTACTTTCACCAGCGTTTCTGGGTGAGCAAAAACAGGAAGGCAAAATG<br>CCGCAAAAAAGGGAATAAGGGCGACACGGAAATGTTGAATACTCATACTCTTCCTTTTTCAATATTATTGAAG<br>CATTTATCAGGGTTATTGTCTCATGAGCGGATACATATTTGAATGTATTTAGAAAAATAAACAAATAGGGGTTC<br>CGCGCACATTTCCCCGAAAAGTGCCACCTGACGTCTAAGAAACCATTATTATCATGACATTAACCTATAAAAA<br>TAGGCGTATCACGAGGCCCTTTCGTCTCGCGCGTTTCGGTGATGACGGTGAAAACCTCTGACACATGCAG<br>CTCCCGGAGACGGTCACAGCTTGTCTGTAAGCGGATGCCGGGAGCAGACAAGCCCGTCAGGGCGCGTCA<br>GCGGGTGTTGGCGGGTGTCGGGGCTGGCTTAACTATGCGGCATCAGAGCAGATTGTACTGAGAGTGCACC<br>ATATGCGGTGTGAAATACCGCACAGATGCGTAAGGAGAAAATACCGCATCAGGAAATTGTAAGCGTTAATATT<br>TTGTTAAAATTCGCGTTAAATTTTTGTTAAATCAGCTCATTTTTTAACCAATAGGCCGAAATCGGCAAAATCCC<br>TTATAAATCAAAAGAATAGACCGAGATAGGGTTGAGTGTTGTTCCAGTTTGGAACAAGAGTCCACTATTAAAG<br>AACGTGGACTCCAACGTCAAAGGGCGAAAAACCGTCTATCAGGGCGATGGCCCACTACGTGAACCATCAC<br>CCTAATCAAGTTTTTTGGGGTCGAGGTGCCGTAAAGCACTAAATCGGAACCCTAAAGGGAGCCCCCGATTT<br>AGAGCTTGACGGGGAAAGCCGGCGAACGTGGCGAGAAAGGAAGGGAAGAAAGCGAAAGGAGCGGGCGC<br>TAGGGCGCTGGCAAGTGTAGCGGTCACGCTGCGCGTAACCACCACACCCGCCGCGCTTAATGCGCCGCT<br>ACAGGGCGCGTCCATTCGCCATTCAGGCTGCGCAACTGTTGGGAAGGGCGATCGGTGCGGGCCTCTTCG<br>CTATTACGCCAGCTGGCGAAAGGGGGATGTGCTGCAAGGCGATTAAGTTGGGTAACGCCAGGGTTTTCCC<br>AGTCACGACGTTGTAAAACGACGGCCAGTGAATTGTCGACTTCTGAGGCGGAAAGAACCAGCTGTGGAAT<br>GTGTGTCAGTTAGGGTGTGGAAAGTCCCCAGGCTCCCCAGCAGGCAGAAGTATGCAAAGCATGCATCTCA<br>ATTAGTCAGCAAGGAAAGTCCCCAGGCTCCCCAGCAGGCAGAAGTATGCAAAGCATGCATCTCAATTAGTC<br>AGCAACCATAGTCCCGCCCCTAACTCCGCCCATCCCGCCCCTAACTCCGCCCAGTTCCGCCCATTCTCCG<br>CCCCATGGCTGACTAATTTTTTTTATTTATGCAGAGGCCGAGGCCGCCTCGGCCTCTGAGCTATTCCAGAAG<br>TAGTGAAGAGGCTTTTTTGGAGGCCTAGGCTTTTGCAAAAAGCTCCGGATCGATCCTGAGAACTTCAGGGT<br>GAGTTTGGGGACCCTTGATTGTTCTTTCTTTTTCGCTATTGTAAAATTCATGTTATATGGAGGGGGCAAAGTT<br>TTCAGGGTGTTGTTTAGAATGGGAAGATGTCCCTTGTATCACCATGGACCCTCATGATAATTTTGTTTCTTTC<br>ACTTTCTACTCTGTTGACAACCATTGTCTCCTCTTATTTTCTTTTCATTTTCTGTAACTTTTTCGTTAAACTTTA<br>GCTTGCATTTGTAACGAATTTTTAAATTCACTTTTGTTTATTTGTCAGATTGTAAGTACTTTCTCTAATCACTTT<br>TTTTTCAAGGCAATCAGGGTATATTATATTGTACTTCAGCACAGTTTTAGAGAACAATTGTTATAATTAAATGAT<br>AAGGTAGAATATTTCTGCATATAAATTCTGGCTGGCGTGGAAATATTCTTATTGGTAGAAACAACTACATCCTG<br>GTCATCATCCTGCCTTTCTCTTTATGGTTACAATGATATACACTGTTTGAGATGAGGATAAAATACTCTGAGTC<br>CAAACCGGGCCCCTCTGCTAACCATGTTCATGCCTTCTTCTTTTTCCTACAGCTCCTGGGCAACGTGCTGG<br>TTATTGTGCTGTCTCATCATTTTGGCAAAGAATTGTAATACGACTCACTATAGGGCGAATTGTCGACCCGGGA<br>AGCTTACCATGGACTACAAGGACGACGATGACAAGCTCGATGGAGGATACCCCTACGACGTGCCCGACTAC<br>GCCAGCGGATCCATGGCGGAGGCGGAAGGGGAAAGCCTGGAGTCCTGGCTCAATAAAGCCACCAATCCT<br>TCCAACCGCCAGGAGGACTGGGAATACATAATTGGCTTCTGTGATCAGATCAACAAGGAGCTGGAAGGGC<br>CACAGATCGCCGTCCGACTGCTGGCCCACAAGATCCAGTCCCCACAGGAATGGGAGGCGCTCCAGGCCC<br>TGACGTACCTGGGGGACAGGGTGTCTGAGAAAGTGAAGACCAAGGTTATTGAGCTGCTGTACAGCTGGAC<br>CATGGCCCTGCCAGAAGAAGCAAAGATCAAAGACGCCTACCACATGCTGAAGAGACAGGGCATAGTGCAG<br>TCTGACCCACCAATTCCTGTGGATAGGACGCTGATCCCCTCTCCACCACCTCGTCCCAAAAACCCTGTTTT

TGATGATGAGGAGAAGTCCAAGCTTTTAGCCAAGCTGCTGAAAAGCAAAAACCCAGATGACCTGCAGGAG
GCCAACAAGCTCATCAAGTCCATGGTGAAGGAAGACGAGGCACGGATCCAGAAGGTGACCAAGCGTCTG
CACACGTTAGAGGAAGTTAACAACAACGTGAGACTGCTCAGTGAGATGCTGCTTCATTACAGCCAGGAGGA
CTCTTCGGACGGGGACAGAGAGCTGATGAAGGAGCTGTTTGATCAGTGTGAGAACAAGAGGCGGACTTTA
TTTAAACTCGCCAGTGAGACTGAGGACAATGATAACAGTTTGGGGGACATCCTGCAAGCCAGTGACAACCT
CTCCCGGGTCATCAACTCTTACAAAACAATTATTGAAGGGCAGGTCATCAATGGCGAGGTGGCTACCTTAAC
CCTGCCTGACTCGGAAGGAAACAGTCAGTGCAGTAACCAAGGCACGCTCATCGACCTTGCGGAGCTGGA
CACGACCAACAGTTTGTCCTCCGTGTTGGCCCCAGCACCTACTCCACCCTCCTCAGGCATCCCAATCCTCC
CTCCACCACCCCAGGCCTCAGGACCTCCACGGAGCCGCTCCTCTAGCCAGGCCGAGGCCACCCTGGGG
CCCAGCAGCACAAGCAACGCCCTCTCCTGGCTGGACGAGGAGCTACTCTGCTTGGGCCTCGCCGACCCA
GCCCCTAATGTTCCTCCCAAAGAGTCAGCTGGGAACAGCCAGTGGCACCTGCTCCAGAGGGAACAGTCC
GACCTGGACTTCTTCAGCCCCAGGCCGGGGACCGCTGCCTGTGGCGCCTCCGACGCTCCTCTGCTCCAG
CCCTCAGCCCCCTCCTCAAGCAGCTCCCAAGCTCCACTGCCGCCTCCCTTCCCAGCTCCTGTGGTCCCAG
CCAGTGTTCCTGCCCCCAGTGCGGGCTCCTCCTTGTTTTCTACTGGAGTGGCCCCAGCCTTGGCCCCAAA
AGTTGAGCCCGCAGTCCCTGGGCACCATGGCTTGGCGTTGGGCAACAGCGCGCTGCACCACCTGGATGC
CCTCGATCAGCTTCTAGAAGAGGCCAAAGTGACCTCGGGCTTGGTGAAACCCACTACCTCCCCTCTCATCC
CCACCACCACCCCAGCCAGGCCCCTCCTGCCCTTCTCCACGGGGCCCGGCAGCCCGCTCTTCCAGCCAC
TGAGTTTCCAGTCCCAGGGCAGCCCCCCGAAGGGGCCTGAGCTCTCCCTGGCCAGCATCCACGTGCCCC
TGGAATCGATCAAGCCTAGCAGTGCCCTTCCTGTGACAGCCTACGATAAAAACGGCTTCCGCATCCTCTTC
CACTTTGCCAAGGAGTGTCCCCCAGGACGACCTGACGTGCTGGTGGTGGTGGTGTCCATGCTGAACACG
GCTCCCTTACCTGTCAAGAGCATCGTGCTGCAGGCTGCAGTGCCCAAGTCAATGAAAGTGAAGCTGCAGC
CACCTTCTGGGACAGAACTCTCTCCATTTAGCCCCATCCAGCCACTTGCAGCCATCACCCAGGTCATGTTG
CTGGCCAATCCACTGAAGGAGAAGGTGCGGCTTCGGTATAAGCTGACCTTCGCCCTGGGGGAGCAGCTG
AGCACAGAGGTGGGCGAGGTGGACCAGTTCCCTCCTGTGGAACAGTGGGGGAACCTATGAGAATTCCCC
GATATCGCCTCGAGTCTAGATCCAGATCTTATTAAAGCAGAACTTGTTTATTGCAGCTTATAATGGTTACAAAT
AAAGCAATAGCATCACAAATTTCACAAATAAAGCATTTTTTTCACTGCATTCTAGTTGTGGTTTGTCCAAACTC
ATCAATGTATCTTATCATGTCTGGTCGACTCTAGACTCTTCCGCTTCCTCGCTCACTGACTCGCTGCGCTCG
GTCGTTCGGCTGCGGCGAGCGGTATCAGCTCACTCAAAGGCGGTAATACGGTTATCCACAGAATCAGGGG
ATAACGCAGGAAAGAACATGTGAGCAAAAGGCCAGCAAAAGGCCAGGAACCGTAAAAAGGCCGCGTTGCT
GGCGTTTTTCCATAGGCTCCGCCCCCCTGACGAGCATCACAAAAATCGACGCTCAAGTCAGAGGTGGCGA
AACCCGACAGGACTATAAAGATACCAGGCGTTTCCCCCTGGAAGCTCCCTCGTGCGCTCTCCTGTTCCGAC
CCTGCCGCTTACCGGATACCTGTCCGCCTTTCTCCCTTCGGGAAGCGTGGCGCTTTCTCATAGCTCACGCT
GTAGGTATCTCAGTTCGGTGTAGGTCGTTCGCTCCAAGCTGGGCTGTGTGCACGAACCCCCCGTTCAGCC
CGACCGCTGCGCCTTATCCGGTAACTATCGTCTTGAGTCCAACCCGGTAAGACACGACTTATCGCCACTGG
CAGCAGCCACTGGTAACAGGATTAGCAGAGCGAGGTATGTAGGCGGTGCTACAGAGTTCTTGAAGTGGTG
GCCTAACTACGGCTACACTAGAAGAACAGTATTTGGTATCTGCGCTCTGCTGAAGCCAGTTACCTTCGGAAA
AAGAGTTGGTAGCTCTTGATCCGGCAAACAAACCACCGCTGGTAGCGGTGGTTTTTTTGTTTGCAAGCAGC
AGATTACGCGCAGAAAAAAAGGATCTCAAGAAGATCCTTTGATCTTTTCTACGGGGTCTGACGCTCAGTGG
AACGAAAACTCACGTTAAGGGATTTTGGTCATGAGATTATCAAAAAGGATCTTCACCTAGATCCTTTTAAATTA
AAAATGAAGTTTTAAATCAATCTAAAGTATATATGAGTAAACTTGGTCTGACAGTTACCAATGCTTAATCAGTG
AGGCACCTATCTCAGCGATCTGTCTATTTCGTTCATCCATAGTTGCCTGACTCCCCGTCGTGTAGATAACTAC
GATACGGGAGGGCTTACCATCTGGCCCCAGTGCTGCAATGATACCGCGAGACCCACGCTCACCGGCTCCA
GATTTATCA

Table S6: List of primers used for making GGA1, NHE9 constructs.

Genes and domains	Forward primers	Reverse primers
mGGA1 –FL-GFP	5'- GCC GCC ATG GAG CCC GCG ATG GAG CCG GAG -3'	5'- GAG GCT CCC CCA GGT CTC TGG TGG-3'
mGGA1 –VHS-GFP	5'- GCC GCC ATG GCC ACA AAT CCC CTG AAC AAG GAG-3'	5'- CGA CTT CAC AAT CCC CTG CTT CTT CAG CAT CTG GTA-3'
mGGA1 –GAT-GFP	5'- GCC GCC ATG GAT GAG GAG AAG TCC AAG ATG CTG GCC-3'	5'- CCG GAC CAG CTG CTT GTA CAG GTT GAT CAC CTG GGT-3'
mGGA1 –GAE-GFP	5'- GCC GCC ATG GCC AGC AGC ATC TTG CCA GTG ACC GTT -3'	5'-GGG GAA CTG ATC CAC ATC TCC CAT CTC ATT GTA GGT-3'
mGGA1 –Hinge-GFP	5'- GCC GCC ATG GGC GAG GAG GTC AAC GGT GAT GCC-3'	5'- GGG TTT GAT AGA CTC CAG GGG CAC AGT GAT-3'
mGGA1 –del GAE-GFP	5'- GCC GCC ATG GAG CCC GCG ATG GAG CCG GAG -3'	5'- GGG TTT GAT AGA CTC CAG GGG CAC AGT GAT-3'
mGGA1 –del VHS-GFP	5'- GCC GCC ATG GAT GAG GAG AAG TCC AAG ATG CTG GCC-3'	5'- GAG GCT CCC CCA GGT CTC TGG TGG-3'
mGGA1 –VHS+GAT-GFP	5'- GCC GCC ATG GCC ACA AAT CCC CTG AAC AAG GAG-3'	5'- CCG GAC CAG CTG CTT GTA CAG GTT GAT CAC CTG GGT-3'
mGGA1 –Hinge+GAE-GFP	5'- GCC GCC ATG GGC GAG GAG GTC AAC GGT GAT GCC-3'	5'-GGG GAA CTG ATC CAC ATC TCC CAT CTC ATT GTA GGT-3'
mGGA1 –GAT+Hinge-GFP	5'- GCC GCC ATG GAT GAG GAG AAG TCC AAG ATG CTG GCC-3'	5'- GGG TTT GAT AGA CTC CAG GGG CAC AGT GAT-3'
mGGA1 –del Hinge-GFP	5'-AGC AGC TGG TCC GGA GCA GCA TCT TGCC-3'	5'-GGC AAG ATG CTG CTC CGG ACC AGC TGCT-3'
c-Myc- mGGA1	5'- CG GA ATT CCG ATG GAG CCC GCG ATG GAG CCG GAG AC -3'	5'- CCG CTC GAG CTA GAG GCT CCC CCA GGT CTC TGG TGG-3'
pmNHE9 GFP	5'- CAG TCG GTC GAG TGG AGA ATG-3'	5'- GTC CAT CTG GGG TTG ACC C-3'

Table S7: List of primers for making in fusion snap constructs.

Chimeric plasmids	oligoes
Snap_ NHE6N/NHE1C _oligo1	TACCTCGAGTGCGGCCGC
Snap_ NHE6N/NHE1C _oligo2	CAAGCATGACAGCATTGCAGTGG
Snap_ NHE6N/NHE1C _oligo3	ATGCTGTCATGCTTGCGGCCCCTGGTAGACCTG
Snap_ NHE6N/NHE1C _oligo4	GCCGCACTCGAGGTACTGCCCCTTGGGGAAGAACG
Snap_ NHE1N/NHE6C_oligo1	CATATCAGGGTTGGTGTTGATTCAG
Snap_ NHE1N/NHE6C_oligo2	GGTACCGAATTCCTTCAAGCCTG
Snap_ NHE1N/NHE6C_oligo3	AAGGAATTCGGTACCATGGTTCTGCGGTCTGGC
Snap_ NHE1N/NHE6C_oligo4	ACCAACCCTGATATGAATGGTCATGCCCTGCAC
hNHE5/7/9-HA-SNAP-oligo1	TACCTCGAGTGCGGCCGC
hNHE5/7/9-HA-SNAP-oligo2	GGTACCGAATTCCTTCAAGCCTGC
hNHE5-HA-SNAP-oligo3	AAGGAATTCGGTACCATGCTGAGAGCCGCCCTG
hNHE5-HA-SNAP-oligo4	GCCGCACTCGAGGTACAGCCTGGAGCCTCTGTTG
hNHE7-HA-SNAP-oligo3	AAGGAATTCGGTACCATGGAGCCTGGCGATGCC
hNHE7-HA-SNAP-oligo4	GCCGCACTCGAGGTAGGCGTTGTCCTCCAGTGGG
mNHE9-HA-SNAP-oligo3	AAGGAATTCGGTACCATGGCTGGGCAGCTTCGG
mNHE9-HA-SNAP-oligo4	GCCGCACTCGAGGTAGTCCATCTGGGGTTGACCCC
hNHE1-G720D-HA-oligo1	GTCATCACCATCGACCCGGCTTCCCCG
hNHE1-G720D-HA-oligo2	CGGGGAAGCCGGGTCGATGGTGATGAC
hNHE1-HA -SNAP-oligo1	TACCTCGAGTGCGGCCGC
hNHE1-HA -SNAP-oligo2	GGTACCGAATTCCTTCAAGCCTGC
hNHE1-HA -SNAP-oligo3	AAGGAATTCGGTACCATGGTTCTGCGGTCTGGC
hNHE1-HA -SNAP-oligo4	GCCGCACTCGAGGTACTGCCCCTTGGGGAAGAACG

Table S8: Fractionation ultracentrifuge speed and time

Fractionation	Ultracentrifuge speed	Time (hrs)
Endosome	41,600 rpm	2-3
Lysosome	145,000 g	2
Golgi	120,000 g	3
